# Site-Selective C–H Amination of Phenol-Containing Biomolecules

**DOI:** 10.1021/acs.orglett.3c01560

**Published:** 2023-06-07

**Authors:** Carlota Girón-Elola, Ibon Sasiain, Rosalía Sánchez-Fernández, Elena Pazos, Arkaitz Correa

**Affiliations:** † University of the Basque Country (UPV/EHU), Department of Organic Chemistry I, Joxe Mari Korta R&D Center, Avda. Tolosa 72, 20018 Donostia-San Sebastián, Spain,; ‡ CICA − Centro Interdisciplinar de Química e Bioloxía and Departamento de Química, Facultade de Ciencias, Universidade da Coruña, Campus de Elviña, 15071 A Coruña, Spain

## Abstract

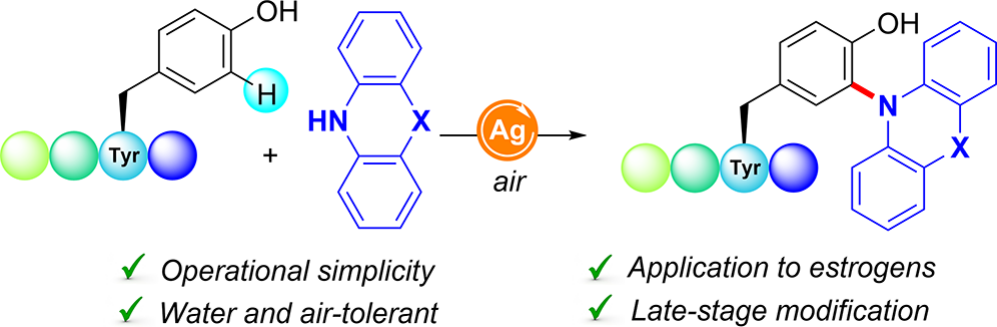

A C–N bond-forming cross-dehydrogenative coupling of a collection of Tyr-containing peptides and estrogens with heteroarenes is described. This oxidative coupling is distinguished by its scalability, operational simplicity, and air tolerance and enables the appendance of phenothiazines and phenoxazines in phenol-like compounds. When incorporated into a Tb(III) metallopeptide, the Tyr-phenothiazine moiety acts as a sensitizer for the Tb(III) ion, providing a new tool for the design of luminescent probes.

Owing to their chemical versatility, amino acids are privileged motifs in a number of disciplines such as organic synthesis, medicinal chemistry, and chemical biology. The strategical incorporation of noncanonical amino acids into a given peptide template represents a unique platform to create molecular diversity within the peptide drug discovery space.^1^ In fact, peptides housing nonproteinogenic amino acids often exhibit improved permeability and higher stability to those of their parent native analogues. As a result, the past decade has witnessed an exponential growth of the peptide therapeutics market,^2^ and the development of general methods to modify peptides in a late-stage fashion poses a challenging task of paramount importance.

Metal-catalyzed C–H functionalization reactions have recently evolved into powerful yet innovative technologies toward the site-selective modification of amino acids and peptides.^3^ In this respect, a wide range of bioconjugation methods are available today to tag highly reactive residues such as Cys, Lys, or Trp. Conversely, despite its prevalence in a myriad of medicinally relevant compounds, the modification of tyrosine (Tyr) has been comparatively overlooked.^4^ The introduction of directing groups (DGs) into the oxygen atom of the phenol ring has enabled olefination,^5^ hydroxylation,^6^ acylation,^7^ acetoxylation,^8^ and acyloxylation^9^ reactions at the *ortho-*C(sp^2^)–H bond. Although chelation assistance constitutes a common practice within the field and results in the assembly of unprecedented peptides, the cleavage of the required DG is often a low-yielding step, which deeply jeopardizes the practicality of the latter methods. Accordingly, the performance of bioconjugation reactions in native Tyr-containing peptides stands out as a streamlined and preferred avenue. In this respect, a number of elegant C-modification methods have been reported for the appendance of different functional groups including Mannich-type reactions,^10^ nitrations,^11^ and trifluoromethylations,^12^ among others. Based on the attractive features of phenothiazines,^13^ of particular importance are the methods developed by Lei^14^ and MacMillan^15^ to label Tyr derivatives upon electrochemistry and photocatalysis, respectively (Scheme 1).

**Scheme 1:**
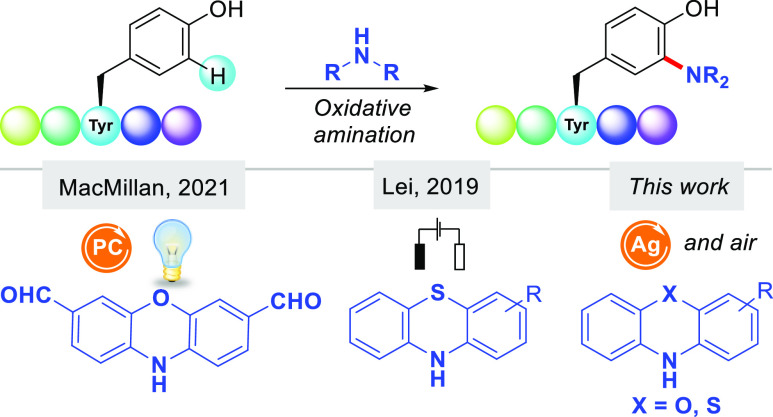
Amination of Tyr-Containing Peptides

The click-like phenol–phenothiazine coupling reaction^16^ can occur in a plethora of reaction conditions;^17^ however, its application at more complex biomolecules has been less explored. Whereas the reported amination protocols are effective to append phenothiazine^14^ and phenoxazines^15^ in Tyr-containing proteins, the requirement of sophisticated reaction devices may reduce their use in a mainstream context. Inspired by these elegant methods, we wondered whether we could perform those couplings in an operationally simple fashion without special reaction equipment. In this Letter, we unlock the prowess of silver carbonate to assist a reliable and scalable oxidative C–H amination process. This method enables the practical appendance of benzoxazines and benzothiazines into phenol-containing biomolecules, such as Tyr-containing peptides and estrogens.

We started our studies by selecting the cross-dehydrogenative coupling (CDC) of simple Boc-Tyr-OMe (**1a**) with phenothiazine as the model reaction. After some experimentation,^18^ we found that Ag_2_CO_3_ (30 mol %) in *o*-xylene under air at 90 °C provided **2aa** quantitatively (Table 1, entry 1). Control experiments underpinned the crucial role of silver carbonate^19^ and air within the reaction outcome (entries 2–3). The use of other Ag(I) salts or a lower amount of silver carbonate resulted in lower yields of **2aa** (entries 5–7). Importantly, the reaction could be performed in neat water,^18^ albeit a stoichiometric amount of Ag_2_CO_3_ and an argon atmosphere were required (entry 10). The latter evidenced the potential utility of the method in more complex Tyr-containing biomolecules, which usually require an aqueous system. Moreover, the synthesis of **2aa** could be performed with 3.0 g (15.06 mmol) of phenothiazine with an excellent 89% isolated yield (entry 9).^18^ These preliminary studies revealed that the challenging phenothiazination of a Tyr residue could be performed in a reliable practical fashion without a sophisticated setup. Notably, the parent benzoxazine as well as substituted benzothiazines could be also used as the coupling partners (Scheme 2). It is important to highlight the tolerance to alkynes and chlorides, which represent versatile reaction sites for creating molecular diversity. In accordance with previous studies in simple phenol systems,^17^ other heteroarenes such as carbazole or indoles were found unreactive under the standard conditions, thus highlighting the unique capacity of phenothiazine to form *N*-centered radicals.

**Table 1:**
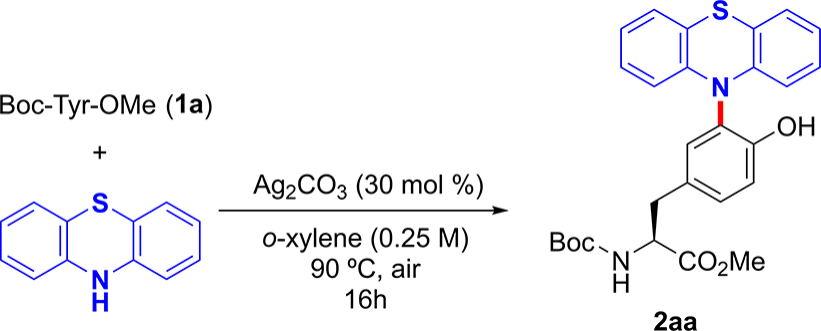
C–H Phenothiazination of **1a**^a^

entry	Change from standard conditions	**2aa** (%)^b^
**1**	**none**	**99**
2	Without Ag_2_CO_3_	0
3	Under Ar	45
4	H_2_O instead of *o*-xylene	11
5	AgOAc instead of Ag_2_CO_3_	23
6	Ag_2_O instead of Ag_2_CO_3_	47
7	Ag_2_CO_3_ (25 mol %)	76
8	At 70 °C	82
**9**	**Boc-Tyr-OMe (1.2 equiv)**	**99 (89)**^c^
**10**	**Ag**_**2**_**CO**_**3**_**(1.0 equiv) in H**_**2**_**O under Ar**	**83**

a Reaction conditions: **1a** (0.50 mmol), phenothiazine (0.25 mmol), Ag_2_CO_3_ (30 mol %), *o*-xylene (1.0 mL) at 90 °C for 16 h under air.

b Yield of isolated product after column chromatography.

c Reaction performed with 15.06 mmol of phenothiazine.

**Scheme 2:**
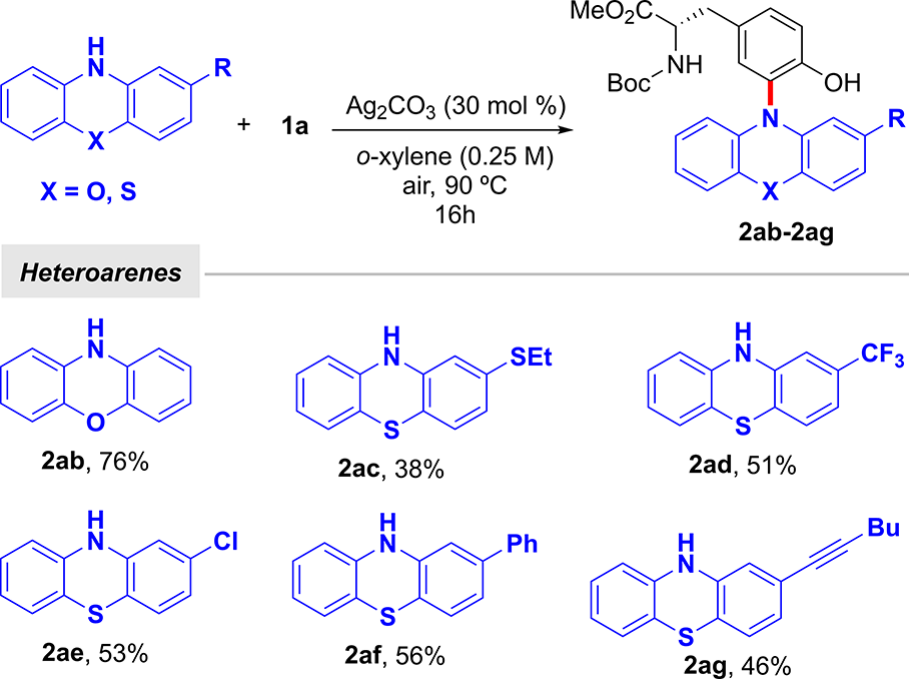
Scope of the Heteroarene^,^ Reaction conditions: **1a** (0.30 mmol), heteroarene (0.25 mmol), Ag_2_CO_3_ (30 mol %), *o*-xylene (1.0 mL) at 90 °C for 16 h under air. Yield of isolated product after column chromatography, average of at least two independent runs.

With the optimized conditions in hand, we next investigated the preparative scope of the method to assemble a new family of decorated Tyr-containing peptides in a simple fashion (Scheme 3). A wide variety of dipeptides underwent the corresponding C–N bond-forming coupling in good to excellent yields. Remarkably, phenothiazine was efficiently installed at dipeptides housing Leu (**4a**), Phe (**4b**), Ala (**4c**–**d**), Ile (**4e**), Pro (**4f**), Ser (**4g**), Thr (**4h**), Met (**4i**), Asp (**4j**), Glu (**4k**), Lys (**4l**), Arg (**4m**), and Trp (**4n**–**o**). Of particular importance are compounds incorporating oxidizable functional groups such as hydroxyl, thioether, and indole, which remained intact along the process. It is noteworthy that the performance of the process in water in the presence of 1.0 equiv of Ag_2_CO_3_ ushered in higher yields for certain highly polar dipeptides such as those incorporating Pro (**4f**), Thr (**4h**), and Asp (**4j**), which may be due to solubility issues. Interestingly, more challenging tri- and tetrapeptides (**4p**–**x**) could be efficiently tagged with phenothiazine. Importantly, tetrapeptides **4w** and **4x** bearing the amino acid sequence of biologically relevant *Endomorphin-1* and *Endomorphin-2*, respectively, were also labeled in a late-stage fashion. Other Tyr derived from naturally occurring compounds or active pharmaceuticals including those derived from palmitic acid (**6a**), oleic acid (**6b**), ibuprofen (**6d**), and artificial sweetener neotame (**6g**) smoothly underwent the corresponding amination reaction (Scheme 4). Likewise, non-natural Tyr residues incorporating adamantane (**6c**), photoswitchable diazobenzene (**6e**), and carboxamide (**6f**) site-selectively underwent our amination manifold. In order to demonstrate the synthetic utility of the method, we further explored its use toward the late-stage modification of estrogens, such as estrone and estradiol. The coupling of estrone with phenothiazines preferentially occurred at the C2 position, although variable amounts of the parent isomer substituted at the C4 were also formed (Scheme 4).

**Scheme 3:**
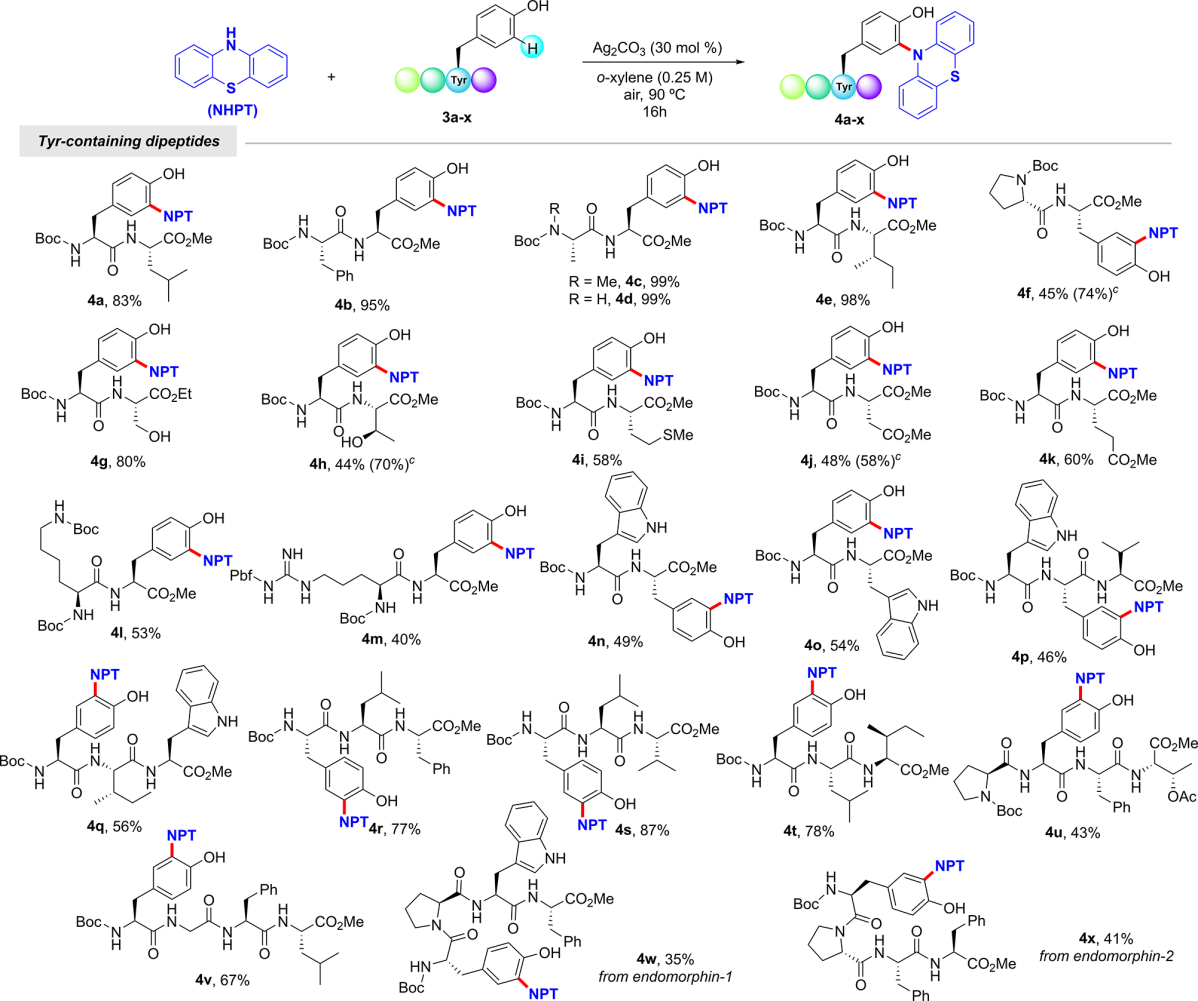
C–H Phenothiazination of Tyr-Containing Peptides^,^ Reaction conditions: **3** (0.30 mmol), phenothiazine (0.25 mmol), Ag_2_CO_3_ (30 mol %), *o*-xylene (1.0 mL) at 90 °C for 16 h under air. Yield of isolated product after column chromatography, average of at least two independent runs with no more than 5% variation in yield between runs. **3** (0.30 mmol), phenothiazine (0.25 mmol), Ag_2_CO_3_ (1.0 equiv), H_2_O (1.0 mL) at 90 °C for 16 h under Ar.

**Scheme 4:**
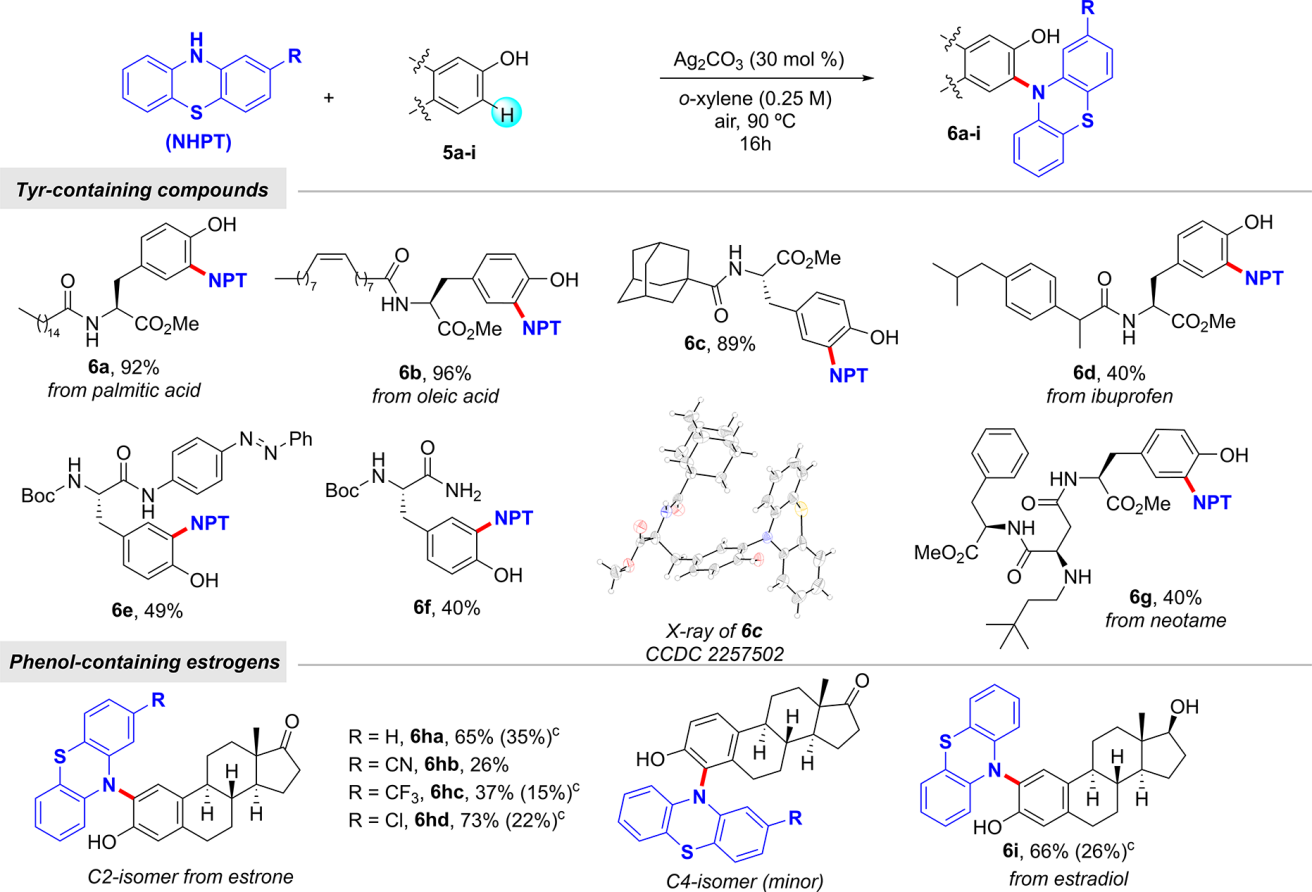
C–H Phenothiazination of Tyr-Containing Compounds and Estrogens^,^ Reaction conditions: **5** (0.30 mmol), phenothiazine (0.25 mmol), Ag_2_CO_3_ (30 mol %), *o*-xylene (1.0 mL) at 90 °C for 16 h under air. Yield of isolated product after column chromatography, average of at least two independent runs with no more than 5% variation in yield between runs. Yield of the minor C4-functionalized derivative.

In order to gain some insight into the reaction mechanism, some experiments were conducted. The presence of a free-hydroxyl group within the Tyr was found key as Tyr housing OTs, OAc, or OMe groups remained intact (Table S4).^18^ The addition of BHT as a radical trap ushered in the entire inhibition of the process. Conversely, the addition of TEMPO resulted in slightly lower yields of **2aa** (68% vs 99%), but the process was not entirely shut down (Table S3).^18^ This reactivity pattern has been observed in similar processes,^16,17^ and some authors have suggested that TEMPO may prolong the lifetime of the transient *N*-centered radical species upon the formation of covalent intermediates.^17c,17d,19^ Accordingly,^20^ we assumed that the process would start with the Ag-assisted formation of an electrophilic *N*-centered radical **I** at the phenothiazine (Scheme S1).^18^ The latter could likely be trapped by the phenol ring of the Tyr in a polarity-matched fashion to yield radical intermediate **II** that would eventually evolve into the target product through further oxidation to **III** and aromatization (Scheme S1, *path a*). However, a radical–radical coupling between the phenothiazine radical I and the phenoxy radical **IV** derived from the starting phenol has been often proposed and cannot be ruled out at this stage (*path b*).^20^ The key role of air could be attributed to the reoxidation of the *in situ* formed Ag(0) species.

Considering the potential use of the Tyr(NPT) moiety as a fluorophore^14,15^ and the attractive luminescent properties of lanthanide ions, i.e., long lifetimes in the order of milli-seconds and narrow emission bands in the visible and near-infrared region,^21^ we wondered whether the Tyr(NPT) unit could be used as an antenna for lanthanide ions, and therefore synthesized peptide **7[Tb]** (Figure 1A).^18,22^ As expected, compared to parent Tyr analogue **8**, peptide **7** exhibited red-shifted fluorescence with an emission band centered at 446 nm (Figure 1B). Interestingly, the time-gated emission spectrum of the metallopeptide **7[Tb]** showed the characteristic emission bands of the Tb(III) ion at 489, 544, 586, and 620 nm, for the corresponding transitions ^5^D_4_ → ^7^F_*J*_, *J* = 6, 5, 4, 3, confirming that the Tyr(NPT) chromophore is an adequate Tb(III) antenna.

**Figure 1:**
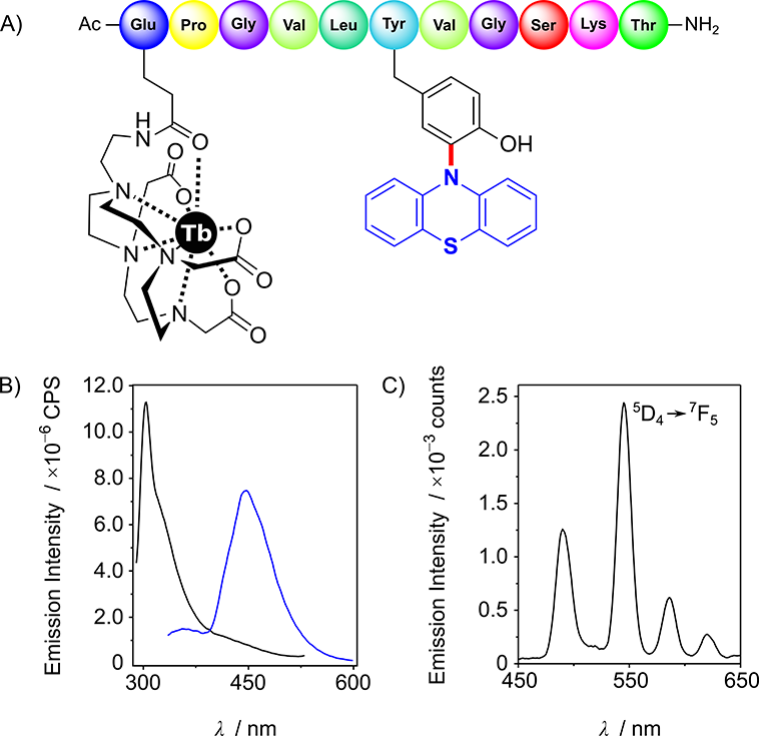
(A) Peptide sequence of **7[Tb]**. (B) Fluorescence spectra of 2 μM **7** (blue line) and **8** (black line) in 10 mM HEPES, 100 mM NaCl, pH 7.5, excitation 254 and 274 nm, respectively. (C) Luminescence spectra of 2 μM **7[Tb]** in 10 mM HEPES, 100 mM NaCl, pH 7.5, excitation 254 nm.

In summary, we have developed a CDC of Tyr-containing peptides with phenothiazines and phenoxazines. Salient features of this method are the scalability, operational simplicity, tolerance to air and water, and application for the late-stage modification of estrogens. In addition, we have shown that the Tyr(NPT) moiety can be used as an antenna for Tb(III) ions, providing a new tool for the design of luminescent probes for biologically relevant targets.

## Data Availability

The data underlying this study are available in the published article and its Supporting Information.

## Supporting Information Available

Experimental procedures, syntheses and characterization of all new compounds, and tables with details of several optimization studies (PDF)
