# Supplementary material for: Site-Selective C–H Amination of Phenol-Containing Biomolecules
Source: Org Lett. 2023 Jun 7;25(23):4383–7. doi: 10.1021/acs.orglett.3c01560 (PMC10278169; doi:10.1021/acs.orglett.3c01560)

Supporting Information

Site-Selective C–H Amination of Phenol-Containing Biomolecules

Carlota Girón-Elola,[a] Ibon Sasiain,[a] Rosalía Sánchez-Fernández,[b]
*Elena Pazos[b] and Arkaitz Correa[a]**
[a]University of the Basque Country (UPV/EHU), Department of Organic Chemistry I, Joxe Mari Korta R&D Center, Avda. Tolosa 72, 20018 Donostia-San Sebastián (Spain)
E-mail: arkaitz.correa@ehu.eus
[b]CICA – Centro Interdisciplinar de Química e Bioloxía and Departamento de Química, Facultade de Ciencias, Universidade da Coruña, Campus de Elviña, 15071 A Coruña (Spain)

1.-General Considerations **S2**

2.-Optimization Details **S3**

3.-Synthesis of the Starting Materials **S6**

4.- C(sp^2)-H Amination of Tyr-Containing Compounds **S15**

5.-Gram-Scale Experiments **S45**

6.- Peptide 7[Tb] Synthesis and Spectroscopic Characterization **S46**

7.-Control Experiments and Mechanism Proposal **S52**

8.-X-Ray Crystallography **S54**

9.- ^{1}H NMR, ^{13}C NMR and ^{19}F NMR Spectra **S59**

1.-General Considerations

Reagents. Commercially available materials were used without further purification. Silver carbonate (99.5% metal basis) and *o*-xylene (HPLC grade) were purchased from *Alfa Aesar*. Boc-Tyr-OMe was purchased from *Apollo Scientific*. Unless otherwise indicated, phenothiazines and phenoxazines were purchased from *TCI* and were used without further purification.

Analytical Methods. ^{1}H NMR and ^{13}C NMR spectra as well as HRMS and melting points (where applicable) are included for all new compounds. ^{1}H NMR and ^{13}C NMR spectra were recorded on a Bruker 400 or 500 MHz at 80 °C, unless otherwise indicated. All ^{1}H NMR spectra are reported in parts per million (ppm) downfield of TMS and were measured relative to the signals for $CHCl_3$ (7.26 ppm), unless otherwise indicated. All ^{13}C NMR spectra were reported in ppm relative to residual $CHCl_3$ (77 ppm), unless otherwise indicated, and were obtained with ^{1}H decoupling. Coupling constants, *J*, are reported in Hertz. Melting points were measured using open glass capillaries in a Büchi SMP-20 apparatus. High resolution mass spectra (HRMS) were performed by SGIker and were acquired on a LC/Q-TOF mass spectrometer equipped with an electrospray source ESI Agilent Jet Stream. Flash chromatography was performed with EM Science silica gel 60 (230-400 mesh). The yields reported in the manuscript correspond to isolated yields and represent an average of at least two independent runs.

2.-Optimization Details

General Procedure:

A round-bottom flask containing a stirring bar was charged with Boc-Tyr-OMe (0.50 mmol, 148 mg), phenothiazine (0.25 mmol, 50 mg) and the corresponding metal source. The reaction tube was then evacuated and back-filled with dry argon (this sequence was repeated up to three times). Then, the corresponding solvent (1.0 mL) was added by syringe under argon atmosphere. The reaction tube was next warmed up to the corresponding temperature in a heating block and stirred for 16 hours. The mixture was allowed to cool to room temperature, and concentrated under reduced pressure. The resulting crude was purified by flash chromatography (hexanes/EtOAc, 7/3). The purity of the corresponding product **2aa** was verified by ^{1}H NMR.

Table S1. Screening of phenothiazination of Boc-Tyr-OMe[a]

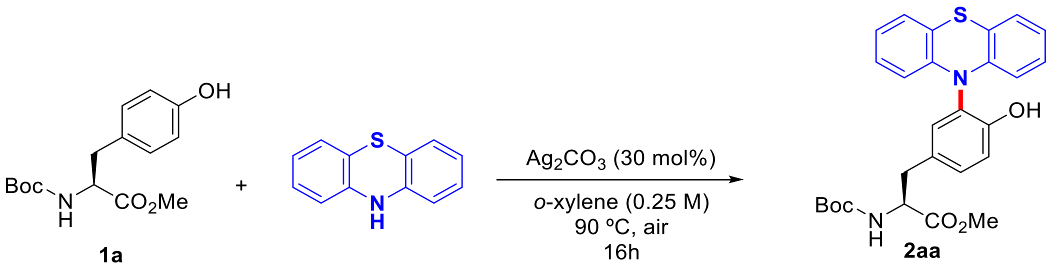

Entry	Variation from the standard conditions	2aa (%)[b]
1	none	99
2	Without Ag_2CO_3	0
3	Under Ar	45
4	H_2O instead of *o*-xylene	11
5	*o*-xylene: H_2O (1:1) as solvent	39
6	*o*-xylene: H_2O (7:3) as solvent	48
7	toluene instead of *o*-xylene	95
8	MeCN instead of *o*-xylene	traces
9	At 70 ºC	82
10	At 50 ºC	82
11	At rt	11
12	Ag_2CO_3 (25 mol%)	76
13	Ag_2CO_3 (15 mol%)	48
14	Ag_2O instead of Ag_2CO_3	47
15	AgOAc instead of Ag_2CO_3	23
16	H_2O_2 (4.0 equiv.) instead of air	83
**17**	**Boc-Tyr-OMe (1.2 equiv.)**	**99**
18	Reaction time = 2h	49
19	Reaction time = 6h	81

[a] Reaction conditions: **1a** (0.50 mmol), phenothiazine (0.25 mmol), Ag_2CO_3 (30 mol %), *o*-xylene (1.0 mL) at 90 ºC for 16 h under air. [b] Yield of isolated product after column chromatography.

Table S2. Aqueous phenothiazination of Boc-Tyr-OMe[a]

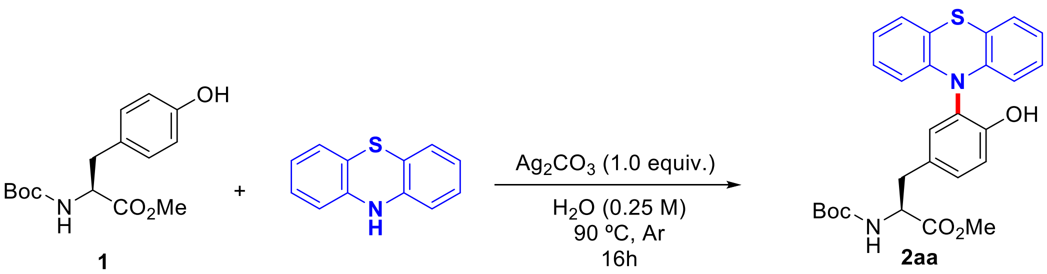

Entry	Variation from the standard conditions	2aa (%)[b]
1	none	83
2	under air	71
3	Ag_2O instead of Ag_2CO_3	61
4	AgOAc instead of Ag_2CO_3	46
5	$Cu(OAc)_2$ instead of Ag_2CO_3	38
6	$Co(OAc)_2$ instead of Ag_2CO_3	0
7	$Co(acac)_2$ instead of Ag_2CO_3	0
8	$Fe(OAc)_2$ instead of Ag_2CO_3	0
9	$FeCl_2$ instead of Ag_2CO_3	0
10	Ag_2CO_3 (2.0 equiv.) at 90 ºC	93
11	Ag_2CO_3 (0.5 equiv.) at 90 ºC	50
12	Ag_2CO_3 (1.0 equiv.) at 60 ºC	74
13	AgOAc (1.0 equiv.) at 90 ºC	35

[a] Reaction conditions: **1a** (0.50 mmol), phenothiazine (0.25 mmol), metal source (100 mol %), water (1.0 mL) at 90 ºC for 16 h under air. [b] Yield of isolated product after column chromatography.

3.-Synthesis of the Starting Materials

Tyr-containing peptides

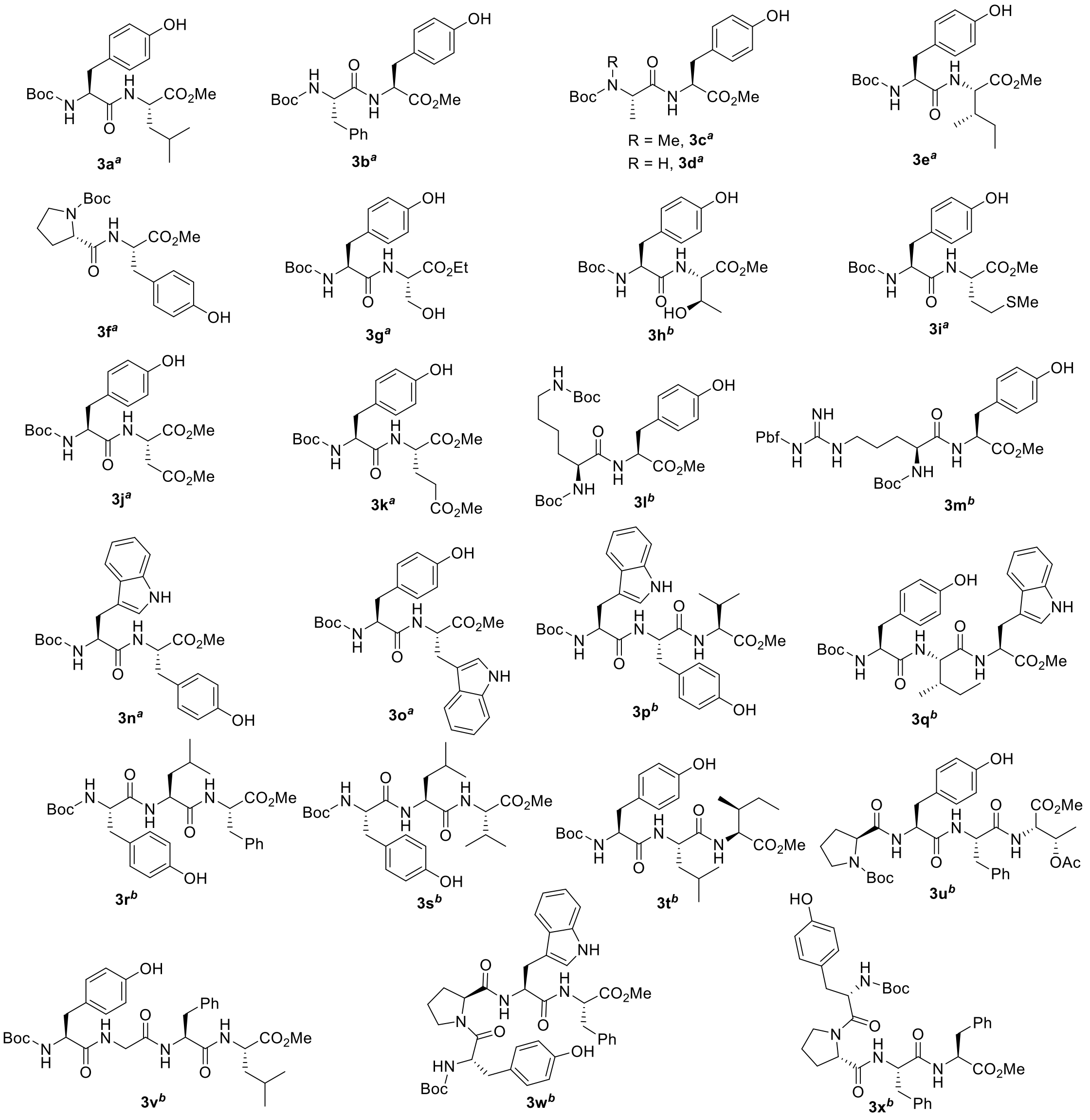

Tyr derivatives

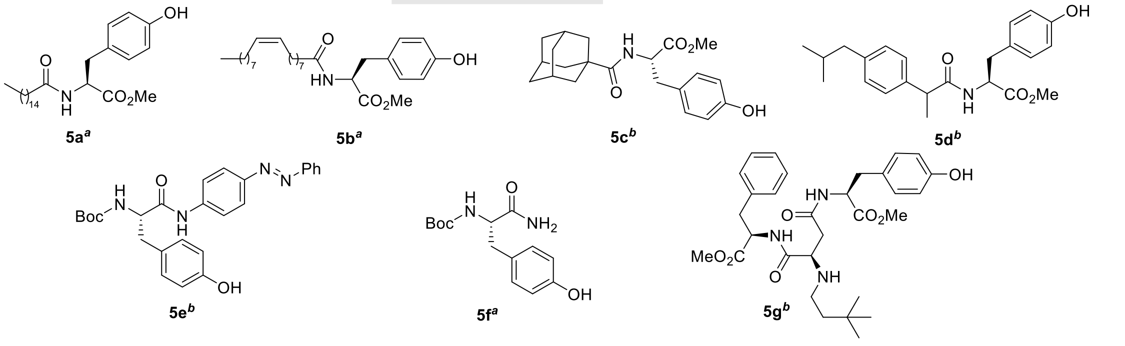

[a] Prepared following literature procedures. [b] Synthesis reported herein.

N-terminal Tyr-containing peptides

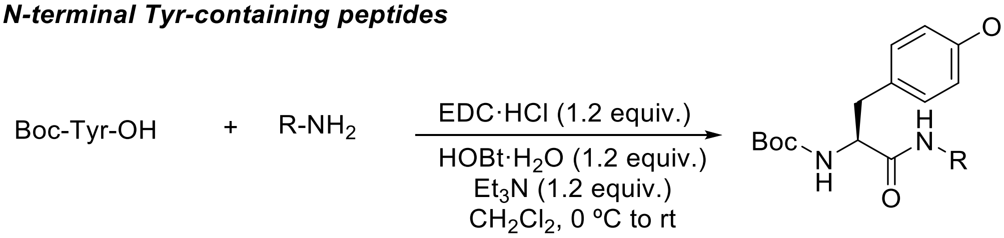

C-terminal Tyr-containing peptides

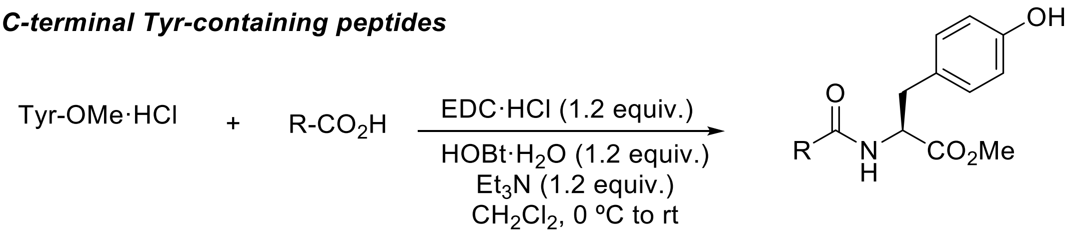

General Procedure: The corresponding carboxylic acid was dissolved in dichloromethane at 0 ºC. EDC·HCl (1.2 equiv.), HOBt·H_2O (1.2 equiv), the corresponding amine (1.2 equiv.) and triethylamine (1.2 equiv.) were subsequently added and stirred overnight at room temperature. The resulting solution was washed with water and extracted with dichloromethane. The combined organic phases were dried over $MgSO_4$ and evaporated under vacuum. The resulting residue was purified by flash chromatography to yield the corresponding Tyr-containing compound.

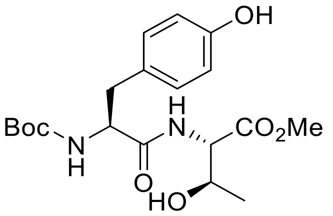

(3h)

Boc-Tyr-Thr-OMe (3h). Following the general procedure with *L*-Thr-OMe·HCl (17.69 mmol, 3.00 g) and Boc-Tyr-OH (14.73 mmol, 4.14 g) afforded **3h** (5.10 g, 87% yield) as a white solid. Mp 54-55 ºC. Column chromatography (Hex/EtOAc 3:7). ^{1}H NMR (400 MHz, $CDCl_3$) δ 7.08 – 6.90 (m, 2H), 6.81 – 6.59 (m, 2H), 5.32 (ddd, *J* = 15.8, 7.4, 3.2 Hz, 1H), 4.54 (dd, *J* = 8.9, 2.7 Hz, 1H), 4.49 – 4.21 (m, 2H), 3.67 (s, 3H), 3.17 – 2.72 (m, 2H), 1.39 (s, 9H), 1.14 (d, *J* = 6.3 Hz, 3H). ^{13}C NMR (101 MHz, $CDCl_3$) δ 172.5, 171.2, 155.9, 155.4, 130.5, 127.7, 115.7, 80.7, 68.3, 57.6, 56.2, 52.8, 37.5, 28.4, 19.9. HRMS (ESI-TOF) m/z: (M^+) *calcd.* for ($C_{19}H_{28}N_2O_7$): 396.1897, *found* 396.1902.

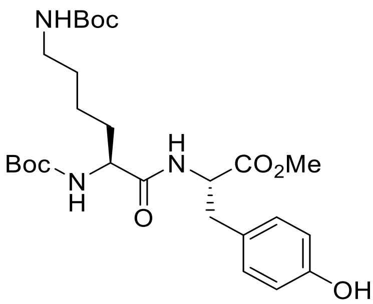

(3l)

Boc-Lys(Boc)-Tyr-OMe (3l). Following the general procedure with Boc-Lys(Boc)-OH (5.77 mmol, 2.00 g) and *L*-Tyr-OMe·HCl (6.35 mmol, 1.47 g) afforded **3l** (1.85 g, 61% yield) as a white solid. Mp 60-61 ºC. Column chromatography (Hex/EtOAc 3:7). ^{1}H NMR (400 MHz, $CDCl_3$) δ 6.93 (dd, J = 8.4, 3.2 Hz, 2H), 6.84 – 6.55 (m, 2H), 5.17 (s, 1H), 4.88 – 4.63 (m, 2H), 4.00 (s, 1H), 3.72 (s, 3H), 3.25 – 2.68 (m, 4H), 1.43 (s, 22H), 1.17 (s, 2H). ^{13}C NMR (101 MHz, $CDCl_3$) δ 172.1, 156.7, 155.8, 130.4, 127.0, 115.8, 80.4, 79.7, 54.8, 53.2, 52.5, 40.2, 37.2, 32.1, 29.8, 28.6, 28.4, 22.5. HRMS (ESI-TOF) m/z: (M^+) *calcd.* for ($C_{26}H_{41}N_3O_8$): 523.2894, *found* 523.2893.

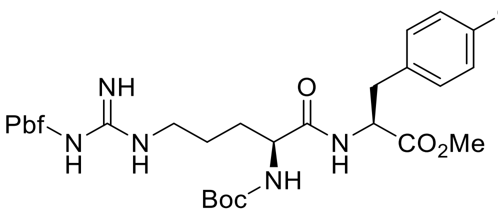

(3m)

Boc-Arg(Pbf)-Tyr-OMe (3m). Following the general procedure with Boc-Arg(Pbf)-OH (2.85 mmol, 1.50 g) and *L*-Tyr-OMe·HCl (3.41 mmol, 792 mg) afforded **3m** (1.75 g, 87% yield) as a white solid. Mp 119-120 ºC. Column chromatography (EtOAc). ^{1}H NMR (400 MHz, $CDCl_3$) δ 7.21 – 7.01 (m, 1H), 6.93 (d, J = 7.9 Hz, 2H), 6.74 (d, J = 7.9 Hz, 2H), 6.27 (s, 2H), 6.08 (s, 1H), 5.66 (s, 1H), 4.74 (t, J = 7.1 Hz, 1H), 4.04 – 3.84 (m, 1H), 3.69 (s, 3H), 3.27 – 2.99 (m, 3H), 2.93 (s, 3H), 2.56 (s, 3H), 2.49 (s, 3H), 2.07 (s, 3H), 1.65 – 1.45 (m, 4H), 1.44 (s, 6H), 1.38 (s, 9H). ^{13}C NMR (101 MHz, $CDCl_3$) δ 172.6, 172.2, 159.0, 156.4, 156.0, 155.7, 138.5, 132.4, 130.5, 127.2, 124.8, 117.7, 115.7, 86.6, 80.4, 54.7, 53.5, 52.6, 43.3, 40.6, 37.0, 29.5, 28.7, 28.4, 25.3, 19.4, 18.1, 12.6. HRMS (ESI-TOF) m/z: (M^+) *calcd.* for ($C_{34}H_{49}N_5O_9S$): 703.3251, *found* 703.3232.

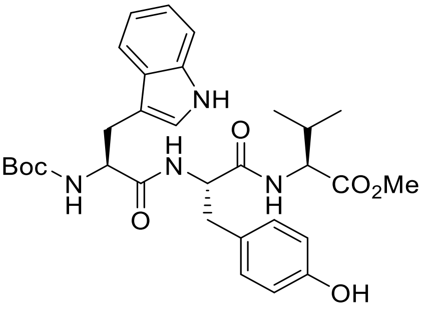

(3p)

Boc-Trp-Tyr-Val-OMe (3p). Following the general procedure with Boc-Trp-Tyr-OH (3.50 mmol, 1.63 g) and *L*-Val-OMe·HCl (4.20 mmol, 704 mg) afforded **3p** (1.10 g, 54% yield) as a white solid. Mp 84-85 ºC. Column chromatography (Hex/EtOAc 3:7). ^{1}H NMR (400 MHz, $CDCl_3$) δ 8.48 (s, 1H), 7.63 (d, *J* = 7.9 Hz, 1H), 7.36 (d, *J* = 8.1 Hz, 1H), 7.25 – 7.17 (m, 1H), 7.16 – 7.07 (m, 1H), 6.88 (s, 1H), 6.81 – 6.52 (m, 4H), 5.27 – 5.06 (m, 1H), 4.71 – 4.54 (m, 1H), 4.43 (ddd, *J* = 19.8, 10.4, 5.7 Hz, 2H), 3.70 (s, 3H), 3.30 – 3.29 (m, 1H), 3.14 (dd, *J* = 14.6, 6.4 Hz, 1H), 2.79 (dd, *J* = 42.0, 10.8 Hz, 2H), 2.10 – 2.09 (m, 1H), 1.38 (s, 9H), 0.87 (dd, *J* = 11.0, 6.8 Hz, 6H). ^{13}C NMR (101 MHz, $CDCl_3$) δ 172.2, 171.9, 171.0, 155.7, 155.4, 136.4, 130.5, 127.5, 123.4, 122.4, 119.8, 118.9, 115.7, 111.5, 110.0, 80.6, 57.8, 54.7, 52.3, 36.9, 31.2, 28.3, 19.0, 18.1. HRMS (ESI-TOF) m/z: (M^+) *calcd.* for ($C_{31}H_{40}N_4O_7$): 580.2897, *found* 580.2903.

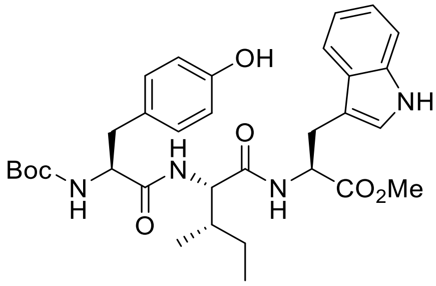

(3q)

Boc-Tyr-Ile-Trp-OMe (3q). Following the general procedure with Boc-Tyr-Ile-OH (3.67 mmol, 1.44 g) and *L*-Trp-OMe·HCl (4.40 mmol, 1.12 g) afforded **3q** (1.15 g, 53% yield) as a white solid. Mp 164-165 ºC. Column chromatography (Hex/EtOAc 1:1). ^{1}H NMR (400 MHz, $CDCl_3$) δ 8.78 – 8.25 (m, 1H), 7.48 (d, *J* = 7.7 Hz, 1H), 7.33 – 7.27 (m, 1H), 7.11 (dtd, *J* = 20.7, 7.1, 1.2 Hz, 2H), 6.99 – 6.84 (m, 3H), 6.66 (dd, *J* = 20.0, 7.9 Hz, 4H), 5.13 (d, *J* = 7.8 Hz, 1H), 4.86 (dt, *J* = 7.8, 5.7 Hz, 1H), 4.37 – 4.17 (m, 2H), 3.63 (s, 3H), 3.36 – 3.16 (m, 2H), 2.91 – 2.87 (m, 2H), 1.80 – 1.77 (m, 1H), 1.40 (s, 9H), 1.03 – 0.95 (m, 1H), 0.90 – 0.70 (m, 6H). ^{13}C NMR (101 MHz, $CDCl_3$) δ 172.1, 171.8, 170.8, 155.4, 155.7, 136.3, 130.4, 127.8, 127.4, 123.5, 122.2, 119.7, 118.5, 115.8, 111.6, 109.4, 80.7, 57.9, 56.2, 52.8, 52.6, 37.3, 28.4, 27.6, 24.8, 15.2, 11.3. HRMS (ESI-TOF) m/z: (M^+) *calcd.* for ($C_{32}H_{42}N_4O_7$): 594.3053, *found* 594.3050.

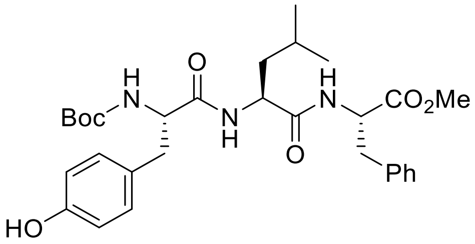

(3r)

Boc-Tyr-Leu-Phe-OMe (3r). Following the general procedure with Boc-Tyr-Leu-OH (7.60 mmol, 3.00 g) and *L*-Phe-OMe·HCl (9.13 mmol, 1.97 g) afforded **3r** (3.40 g, 81% yield) as a white solid. Mp 79-80 ºC. Column chromatography (Hex/EtOAc 4:6). ^{1}H NMR (400 MHz, $CDCl_3$) δ 7.39 – 7.20 (m, 3H), 7.20 – 7.09 (m, 2H), 6.99 (dd, *J* = 8.6, 2.7 Hz, 2H), 6.80 – 6.67 (m, 3H), 5.14 – 5.09 (m, 1H), 4.80 (dt, *J* = 7.8, 6.3 Hz, 1H), 4.44 – 4.38 (m, 1H), 4.28 (d, *J* = 8.0 Hz, 1H), 3.70 (s, 3H), 3.16 – 3.04 (m, 2H), 3.02 – 2.79 (m, 2H), 1.67 – 1.49 (m, 2H), 1.42 (s, 9H), 0.92 – 0.79 (m, 6H). ^{13}C NMR (101 MHz, $CDCl_3$) δ 171.8, 171.6, 155.7, 155.4, 135.9, 130.5, 129.4, 128.7, 127.8, 127.3, 115.8, 80.6, 56.0, 53.6, 52.5, 51.9, 41.0, 37.9, 37.3, 28.4, 24.6, 22.9, 22.1. HRMS (ESI-TOF) m/z: (M^+) *calcd.* for ($C_{30}H_{41}N_3O_7$): 555.2945, *found* 555.2924.

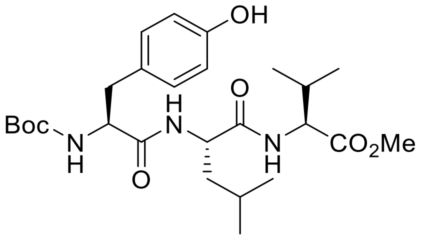

(3s)

Boc-Tyr-Leu-Val-OMe (3s). Following the general procedure with Boc-Tyr-Leu-OH (7.60 mmol, 3.00 g) and *L*-Val-OMe·HCl (9.13 mmol, 1.53 g) afforded **3s** (3.40 g, 88% yield) as a white solid. Mp 108-109 ºC. Column chromatography (Hex/EtOAc 4:6). ^{1}H NMR (400 MHz, $CDCl_3$) δ 7.20 – 6.83 (m, 2H), 6.83 – 6.59 (m, 2H), 5.43 – 5.02 (m, 1H), 4.49 (dd, *J* = 8.6, 5.1 Hz, 2H), 4.32 (t, *J* = 8.6 Hz, 1H), 3.71 (s, 3H), 2.96 – 2.82 (m, 2H), 2.21 – 2.04 (m, 1H), 1.62 – 1.54 (m, 4H), 1.36 (s, 9H), 0.91 – 0.85 (m, 12H). ^{13}C NMR (101 MHz, $CDCl_3$) δ 172.2, 155.5, 130.4, 127.7, 115.7, 80.4, 57.5, 55.9, 52.3, 52.1, 41.0, 37.4, 31.2, 28.4, 24.7, 22.8, 22.3, 19.1, 18.0. HRMS (ESI-TOF) m/z: (M^+) *calcd.* for ($C_{26}H_{41}N_3O_7$): 507.2945, *found* 507.2946.

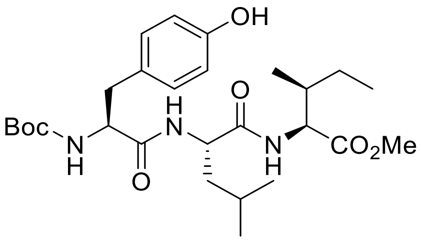

(3t)

Boc-Tyr-Leu-Ile-OMe (3t). Following the general procedure with Boc-Tyr-Leu-OH (7.60 mmol, 3.00 g) and *L*-Ile-OMe·HCl (9.13 mmol, 1.66 g) afforded **3t** (3.15 g, 79% yield) as a white solid. Mp 88-89 ºC. Column chromatography (Hex/EtOAc 4:6). ^{1}H NMR (400 MHz, $CDCl_3$) δ 7.07 – 6.86 (m, 3H), 6.85 – 6.53 (m, 3H), 5.18 (d, *J* = 7.9 Hz, 1H), 4.49 (ddd, *J* = 33.6, 8.4, 5.4 Hz, 2H), 4.37 – 4.23 (m, 1H), 3.71 (s, 3H), 2.98 – 2.86

(m, 2H), 1.90 – 1.86 (m, 1H), 1.63 – 1.45 (m, 5H), 1.38 (s, 9H), 1.30 – 1.04 (m, 2H), 0.97 – 0.73 (m, 12H). ^{13}C NMR (101 MHz, $CDCl_3$) δ 172.1, 172.06, 171.9, 155.7, 155.5, 130.4, 127.7, 115.8, 80.5, 56.8, 55.9, 52.2, 52.1, 41.0, 37.8, 37.4, 28.4, 25.2, 24.7, 22.9, 22.2, 15.6, 11.7. HRMS (ESI-TOF) m/z: (M^+) *calcd.* for ($C_{27}H_{43}N_3O_7$): 521.3101, *found* 521.3102.

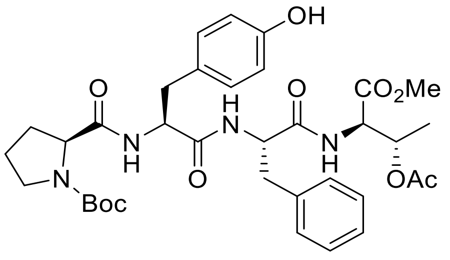

(3u)

Boc-Pro-Tyr-Phe-Thr(OAc)-OMe (3u). Following the general procedure with Boc-Pro-Tyr-OH (3.82 mmol) and H-Phe-Thr(OAc)-OMe (5.37 mmol) afforded **3u** (1.79 g, 69% yield) as a white solid. Mp 66-68 ºC. Column chromatography (Hex/EtOAc 3:7). ^{1}H NMR (500 MHz, DMSO-d_6 at 80 ºC) δ 8.86 (s, 1H), 8.04 (d, *J* = 8.6 Hz, 1H), 7.85 (d, *J* = 8.1 Hz, 1H), 7.40 (d, *J* = 8.0 Hz, 1H), 7.31 (dd, *J* = 8.2, 3.6 Hz, 4H), 7.27 – 7.18 (m, 1H), 7.06 – 6.94 (m, 2H), 6.67 (dd, *J* = 8.6, 2.2 Hz, 2H), 5.32 – 5.27 (m, 1H), 4.80 (td, *J* = 8.2, 5.3 Hz, 1H), 4.68 (ddd, *J* = 8.8, 4.1, 1.8 Hz, 1H), 4.52 (td, *J* = 8.1, 5.0 Hz, 1H), 4.18 – 4.01 (m, 1H), 3.70 (s, 3H), 3.34 – 3.32 (m, 1H), 3.24 – 3.09 (m, 2H), 2.95 (ddd, *J* = 14.3, 7.1, 5.4 Hz, 2H), 2.78 (dd, *J* = 14.2, 8.3 Hz, 1H), 2.03 (s, 3H), 1.85 – 1.58 (m, 4H), 1.36 (s, 9H), 1.25 (d, *J* = 6.4 Hz, 3H). ^{13}C NMR (126 MHz, DMSO-d_6 at 80 ºC) δ 171.3, 171.0, 170.5, 169.1, 168.8, 155.5, 137.0, 129.5, 129.47, 128.7, 127.5, 127.48, 127.2, 125.8, 125.7, 114.5, 78.4, 68.9, 59.4, 54.9, 53.5, 53.4, 53.2, 51.5, 46.1, 37.1, 36.4, 27.7, 27.6, 20.1, 16.1, 16.0. HRMS (ESI-TOF) m/z: (M^+) *calcd.* for ($C_{35}H_{46}N_4O_{10}$): 682.3214, *found* 682.3203.

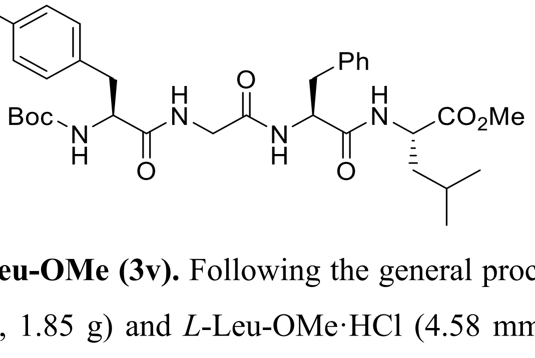

(**3v**)

Boc-Tyr-Gly-Phe-Leu-OMe (3v). Following the general procedure with Boc-Tyr-Gly-Phe-OH (3.82 mmol, 1.85 g) and *L*-Leu-OMe·HCl (4.58 mmol, 832 mg) afforded **3v** (1.50 g, 64% yield) as a white solid. Mp 84-85 ºC. Column chromatography (Hex/EtOAc 3:7). ^{1}H NMR (400 MHz, $CDCl_3$) δ 7.32 – 7.15 (m, 5H), 7.09 – 7.00 (m, 1H), 6.97 – 6.95 (m, 2H), 6.76 – 6.68 (m, 2H), 5.35 (br s, 1H), 4.75 – 4.69 (m, 1H), 4.55 (dq, *J* = 7.6, 5.4

Hz, 1H), 4.41 – 4.22 (m, 1H), 3.93 – 3.78 (m, 1H), 3.73 – 3.71 (m, 1H), 3.69 (s, 3H), 3.25 – 2.87 (m, 4H), 1.66 – 1.46 (m, 3H), 1.42 (s, 9H), 1.02 – 0.77 (m, 6H). ^{13}C NMR (101 MHz, $CDCl_3$) δ 173.4, 173.0, 171.7, 169.4, 156.2, 155.9, 136.8, 131.0, 130.7, 129.7, 128.9, 128.0, 127.4, 116.0, 80.7, 56.4, 55.1, 52.7, 51.4, 43.4, 41.4, 38.6, 38.4, 28.8, 25.1, 23.1, 22.2. HRMS (ESI-TOF) m/z: (M^+) *calcd.* for ($C_{32}H_{44}N_4O_8$): 612.3159, *found* 612.3182.

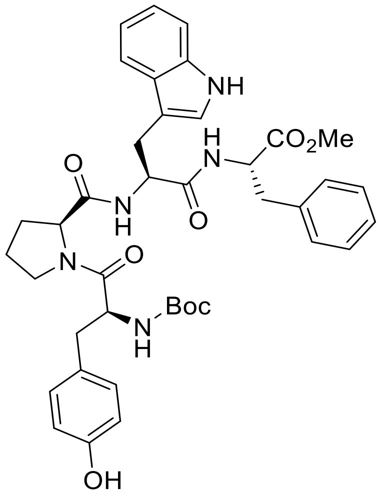

(3w)

Boc-Tyr-Pro-Trp-Phe-OMe (3w). Following the general procedure with Boc-Tyr-Pro-OH (6.89 mmol, 2.61 g) and H-Trp-Phe-OMe (8.28 mmol, 3.02 g) afforded **3w** (2.81 g, 56% yield) as a white solid. Mp 112-113 ºC. Column chromatography (Hex/EtOAc 2:8). ^{1}H NMR (500 MHz, DMSO-d_6 at 80 ºC) δ 10.65 (s, 1H), 7.99 (d, *J* = 7.4 Hz, 1H), 7.57 (dd, *J* = 24.8, 8.6 Hz, 1H), 7.36 – 7.15 (m, 5H), 7.14 – 6.93 (m, 4H), 6.66 (d, *J* = 8.3 Hz, 1H), 4.59 – 4.51 (m, 2H), 4.47 – 4.19 (m, 2H), 3.57 (s, 3H), 3.26 – 3.09 (m, 2H), 3.10 – 2.86 (m, 3H), 2.83 – 2.70 (m, 1H), 2.70 – 2.54 (m, 1H), 1.97 – 1.68 (m, 3H), 1.31 (s, 9H). ^{13}C NMR (126 MHz, DMSO-d_6 at 80 ºC) δ 171.2, 170.9, 170.7, 170.6, 155.6, 136.8, 135.9, 130.0, 129.9, 128.7, 127.9, 127.7, 127.3, 126.2, 123.2, 120.5, 117.94, 117.91, 114.7, 110.9, 109.6, 77.8, 59.4, 53.3, 53.0, 51.4, 46.4, 40.0, 39.9, 39.8, 39.7, 39.5, 39.3, 39.2, 39.0, 36.7, 27.9, 27.2, 24.1. HRMS (ESI-TOF) m/z: (M^+) *calcd.* for ($C_{40}H_{47}N_5O_8$): 725.3425, *found* 725.3426.

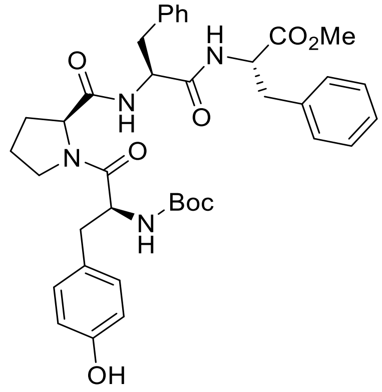

(3x)

Boc-Tyr-Pro-Phe-Phe-OMe (3x). Following the general procedure with Boc-Tyr-Pro-Phe-OH (3.10 mmol, 1.62 g) and *L*-Phe-OMe·HCl (3.70 mmol, 798 mg) afforded **3x** (1.21 g, 57% yield) as a white solid. Mp 75-77 ºC. Column chromatography (Hex/EtOAc 2:8). ^{1}H NMR (500 MHz, DMSO-d_6 at 80 ºC) δ 7.96 (d, J = 7.3 Hz, 1H), 7.50 (s, 1H), 7.32 – 7.14 (m, 10H), 7.05 (d, J = 8.0 Hz, 2H), 6.73 – 6.61 (m, 2H), 6.36 (s, 1H), 4.56 (q, J = 7.3 Hz, 2H), 4.40 – 4.33 (m, 2H), 3.59 (s, 3H), 3.20 – 2.55 (m, 8H), 1.92 – 1.76 (m, 4H), 1.33 (s, 9H). ^{13}C NMR (126 MHz, DMSO-d_6 at 80 ºC) δ 171.0, 170.5, 170.3, 155.5, 137.2, 136.7, 129.7, 128.7, 128.5, 127.8, 127.7, 127.5, 127.5, 126.0, 125.7, 114.7, 77.8, 59.4, 53.3, 53.2, 51.2, 46.3, 36.9, 36.8, 36.7, 36.0, 28.5, 28.0, 27.8, 23.9. HRMS (ESI-TOF) m/z: (M^{+}) *calcd.* for ($C_{38}H_{46}N_4O_8$): 686.3316, *found* 686.3317.

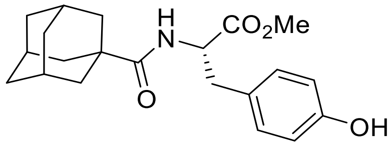

(5c)

Methyl adamantane-1-carbonyl-*L*-tyrosinate (5c). Following the general procedure with *L*-Tyr-OMe·HCl (6.47 mmol, 1.50 g) and 1-adamantane carboxylic acid (5.39 mmol, 971 mg) afforded **5c** (1.80 g, 94% yield) as a white solid. Mp 58-59 ºC. Column chromatography (Hex/EtOAc 1:1). ^{1}H NMR (400 MHz, $CDCl_3$) δ 7.00 – 6.85 (m, 2H), 6.85 – 6.67 (m, 2H), 6.14 (d, J = 7.8 Hz, 1H), 4.83 (dt, J = 7.9, 5.8 Hz, 1H), 3.73 (s, 3H), 3.07 (dd, J = 14.0, 5.7 Hz, 1H), 2.97 (dd, J = 14.0, 6.0 Hz, 1H), 2.00 – 1.99 (m, 3H), 1.80 – 1.57 (m, 12H). ^{13}C NMR (101 MHz, $CDCl_3$) δ 178.1, 172.6, 155.7, 130.4, 127.1, 115.7, 53.1, 52.5, 40.8, 39.1, 37.3, 36.5, 28.1. HRMS (ESI-TOF) m/z: (M^{+}) *calcd.* for ($C_{21}H_{27}NO_4$): 357.1940, *found* 357.1936.

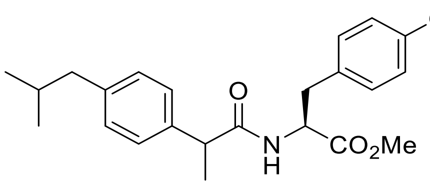

(5d)

Methyl [2-(4-isobutylphenyl)propanoyl]-*L*-tyrosinate (5d). Following the general procedure with *L*-Tyr-OMe·HCl (6.47 mmol, 1.50 g) and (*rac*)-ibuprofen (5.39 mmol, 1.11 g) afforded **5d** (1.97 g, 96% yield) as a white solid. The latter was obtained as a diastereomeric mixture (*dr* 1:1) and the following data correspond to the mixture of both isomers. Mp 58-59 ºC. Column chromatography (Hex/EtOAc 1:1). ^{1}H NMR (400 MHz, $CDCl_3$) δ 7.12 (d, *J* = 6.0 Hz, 4H), 6.81 – 6.65 (m, 2H), 6.62 (s, 2H), 6.06 – 5.90 (m, 1H), 4.81 (ddt, *J* = 26.9, 7.7, 5.7 Hz, 1H), 3.68 (d, *J* = 11.4 Hz, 3H), 3.62 – 3.43 (m, 1H), 3.09 – 2.77 (m, 2H), 2.46 (dd, *J* = 7.2, 5.4 Hz, 2H), 2.00 – 1.72 (m, 1H), 1.49 (dd, *J* = 16.7, 7.2 Hz, 3H), 0.90 (dd, *J* = 6.6, 3.4 Hz, 6H). ^{13}C NMR (101 MHz, $CDCl_3$) δ 175.0, 174.6, 172.3, 172.1, 155.7, 155.7, 141.0, 141.0, 137.9, 137.3, 130.3, 130.2, 130.0, 129.7, 127.4, 126.7, 126.5, 115.6, 115.6, 53.5, 53.2, 52.5, 52.4, 46.7, 46.6, 45.1, 37.0, 30.2, 22.4, 18.1. HRMS (ESI-TOF) m/z: (M^+) *calcd.* for ($C_{23}H_{29}NO_4$): 383.2097, *found* 383.2083.

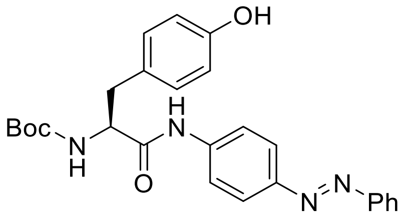

(5e)

***tert*-Butyl (*S*,*E*)-{3-(4-hydroxyphenyl)-1-oxo-1-[(4-(phenyldiazenyl)phenyl)amino] propan-2-yl}carbamate (5e).** To a solution of Boc-Tyr-OH (5.33 mmol, 1.50 g) in dichloromethane (20 mL) Et_3N (5.33 mmol, 0.73 mL) was added at -30 ºC. Then, $ClCO_2Et$ (5.33 mmol, 0.51 mL) was added under argon atmosphere and the resulting solution was stirred at -30 ºC for 30 min. Then, 4-aminoazobenzene (5.33 mmol, 1.05 g) was added and the resulting mixture was next warmed up to room temperature and stirred for 16 h. The solution was washed with an aqueous solution of HCl, and extracted with dichloromethane. The organic layers were combined and evaporated under vacuum. The resulting crude was then purified by column chromatography to afford **5e** (710 mg, 30% yield) as an orange solid. Mp 92-93 ºC. Column chromatography (Hex/EtOAc 1:1). ^{1}H NMR (400 MHz, $CDCl_3$) δ 8.47 (s, 1H), 7.92 – 7.75 (m, 4H), 7.60 – 7.37 (m, 5H), 7.07 (d, *J* = 8.0 Hz, 2H), 6.81 – 6.69 (m, 2H), 5.38 (s, 1H), 4.50 (s, 1H), 3.12 – 2.99 (m, 2H),

1.43 (s, 9H). ^{13}C NMR (101 MHz, $CDCl_3$) δ 170.4, 155.2, 152.7, 149.2, 140.0, 130.9, 130.6, 129.2, 128.2, 124.0, 122.9, 121.6, 120.1, 115.9, 81.2, 57.2, 37.7, 28.4 HRMS (ESI-TOF) m/z: (M^+) *calcd.* for ($C_{26}H_{28}N_4O_4$): 460.2111, *found* 460.2106.

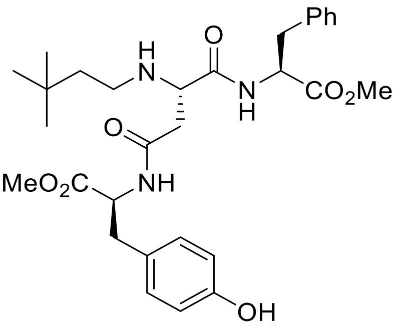

(5g)

Methyl [(*S*)-3-((3,3-dimethylbutyl)amino)-4-(((*S*)-1-methoxy-1-oxo-3-phenylpropan-2-yl)amino)-4-oxobutanoyl]-*L*-tyrosinate (5g). Following the general procedure with neotame (5.28 mmol, 2.00 g) and *L*-Tyr-OMe·HCl (6.34 mmol, 1.47 g) afforded **5g** (1.50 g, 51% yield) as a white solid. Mp 52-53 ºC. Column chromatography (Hex/EtOAc 2:8). ^{1}H NMR (400 MHz, $CDCl_3$) δ 8.18 (d, *J* = 8.5 Hz, 1H), 7.62 – 7.48 (m, 3H), 7.45 – 7.36 (m, 2H), 7.22 (d, *J* = 8.4 Hz, 2H), 6.98 (d, *J* = 8.4 Hz, 2H), 5.32 – 4.87 (m, 2H), 4.51 – 4.18 (m, 2H), 3.99 (s, 3H), 3.98 (s, 3H), 3.60 (dd, *J* = 8.3, 3.9 Hz, 1H), 3.44 – 3.22 (m, 4H), 2.89 – 2.68 (m, 3H), 2.60 (dd, *J* = 14.9, 8.4 Hz, 1H), 1.68 – 1.48 (m, 2H), 1.13 (s, 9H). ^{13}C NMR (101 MHz, $CDCl_3$) δ 173.7, 172.1, 171.7, 171.0, 155.8, 135.9, 130.4, 129.3, 128.7, 127.3, 127.0, 115.8, 60.0, 53.6, 53.1, 52.4, 44.7, 44.0, 38.0, 37.9, 37.0, 29.8, 29.6. HRMS (ESI-TOF) m/z: (M^+) *calcd.* for ($C_{30}H_{41}N_3O_7$): 555.2945, *found* 555.2940.

4.-C(sp^2)-H Amination of Tyr-Containing Compounds

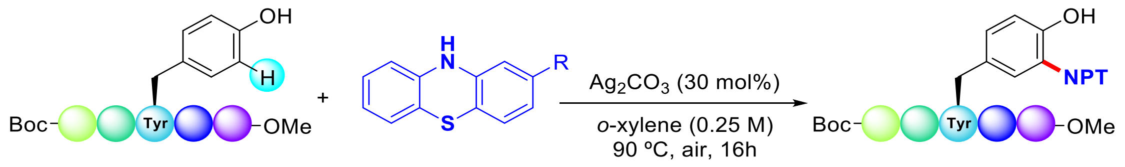

General Procedure A : A reaction tube containing a stirring bar was charged with the corresponding peptide (0.30 mmol), the phenothiazine derivative (0.25 mmol) and Ag_2CO_3 (30 mol%). Then, *o*-xylene (1 mL) was added under air. The reaction tube was next warmed up to 90 ºC in a heating block and stirred for 16 hours. The mixture was then allowed to warm to room temperature, evaporated under vacuum and the resulting crude was then purified by column chromatography to afford the corresponding product.

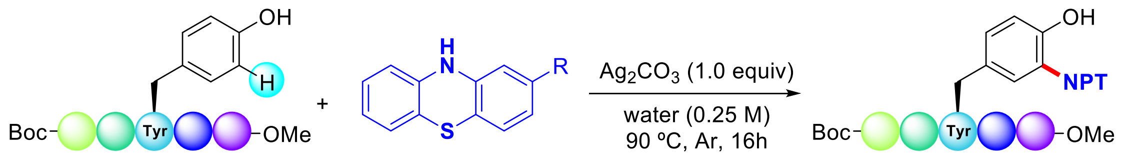

General Procedure B: A reaction tube containing a stirring bar was charged with the corresponding peptide (0.30 mmol), the phenothiazine derivative (0.25 mmol) and Ag_2CO_3 (0.25 mmol). The reaction tube was then evacuated and back-filled with dry argon (this sequence was repeated up to three times). Then, water (1 mL) was added by syringe under argon atmosphere. The reaction tube was next warmed up to 90º C in a heating block and stirred for 16 hours. The mixture was then allowed to warm to room temperature, the aqueous layer was extracted with EtOAc (3 x 20 mL), dried over $MgSO_4$ and evaporated under vacuum. The resulting crude was then purified by column chromatography to afford the corresponding product.

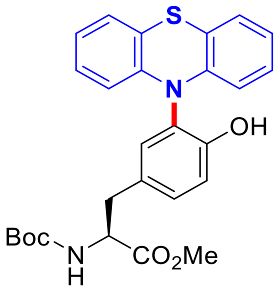

(2aa)

Methyl (*S*)-2-[(*tert*-butoxycarbonyl)amino]-3-[4-hydroxy-3-(10*H*-phenothiazin-10-yl)phenyl]propanoate (2aa). Following the general procedure A, using commercially available Boc-Tyr-OMe (0.30 mmol, 89 mg) and phenothiazine (0.25 mmol, 50 mg) provided 126 mg (99% yield) of **2aa** as a white solid.[1] Mp 73-74 ºC. Column chromatography (Hex/EtOAc 7:3). ^{1}H NMR (500 MHz, DMSO-d_6 at 80 ºC) δ 9.38 (s, 1H), 7.21 (dd, *J* = 8.3, 2.2 Hz, 1H), 7.12 – 7.03 (m, 2H), 6.95 (dd, *J* = 7.5, 1.6 Hz, 1H), 6.85 (td, *J* = 8.3, 7.9, 1.6 Hz, 2H), 6.77 (td, *J* = 7.4, 1.3 Hz, 2H), 6.72 (s, 1H), 6.12 (dd, *J* = 8.2, 1.3 Hz, 2H), 4.24 (td, *J* = 8.5, 5.7 Hz, 1H), 3.59 (s, 3H), 3.00 (dd, *J* = 14.0, 5.8 Hz, 1H), 2.89 (dd, *J* = 14.0, 9.0 Hz, 1H), 1.33 (s, 9H). ^{13}C NMR (126 MHz, DMSO-d_6 at 80 ºC) δ 171.7, 153.5, 142.5, 131.6, 130.1, 129.6, 126.5, 126.1, 125.5, 121.5, 118.1, 116.9, 115.0, 77.9, 55.0, 51.0, 35.5, 27.6. HRMS (ESI-TOF) m/z: (M^+) *calcd.* for ($C_{27}H_{28}N_2O_5S$): 492.1719, *found* 492.1732.

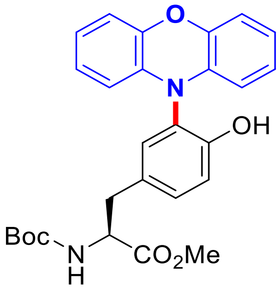

(2ab)

Methyl (*S*)-2-[(*tert*-butoxycarbonyl)amino]-3-[4-hydroxy-3-(10*H*-phenoxazin-10-yl)phenyl]propanoate (2ab). Following the general procedure A, using commercially available Boc-Tyr-OMe (0.30 mmol, 89 mg) and phenoxazine (0.25 mmol, 46 mg) provided 91 mg (76% yield) of **2ab** as a dark-pink solid. Mp 65-70 ºC. Column chromatography (Hex/EtOAc 7:3). ^{1}H NMR (500 MHz, DMSO-d_6 at 80 ºC) δ 9.33 (s, 1H), 7.19 (dd, *J* = 8.4, 2.2 Hz, 1H), 7.07 (d, *J* = 2.3 Hz, 1H), 7.03 (d, *J* = 8.4 Hz, 1H), 6.73 (s, 1H), 6.65 (dd, *J* = 22.4, 5.8 Hz, 6H), 5.90 (d, *J* = 6.3 Hz, 2H), 4.29 – 4.18 (m, 1H), 3.61 (s, 3H), 2.99 (dd, *J* = 14.1, 5.7 Hz, 1H), 2.88 (dd, *J* = 14.0, 9.0 Hz, 1H), 1.34 (s, 9H). ^{13}C NMR (126 MHz, DMSO-d_6 at 80 ºC) δ 171.8, 154.7, 153.4, 143.3, 133.4,

[1] Sun, J.; Liu, Z.; Jin, J. *Eur. J. Org. Chem.* **2023**, *26*, e202300081.

131.7, 130.3, 129.6, 123.7, 123.0, 120.6, 117.4, 114.6, 112.8, 78.1, 55.1, 51.2, 35.7, 27.8. HRMS (ESI-TOF) m/z: (M^+) *calcd.* for ($C_{27}H_{28}N_2O_6$): 476.1947, *found* 476.1960.

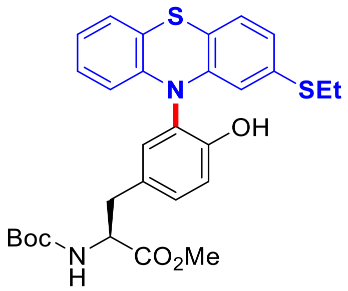

(2ac)

Methyl (*S*)-2-[(*tert*-butoxycarbonyl)amino]-3-{3-[2-(ethylthio)-10*H*-phenothiazin-10-yl]-4-hydroxyphenyl}propanoate (2ac). Following the general procedure A, using commercially available Boc-Tyr-OMe (0.30 mmol, 89 mg) and 2-(ethylthio)-10*H*-phenothiazine (0.25 mmol, 65 mg) provided 53 mg (38% yield) of **2ac** as a green solid. Mp 75-78 ºC. Column chromatography (Hex/EtOAc 7:3). ^{1}H NMR (500 MHz, DMSO-d_6 at 80 ºC) δ 9.52 (s, 1H), 7.23 (dd, J = 8.4, 2.3 Hz, 1H), 7.09 (d, J = 2.2 Hz, 1H), 7.07 (d, J = 8.3 Hz, 1H), 6.95 (dd, J = 7.5, 1.6 Hz, 1H), 6.90 (d, J = 7.9 Hz, 1H), 6.86 (td, J = 7.8, 1.6 Hz, 1H), 6.80 – 6.73 (m, 2H), 6.10 (dd, J = 8.2, 1.3 Hz, 1H), 6.05 (d, J = 1.8 Hz, 1H), 4.25 – 4.18 (m, 1H), 3.58 (s, 3H), 2.98 (dd, J = 14.1, 5.9 Hz, 1H), 2.89 (dd, J = 14.0, 8.8 Hz, 1H), 2.71 (q, J = 7.3 Hz, 2H), 1.32 (s, 9H), 1.12 (t, J = 7.3 Hz, 3H). ^{13}C NMR (126 MHz, DMSO-d_6 at 80 ºC) δ 171.8, 153.6, 142.8, 142.2, 134.6, 131.3, 130.3, 129.8, 126.6, 125.9, 125.8, 125.6, 121.8, 121.7, 118.1, 116.8, 115.7, 115.2, 115.2, 77.9, 55.0, 51.0, 35.4, 27.7, 26.4, 13.7. HRMS (ESI-TOF) m/z: (M^+) *calcd.* for ($C_{29}H_{32}N_2O_5S_2$): 552.1752, *found* 552.1725.

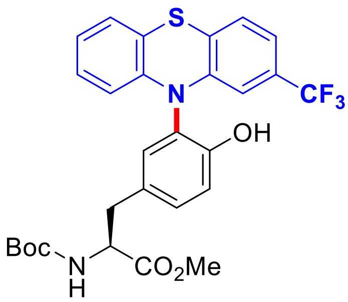

(2ad)

Methyl (*S*)-2-[(*tert*-butoxycarbonyl)amino]-3-{4-hydroxy-3-[2-(trifluoromethyl)-10*H*-phenothiazin-10-yl]phenyl}propanoate (2ad). Following the general procedure A, using commercially available Boc-Tyr-OMe (0.30 mmol, 89 mg) and 2-(trifluoromethyl)-10*H*-phenothiazine (0.25 mmol, 67 mg) provided 71 mg (51% yield) of **2ad** as a pink solid. Mp 75-78 ºC. Column chromatography (Hex/EtOAc 7:3). ^{1}H NMR (500 MHz, DMSO-d_6 at 80 ºC) δ 9.62 (s, 1H), 7.25 (dd, J = 8.4, 2.2 Hz, 1H), 7.16 (d, J =

8.0 Hz, 1H), 7.13 (d, J = 2.2 Hz, 1H), 7.09 (d, J = 8.3 Hz, 1H), 7.07 (dd, J = 8.0, 1.8 Hz, 1H), 6.98 (dd, J = 7.5, 1.6 Hz, 1H), 6.92 – 6.87 (m, 1H), 6.83 (td, J = 7.4, 1.3 Hz, 1H), 6.71 (s, 1H), 6.28 (d, J = 1.9 Hz, 1H), 6.10 (dd, J = 8.2, 1.3 Hz, 1H), 4.23 (td, J = 8.4, 5.9 Hz, 1H), 3.57 (s, 3H), 3.01-2.97 (m, 1H), 2.90 (dd, J = 14.0, 8.8 Hz, 1H), 1.31 (s, 9H). ^{13}C NMR (126 MHz, DMSO-d_6 at 80 ºC) δ 171.7, 153.4, 143.0, 141.8, 131.1, 130.7, 130.0, 127.6 (q, $J_{C\text{-}F}$ = 32.6 Hz), 127.0, 126.2, 125.7, 125.4, 123.8 (q, $J_{C\text{-}F}$ = 274.7 Hz), 123.8, 122.3, 117.9 (q, $J_{C\text{-}F}$ = 3.8 Hz), 117.0, 115.5, 110.6 (q, $J_{C\text{-}F}$ = 3.8 Hz), 77.9, 54.9, 50.9, 35.4, 27.6. ^{19}F NMR (376 MHz, $CDCl_3$) δ -62.78. HRMS (ESI-TOF) m/z: (M^+) *calcd*. for ($C_{28}H_{27}F_3N_2O_5S$):560.1593, *found* 560.1584.

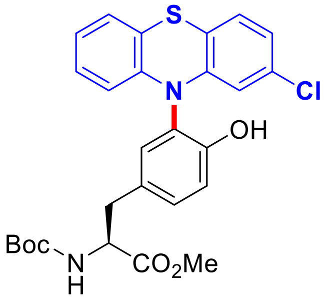

(2ae)

Methyl (*S*)-2-[(*tert*-butoxycarbonyl)amino]-3-[3-(2-chloro-10*H*-phenothiazin-10-yl)-4-hydroxyphenyl]propanoate (2ae). Following the general procedure A, using commercially available Boc-Tyr-OMe (0.30 mmol, 89 mg) and 2-chloro-10*H*-phenothiazine (0.25 mmol, 58 mg) provided 70 mg (53% yield) of **2ae** as a pink solid. Mp 77-80 ºC. Column chromatography (Hex/EtOAc 7:3). ^{1}H NMR (500 MHz, DMSO-d_6 at 80 ºC) δ 9.59 (s, 1H), 7.24 (dd, J = 8.2, 2.4 Hz, 1H), 7.17 – 7.05 (m, 2H), 6.97 (d, J = 8.5 Hz, 1H), 6.93 – 6.71 (m, 4H), 6.10 (d, J = 8.2 Hz, 1H), 6.04 (s, 1H), 4.31 – 4.17 (m, 1H), 3.58 (s, 3H), 3.00 – 2.97 (m, 1H), 2.89 (dd, J = 14.0, 9.0 Hz, 1H), 1.32 (s, 9H). ^{13}C NMR (126 MHz, DMSO-d_6 at 80 ºC) δ 171.8, 153.3, 143.8, 141.8, 131.3, 131.2, 130.6, 129.9, 126.8, 126.7, 125.6, 125.5, 122.2, 121.1, 117.8, 117.2, 117.0, 115.4, 114.5, 77.9, 54.9, 51.0, 35.4, 27.6. HRMS (ESI-TOF) m/z: (M^+) *calcd.* for ($C_{27}H_{27}ClN_2O_5S$): 526.1329, *found* 526.1338.

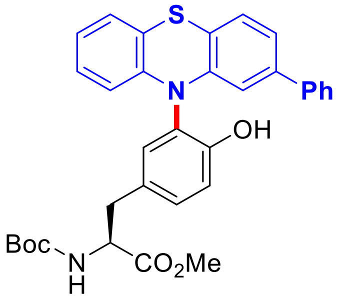

(2af)

Methyl (*S*)-2-[(*tert*-butoxycarbonyl)amino]-3-[4-hydroxy-3-(2-phenyl-10*H*-phenothiazin-10-yl)phenyl]propanoate (2af). Following the general procedure A, using commercially available Boc-Tyr-OMe (0.30 mmol, 89 mg) and 2-phenyl-10*H*-phenothiazine[2] (0.25 mmol, 69 mg) provided 80 mg (56% yield) of **2af** as a green solid. Mp 72-76 ºC. Column chromatography (Hex/EtOAc 1:1). ^{1}H NMR (500 MHz, DMSO-d_6 at 80 ºC) δ 9.51 (s, 1H), 7.37 – 7.30 (m, 4H), 7.29 – 7.25 (m, 1H), 7.23 (dd, *J* = 8.4, 2.3 Hz, 1H), 7.15 (d, *J* = 2.2 Hz, 1H), 7.08 (d, *J* = 8.3 Hz, 1H), 7.06 – 7.01 (m, 2H), 6.98 (dd, *J* = 7.5, 1.6 Hz, 1H), 6.87 (td, *J* = 7.8, 1.6 Hz, 1H), 6.80 (td, *J* = 7.4, 1.3 Hz, 1H), 6.74 (s, 1H), 6.36 (d, *J* = 1.7 Hz, 1H), 6.13 (dd, *J* = 8.2, 1.3 Hz, 1H), 4.28 – 4.16 (m, 1H), 3.54 (s, 3H), 2.99 (dd, *J* = 14.0, 5.9 Hz, 1H), 2.90 (dd, *J* = 14.0, 8.7 Hz, 1H), 1.28 (s, 9H). ^{13}C NMR (126 MHz, DMSO-d_6 at 80 ºC) δ 171.8, 153.7, 142.8, 142.4, 139.6, 139.0, 131.4, 130.3, 129.8, 128.3, 126.8, 126.6, 126.1, 126.0, 125.6, 125.6, 121.7, 120.0, 118.0, 117.6, 116.9, 115.3, 113.4, 77.9, 55.0, 51.0, 35.5, 27.6. HRMS (ESI-TOF) m/z: (M^+) *calcd.* for ($C_{33}H_{32}N_2O_5S$): 568.2032, *found* 568.2028.

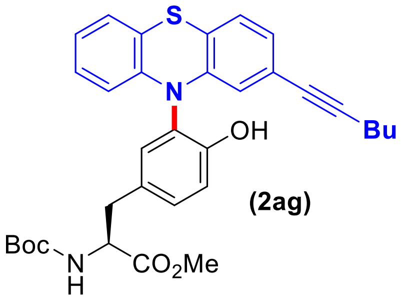

(2ag)

Methyl (*S*)-2-[(*tert*-butoxycarbonyl)amino]-3-[3-(2-(hex-1-yn-1-yl)-10*H*-phenothiazin-10-yl)-4-hydroxyphenyl]propanoate (2ag). Following the general procedure A, using Boc-Tyr-OMe (0.30 mmol, 89 mg) and 3-(hex-1-yn-1-yl)-10*H*-phenothiazine[3] (0.25 mmol, 70 mg) provided 66.6 mg (46% yield) of **2ag** as a brownish solid. Mp 77-78 ºC. Column chromatography (Hex/EtOAc 7:3). ^{1}H NMR (500 MHz, DMSO-d_6 at 80 ºC) δ 9.88 (s, 1H), 7.33 – 7.20 (m, 2H), 7.13 – 7.03 (m, 2H), 7.02 – 6.92 (m, 2H), 6.88 (td, *J* = 7.8, 1.6 Hz, 1H), 6.80 (ddd, *J* = 7.7, 5.8, 1.5 Hz, 2H), 6.13 – 5.88 (m, 2H), 4.28 – 4.05 (m, 1H), 3.54 (s, 3H), 2.93 (dd, *J* = 13.9, 5.8 Hz, 1H), 2.83 (dd, *J* = 13.8, 9.6 Hz, 1H), 2.29 (t, *J* = 7.1 Hz, 2H), 1.47 – 1.38 (m, 2H), 1.38 – 1.25 (m, 11H), 0.85 (t, *J* = 7.3 Hz, 3H). ^{13}C NMR (126 MHz, DMSO-d_6 at 80 ºC) δ 172.6, 155.2, 154.0, 142.7, 142.4, 131.6, 131.0, 130.2, 127.3, 126.2, 126.2, 125.7, 125.1, 122.4, 121.9, 118.8, 118.1, 117.4, 117.0,

---

[2] Onoabedje, E. A.; Okoro, U. C.; Knight, D. W.; Sarkar, A. *J. Heterocyclic Chem.* **2016**, *53*, 1787.
[3] Onoabedje, E. A.; Okoro, U. C.; Sarkar, A.; Knight, D. W. *J. Sulphur Chem.* **2016**, *37*, 269.

115.6, 90.6, 80.3, 78.1, 55.1, 51.6, 35.5, 30.1, 28.0, 27.8, 21.5, 21.4, 18.2, 13.4. HRMS (ESI-TOF) m/z: (M^+) *calcd.* for ($C_{33}H_{36}N_2O_5S$): 572.2345, *found* 572.2348.

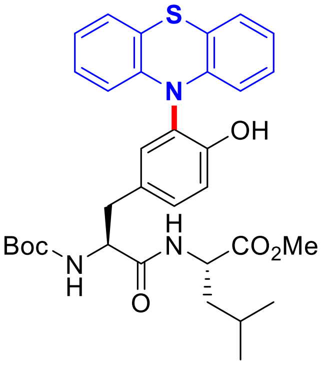

(4a)

Methyl [(*S*)-2-((*tert*-butoxycarbonyl)amino)-3-(4-hydroxy-3-(10*H*-phenothiazin-10-yl)phenyl)propanoyl]-*L*-leucinate (4a). Following the general procedure A, using Boc-Tyr-Leu-OMe[4] (0.30 mmol, 122.5 mg) and phenothiazine (0.25 mmol, 50 mg) provided 125.2 mg (83% yield) of **4a** as a pink solid. Mp 92-93 ºC. Column chromatography (Hex/EtOAc 6:4). ^{1}H NMR (500 MHz, DMSO-d_6 at 80 ºC) δ 9.33 (s, 1H), 7.89 (d, *J* = 8.0 Hz, 1H), 7.22 (dt, *J* = 8.2, 2.0 Hz, 1H), 7.17 (d, *J* = 2.1 Hz, 1H), 7.03 (dt, *J* = 8.2, 1.8 Hz, 1H), 6.94 (dt, *J* = 7.4, 1.6 Hz, 2H), 6.90 – 6.81 (m, 2H), 6.77 (t, *J* = 7.5 Hz, 2H), 6.32 (br s, 1H), 6.22 – 6.08 (m, 2H), 4.43 – 4.32 (m, 1H), 4.30 – 4.18 (m, 1H), 3.62 (s, 3H), 2.99 (ddd, *J* = 14.2, 4.4, 2.1 Hz, 1H), 2.87 – 2.62 (m, 1H), 1.78 – 1.51 (m, 3H), 1.31 (t, *J* = 1.7 Hz, 9H), 0.89 (ddt, *J* = 18.2, 6.6, 1.7 Hz, 6H). ^{13}C NMR (126 MHz, DMSO-d_6 at 80 ºC) δ 172.1, 170.9, 154.4, 153.3, 142.6, 131.6, 130.3, 130.1, 126.5, 126.0, 125.4, 121.4, 118.1, 116.6, 115.1, 77.7, 55.3, 51.1, 50.0, 36.3, 27.7, 27.6, 23.8, 22.1, 21.0. HRMS (ESI-TOF) m/z: (M^+) *calcd.* for ($C_{33}H_{39}N_3O_6S$): 605.2560, *found* 605.2546.

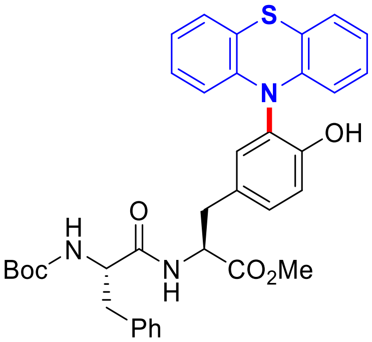

(4b)

Methyl (*S*)-2-[(*S*)-2-((*tert*-butoxycarbonyl)amino)-3-phenylpropanamido]-3-[4-hydroxy-3-(10*H*-phenothiazin-10-yl)phenyl]propanoate (4b). Following the general

[4] Percec, V.; Dulcey, A. E.; Peterca, M.; Adelman, P.; Samant, R.; Balagurusamy, V. S. K.; Heiney, P. A. *J. Am. Chem. Soc.* **2007**, *129*, 5992.

procedure A, using Boc-Phe-Tyr-OMe[5] (0.30 mmol, 132.7 mg) and phenothiazine (0.25 mmol, 50 mg) provided 152.1 mg (95% yield) of **4b** as a reddish solid. Mp 104-105 ºC. Column chromatography (Hex/EtOAc 6:4). ^{1}H NMR (500 MHz, DMSO-d_6 at 80 ºC) δ 9.42 (s, 1H), 7.93 (d, *J* = 7.7 Hz, 1H), 7.27 – 7.21 (m, 3H), 7.20 – 7.14 (m, 3H), 7.11 – 7.05 (m, 2H), 6.93 (dd, *J* = 7.6, 1.6 Hz, 2H), 6.83 (ddd, *J* = 8.4, 7.3, 1.6 Hz, 2H), 6.75 (t, *J* = 7.4 Hz, 2H), 6.31 (br s, 1H), 6.10 (dd, *J* = 8.2, 1.2 Hz, 2H), 4.59 (td, *J* = 7.8, 6.3 Hz, 1H), 4.22 (td, *J* = 9.0, 4.8 Hz, 1H), 3.56 (s, 3H), 3.05 (dd, *J* = 14.1, 6.3 Hz, 1H), 3.00 – 2.88 (m, 2H), 2.74 (dd, *J* = 14.0, 9.3 Hz, 1H), 1.32 (s, 9H). ^{13}C NMR (126 MHz, DMSO-d_6 at 80 ºC) δ 171.0, 170.9, 154.3, 153.6, 142.5, 137.3, 131.6, 130.1, 129.1, 128.6, 127.4, 126.5, 126.2, 125.6, 125.5, 121.5, 118.1, 117.1, 115.0, 77.9, 55.3, 53.2, 51.1, 37.4, 35.8, 27.6. HRMS (ESI-TOF) m/z: (M^+) *calcd.* for ($C_{36}H_{37}N_3O_6S$): 639.2403, *found* 639.2412.

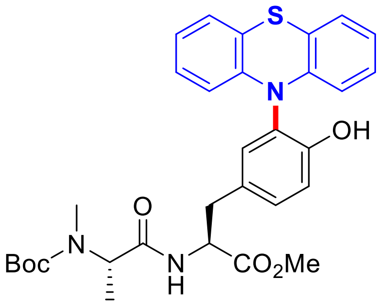

(4c)

Methyl (*S*)-2-[(*S*)-2-((*tert*-butoxycarbonyl)(methyl)amino)propanamido]-3-[4-hydroxy-3-(10*H*-phenothiazin-10-yl)phenyl]propanoate (4c). Following the general procedure A, using Boc-*N*-MeAla-Tyr-OMe[6] (0.30 mmol, 114 mg) and phenothiazine (0.25 mmol, 50 mg) provided 142 mg (99% yield) of **4c** as a purple solid. Mp 162-163 ºC. Column chromatography (Hex/EtOAc 6:4). ^{1}H NMR (500 MHz, DMSO-d_6 at 80 ºC) δ 9.43 (s, 1H), 7.63 (d, *J* = 8.0 Hz, 1H), 7.20 (dd, *J* = 8.3, 2.3 Hz, 1H), 7.14 – 6.99 (m, 2H), 6.99 – 6.65 (m, 6H), 6.10 (dd, *J* = 8.2, 1.3 Hz, 2H), 4.70 – 4.34 (m, 2H), 3.58 (s, 3H), 3.14 – 2.89 (m, 2H), 2.62 (s, 3H), 1.41 (s, 9H), 1.19 (d, *J* = 7.2 Hz, 3H). ^{13}C NMR (126 MHz, DMSO-d_6 at 80 ºC) δ 171.1, 170.7, 154.5, 153.6, 142.5, 131.5, 130.1, 129.4, 126.5, 126.3, 125.5, 121.5, 118.2, 117.0, 115.0, 78.6, 53.5, 53.2, 51.1, 35.3, 29.4, 27.6, 14.0. HRMS (ESI-TOF) m/z: (M^+) *calcd.* for ($C_{31}H_{35}N_3O_6S$): 577.2247, *found* 577.2265.

[5] Ge, J.; Zhang, C.-J.; Li, L.; Chong, L. M.; Wu, X.; Hao, P.; Sze, S. K.; Yao, S. K. *ACS Chem. Biol.* **2013**, *8*, 2577.
[6] Brady, S. F.; Freidinger, R. M.; Paleveda, W. J.; Colton, C. D.; Homnick, C. F.; Whitter, W. L.; Curley, P.; Nutt, R. F.; Veber, D. F. *J. Org. Chem.* **1987**, *52*, 764.

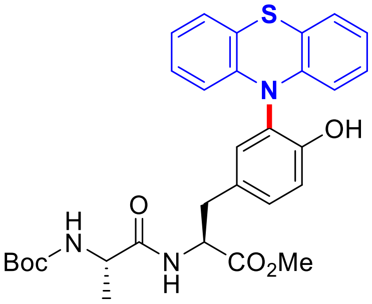

(4d)

Methyl (*S*)-2-[(*S*)-2-((*tert*-butoxycarbonyl)amino)propanamido)-3-(4-hydroxy-3-(10*H*-phenothiazin-10-yl)phenyl]propanoate (4d). Following the general procedure A, using Boc-Ala-Tyr-OMe[7] (0.30 mmol, 109.9 mg) and phenothiazine (0.25 mmol, 50 mg) provided 140 mg (99% yield) of **4d** as a brownish solid. Mp 95-96 ºC. Column chromatography (Hex/EtOAc 6:4). ^{1}H NMR (500 MHz, DMSO-d_6 at 80 ºC) δ 9.41 (s, 1H), 7.78 (d, *J* = 7.7 Hz, 1H), 7.21 (dd, *J* = 8.3, 2.3 Hz, 1H), 7.06 (d, *J* = 8.4 Hz, 2H), 6.94 (dd, *J* = 7.5, 1.7 Hz, 1H), 6.91 – 6.68 (m, 3H), 6.32 (br s, 1H), 6.09 (d, *J* = 8.2 Hz, 1H), 4.55 (td, *J* = 7.8, 6.1 Hz, 1H), 4.00 – 3.97 (m, 1H), 3.55 (s, 3H), 3.02 (dd, *J* = 14.0, 6.3 Hz, 1H), 2.94 (dd, *J* = 14.1, 8.0 Hz, 1H), 1.39 (s, 9H), 1.15 (d, *J* = 7.1 Hz, 3H). ^{13}C NMR (126 MHz, DMSO-d_6 at 80 ºC) δ 172.0, 171.1, 154.3, 131.9, 131.6, 130.7, 130.1, 129.1, 126.5, 125.4, 121.5, 117.0, 115.0, 77.8, 53.1, 51.1, 49.5, 35.7, 27.7, 17.7. HRMS (ESI-TOF) m/z: (M^+) *calcd.* for ($C_{30}H_{33}N_3O_6S$): 563.2090, *found* 563.2090.

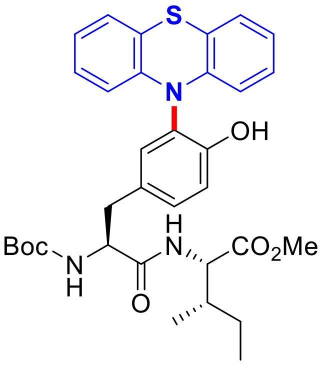

(4e)

Methyl [(*S*)-2-((*tert*-butoxycarbonyl)amino)-3-(4-hydroxy-3-(10*H*-phenothiazin-10-yl)phenyl)propanoyl]-*L*-isoleucinate (4e). Following the general procedure A, using Boc-Tyr-Ile-OMe[4] (0.30 mmol, 122.5 mg) and phenothiazine (0.25 mmol, 50 mg) provided 148.9 mg (98% yield) of **4e** as a brownish solid. Mp 88-89 ºC. Column chromatography (Hex/EtOAc 6:4). ^{1}H NMR (500 MHz, DMSO-d_6 at 80 ºC) δ 9.32 (s, 1H), 7.74 (d, *J* = 8.3 Hz, 1H), 7.22 (dd, *J* = 8.4, 2.2 Hz, 1H), 7.17 (d, *J* = 2.2 Hz, 1H), 7.04 (d, *J* = 8.2 Hz, 1H), 6.95 (dd, *J* = 7.5, 1.6 Hz, 2H), 6.85 (ddd, *J* = 8.4, 7.3, 1.6 Hz,

[7] Peters, D. S.; Romesberg, F. R.; Baran, P. S. *J. Am. Chem. Soc.* **2018**, *140*, 2072.

2H), 6.77 (t, J = 7.5 Hz, 2H), 6.42 (br s, 1H), 6.14 (dd, J = 8.2, 1.2 Hz, 2H), 4.41 – 4.19 (m, 2H), 3.63 (s, 3H), 2.99 (dd, J = 14.1, 4.6 Hz, 1H), 2.76 (dd, J = 14.1, 9.6 Hz, 1H), 1.82 (dtd, J = 8.3, 6.6, 4.8 Hz, 1H), 1.44 (ddd, J = 13.7, 7.3, 4.8 Hz, 1H), 1.31 (s, 9H), 1.23 – 1.16 (m, 1H), 0.98 – 0.78 (m, 6H). ^{13}C NMR (126 MHz, DMSO-d_6 at 80 ºC) δ 171.1, 171.0, 154.5, 153.3, 142.6, 131.6, 130.3, 130.1, 126.5, 126.0, 125.4, 121.4, 118.1, 116.6, 115.1, 77.8, 55.8, 55.3, 50.9, 36.3, 36.2, 27.6, 24.3, 14.8, 10.4. HRMS (ESI-TOF) m/z: (M^+) *calcd.* for ($C_{33}H_{39}N_3O_6S$): 605.2560, *found* 605.2554.

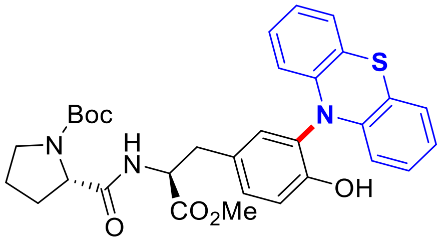

(4f)

***tert*-Butyl (*S*)-2-[((*S*)-3-(4-hydroxy-3-(10*H*-phenothiazin-10-yl)phenyl)-1-methoxy-1-oxopropan-2-yl)carbamoyl]pyrrolidine-1-carboxylate (4f).** Following the general procedure A, using Boc-Pro-Tyr-OMe[8] (0.30 mmol, 118 mg) and phenothiazine (0.25 mmol, 50 mg) provided 66 mg (45% yield) of **4f** as a brownish solid. Following the general procedure B, 104 mg (74%) of **4f** were obtained. Mp 98-100 ºC. Column chromatography (Hex/EtOAc 1:1). ^{1}H NMR (500 MHz, DMSO-d_6 at 80 ºC) δ 9.43 (s, 1H), 7.74 (d, J = 7.8 Hz, 1H), 7.22 (dd, J = 8.3, 2.3 Hz, 1H), 7.12 – 7.00 (m, 2H), 6.95 (dd, J = 7.5, 1.7 Hz, 1H), 6.86 (td, J = 7.8, 1.6 Hz, 2H), 6.78 (d, J = 7.5 Hz, 2H), 6.11 (dd, J = 8.2, 1.2 Hz, 2H), 4.56 (td, J = 8.0, 6.0 Hz, 1H), 4.11 (dd, J = 8.7, 3.6 Hz, 1H), 3.58 (s, 3H), 3.40 – 3.15 (m, 2H), 3.06 – 2.95 (m, 2H), 2.04 – 1.97 (m, 1H), 1.87 – 1.57 (m, 3H), 1.36 (s, 9H). ^{13}C NMR (126 MHz, DMSO-d_6 at 80 ºC) δ 171.7, 171.1, 153.7, 153.2, 142.5, 131.9, 131.5, 130.7, 130.0, 129.3, 126.5, 126.2, 125.5, 121.5, 121.2, 118.2, 117.0, 115.1, 78.2, 59.3, 53.1, 51.0, 46.1, 35.5, 27.6, 22.8. HRMS (ESI-TOF) m/z: (M^+) *calcd.* for ($C_{32}H_{35}N_3O_6S$): 589.2247, *found* 589.2241.

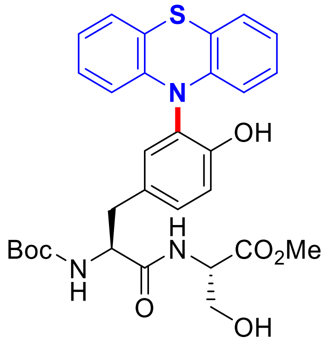

(4g)

[8] Laulloo, S. J.; Khodaboccus, A.; Hemraz, U.; Sunnassee, S. *Synth. Commun.* **2007**, *37*, 4191.

Methyl {(*S*)-2-[(*tert*-butoxycarbonyl)amino]-3-[4-hydroxy-3-(10*H*-phenothiazin-10-yl)phenyl]propanoyl}-*L*-serinate (4g). Following the general procedure A, using peptide Boc-Tyr-Ser-OMe[9] (0.30 mmol, 115 mg) and phenothiazine (0.25 mmol, 50 mg) provided 116 mg (80% yield) of **4g** as a dark-pink solid. Mp 110-114 ºC. Column chromatography (Hex/EtOAc 1:4). ^{1}H NMR (500 MHz, DMSO-d_6 at 80 ºC) δ 9.30 (s, 1H), 7.89 (d, J = 7.9 Hz, 1H), 7.25 – 7.22 (m, 1H), 7.18 (d, J = 2.2 Hz, 1H), 7.03 (d, J = 8.3 Hz, 1H), 6.94 (dd, J = 7.5, 1.6 Hz, 2H), 6.84 (td, J = 7.7, 1.6 Hz, 2H), 6.77 (t, J = 7.4 Hz, 2H), 6.36 (s, 1H), 6.16 – 6.12 (m, 2H), 4.39 (dt, J = 7.8, 4.9 Hz, 1H), 4.30 (td, J = 8.9, 4.3 Hz, 1H), 3.76 – 3.71 (m, 1H), 3.63 (s, 3H), 3.03 (dd, J = 14.0, 4.5 Hz, 2H), 2.76 (dd, J = 14.0, 9.5 Hz, 1H), 1.31 (s, 9H). ^{13}C NMR (126 MHz, DMSO-d_6 at 80 ºC) δ 171.0, 170.2, 153.2, 142.6, 131.8, 130.3, 130.1, 129.6, 126.5, 125.4, 121.4, 120.2, 118.1, 116.7, 115.1, 77.8, 61.0, 55.3, 54.2, 51.1, 36.4, 27.7. HRMS (ESI-TOF) m/z: (M^+) *calcd*. for ($C_{30}H_{33}N_3O_7S$): 579.2039, *found* 579.2033.

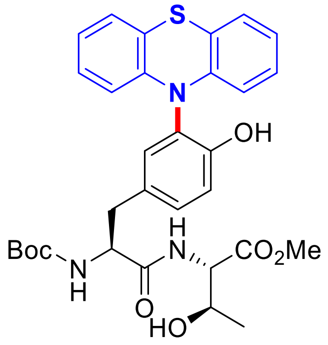

(4h)

Methyl [(*S*)-2-((*tert*-butoxycarbonyl)amino)-3-(4-hydroxy-3-(10*H*-phenothiazin-10-yl)phenyl)propanoyl]-*L*-threoninate (4h). Following the general procedure A, using **3h** (0.30 mmol, 119 mg) and phenothiazine (0.25 mmol, 50 mg) provided 65 mg (44% yield) of **4h** as a white solid. Following the general procedure B, 103 mg (70%) of **4h** were obtained. Mp 114-115 ºC. Column chromatography (Hex/EtOAc 4:6). ^{1}H NMR (500 MHz, DMSO-d_6 at 80 ºC) δ 9.31 (s, 1H), 7.70 (d, J = 8.5 Hz, 1H), 7.24 (dd, J = 8.3, 2.2 Hz, 1H), 7.19 (d, J = 2.2 Hz, 1H), 7.03 (d, J = 8.2 Hz, 1H), 6.95 (dd, J = 7.5, 1.6 Hz, 2H), 6.84 (td, J = 8.3, 7.9, 1.7 Hz, 2H), 6.77 (t, J = 7.4 Hz, 1H), 6.55 (br s, 1H), 6.13 (dd, J = 8.2, 1.3 Hz, 1H), 4.34 – 4.29 (m, 2H), 4.13 (dd, J = 6.4, 3.5 Hz, 1H), 3.62 (s, 3H), 3.04 (dd, J = 14.1, 4.4 Hz, 1H), 2.76 (dd, J = 14.0, 9.9 Hz, 1H), 1.30 (s, 9H), 1.08 (d, J = 6.4 Hz, 3H). ^{13}C NMR (126 MHz, DMSO-d_6 at 80 ºC) δ 171.5, 170.4, 154.6, 153.3, 142.6, 131.8, 130.4, 130.3, 126.6, 126.0, 125.5, 121.5, 118.1, 116.7, 115.2, 77.8, 65.9, 57.4,

[9] Mollica, A,; Costante, R.;Fiorito, S.; Genovese, S.; Stefanucci, A.; Mathieu, V.; Kiss, R.; Epifano, F. *Fitoterapia* **2014**, *98*, 91.

55.4, 51.2, 36.2, 27.8, 27.7, 19.6. HRMS (ESI-TOF) m/z: (M^+) *calcd.* for ($C_{31}H_{35}N_3O_7S$): 593.2196, *found* 593.2183.

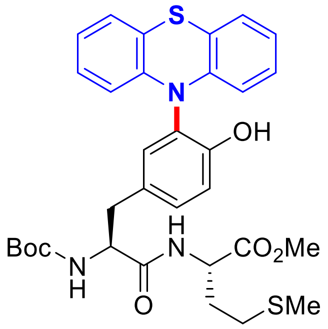

(4i)

Methyl [(*S*)-2-((*tert*-butoxycarbonyl)amino)-3-(4-hydroxy-3-(10*H*-phenothiazin-10-yl)phenyl)propanoyl]-*L*-methioninate (4i). Following the general procedure A, using Boc-Tyr-Met-OMe[10] (0.30 mmol, 128 mg) and phenothiazine (0.25 mmol, 50 mg) provided 89.4 mg (58% yield) of **4i** as a brownish solid. Mp 71-72 ºC. Column chromatography (Hex/EtOAc 6:4). ^{1}H NMR (500 MHz, DMSO-d_6 at 80 ºC) δ 9.32 (s, 1H), 7.98 (d, *J* = 7.9 Hz, 1H), 7.23 (dd, *J* = 8.3, 2.2 Hz, 1H), 7.17 (d, *J* = 2.1 Hz, 1H), 7.04 (d, *J* = 8.3 Hz, 1H), 6.95 (dd, *J* = 7.5, 1.6 Hz, 2H), 6.88 – 6.83 (m, 2H), 6.83 – 6.73 (m, 2H), 6.34 (br s, 1H), 6.14 (dd, *J* = 8.2, 1.2 Hz, 2H), 4.45 (td, *J* = 8.2, 5.1 Hz, 1H), 4.26 (td, *J* = 9.1, 4.6 Hz, 1H), 3.63 (s, 3H), 3.00 (dd, *J* = 14.1, 4.6 Hz, 2H), 2.77 (dd, *J* = 14.1, 9.4 Hz, 1H), 2.54 – 2.48 (m, 1H), 2.05 (s, 3H), 2.04 – 1.97 (m, 1H), 1.95 – 1.85 (m, 1H), 1.31 (s, 9H). ^{13}C NMR (126 MHz, DMSO-d_6 at 80 ºC) δ 171.3, 171.1, 154.4, 153.3, 142.6, 131.6, 130.3, 130.0, 126.5, 126.1, 125.4, 121.4, 118.1, 116.7, 115.1, 77.7, 55.3, 51.2, 50.6, 36.2, 30.7, 29.2, 27.6, 14.2. HRMS (ESI-TOF) m/z: (M^+) *calcd.* for ($C_{32}H_{37}N_3O_6S_2$): 623.2124, *found* 623.2137.

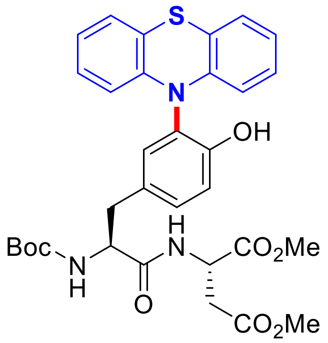

(4j)

Dimethyl [(*S*)-2-((*tert*-butoxycarbonyl)amino)-3-(4-hydroxy-3-(10*H*-phenothiazin-10-yl)phenyl)propanoyl]-*L*-aspartate (4j). Following the general procedure A, using Boc-Tyr-Asp(OMe)-OMe[11] (0.30 mmol, 127 mg) and phenothiazine (0.25 mmol, 50 mg)

[10] Koran, K. *J. Polym. Res.* **2022**, *29*, 532.
[11] Adhikari, B.; Banerjee, A. *Soft Matter* **2011**,*7*, 9259.

provided 75.9 mg (48% yield) of **4j** as a brownish solid. Following the general procedure B, 91 mg (58%) of **4j** were obtained. Mp 82-83 ºC. Column chromatography (Hex/EtOAc 1:1). ^{1}H NMR (500 MHz, DMSO-d_6 at 80 ºC) δ 9.39 (s, 1H), 8.13 (d, *J* = 7.9 Hz, 1H), 7.22 (dd, *J* = 8.3, 2.2 Hz, 1H), 7.15 (d, *J* = 2.2 Hz, 1H), 7.03 (d, *J* = 8.4 Hz, 1H), 6.95 (dd, *J* = 7.5, 1.6 Hz, 2H), 6.89 – 6.63 (m, 4H), 6.42 (br s, 1H), 6.12 (d, *J* = 8.2 Hz, 2H), 4.78 – 4.58 (m, 1H), 4.22 (td, *J* = 9.1, 4.4 Hz, 1H), 3.62 (s, 3H), 3.61 (s, 3H), 2.98 (dd, *J* = 14.1, 4.5 Hz, 1H), 2.83 – 2.67 (m, 3H), 1.30 (s, 9H). ^{13}C NMR (126 MHz, DMSO-d_6 at 80 ºC) δ 171.0, 170.4, 169.8, 154.5, 153.4, 142.6, 131.7, 130.4, 130.1, 126.6, 126.0, 125.5, 121.5, 118.1, 116.7, 115.1, 77.8, 55.3, 51.6, 51.1, 48.3, 36.3, 35.4, 27.7. HRMS (ESI-TOF) m/z: (M$^+$) *calcd.* for ($C_{32}H_{35}N_3O_8S$): 621.2145, *found* 621.2127.

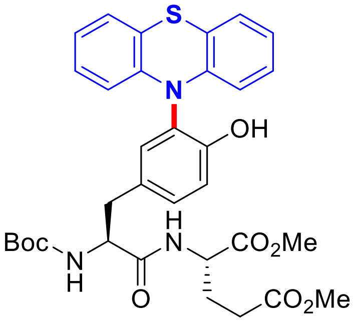

(4k)

Dimethyl {(*S*)-2-[(*tert*-butoxycarbonyl)amino]-3-[4-hydroxy-3-(10*H*-phenothiazin-10-yl)phenyl]propanoyl}-*L*-glutamate (4k). Following the general procedure A, using Boc-Tyr-Glu(OMe)-OMe[12] (0.30 mmol, 132 mg) and phenothiazine (0.25 mmol, 50 mg) provided 96 mg (60% yield) of **4k** as a pink solid. Mp 83-86 ºC. Column chromatography (Hex/EtOAc 1:1). ^{1}H NMR (500 MHz, DMSO-d_6 at 80 ºC) δ 9.32 (s, 1H), 8.01 – 7.90 (m, 1H), 7.22 (dd, *J* = 8.3, 2.2 Hz, 1H), 7.16 (d, *J* = 2.2 Hz, 1H), 7.03 (d, *J* = 8.2 Hz, 1H), 6.94 (dd, *J* = 7.5, 1.6 Hz, 2H), 6.84 (td, *J* = 7.7, 1.7 Hz, 2H), 6.77 (td, *J* = 7.4, 1.2 Hz, 2H), 6.34 (s, 1H), 6.13 (dd, *J* = 8.2, 1.3 Hz, 2H), 4.39 – 4.33 (m, 1H), 4.25 (td, *J* = 9.0, 4.5 Hz, 1H), 3.62 (s, 3H), 3.60 (s, 3H), 3.01-2.97 (m, 1H), 2.79 – 2.72 (m, 1H), 2.37 (t, *J* = 7.6 Hz, 2H), 2.05 (dtd, *J* = 13.4, 7.8, 5.5 Hz, 1H), 1.93 – 1.85 (m, 1H), 1.31 (s, 9H). ^{13}C NMR (126 MHz, DMSO-d_6 at 80 ºC) δ 171.9, 171.2, 171.1, 154.5, 153.3, 142.6, 131.7, 130.3, 130.1, 126.5, 126.1, 125.4, 121.5, 118.1, 116.7, 115.1, 77.8, 55.3, 51.2, 50.8, 50.7, 36.3, 29.2, 27.6, 26.0. HRMS (ESI-TOF) m/z: (M$^+$) *calcd*. for ($C_{33}H_{37}N_3O_8S$): 635.2301, *found* 635.2309.

[12] Duan, J.; Du, Y.-F.; Pang, X.; Shu, X.-Z. *Chem. Sci.* **2019**, *10*, 8706.

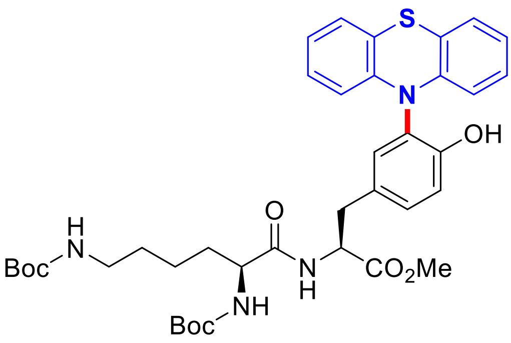

(4l)

Methyl (*S*)-2-[(*S*)-2,6-bis((*tert*-butoxycarbonyl)amino)hexanamido]-3-[4-hydroxy-3-(10*H*-phenothiazin-10-yl)phenyl]propanoate (4l). Following the general procedure A, using **1l** (0.30 mmol, 157 mg) and phenothiazine (0.25 mmol, 50 mg) provided 94.5 mg (53% yield) of **4l** as a brownish solid. Mp 95-96 ºC. Column chromatography (Hex/EtOAc 2:8). ^{1}H NMR (500 MHz, DMSO-d_6 at 80 ºC) δ 9.46 (s, 1H), 7.85 (d, *J* = 7.8 Hz, 1H), 7.20 (dd, *J* = 8.4, 2.3 Hz, 1H), 7.06 – 7.04 (m, 2H), 6.94 (dd, *J* = 7.5, 1.6 Hz, 2H), 6.85 (td, *J* = 7.8, 1.6 Hz, 2H), 6.77 (t, *J* = 7.4 Hz, 2H), 6.31 (d, *J* = 9.3 Hz, 2H), 6.12 – 6.02 (m, 2H), 4.54 (td, *J* = 7.8, 6.3 Hz, 1H), 3.91 – 3.89 (m, 1H), 3.54 (s, 3H), 3.06 – 2.79 (m, 4H), 1.58 – 1.54 (m, 4H), 1.44 – 1.12 (m, 20H). ^{13}C NMR (126 MHz, DMSO-d_6 at 80 ºC) δ 171.6, 171.2, 155.1, 154.6, 153.6, 142.5, 131.6, 130.2, 129.1, 126.8, 126.6, 126.1, 126.0, 125.5, 122.1, 121.6, 118.0, 117.0, 115.0, 77.8, 77.0, 54.1, 53.2, 51.1, 35.7, 31.4, 28.8, 27.9, 27.8, 22.1. HRMS (ESI-TOF) m/z: (M$^+$) *calcd.* for ($C_{38}H_{48}N_4O_8S$): 720.3193, *found* 720.3206.

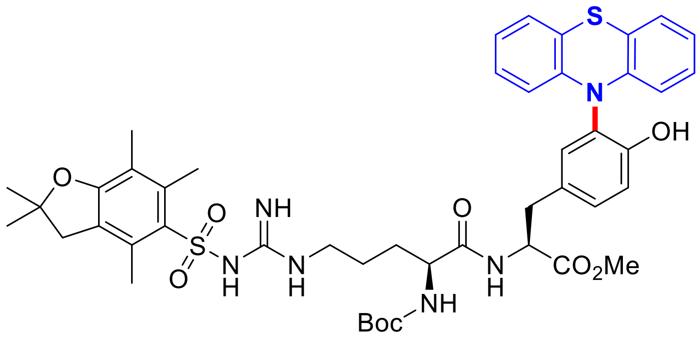

(4m)

Methyl (*S*)-2-[(*S*)-2-((*tert*-butoxycarbonyl)amino)-5-(3-((2,2,4,6,7-pentamethyl-2,3-dihydrobenzofuran-5-yl)sulfonyl)guanidino)pentanamido]-3-[4-hydroxy-3-(10*H*-phenothiazin-10-yl)phenyl]propanoate (4m). Following the general procedure A, using **3m** (0.30 mmol, 211 mg) and phenothiazine (0.25 mmol, 50 mg) provided 90 mg (40% yield) of **4m** as a white solid. Mp 155-156 ºC. Column chromatography (Hex/EtOAc 2:8). ^{1}H NMR (500 MHz, DMSO-d_6 at 80 ºC) δ 9.42 (s, 1H), 7.86 (d, *J* = 7.8 Hz, 1H), 7.21 – 7.19 (m, 1H), 7.12 – 7.01 (m, 2H), 6.93 (dd, *J* = 7.5, 2.6 Hz, 2H), 6.85 (dt, *J* = 8.1, 4.1 Hz, 2H), 6.76 (t, *J* = 7.9 Hz, 2H), 6.55 (s, 1H), 6.41 (s, 2H), 6.31 (br s, 1H), 6.08 (dd, *J* =

8.2, 3.0 Hz, 2H), 4.54 (dq, J = 7.5, 4.5 Hz, 1H), 3.93 (dd, J = 9.9, 5.7 Hz, 1H), 3.53 (s, 3H), 3.03 – 2.95 (m, 6H), 2.53 (s, 3H), 2.47 (s, 3H), 2.03 (s, 3H), 1.68 – 1.52 (m, 4H), 1.43 (s, 6H), 1.38 (s, 9H). ^{13}C NMR (126 MHz, DMSO-d_6 at 80 ºC) δ 171.3, 171.0, 157.1, 155.7, 154.6, 153.6, 142.4, 136.7, 134.2, 132.0, 131.6, 131.1, 130.7, 130.1, 129.1, 126.5, 126.2, 125.5, 123.8, 121.5, 121.2, 118.0, 117.1, 116.5, 115.7, 115.1, 115.0, 85.7, 77.9, 53.7, 53.2, 51.1, 42.3, 35.7, 29.1, 27.8, 27.7, 24.9, 18.2, 16.9, 11.6. HRMS (ESI-TOF) m/z: (M^+) *calcd.* for ($C_{46}H_{56}N_6O_9S_2$): 900.3550, *found* 900.3550.

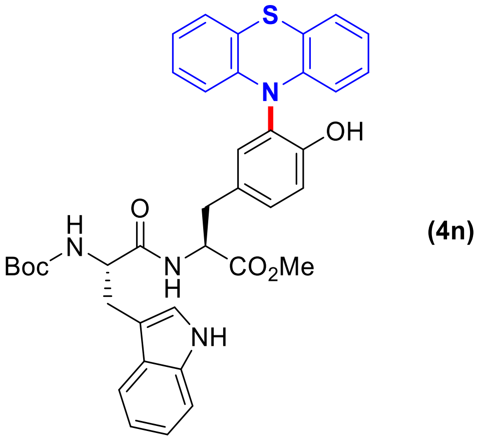

(4n)

Methyl (*S*)-2-[(*S*)-2-((*tert*-butoxycarbonyl)amino)-3-(1*H*-indol-3-yl)propanamido]-3-[4-hydroxy-3-(10*H*-phenothiazin-10-yl)phenyl]propanoate (4n). Following the general procedure A, using Boc-Trp-Tyr-OMe[13] (0.30 mmol, 144 mg) and phenothiazine (0.25 mmol, 50 mg) provided 83.7 mg (49% yield) of **4n** as a white solid. Mp 133-135 ºC. Column chromatography (Hex/EtOAc 6:4). ^{1}H NMR (500 MHz, DMSO-d_6 at 80 ºC) δ 10.57 (s, 1H), 9.45 (s, 1H), 7.93 (d, J = 7.7 Hz, 1H), 7.53 (d, J = 7.9 Hz, 1H), 7.33 (d, J = 8.1 Hz, 1H), 7.21 (dd, J = 8.3, 2.3 Hz, 1H), 7.11 – 7.00 (m, 4H), 7.00 – 6.90 (m, 3H), 6.90 – 6.81 (m, 2H), 6.75 (t, J = 7.6 Hz, 2H), 6.23 (br s, 1H), 6.09 (d, J = 8.2 Hz, 2H), 4.61–4.56 (m, 1H), 4.25 (td, J = 8.5, 4.9 Hz, 1H), 3.54 (s, 3H), 3.04 (ddd, J = 24.2, 14.4, 5.7 Hz, 2H), 2.91 (ddd, J = 19.7, 14.4, 8.2 Hz, 2H), 1.31 (s, 9H). ^{13}C NMR (126 MHz, DMSO-d_6 at 80 ºC) δ 171.3, 171.1, 154.4, 153.6, 142.5, 135.8, 131.7, 130.2, 129.1, 127.1, 126.6, 126.1, 125.5, 123.1, 121.5, 121.3, 120.3, 118.0, 117.9, 117.7, 117.0, 115.0, 110.8, 109.7, 77.8, 55.0, 53.2, 51.1, 35.9, 27.7, 27.6. HRMS (ESI-TOF) m/z: (M^+) *calcd.* for ($C_{38}H_{38}N_4O_6S$): 678.2512, *found* 678.2518.

[13] Laroche, B.; Tang, X.; Archer, G.; Di Sanza, R.; Melchiorre, P. *Org. Lett.* **2021**, *23*, 285.

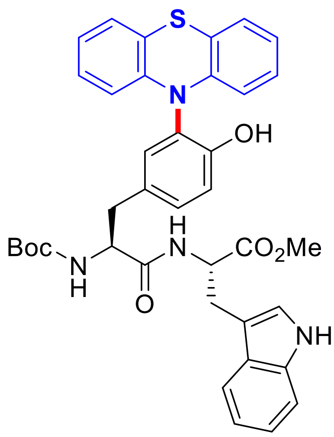

(4o)

Methyl {(*S*)-2-[(*tert*-butoxycarbonyl)amino]-3-[4-hydroxy-3-(10*H*-phenothiazin-10-yl)phenyl]propanoyl}-*L*-tryptophanate (4o). Following the general procedure A, using peptide Boc-Tyr-Trp-OMe[12] (0.30 mmol, 144 mg) and phenothiazine (0.25 mmol, 50 mg) provided 92 mg (54% yield) of **4o** as a dark-pink solid. Mp 107-110 ºC. Column chromatography (Hex/EtOAc 1:1). ^{1}H NMR (500 MHz, DMSO-d_6 at 80 ºC) δ 10.59 (s, 1H), 9.31 (s, 1H), 7.96 – 7.93 (m, 1H), 7.50 (d, *J* = 8.0 Hz, 1H), 7.34 (d, *J* = 8.1 Hz, 1H), 7.19 (dd, *J* = 8.3, 2.2 Hz, 1H), 7.14 (dd, *J* = 11.5, 2.3 Hz, 2H), 7.07 (t, *J* = 7.6 Hz, 1H), 7.02 (d, *J* = 8.2 Hz, 1H), 6.99 (t, *J* = 7.4 Hz, 1H), 6.94 (dd, *J* = 7.5, 1.6 Hz, 2H), 6.88 – 6.80 (m, 2H), 6.80 – 6.72 (m, 2H), 6.32 (s, 1H), 6.14 (d, *J* = 8.2 Hz, 2H), 4.63 (q, *J* = 7.0 Hz, 1H), 4.26 (td, *J* = 9.1, 4.4 Hz, 1H), 3.56 (s, 3H), 3.25 – 3.16 (m, 1H), 3.12 (dd, *J* = 14.7, 7.2 Hz, 1H), 2.98 (dd, *J* = 14.0, 4.5 Hz, 1H), 2.73 (dd, *J* = 14.0, 9.6 Hz, 1H), 1.30 (s, 9H). ^{13}C NMR (126 MHz, DMSO-d_6 at 80 ºC) δ 171.4, 170.9, 154.4, 153.3, 142.6, 135.9, 131.7, 130.3, 130.1, 126.9, 126.5, 126.1, 125.4, 123.1, 121.5, 120.5, 118.1, 118.0, 117.5, 116.7, 115.2, 110.9, 108.9, 77.8, 55.4, 52.6, 51.1, 36.4, 27.7, 27.0. HRMS (ESI-TOF) m/z: (M^+) *calcd*. for ($C_{38}H_{38}N_4O_6S$): 678.2512, *found* 678.2521.

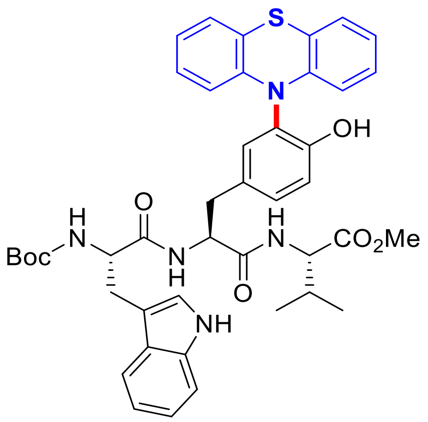

(4p)

Methyl [(*S*)-2-((*S*)-2-((*tert*-butoxycarbonyl)amino)-3-(1*H*-indol-3-yl)propanamido)-3-(4-hydroxy-3-(10*H*-phenothiazin-10-yl)phenyl)propanoyl]-*L*-valinate (4p). Following the general procedure A, using **3p** (0.30 mmol, 174 mg) and phenothiazine

(0.25 mmol, 50 mg) provided 90.8 mg (46% yield) of **4p** as a white solid. Mp 162-163 ºC. Column chromatography (Hex/EtOAc 6:4). ^{1}H NMR (500 MHz, DMSO-*d*$_6$ at 80 ºC) δ 10.56 (s, 1H), 9.36 (s, 1H), 7.99 (d, *J* = 8.3 Hz, 1H), 7.67 (d, *J* = 8.2 Hz, 1H), 7.52 (d, *J* = 7.9 Hz, 1H), 7.32 (d, *J* = 8.1 Hz, 1H), 7.22 (dd, *J* = 8.3, 2.3 Hz, 1H), 7.18 (d, *J* = 2.2 Hz, 1H), 7.10 – 7.00 (m, 3H), 7.00 – 6.95 (m, 1H), 6.92 (dd, *J* = 7.5, 1.6 Hz, 2H), 6.82 (td, *J* = 7.8, 1.6 Hz, 2H), 6.79 – 6.70 (m, 2H), 6.35 (br s, 1H), 6.12 (dd, *J* = 8.2, 1.2 Hz, 2H), 4.75 (td, *J* = 8.4, 5.0 Hz, 1H), 4.19 (ddd, *J* = 19.6, 8.5, 5.2 Hz, 2H), 3.58 (s, 3H), 3.22 – 2.98 (m, 2H), 2.83 (ddd, *J* = 19.4, 14.5, 9.0 Hz, 2H), 2.08 – 2.01 (m, 1H), 1.29 (s, 9H), 0.89 (t, *J* = 7.0 Hz, 6H). ^{13}C NMR (126 MHz, DMSO-*d*$_6$ at 80 ºC) δ 171.1, 171.07, 170.7, 154.5, 153.3, 142.6, 135.9, 131.9, 130.6, 129.6, 127.1, 126.6, 126.0, 125.5, 123.0, 121.5, 120.4, 120.36, 117.9, 117.9, 117.8, 116.7, 115.2, 110.8, 110.0, 77.9, 57.1, 55.4, 53.1, 51.0, 36.7, 29.6, 27.7, 18.4, 17.7. HRMS (ESI-TOF) m/z: (M$^+$) *calcd.* for ($C_{43}H_{47}N_5O_7S$): 777.3196, *found* 777.3197.

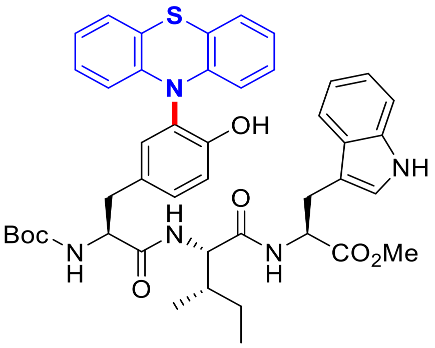

(**4q**)

Methyl [(*S*)-2-((*tert*-butoxycarbonyl)amino)-3-(4-hydroxy-3-(10*H*-phenothiazin-10-yl)phenyl)propanoyl]-*L*-isoleucyl-*L*-tryptophanate (4q). Following the general procedure A, using **1q** (0.30 mmol, 178 mg) and phenothiazine (0.25 mmol, 50 mg) provided 112 mg (56% yield) of **4q** as a white solid. Mp 168-169 ºC. Column chromatography (Hex/EtOAc 3:7). ^{1}H NMR (500 MHz, DMSO-*d*$_6$ at 80 ºC) δ 10.58 (s, 1H), 9.32 (s, 1H), 8.00 (d, *J* = 7.4 Hz, 1H), 7.49 (dd, *J* = 8.3, 4.9 Hz, 2H), 7.33 (d, *J* = 8.1 Hz, 1H), 7.23 (dd, *J* = 8.3, 2.2 Hz, 1H), 7.18 (d, *J* = 2.2 Hz, 1H), 7.13 (d, *J* = 2.4 Hz, 1H), 7.09 – 7.01 (m, 2H), 6.97 (ddd, *J* = 19.2, 7.7, 1.4 Hz, 3H), 6.85 (td, *J* = 7.8, 1.6 Hz, 2H), 6.78 (t, *J* = 7.4 Hz, 2H), 6.53 (br s, 1H), 6.15 (dd, *J* = 8.2, 1.2 Hz, 2H), 4.61 (q, *J* = 7.0 Hz, 1H), 4.30 (dd, *J* = 8.8, 6.9 Hz, 1H), 4.24 (ddd, *J* = 10.2, 8.6, 4.1 Hz, 1H), 3.56 (s, 3H), 3.20 (dd, *J* = 14.7, 6.4 Hz, 1H), 3.12 (dd, *J* = 14.7, 7.3 Hz, 1H), 2.97 (dd, *J* = 14.2, 4.2 Hz, 1H), 2.72 (dd, J = 14.2, 10.3 Hz, 1H), 1.79 –1.74 (m, 1H), 1.50 – 1.42 (m, 1H), 1.29 (s, 9H), 1.11 (ddd, *J* = 13.7, 9.0, 7.2 Hz, 1H), 0.93 – 0.71 (m, 6H). ^{13}C NMR (126 MHz, DMSO-*d*$_6$ at 80 ºC) δ 171.3, 170.8, 170.3, 154.6, 153.2, 142.6, 135.8, 131.6, 130.4, 130.2,

126.8, 126.5, 126.0, 125.4, 123.1, 121.4, 120.4, 118.1, 117.9, 117.4, 116.6, 115.1, 110.9, 109.0, 77.7, 56.3, 55.6, 52.6, 51.0, 36.7, 36.0, 27.6, 26.7, 23.7, 14.7, 10.4. HRMS (ESI-TOF) m/z: (M^+) *calcd.* for ($C_{44}H_{49}N_5O_7S$): 791.3353, *found* 791.3361.

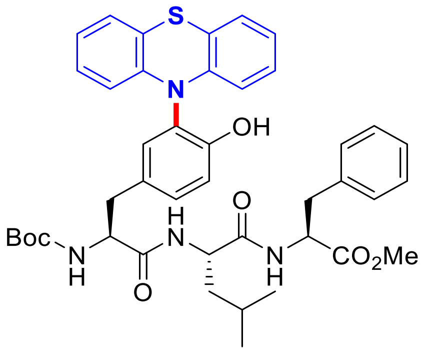

(4r)

Methyl [(*S*)-2-((*tert*-butoxycarbonyl)amino)-3-(4-hydroxy-3-(10*H*-phenothiazin-10-yl)phenyl)propanoyl]-*L*-leucyl-*L*-phenylalaninate (4r). Following the general procedure A, using **3r** (0.30 mmol, 167 mg) and phenothiazine (0.25 mmol, 50 mg) provided 145 mg (77% yield) of **4r** as a white solid. Mp 134-135 ºC. Column chromatography (Hex/EtOAc 1:1). ^{1}H NMR (500 MHz, DMSO-d_6 at 80 ºC) δ 9.31 (s, 1H), 7.90 (d, *J* = 7.6 Hz, 1H), 7.62 (d, *J* = 8.4 Hz, 1H), 7.29 – 7.14 (m, 6H), 7.03 (d, *J* = 8.3 Hz, 1H), 6.95 (dd, *J* = 7.5, 1.7 Hz, 1H), 6.85 (td, *J* = 8.3, 7.8, 1.7 Hz, 2H), 6.77 (td, *J* = 7.4, 1.3 Hz, 2H), 6.43 (br s, 1H), 6.14 (dd, *J* = 8.2, 1.3 Hz, 2H), 4.55 (td, *J* = 7.9, 6.1 Hz, 1H), 4.36 (td, *J* = 8.7, 5.6 Hz, 1H), 4.28 – 4.09 (m, 1H), 3.58 (s, 3H), 3.13 – 2.86 (m, 3H), 2.71 (dd, *J* = 14.1, 10.0 Hz, 1H), 1.72 – 1.58 (m, 1H), 1.58 – 1.38 (m, 2H), 1.29 (s, 9H), 0.87 (dd, *J* = 15.6, 6.5 Hz, 6H). ^{13}C NMR (126 MHz, DMSO-d_6 at 80 ºC) δ 171.2, 170.9, 170.7, 154.5, 153.2, 142.6, 136.5, 131.6, 130.3, 130.3, 128.4, 127.6, 127.6, 126.5, 126.1, 125.9, 125.4, 121.4, 118.1, 116.6, 115.1, 77.7, 55.5, 52.9, 51.1, 50.7, 40.7, 36.4, 36.2, 27.6, 23.6, 22.3, 21.4. HRMS (ESI-TOF) m/z: (M^+) *calcd.* for ($C_{42}H_{48}N_4O_7S$): 752.3244, *found* 752.3276.

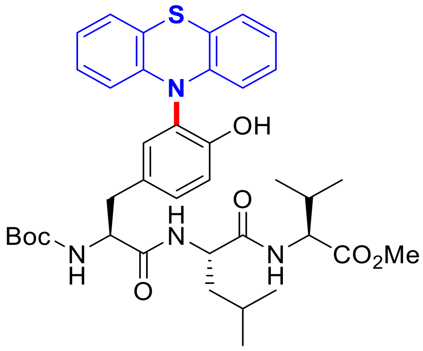

(4s)

Methyl [(*S*)-2-((*tert*-butoxycarbonyl)amino)-3-(4-hydroxy-3-(10*H*-phenothiazin-10-yl)phenyl)propanoyl]-*L*-leucyl-*L*-valinate (4s). Following the general procedure A,

using **3s** (0.30 mmol, 152.3 mg) and phenothiazine (0.25 mmol, 50 mg) provided 152.5 mg (87% yield) of **4s** as a grey solid. Mp 120-122 ºC. Column chromatography (Hex/EtOAc 6:4). ^{1}H NMR (500 MHz, DMSO-d_6 at 80 ºC) δ 9.32 (s, 1H), 7.71 (d, *J* = 8.2 Hz, 2H), 7.23 (d, *J* = 1.9 Hz, 1H), 7.22 – 7.14 (m, 1H), 7.03 (d, *J* = 8.2 Hz, 1H), 6.95 (d, *J* = 7.3 Hz, 2H), 6.85 (t, *J* = 7.7 Hz, 2H), 6.77 (t, *J* = 7.4 Hz, 2H), 6.46 (br s, 1H), 6.14 (d, *J* = 8.1 Hz, 2H), 4.44 (td, *J* = 8.5, 5.5 Hz, 1H), 4.36 – 4.06 (m, 2H), 3.64 (s, 3H), 3.00 (dd, *J* = 14.1, 4.2 Hz, 1H), 2.75 (dd, *J* = 14.0, 9.9 Hz, 1H), 2.13 – 1.98 (m, 1H), 1.68 (dt, *J* = 13.5, 6.7 Hz, 1H), 1.59 – 1.45 (m, 2H), 1.39 – 1.22 (m, 9H), 0.99 – 0.76 (m, 12H). ^{13}C NMR (126 MHz, DMSO-d_6 at 80 ºC) δ 171.5, 171.0, 170.8, 154.5, 153.3, 142.6, 131.6, 130.3, 126.5, 126.1, 125.4, 121.4, 118.2, 116.7, 115.1, 77.7, 57.0, 55.6, 50.9, 50.8, 40.6, 36.2, 29.5, 27.6, 23.7, 22.4, 21.4, 18.3, 17.7. HRMS (ESI-TOF) m/z: (M^+) *calcd.* for ($C_{38}H_{48}N_4O_7S$): 704.3244, *found* 704.3257.

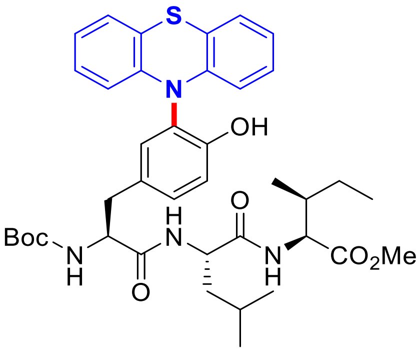

(4t)

Methyl [(*S*)-2-((*tert*-butoxycarbonyl)amino)-3-(4-hydroxy-3-(10*H*-phenothiazin-10-yl)phenyl)propanoyl]-*L*-leucyl-*L*-isoleucinate (4t). Following the general procedure A, using **3t** (0.30 mmol, 156 mg) and phenothiazine (0.25 mmol, 50 mg) provided 139 mg (78% yield) of **4t** as a white solid. Mp 129-130 ºC. Column chromatography (Hex/EtOAc 1:1). ^{1}H NMR (500 MHz, DMSO-d_6 at 80 ºC) δ 9.32 (s, 1H), 7.71 (dd, *J* = 11.4, 8.1 Hz, 2H), 7.22 (dd, *J* = 8.4, 2.2 Hz, 1H), 7.17 (d, *J* = 2.2 Hz, 1H), 7.03 (d, *J* = 8.3 Hz, 1H), 6.95 (dd, *J* = 7.4, 1.6 Hz, 2H), 6.85 (ddd, *J* = 8.4, 7.4, 1.7 Hz, 2H), 6.77 (td, *J* = 7.4, 1.3 Hz, 2H), 6.46 (br s, 1H), 6.14 (dd, *J* = 8.2, 1.2 Hz, 2H), 4.42 (td, *J* = 8.6, 5.6 Hz, 1H), 4.27 (dd, *J* = 8.2, 6.2 Hz, 1H), 4.21 (td, *J* = 9.4, 4.2 Hz, 1H), 3.63 (s, 3H), 3.10 – 2.90 (m, 1H), 2.74 (dd, *J* = 14.1, 10.0 Hz, 1H), 1.81 (ddt, *J* = 8.5, 6.6, 3.3 Hz, 1H), 1.73 – 1.60 (m, 1H), 1.60 – 1.33 (m, 2H), 1.29 (s, 9H), 1.26 – 1.11 (m, 1H), 0.97 – 0.79 (m, 12H). ^{13}C NMR (126 MHz, DMSO-d_6 at 80 ºC) δ 171.4, 171.0, 170.8, 153.3, 142.6, 131.6, 130.3, 130.2, 126.5, 126.1, 125.4, 121.4, 118.1, 116.6, 115.1, 77.7, 55.9, 50.8, 50.7, 40.6, 36.2, 36.0, 27.6, 27.6, 24.4, 23.7, 22.4, 21.4, 14.9, 10.4. HRMS (ESI-TOF) m/z: (M^+) *calcd.* for ($C_{39}H_{50}N_4O_7S$): 718.3400, *found* 718.3404.

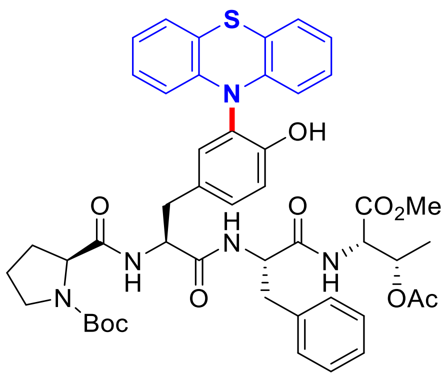

(**4u**)

***tert*-Butyl (*S*)-2-[((*S*)-1-(((*S*)-1-(((2*S*,3*S*)-3-acetoxy-1-methoxy-1-oxobutan-2-yl)amino)-1-oxo-3-phenylpropan-2-yl)amino)-3-(4-hydroxy-3-(10*H*-phenothiazin-10-yl)phenyl)-1-oxopropan-2-yl)carbamoyl]pyrrolidine-1-carboxylate (4u).** Following the general procedure A, using **3u** (0.30 mmol, 205 mg) and phenothiazine (0.25 mmol, 50 mg) provided 95 mg (43% yield) of **4u** as a white solid. Mp 131-133 ºC. Column chromatography (Hex/EtOAc 3:7). ^{1}H NMR (500 MHz, DMSO-d_6 at 80 ºC) δ 9.39 (s, 1H), 8.19 – 7.86 (m, 2H), 7.64 – 7.37 (m, 1H), 7.28 – 7.21 (m, 4H), 7.17 (td, *J* = 7.3, 6.3, 2.5 Hz, 2H), 7.10 (d, *J* = 2.2 Hz, 1H), 7.01 – 6.91 (m, 3H), 6.85 (td, *J* = 7.8, 1.7 Hz, 2H), 6.77 (t, *J* = 7.4 Hz, 2H), 6.11 (dd, *J* = 8.2, 1.3 Hz, 2H), 5.23 (qd, *J* = 6.4, 3.8 Hz, 1H), 4.73 (td, *J* = 8.4, 5.1 Hz, 1H), 4.62 (dd, *J* = 8.7, 3.9 Hz, 1H), 4.52 (td, *J* = 8.7, 4.3 Hz, 1H), 4.01 (dd, *J* = 8.6, 3.8 Hz, 1H), 3.63 (s, 3H), 3.33 – 3.18 (m, 2H), 3.09 (dt, *J* = 14.2, 4.9 Hz, 1H), 2.97 (dd, *J* = 14.2, 4.3 Hz, 1H), 2.87 (dd, *J* = 14.2, 8.7 Hz, 1H), 2.77 (dd, *J* = 14.1, 9.3 Hz, 1H), 1.96 (s, 3H), 1.76 – 1.66 (m, 1H), 1.66 – 1.51 (m, 2H), 1.30 (s, 9H), 1.17 (d, *J* = 6.5 Hz, 3H). ^{13}C NMR (126 MHz, DMSO-d_6 at 80 ºC) δ 171.3, 171.1, 170.5, 169.1, 168.8, 153.5, 142.6, 137.0, 131.6, 130.3, 130.0, 128.6, 127.5, 126.6, 126.0, 125.8, 125.5, 121.6, 118.2, 116.6, 115.2, 78.4, 68.9, 59.6, 54.9, 53.5, 53.2, 51.6, 46.1, 37.1, 36.4, 27.7, 22.8, 20.2, 16.1. HRMS (ESI-TOF) m/z: (M^+) *calcd.* for ($C_{47}H_{53}N_5O_{10}S$): 879.3513, *found* 879.3537.

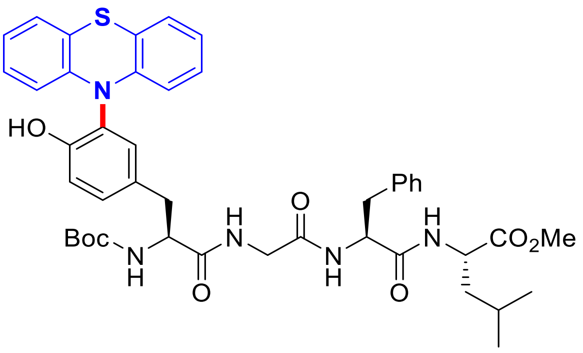

(**4v**)

Methyl [(*S*)-2-((*tert*-butoxycarbonyl)amino)-3-(4-hydroxy-3-(10*H*-phenothiazin-10-yl)phenyl)propanoyl)glycyl-*L*-phenylalanyl-*L*-leucinate (4v). Following the general

procedure A, using **3v** (0.30 mmol, 184 mg) and phenothiazine (0.25 mmol, 50 mg) provided 136.2 mg (67% yield) of **4v** as a white solid. Mp 130-131 ºC. Column chromatography (Hex/EtOAc 2:8). ^{1}H NMR (500 MHz, DMSO-d_6 at 80 ºC) δ 9.32 (s, 1H), 7.96 (d, *J* = 8.0 Hz, 1H), 7.83 (t, *J* = 5.6 Hz, 1H), 7.68 (d, *J* = 8.3 Hz, 1H), 7.28 – 7.20 (m, 5H), 7.20 – 7.06 (m, 2H), 7.03 (d, *J* = 8.3 Hz, 1H), 6.94 (dd, *J* = 7.5, 1.6 Hz, 2H), 6.84 (td, *J* = 7.7, 1.6 Hz, 2H), 6.77 (t, *J* = 7.5 Hz, 2H), 6.42 (br s, 1H), 6.13 (dd, *J* = 8.1, 1.3 Hz, 2H), 4.60 (td, *J* = 8.4, 5.1 Hz, 1H), 4.34 (td, *J* = 8.1, 6.1 Hz, 1H), 4.22 (td, *J* = 9.0, 4.4 Hz, 1H), 3.73 (dd, *J* = 16.4, 5.6 Hz, 1H), 3.69 – 3.57 (m, 4H), 3.06 (dt, *J* = 11.4, 5.8 Hz, 1H), 2.99 (dd, *J* = 14.1, 4.5 Hz, 1H), 2.90 – 2.82 (m, 1H), 2.82 – 2.69 (m, 1H), 1.71 – 1.62 (m, 1H), 1.62 – 1.53 (m, 2H), 1.29 (s, 9H), 0.89 (dt, *J* = 18.5, 6.4 Hz, 6H). ^{13}C NMR (126 MHz, DMSO-d_6 at 80 ºC) δ 171.9, 171.2, 170.3, 167.9, 154.5, 153.3, 142.6, 137.1, 131.6, 130.2, 129.5, 128.6, 127.5, 126.5, 126.0, 125.7, 125.5, 125.4, 121.5, 121.46, 120.3, 118.1, 116.7, 115.1, 115.1, 77.8, 55.5, 53.2, 51.1, 50.1, 41.9, 37.2, 36.4, 27.7, 23.8, 22.1, 21.1. HRMS (ESI-TOF) m/z: (M^+) *calcd.* for ($C_{44}H_{51}N_5O_8S$): 809.3458, *found* 809.3458.

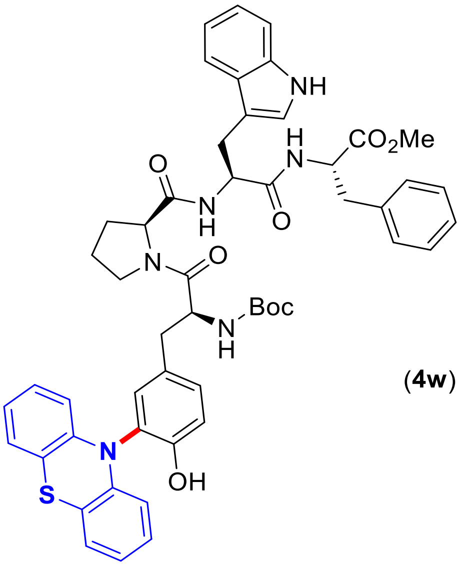

(**4w**)

Methyl [(*S*)-2-((*tert*-butoxycarbonyl)amino)-3-(4-hydroxy-3-(10*H*-phenothiazin-10-yl)phenyl)propanoyl)-*L*-prolyl-*L*-tryptophyl-*L*-phenylalaninate (4w). Following the general procedure A, using **3w** (0.30 mmol, 208 mg) and phenothiazine (0.25 mmol, 50 mg) provided 80 mg (35% yield) of **4w** as a white solid. Mp 134-135 ºC. Column chromatography (CH_2Cl_2/EtOAc 8:2). ^{1}H NMR (500 MHz, DMSO-d_6 at 80 ºC) δ 10.56 (s, 1H), 9.36 (s, 1H), 7.82 (d, *J* = 7.6 Hz, 1H), 7.53 (dd, *J* = 17.0, 7.7 Hz, 2H), 7.39 – 6.94 (m, 14H), 6.94 – 6.71 (m, 4H), 6.41 (br s, 1H), 6.14 (d, *J* = 8.2 Hz, 2H), 4.56 (dd, *J* = 11.3, 6.6 Hz, 2H), 4.38 (d, *J* = 7.8 Hz, 2H), 3.57 (s, 3H), 3.45 (s, 1H), 3.17 (dd, *J* = 14.9,

6.0 Hz, 1H), 3.12 – 2.90 (m, 4H), 2.90 – 2.59 (m, 2H), 2.04 – 1.73 (m, 4H), 1.28 (s, 9H). ^{13}C NMR (126 MHz, DMSO-d_6 at 80 ºC) δ 170.9, 170.7, 170.5, 170.3, 154.5, 153.4, 142.6, 136.6, 135.8, 131.6, 130.6, 130.0, 128.5, 127.7, 127.1, 126.5, 126.0, 125.9, 125.5, 123.1, 121.5, 120.3, 118.1, 117.7, 116.7, 115.1, 110.7, 109.5, 77.7, 59.4, 53.1, 53.0, 51.1, 46.3, 36.6, 35.6, 28.4, 27.9, 27.7, 26.9, 23.9. HRMS (ESI-TOF) m/z: (M$^+$) *calcd.* for ($C_{52}H_{54}N_6O_8S$): 922.3724, *found* 922.3738.

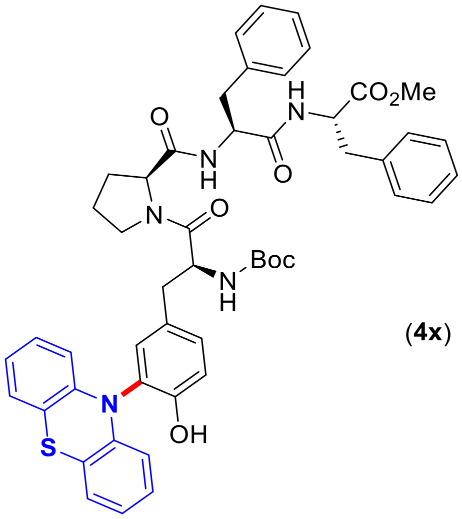

(**4x**)

Methyl [(*S*)-2-((*tert*-butoxycarbonyl)amino)-3-(4-hydroxy-3-(10*H*-phenothiazin-10-yl)phenyl)propanoyl)-*L*-prolyl-*L*-phenylalanyl-*L*-phenylalaninate (4x). Following the general procedure A, using **3x** (0.30 mmol, 206 mg) and phenothiazine (0.25 mmol, 50 mg) provided 91 mg (41% yield) of **4x** as a white solid. Mp 75-77 ºC. Column chromatography (CH_2Cl_2/EtOAc 8:2). ^{1}H NMR (500 MHz, DMSO-d_6 at 80 ºC) δ 8.12 – 7.82 (m, 2H), 7.52 (s, 1H), 7.36 – 7.06 (m, 18H), 7.04 (d, *J* = 8.2 Hz, 1H), 6.95 (dd, *J* = 7.4, 1.6 Hz, 1H), 6.87 – 6.80 (m, 1H), 6.77 (t, *J* = 7.3 Hz, 1H), 6.47 (s, 1H), 6.13 (d, *J* = 8.2 Hz, 2H), 4.54 (td, *J* = 8.1, 5.8 Hz, 2H), 4.44 – 4.25 (m, 2H), 3.61 (s, 3H), 3.11 – 2.68 (m, 8H), 1.95 – 1.82 (m, 4H), 1.29 (s, 9H). ^{13}C NMR (126 MHz, DMSO-d_6 at 80 ºC) δ 171.7, 171.1, 170.7, 170.4, 168.9, 154.6, 153.6, 142.8, 137.3, 137.1, 136.8, 131.9, 130.8, 130.2, 128.8, 128.8, 128.7, 127.9, 127.9, 127.7, 127.6, 127,0 126.7, 126.2, 126.1, 125.9, 125.7, 121.7, 118.3, 116.9, 115.3, 77.9, 59.6, 53.5, 53.3, 53.3, 51.4, 51.3, 51.3, 46.5, 37.0, 36.8, 36.8, 35.9, 27.9, 24.1, 21.9. HRMS (ESI-TOF) m/z: (M$^+$) *calcd.* for ($C_{50}H_{53}N_5O_8S$): 883.3615, *found* 883.3604.

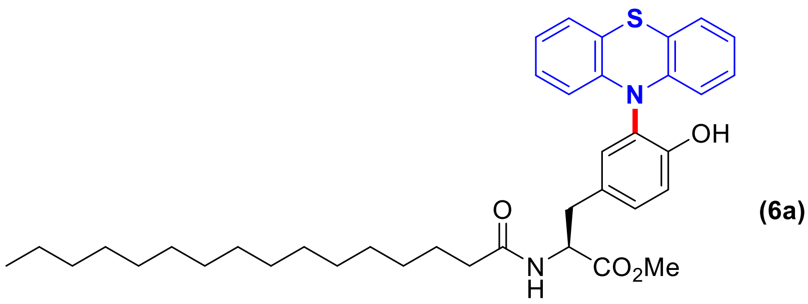

(6a)

Methyl (*S*)-3-[4-hydroxy-3-(10*H*-phenothiazin-10-yl)phenyl]-2-palmitamidopropanoate (6a). Following the general procedure A, using **5a**[14] (0.30 mmol, 130 mg) and phenothiazine (0.25 mmol, 50 mg) provided 144 mg (92% yield) of **6a** as a reddish oil. Column chromatography (Hex/EtOAc 7:3). ^{1}H NMR (500 MHz, DMSO-d_6 at 80 ºC) δ 9.39 (s, 1H), 7.78 (d, *J* = 8.0 Hz, 1H), 7.20 (dd, *J* = 8.3, 2.2 Hz, 1H), 7.08 – 7.01 (m, 2H), 6.93 (dd, *J* = 7.5, 1.7 Hz, 1H), 6.87 – 6.80 (m, 2H), 6.76 (t, *J* = 7.4 Hz, 2H), 6.08 (d, *J* = 8.1 Hz, 2H), 4.52 (td, *J* = 8.9, 8.5, 5.9 Hz, 1H), 3.58 (s, 3H), 3.13 – 2.94 (m, 1H), 2.94 – 2.80 (m, 1H), 2.02 (td, *J* = 7.2, 3.4 Hz, 2H), 1.41 (t, *J* = 7.1 Hz, 2H), 1.27 (s, 24H), 0.87 (td, *J* = 6.9, 2.0 Hz, 3H). ^{13}C NMR (126 MHz, DMSO-d_6 at 80 ºC) δ 171.6, 171.5, 153.5, 142.5, 131.7, 130.0, 129.5, 126.4, 126.1, 125.4, 121.5, 118.0, 117.0, 115.0, 53.1, 51.0, 35.6, 34.7, 30.7, 28.4, 28.4, 28.4, 28.3, 28.1, 28.1, 24.5, 21.4, 13.2. HRMS (ESI-TOF) m/z: (M^+) *calcd.* for ($C_{38}H_{50}N_2O_4S$): 630.3491, *found* 630.3488.

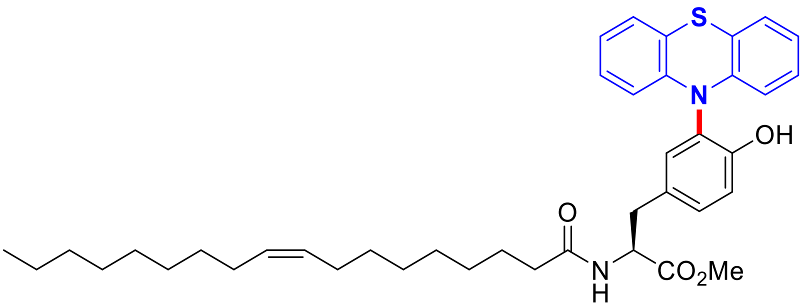

(6b)

Methyl (*S*)-3-[4-hydroxy-3-(10*H*-phenothiazin-10-yl)phenyl]-2-oleamidopropanoate (6b). Following the general procedure A, using **5b**[15] (0.30 mmol, 138 mg) and phenothiazine (0.25 mmol, 50 mg) provided 158 mg (96% yield) of **6b** as a reddish oil. Column chromatography (Hex/EtOAc 7:3). ^{1}H NMR (500 MHz, DMSO-d_6 at 80 ºC) δ 9.39 (s, 1H), 7.78 (d, *J* = 8.0 Hz, 1H), 7.20 (dd, *J* = 8.3, 2.3 Hz, 1H), 7.09 – 7.03 (m, 2H), 6.94 (dd, *J* = 7.6, 1.6 Hz, 1H), 6.84 (ddd, *J* = 8.4, 7.4, 1.6 Hz, 2H), 6.76 (td, *J* = 7.4, 1.3 Hz, 2H), 6.09 (dd, *J* = 8.2, 1.2 Hz, 2H), 5.49 – 5.20 (m, 2H), 4.52 (td, *J* = 8.4, 5.8 Hz,

[14] Liu, B.; Cui, C.; Duan, W.; Zhao, M.; Peng, S.; Wang, L.; Liu, H.; Cui, G. *Eur. J. Med. Chem.* **2009**, *44*, 3596.
[15] Osornio, Y. M.; Uebelhart, P.; Bosshard, S.; Konrad, F.; Siegel, J. S.; Landau, E. M. *J. Org. Chem.* **2012**, *77*, 10583.

1H), 3.58 (s, 3H), 3.01 (dd, J = 14.0, 5.7 Hz, 1H), 2.89 (dd, J = 14.0, 8.8 Hz, 1H), 2.04 – 1.98 (m, 6H), 1.49 – 1.38 (m, 2H), 1.38 – 1.12 (m, 20H), 0.88 (t, J = 6.8 Hz, 3H). ^{13}C NMR (126 MHz, DMSO-d_6 at 80 ºC) δ 171.6, 171.4, 153.5, 142.4, 131.6, 130.0, 129.5, 129.1, 129.1, 126.4, 126.1, 125.4, 121.4, 118.0, 117.0, 115.0, 53.1, 51.0, 35.6, 34.7, 30.7, 28.59, 28.57, 28.2, 28.1, 28.04, 28.02, 28.00, 26.15, 26.13, 24.5, 21.4. HRMS (ESI-TOF) m/z: (M^+) *calcd.* for ($C_{40}H_{52}N_2O_4S$): 656.3648, *found* 656.3661.

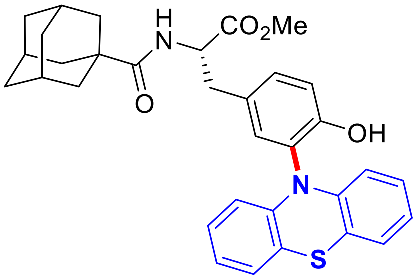

(6c)

Methyl (*S*)-(2-adamantane-1-carboxamido)-3-[4-hydroxy-3-(10*H*-phenothiazin-10-yl)phenyl]propanoate (6c). Following the general procedure A, using **5c** (0.30 mmol, 107 mg) and phenothiazine (0.25 mmol, 50 mg) provided 123 mg (89% yield) of **6c** as a pink solid. Mp 187-188 ºC. Column chromatography (Hex/EtOAc 7:3). ^{1}H NMR (500 MHz, DMSO-d_6 at 80 ºC) δ 9.39 (s, 1H), 7.19 (dd, J = 8.3, 2.2 Hz, 1H), 7.12 – 7.03 (m, 3H), 6.94 (dd, J = 7.5, 1.6 Hz, 1H), 6.85 (td, J = 7.7, 1.6 Hz, 2H), 6.77 (t, J = 7.4 Hz, 2H), 6.10 (dd, J = 8.1, 1.2 Hz, 2H), 4.53 (ddd, J = 9.2, 8.0, 5.0 Hz, 1H), 3.62 (s, 3H), 3.06 (dd, J = 14.0, 5.0 Hz, 1H), 2.99 (dd, J = 14.0, 9.3 Hz, 1H), 1.95 – 1.79 (m, 3H), 1.71 – 1.62 (m, 9H), 1.56 (dq, J = 12.4, 2.2 Hz, 3H). ^{13}C NMR (126 MHz, DMSO-d_6 at 80 ºC) δ 176.1, 171.5, 153.6, 142.5, 131.6, 130.1, 129.8, 126.4, 126.1, 125.4, 121.5, 118.2, 116.8, 115.1, 52.7, 51.1, 38.1, 35.7, 34.9. HRMS (ESI-TOF) m/z: (M^+) *calcd.* for ($C_{33}H_{34}N_2O_4S$): 554.2239, *found* 554.2261.

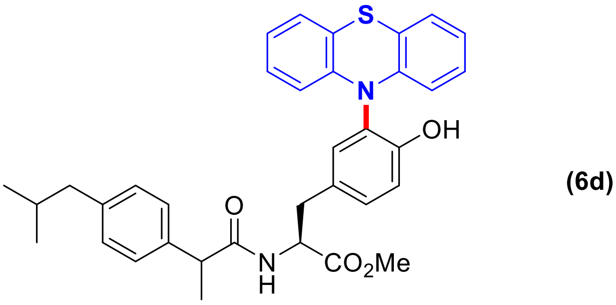

(6d)

Methyl (*S*)-2-[(*S*)-3-((3,3-dimethylbutyl)amino)-4-(((*S*)-1-methoxy-1-oxo-3-phenylpropan-2-yl)amino)-4-oxobutanamido]-3-[4-hydroxy-3-(10*H*-phenothiazin-10-yl)phenyl]propanoate (6d). Following the general procedure A, using **5d** (0.30 mmol, 167 mg) and phenothiazine (0.25 mmol, 50 mg) provided 75.1 mg (40% yield) of

6d as a reddish solid. The latter was obtained as a diastereomeric mixture (*dr* 1:1) and the following data correspond to the mixture of both isomers. Mp 70-71 ºC. Column chromatography (Hex/EtOAc 7:3). ^{1}H NMR (500 MHz, DMSO-d_6 at 80 ºC) δ 9.39 (s, 1H), 7.81 (dd, *J* = 31.8, 7.8 Hz, 1H), 7.23 – 7.10 (m, 3H), 7.10 – 6.97 (m, 4H), 6.95 (ddd, *J* = 7.5, 3.7, 1.6 Hz, 2H), 6.88 – 6.73 (m, 3H), 6.09 (ddd, *J* = 21.7, 8.1, 1.3 Hz, 2H), 4.54 (dtd, *J* = 24.9, 8.2, 5.8 Hz, 1H), 3.68 – 3.49 (m, 4H), 3.05 (dd, *J* = 14.0, 5.7 Hz, 1H), 3.00 – 2.85 (m, 1H), 2.42 (dd, *J* = 7.1, 3.3 Hz, 2H), 1.84 (dtd, *J* = 13.5, 6.7, 1.5 Hz, 1H), 1.31 (d, *J* = 7.1 Hz, 1.5H), 1.22 (d, *J* = 7.1 Hz, 1.5H), 0.88 (dd, *J* = 6.6, 1.4 Hz, 6H). ^{13}C NMR (126 MHz, DMSO-d_6 at 80 ºC) δ 173.0, 172.9, 171.2, 171.19, 153.5, 142.5, 138.7, 138.66, 138.6, 138.1, 131.6, 131.5, 130.1, 130.0, 129.4, 129.2, 128.14, 128.11, 126.5, 126.48, 126.46, 125.4, 121.5, 121.46, 118.1, 118.0, 117.0, 115.04, 115.00, 53.3, 53.1, 51.0, 50.9, 44.1, 44.08, 43.9, 43.8, 35.5, 35.4, 28.8, 21.63, 21.61, 18.0, 17.6. HRMS (ESI-TOF) m/z: (M^+) *calcd.* for ($C_{35}H_{36}N_2O_4S$): 580.2396, *found* 580.2407.

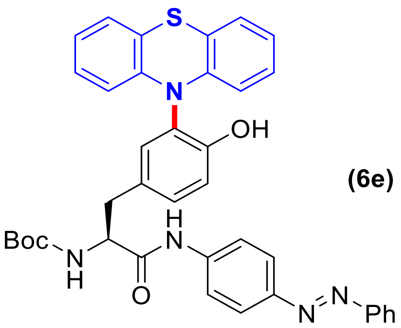

(6e)

***tert*-Butyl (*S*)-[3-(4-hydroxy-3-(10*H*-phenothiazin-10-yl)phenyl)-1-oxo-1-((4-(phenyldiazenyl)phenyl)amino)propan-2-yl]carbamate (6e).** Following the general procedure A, using **5e** (0.30 mmol, 138 mg) and phenothiazine (0.25 mmol, 50 mg) provided 80 mg (49% yield) of **6e** as a yellow solid. Mp 118-120 ºC. Column chromatography (Hex/EtOAc 6:4). ^{1}H NMR (500 MHz, DMSO-d_6 at 80 ºC) δ 10.04 (d, *J* = 3.2 Hz, 1H), 9.37 (s, 1H), 7.92 – 7.71 (m, 6H), 7.63 – 7.46 (m, 3H), 7.41 – 7.25 (m, 1H), 7.20 (d, *J* = 2.1 Hz, 1H), 7.09 (dd, *J* = 39.6, 8.2 Hz, 1H), 6.93 (dd, *J* = 7.4, 1.7 Hz, 1H), 6.87 – 6.70 (m, 3H), 6.59 (br s, 1H), 6.11 (d, *J* = 8.2 Hz, 2H), 4.44 (q, *J* = 3.1 Hz, 1H), 3.19 – 2.84 (m, 2H), 1.34 (s, 9H). ^{13}C NMR (126 MHz, DMSO-d_6 at 80 ºC) δ 170.3, 154.6, 153.5, 151.9, 147.6, 142.5, 141.3, 134.9, 131.6, 130.5, 130.3, 130.26, 129.7, 129.72, 128.7, 126.5, 126.2, 125.4, 122.9, 122.89, 121.7, 121.4, 120.1, 119.5, 119.3, 118.1, 116.7, 115.1, 77.9, 64.0, 56.4, 36.5, 27.7. HRMS (ESI-TOF) m/z: (M^+) *calcd.* for ($C_{38}H_{35}N_5O_4S$): 657.2410, *found* 657.2425.

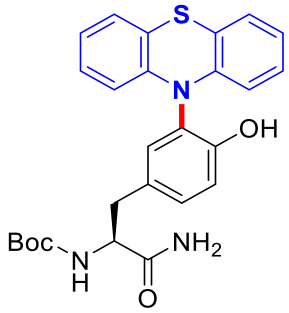

(6f)

***tert*-Butyl (*S*)-[1-amino-3-(4-hydroxy-3-(10*H*-phenothiazin-10-yl)phenyl)-1-oxopropan-2-yl]carbamate (6f).** Following the general procedure A, using **5f**[16] (0.30 mmol, 84.1 mg) and phenothiazine (0.25 mmol, 50 mg) provided 48 mg (40% yield) of **6f** as a brownish solid. Mp 148-150 ºC. Column chromatography (Hex/EtOAc 2:8). ^{1}H NMR (500 MHz, DMSO-d_6 at 80 ºC) δ 9.38 (s, 1H), 7.21 (dd, *J* = 8.3, 2.2 Hz, 1H), 7.12 (d, *J* = 2.2 Hz, 1H), 7.03 (d, *J* = 8.3 Hz, 1H), 6.94 (dd, *J* = 7.5, 1.6 Hz, 2H), 6.88 – 6.81 (m, 2H), 6.77 (t, *J* = 7.5 Hz, 2H), 6.32 (br s, 1H), 6.16 – 6.05 (m, 2H), 4.14 (td, *J* = 9.0, 4.6 Hz, 1H), 2.98 (dd, *J* = 14.0, 4.6 Hz, 1H), 2.73 (dd, *J* = 14.0, 9.5 Hz, 1H), 1.29 (s, 9H). ^{13}C NMR (126 MHz, DMSO-d_6 at 80 ºC) δ 173.8, 155.4, 154.2, 143.5, 132.5, 131.3, 131.2, 127.5, 126.4, 122.4, 119.0, 117.6, 116.0, 78.5, 56.2, 37.4, 28.6. HRMS (ESI-TOF) m/z: (M$^+$) *calcd.* for ($C_{26}H_{27}N_3O_4S$): 477.1722, *found* 477.1732.

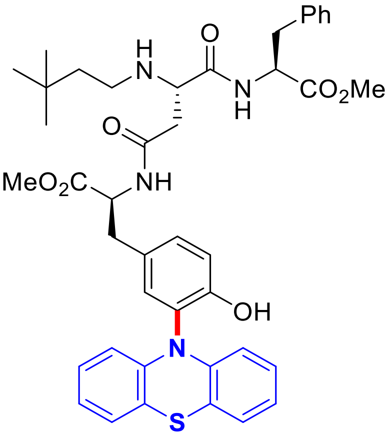

(6g)

Methyl (*S*)-2-[(*S*)-3-((3,3-dimethylbutyl)amino)-4-(((*S*)-1-methoxy-1-oxo-3-phenylpropan-2-yl)amino)-4-oxobutanamido]-3-[4-hydroxy-3-(10*H*-phenothiazin-10-yl)phenyl]propanoate (6g). Following the general procedure A, using **5g** (0.30 mmol, 167 mg) and phenothiazine (0.25 mmol, 50 mg) provided 75.1 mg (40% yield) of **6g** as a brownish solid. Mp 129-130 ºC. Column chromatography (Hex/EtOAc 4:6). ^{1}H NMR (500 MHz, DMSO-d_6 at 80 ºC) δ 9.46 (s, 1H), 8.30 (d, *J* = 7.7 Hz, 1H), 8.03 (d, *J* = 8.2 Hz, 1H), 7.27 (t, *J* = 7.2 Hz, 2H), 7.23 – 7.14 (m, 4H), 7.10 – 7.01 (m, 2H), 6.94 (dd, *J* =

[16] Armstrong, A.; Emmerson, D. P. G.; Milner, H. J.; Sheppard, R. J. *J. Org. Chem.* **2014**, *79*, 3895.

7.5, 1.6 Hz, 1H), 6.84 (ddd, J = 8.4, 7.3, 1.6 Hz, 2H), 6.76 (td, J = 7.4, 1.3 Hz, 2H), 6.08 (dd, J = 8.3, 1.2 Hz, 2H), 4.61 (dd, J = 8.0, 5.6 Hz, 1H), 4.58 – 4.49 (m, 1H), 3.63 (s, 3H), 3.55 (s, 3H), 3.30 (dd, J = 9.9, 3.7 Hz, 1H), 3.14 – 2.82 (m, 3H), 2.40 – 2.29 (m, 3H), 2.16 (dd, J = 15.1, 9.5 Hz, 1H), 1.37 – 1.14 (m, 2H), 0.82 (s, 9H). ^{13}C NMR (126 MHz, DMSO-d_6 at 80 °C) δ 172.3, 171.2, 171.1, 169.7, 153.6, 142.4, 136.5, 132.1, 131.7, 130.8, 130.1, 129.2, 128.5, 127.7, 126.6, 126.1, 126.1, 125.5, 121.6, 121.3, 118.0, 117.1, 116.5, 115.0, 58.7, 53.3, 52.5, 51.3, 51.1, 43.1, 37.7, 36.6, 35.8, 28.9. HRMS (ESI-TOF) m/z: (M$^+$) *calcd.* for ($C_{42}H_{48}N_4O_7S$): 752.3244, *found* 752.3276.

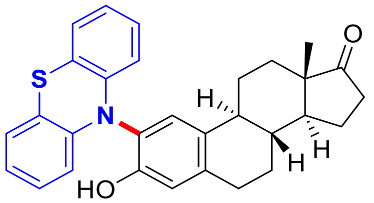

(6ha)

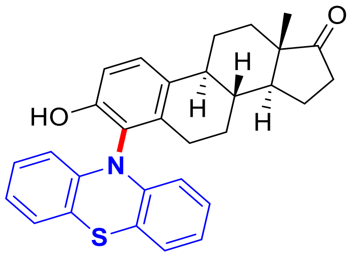

(6ha')

(8*R*,9*S*,13*S*,14*S*)-3-Hydroxy-13-methyl-2-(10*H*-phenothiazin-10-yl)-6,7,8,9,11,12,13,14,15,16-decahydro-17*H*-cyclopenta[*a*]phenanthren-17-one (6ha). Following the general procedure A, using estrone (0.5 mmol, 135 mg) and phenothiazine (0.25 mmol, 50 mg) provided 76 mg (65 % yield) and 41 mg (35% yield) of **6ha** and **6ha'**, respectively, both of them as a light-yellow solid. *Both isomers were separated by column chromatography (CH_2Cl_2) and independently characterized*. **Major isomer 6ha:** Mp 129-132 °C. ^{1}H NMR (500 MHz, DMSO-d_6 at 80 °C) δ 9.22 (s, 1H), 7.06 (s, 1H), 6.94 (d, J = 7.5 Hz, 2H), 6.90 – 6.82 (m, 3H), 6.76 (t, J = 7.4 Hz, 2H), 6.13 (d, J = 8.2 Hz, 2H), 2.94 – 2.88 (m, 2H), 2.48 – 2.39 (m, 1H), 2.33 – 2.18 (m, 2H), 2.11 – 1.96 (m, 3H), 1.74 (d, J = 9.0 Hz, 1H), 1.63 – 1.20 (m, 6H), 0.85 (s, 3H). ^{13}C NMR (126 MHz, DMSO-d_6 at 80 °C) δ 218.4, 152.6, 142.8, 137.9, 132.3, 127.2, 126.6, 125.5, 124.3, 121.4, 118.2, 116.8, 115.2, 49.4, 46.8, 423.0, 37.4, 34.9, 31.0, 28.3, 25.5, 25.2, 20.6, 13.2. HRMS (ESI-TOF) m/z: (M$^+$) *calcd.* for ($C_{30}H_{29}NO_2S$): 467.1919, *found* 467.1924. **Minor isomer 6ha'**: Mp 114-117 °C. ^{1}H NMR (500 MHz, DMSO-d_6 at 80 °C) δ 9.24 (s, 1H), 7.29 (d, J = 8.6 Hz, 1H), 6.94 (d, J = 8.5 Hz, 1H), 6.92 (dt, J = 7.5, 1.9 Hz, 2H), 6.84 (ddt, J = 8.5, 7.3, 1.5 Hz, 2H), 6.75 (tt, J = 7.4, 1.5 Hz, 2H), 6.08 (dd, J = 8.2, 1.2 Hz, 1H), 6.02 (dd, J = 8.2, 1.3 Hz, 1H), 2.87 (ddd, J = 17.7, 6.4, 2.2 Hz, 1H), 2.60 (ddd, J = 18.0, 11.5, 6.9 Hz, 1H), 2.45 – 2.34 (m, 2H), 2.29 – 2.20 (m, 1H), 2.03 (dt, J = 18.8, 8.7 Hz, 1H), 1.96 – 1.85 (m, 2H), 1.81 (dt, J = 12.6, 3.0 Hz, 1H), 1.58 – 1.20 (m, 6H), 0.84 (s, 3H). ^{13}C NMR (126 MHz, DMSO-d_6 at 80 °C) δ 218.5, 153.1, 141.3, 141.2, 135.7, 132.2, 126.8, 126.7, 125.8,

125.5, 124.4, 121.6, 121.5, 118.2, 118.1, 114.4, 114.3, 113.7, 49.4, 46.8, 43.1, 37.1, 34.9, 31.0, 25.2, 25.1, 23.7, 20.6, 13.2. HRMS (ESI-TOF) m/z: (M^+) *calcd*. for ($C_{30}H_{29}NO_2S$): 467.1919, *found* 467.1919.

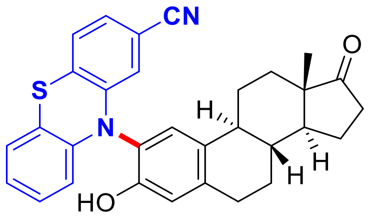

(6hb)

10-[(8*R*,9*S*,13*S*,14*S*)-3-Hydroxy-13-methyl-17-oxo-7,8,9,11,12,13,14,15,16,17 decahydro-6*H*-cyclopenta[*a*]phenanthren-2-yl]-10*H*-phenothiazine-2-carbonitrile (6hb). Following the general procedure A, using estrone (0.5 mmol, 135 mg) and 10*H*-phenothiazine-2-carbonitrile (0.25 mmol, 56 mg) provided 32 mg (26 % yield) of **6hb** as a yellow solid. Mp 135-140 ºC. Column chromatography (CH_2Cl_2). ^{1}H NMR (500 MHz, DMSO-d_6 at 80 ºC) δ 9.44 (s, 1H), 7.18 – 7.06 (m, 3H), 6.96 (dd, *J* = 7.6, 1.6 Hz, 1H), 6.94 – 6.87 (m, 2H), 6.82 (td, *J* = 7.4, 1.3 Hz, 1H), 6.22 (d, *J* = 1.5 Hz, 1H), 6.10 (dd, *J* = 8.3, 1.2 Hz, 1H), 2.97 – 2.87 (m, 2H), 2.44 (dd, *J* = 18.5, 8.7 Hz, 1H), 2.33 – 2.21 (m, 2H), 2.13 – 1.96 (m, 3H), 1.75 (dd, *J* = 9.0, 2.3 Hz, 1H), 1.66 – 1.22 (m, 6H), 0.86 (s, 3H). ^{13}C NMR (126 MHz, DMSO-d_6 at 80 ºC) δ 218.4, 152.2, 143.3, 141.7, 138.6, 132.8, 127.3, 126.8, 126.5, 125.9, 125.7, 125.0, 123.2, 122.4, 118.2, 117.0, 117.0, 116.5, 115.6, 109.1, 49.4, 46.8, 42.9, 37.3, 34.9, 31.0, 28.3, 25.4, 25.2, 20.6, 13.2. HRMS (ESI-TOF) m/z: (M^+) *calcd*. for ($C_{31}H_{28}N_2O_2S$): 492.1871, *found* 492.1879.

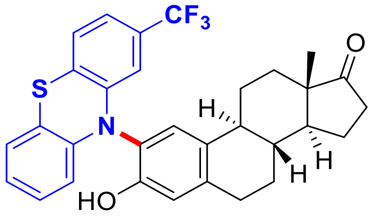

(6hc)

(8*R*,9*S*,13*S*,14*S*)-3-Hydroxy-13-methyl-2-[2-(trifluoromethyl)-10*H*-phenothiazin-10-yl]-6,7,8,9,11,12,13,14,15,16-decahydro-17*H*-cyclopenta[*a*]phenanthren-17-one (6hc). Following the general procedure A, using estrone (0.5 mmol, 135 mg) and 2-(trifluoromethyl)-10*H*-phenothiazine (0.25 mmol, 67 mg) provided 50 mg (37% yield) and 20 mg (15% yield) of **6hc** and **6hc'**, respectively, both of them as a yellow solid. *Both isomers were separated by column chromatography (CH_2Cl_2) and the following data correspond to the major isomer* **6hc**. Mp 122-124 ºC. ^{1}H NMR (500 MHz, DMSO-d_6 at 80 ºC) δ 9.41 (s, 1H), 7.15 (d, *J* = 8.0 Hz, 1H), 7.10 (s, 1H), 7.06 (d, *J* = 8.0 Hz, 1H), 6.97 (dd, *J* = 7.5, 1.6 Hz, 1H), 6.94 – 6.87 (m, 2H), 6.82 (td, *J* = 7.4, 1.3 Hz, 1H), 6.34 (s, 1H),

6.12 (d, J = 8.2 Hz, 1H), 2.93 (m, 2H), 2.44 (dd, J = 18.5, 8.6 Hz, 1H), 2.32 – 2.21 (m, 2H), 2.11 – 1.95 (m, 3H), 1.79 – 1.69 (m, 1H), 1.65 – 1.23 (m, 6H), 0.85 (s, 3H). ^{13}C NMR (126 MHz, DMSO-d_6 at 80 ºC) δ 218.4, 152.4, 143.4, 142.1, 138.5, 132.7, 127.5 (q, J = 31.5 Hz), 127.1, 126.9, 126.3, 125.7, 124.0, 123.5 (q, $J_{C\text{-}F}$ = 272.2 Hz), 123.4, 122.3, 117.9 (q, $J_{C\text{-}F}$ = 3.8 Hz), 117.4, 116.8, 115.6, 110.7 (q, $J_{C\text{-}F}$ = 3.8 Hz), 49.4, 46.8, 42.9, 37.4, 34.9, 30.9, 28.3, 25.4, 25.2, 20.6, 13.2. ^{19}F NMR (376 MHz, $CDCl_3$) δ -62.80. HRMS (ESI-TOF) m/z: (M^+) *calcd*. for ($C_{31}H_{28}F_3NO_2S$): 535.1793, *found* 535.1812.

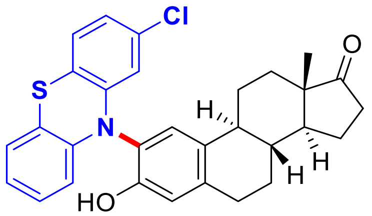

(6hd)

(8*R*,9*S*,13*S*,*14S*)-2-(2-Chloro-10*H*-phenothiazin-10-yl)-3-hydroxy-13-methyl-6,7,8,9,11,12,13,14,15,16-decahydro-17*H*-cyclopenta[*a*]phenanthren-17-one (6hd). Following the general procedure A, using estrone (0.5 mmol, 135 mg) and 2-chloro-10*H*-phenothiazine (0.25 mmol, 58 mg) provided 92 mg (73% yield) and 27 mg (22% yield) of **6hd** and **6hd'**, respectively, both of them as a white-yellowish solid. *Both isomers were separated by column chromatography (CH_2Cl_2) and the following data correspond to the major isomer* **6hd**. Mp 146-150 ºC. ^1H NMR (400 MHz, MeOD-d_4) δ 7.08 (s, 1H), 6.91 (dd, J = 7.4, 1.8 Hz, 1H), 6.87 (d, J = 8.2 Hz, 1H), 6.85 – 6.71 (m, 4H), 6.15 (dd, J = 8.0, 1.4 Hz, 1H), 6.11 (d, J = 2.1 Hz, 1H), 2.98-2.94 (m, 2H), 2.48 (dd, J = 18.3, 8.6 Hz, 1H), 2.34 – 2.21 (m, 2H), 2.18 – 2.02 (m, 3H), 1.84 (dd, J = 9.0, 2.4 Hz, 1H), 1.74 – 1.25 (m, 6H), 0.91 (s, 3H). ^{13}C NMR (126 MHz, DMSO-d_6 at 80 ºC) δ 218.4, 152.3, 144.2, 142.1, 138.4, 132.6, 131.2, 126.9, 126.8, 126.7, 125.6, 123.7, 122.1, 121.0, 118.0, 117.4, 116.9, 115.5, 114.6, 49.4, 46.8, 42.9, 37.4, 34.9, 31.0, 28.3, 25.4, 25.2, 20.6, 13.2. HRMS (ESI-TOF) m/z: (M^+) *calcd*. for ($C_{30}H_{28}ClNO_2S$): 501.1529, *found* 501.1543.

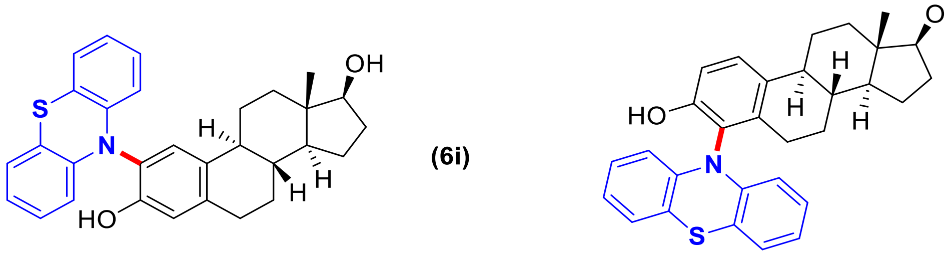

(6i) **(6i')**

(8*R*,9*S*,13*S*,14*S*,17*S*)-13-Methyl-2-(10*H*-phenothiazin-10-yl)-7,8,9,11,12,13,14,15,16,17-decahydro-6*H*-cyclopenta[*a*]phenanthrene-3,17-diol (6i). Following the general procedure A, using estradiol (0.5 mmol, 136 mg) and phenothiazine (0.25 mmol, 50 mg)

provided 77 mg (66% yield) and 31 mg (26% yield) of **6i** and **6i'**, respectively, both of them as a white solid. *Both isomers were separated by column chromatography (CH_2Cl_2) and independently characterized.* **Major isomer 6i:** Mp 141-145ºC. ^{1}H NMR (500 MHz, DMSO-d_6 at 80 ºC) δ 7.05 (s, 1H), 6.94 (dd, J = 7.5, 1.6 Hz, 2H), 6.86 (ddd, J = 8.5, 7.3, 1.6 Hz, 2H), 6.84 (s, 1H), 6.76 (t, J = 7.4 Hz, 2H), 6.14 (dd, J = 8.2, 1.3 Hz, 2H), 3.55 (t, J = 8.5 Hz, 1H), 2.89 – 2.80 (m, 2H), 2.22-2.14 (m, 2H), 1.96 – 1.81 (m, 3H), 1.63 (dddd, J = 12.3, 9.7, 7.0, 3.3 Hz, 1H), 1.46 – 1.24 (m, 5H), 1.21 – 1.11 (m, 2H), 0.71 (s, 3H). ^{13}C NMR (126 MHz, DMSO-d_6 at 80 ºC) δ 152.4, 142.8, 137.9, 132.8, 127.1, 126.6, 125.5, 124.2, 121.4, 118.2, 116.7, 115.1, 79.7, 49.4, 43.0, 42.5, 38.1, 36.2, 29.6, 28.4, 26.4, 25.8, 22.4, 10.8. HRMS (ESI-TOF) m/z: (M$^+$) *calcd.* for ($C_{30}H_{31}NO_2S$): 469.2075, *found* 469.2079. **Minor isomer 6i':** Mp 115-119ºC. ^{1}H NMR (500 MHz, DMSO-d_6 at 80 ºC) δ 9.18 (s, 1H), 7.28 (d, J = 8.5 Hz, 1H), 6.95 – 6.87 (m, 3H), 6.86 – 6.81 (m, 2H), 6.78 – 6.70 (m, 2H), 6.07 (d, J = 8.2 Hz, 1H), 6.00 (d, J = 8.3 Hz, 1H), 4.13 (s, 1H), 3.55 (t, J = 8.4 Hz, 1H), 2.81 (dd, J = 17.8, 6.0 Hz, 1H), 2.58 – 2.51 (m, 1H), 2.35 – 2.24 (m, 1H), 2.17 (td, J = 11.2, 4.0 Hz, 1H), 1.94 – 1.82 (m, 2H), 1.81 – 1.71 (m, 1H), 1.58 – 1.52 (m, 1H), 1.49 – 1.05 (m, 7H), 0.69 (d, J = 1.7 Hz, 3H). ^{13}C NMR (126 MHz, DMSO-d_6 at 80 ºC) δ 152.9, 141.3, 141.2, 135.7, 132.8, 126.8, 126.7, 125.8, 125.5, 125.4, 124.3, 121.5, 121.5, 118.1, 118.1, 114.4, 114.2, 113.6, 79.7, 49.3, 43.2, 42.4, 37.8, 36.2, 29.6, 25.9, 25.7, 23.8, 22.2, 10.7. HRMS (ESI-TOF) m/z: (M$^+$) *calcd.* for ($C_{30}H_{31}NO_2S$): 469.2075, *found* 469.2060.

5- Gram-Scale Experiments

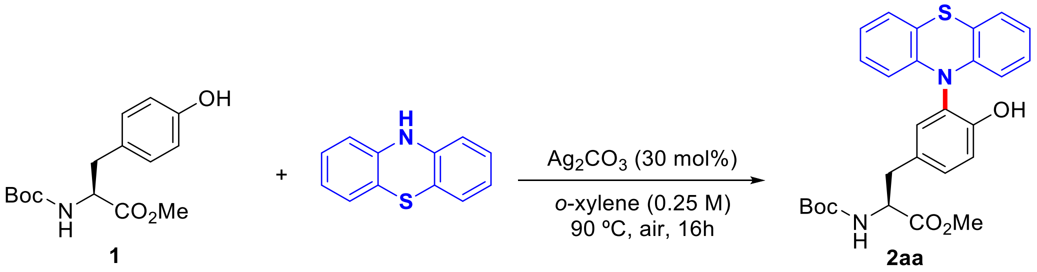

<u>**Gram-scale :**</u> A round-bottom flask containing a stirring bar was charged with Boc-Tyr-OMe (6.02 mmol, 1.78 g), phenothiazine (5.02 mmol, 1.00 g) and Ag_2CO_3 (30 mol%, 1.506 mmol, 415.3 mg). Then, *o*-xylene (20 mL) was added under air. The reaction tube was next warmed up to 90 ºC in a heating block and stirred for 16 hours. The mixture was then allowed to warm to room temperature, evaporated under vacuum and the resulting crude was then purified by column chromatography (Hex/EtOAc, 7:3) to provide 2.21g (89% yield) of **2aa** as a white solid.

<u>**Multigram-scale :**</u> A round-bottom flask containing a stirring bar was charged with Boc-Tyr-OMe (18.06 mmol, 5.34 g), phenothiazine (15.06 mmol, 3.00 g) and Ag_2CO_3 (30 mol%, 4.518 mmol, 1.24 g). Then, *o*-xylene (60 mL) was added under air. The reaction tube was next warmed up to 90 ºC in a heating block and stirred for 16 hours. The mixture was then allowed to warm to room temperature, evaporated under vacuum and the resulting crude was then purified by column chromatography (Hex/EtOAc, 7:3) to provide 6.91 g (93% yield) of **2aa** as a white solid.

6.- Peptide 7[Tb] Synthesis and Spectroscopic Characterization

6.1.-Materials

Amino acid derivatives, as Fmoc amino acids with the standard side chain protecting scheme, and coupling agents were purchased from *Iris Biotech GmbH* and *NovaBioChem*. *C*-terminal amide peptides were synthesized on a 0.1 mmol scale using a 0.41 mmol/g loading H-Rink amide ChemMatrix resin from *Merck.* All other chemicals were purchased from *Fisher Scientific* and *Merck*. All solvents were synthesis grade, except for DMF, DIEA, and TFA that were peptide synthesis grade. Water was purified using a Milli-Q system (*Millipore*).

6.2.-Synthesis of peptide 7[Tb]

Peptide **7** was synthesized using standard Fmoc solid phase peptide synthesis procedures. Amino acid couplings were conducted using a 4-fold excess and HBTU (4 equiv.) as activating agent. Each amino acid was activated for 2 min in DIEA/DMF (6 equiv.) before being added onto the resin. Peptide bond-forming couplings were carried out for 30 min. Then, the Fmoc group was removed by treating the resin with a 20% 4-methylpiperidine (4-MP) solution in DMF for 15 min. After filtration, the resin was washed with DMF and CH_2Cl_2.

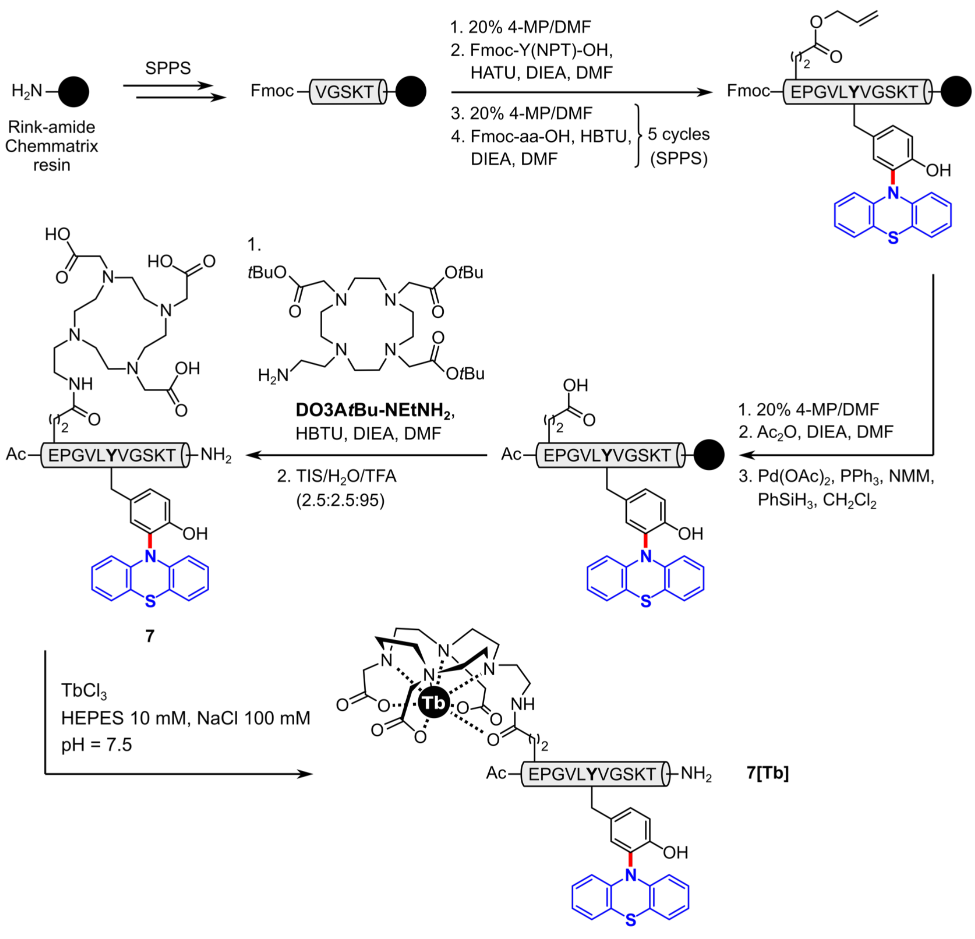

Scheme S1. Synthesis of the metallopeptide **7[Tb]**.

Y(NPT) coupling: A solution of **Fmoc-Y(NPT)-OH** (0.12 mmol, 4 equiv.) and HATU (0.12 mmol, 4 equiv.) in DFM (3 mL) was mixed with DIEA/DMF (0.195 M, 0.18 mmol, 6 equiv.) for 2 min before being added over the resin (0.03 mmol). The mixture was left stirring under N_2 (g) for 1 hour. After filtration, the resin was washed with DMF (3 × 3 mL × 3 min) and CH_2Cl_2 (3 × 3 mL × 3 min).

5 mg of the resin were resuspended in 125 µL of a TFA cleavage cocktail (2.5% H_2O, 2.5% triisopropysilane (TIS) and 95% TFA), to simultaneously cleave the peptide from the resin and remove the side-chain protecting groups, and the mixture was stirred for 1.5 h. After precipitation of the TFA filtrates in 1.2 mL cold Et_2O, the crude was dissolved in 1:1 MeCN/H_2O. The sample was analysed by reversed phase HPLC (RP-HPLC) and Electrospray Ionization Mass Spectrometry (ESI-MS) (Figure S1).

Fmoc-Y(NPT)VGSKT-NH$_2$: HPLC: t_R = 20.3 min. ESI-MS (m/z): $[MH]^+$ calculated for $C_{56}H_{65}N_9O_{11}S$: 1072.4597; found 1072.59 $[M+H]^+$, 536.76 $[M+2H]^{2+}$.

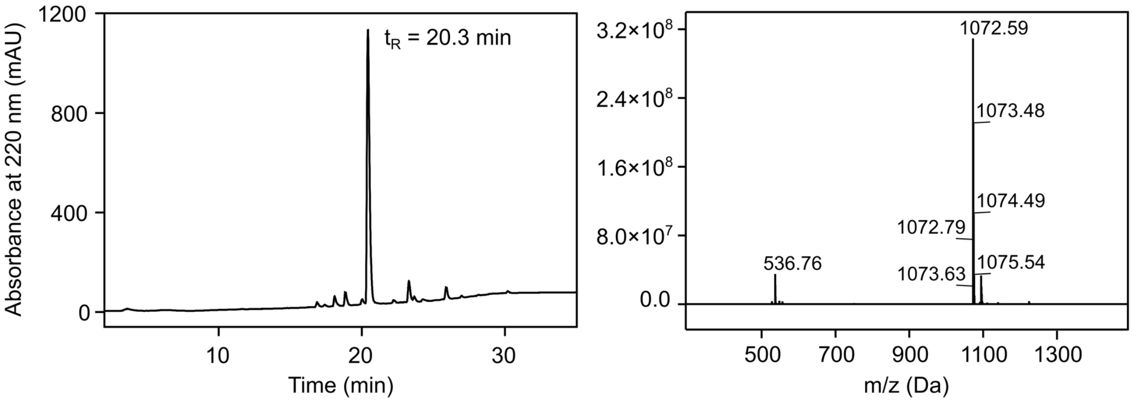

Figure S1. HPLC chromatogram at 220 nm of the reaction crude (left) and ESI-MS spectrum of the t_R = 20.3 min peak, identified as **Fmoc-Y(NPT)VGSKT-NH$_2$** (right).

Acetylation of the *N*-terminus: After peptide chain elongation, the Fmoc group was removed by treating the resin (0.03 mmol) with a 20% 4-MP solution in DMF for 15 min. The peptide was then acetylated by the treatment of the resin with a mixture of Ac_2O (12 equiv.) and DIEA/DMF (0.195 M, 10 equiv.) for 45 min. After filtration, the resin was washed with DMF (3 × 3 mL × 3 min) and CH_2Cl_2 (3 × 3 mL × 3 min).

Deprotection of the orthogonally protected Glu(Alloc) side chain: The side chain of the *C*-terminal Glu(Alloc) residue was selectively deprotected by treating the resin (0.03 mmol) with a mixture of $Pd(OAc)_2$ (0.3 equiv.), PPh_3 (1.5 equiv.), N-methylmorpholine (NMM, 10 equiv.), and $PhSiH_3$ (10 equiv.) in CH_2Cl_2 (2.5 mL) overnight at RT. The mixture was filtered and the resin was washed with DMF (3 × 3 mL × 3 min), *N,N*-diethyldithiocarbamate (DEDTC) (25 mg in 5 mL of DMF, 10 min), and CH_2Cl_2 (3 × 3 mL × 3 min).

DO3A*t*Bu-NEtNH$_2$ coupling: DO3A*t*Bu-NEtNH$_2$ was synthesized following published procedures.[17] A solution of HBTU (1 equiv.) and DIEA (4 equiv.) in DMF (2.5 mL) was added to the resin (0.03 mmol) and the suspension was shaken for 2 min before the addition of DO3A*t*Bu-NEtNH$_2$ (2 equiv.) over the mixture. The resulting mixture was left stirring under N_2 for 2 hours. After filtration, the resin was washed with DMF (3 × 3 mL × 3 min) and CH_2Cl_2 (3 × 3 mL × 3 min).

17 Sánchez-Fernández, R.; Sánchez-Temprano, A.; Esteban-Gómez, D.; Pazos, E. *ChemBioChem* **2023**, e202300072.

Cleavage and deprotection of semipermanent protecting groups: 3 mL of the TFA cleavage cocktail were added to the resin-bound peptides (0.03 mmol) and the mixture was left stirring for 5.5 h. After precipitation of the TFA filtrates in cold Et_2O, the peptide was dissolved in 1:1 MeCN/H_2O. The purification of the peptide was performed in an *Agilent* 1200 series using an Aeris semipreparative column (peptide XB-C18 stationary phase, 5 µm, 100 Å pore size, 250 × 10 mm), from *Phenomenex* using the method 30 → 60% MeCN, 0.1% TFA / H_2O, 0.1% TFA over 22 min.

Analytical RP-HPLC was carried out using an *Agilent* 1200 series connected to a photo-diode array (PDA) detector. RP-HPLC and ESI-MS analyses were performed using a Liquid Chromatograph Mass Spectrometer system, *Bruker* Elute UHPLC connected to a mass spectrometer *Bruker* amaZon speed Toxtyper or an *Agilent* 1290 Infinity II LC system coupled to an *Agilent* 6546 quadrupole time of flight (Q-TOF) mass spectrometer. An Aeris analytical column from *Phenomenex* (peptide XB-C18 stationary phase, 3.6 µm, 100 Å pore size, 150 × 2.1 mm) was used for all the analyses. The standard method used for analytical HPLC was 5 → 95% MeCN, 0.04% TFA / H_2O, 0.04% TFA over 23 min. HPLC-MS quality solvents were used to prepare the eluents.

7: HPLC: t_R = 16.0 min. ESI-MS (m/z): $[MH]^+$ calculated for $C_{82}H_{123}N_{19}O_{22}S$: 1758.8884; found 1758.8865 $[M+H]^+$, 879.9476 $[M+2H]^{2+}$, 587.3016 $[M+3H]^{3+}$.

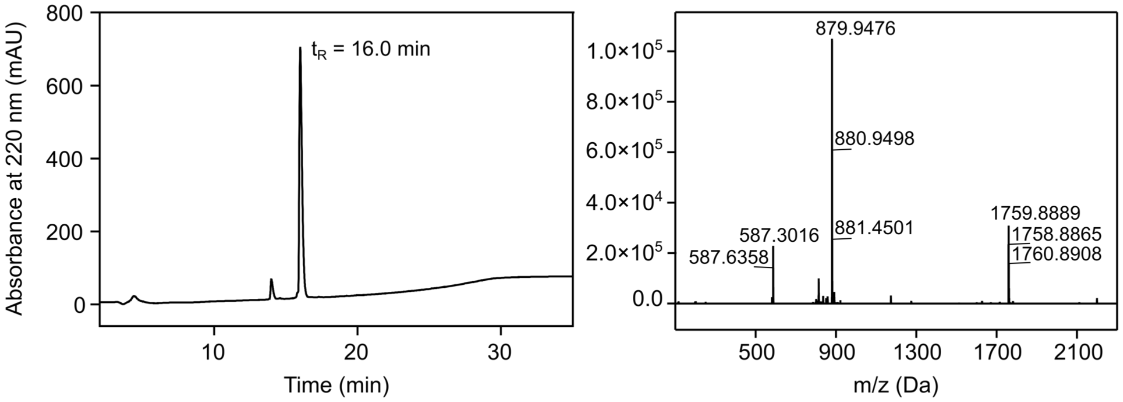

Figure S2. HPLC chromatogram at 220 nm (left) and MS spectrum of the t_R = 16.0 min peak, identified as **7** (right).

Lanthanide chelation: To a 50 µM **7** solution in HEPES buffer (10 mM HEPES, 100 mM NaCl, pH 7.5) 2 µL of a solution of $TbCl_3$ in 1 mM HCl were added (final concentration 50 µM, 1 equiv.), the mixture was left overnight at RT and analysed by RP-HPLC-MS.

7[Tb]: ESI-MS (m/z): $[MH]^+$ calculated for $C_{82}H_{120}N_{19}O_{22}STb$: 1914.7903; found 1914.7903 $[M+H]^+$, 957.8999 $[M+2H]^{2+}$, 638.9357 $[M+3H]^{3+}$.

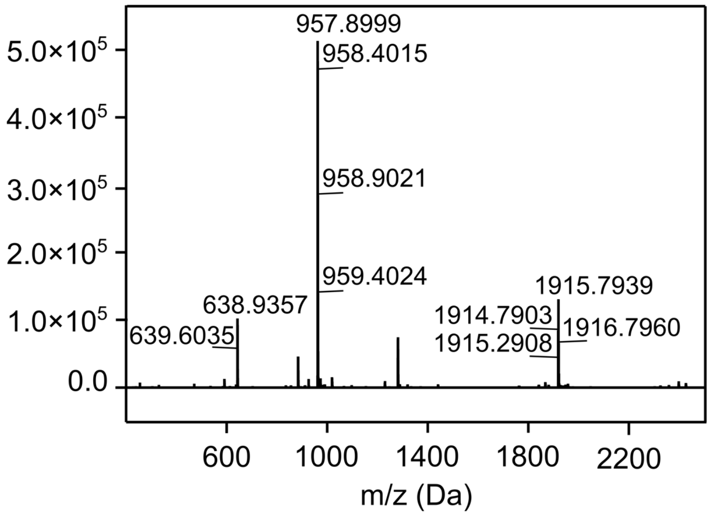

Figure S3. ESI-MS spectra of **7[Tb]**.

6.3.-Spectroscopic characterization

Concentration calculation: The concentration of the peptide **7** was determined using the extinction coefficient of **H-Y(NPT)-OH**, 43527 $M^{-1}cm^{-1}$, determined by UV spectroscopy in 0.1 M phosphate buffer, pH 7.4, at 254 nm.

UV-vis spectra of peptides 7 and 8 and complexes 7[Tb] and 8[Tb]: The UV-vis spectra of both peptide **7** and its analogue **8**, which contains an unmodified Tyr residue instead of the Tyr-NPT conjugate, and their corresponding Tb(III) complexes were recorded from 200 to 450 nm on a *Jasco* V-750 spectrometer, using a standard 10 mm light pass *Hellma* Semi-Micro cuvette (114-10-40) at 25 ºC with the following settings: UV/Vis bandwidth 5.0 nm, UV/Vis response 0.06 s; data interval 1.0 nm; scan mode continuous, scan speed 200 nm/min.

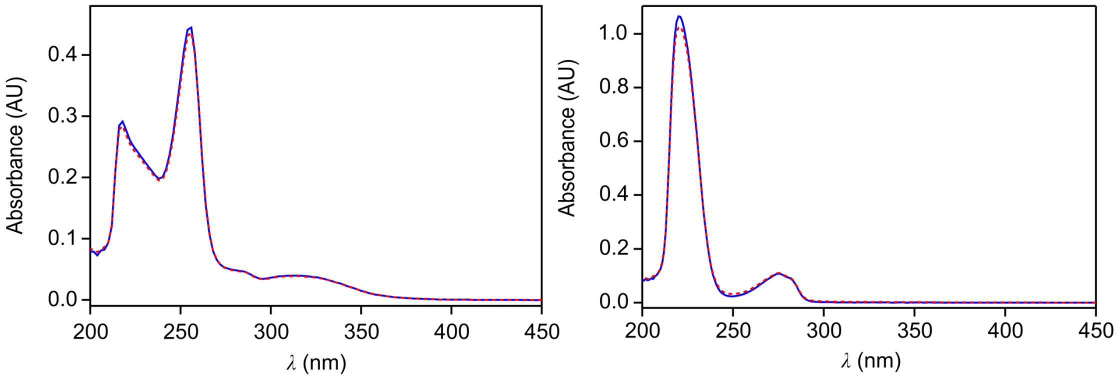

Figure S4. Left. UV-vis spectra of a 10 µM **7** solution (blue solid line) and a 10 µM **7[Tb]** solution (red dashed line). **Right.** UV-vis spectra of a 100 µM **8** solution (blue solid line) and a 100 µM **8[Tb]** solution (red dashed line).

Fluorescence spectra of peptides 7 and 8: Steady-state emission measurements were made with a *Jobin-Yvon Fluoromax-3*. All measurements were made with a *Hellma* Semi-Micro cuvette (114F-10-40, 10 mm light path) at RT, using the following settings: excitation wavelength 254 nm for **7** and 274 nm for **8**; excitation slit width 5.0 nm; emission slit width 10.0 nm; increment 1.0 nm; integration time 0.10 s. The emission spectrum of **7** was recorded from 335 to 600 nm with a 331 nm long-pass filter to avoid interference from harmonic doubling, and the spectrum of **8** was recorded from 290 to 530 nm.

Time-gated emission spectra of 7[Tb] and 8[Tb]: Time-gated emission measurements were made with a FluoroMax Plus-P spectrofluorometer from *Horiba Scientific* equipped with an R928P photon counting emission detector, in the phosphorescence mode using a xenon flash lamp. All measurements were made with a *Hellma* Semi-Micro cuvette (114F-10-40, 10 mm light path) at 25 ºC, using the following settings: excitation wavelength 254 nm; excitation slit width 10.0 nm; emission slit width 3.0 nm; increment 2.0 nm; time between flashes 0.061 s; initial delay 0.2 ms; sample window 0.02 s; flash count 0.01 s; HV detector voltage 950 V. The emission spectrum was recorded from 450 to 650 nm.

7- Control Experiments and Mechanism Proposal

Table S3. Influence of Radical Traps[a]

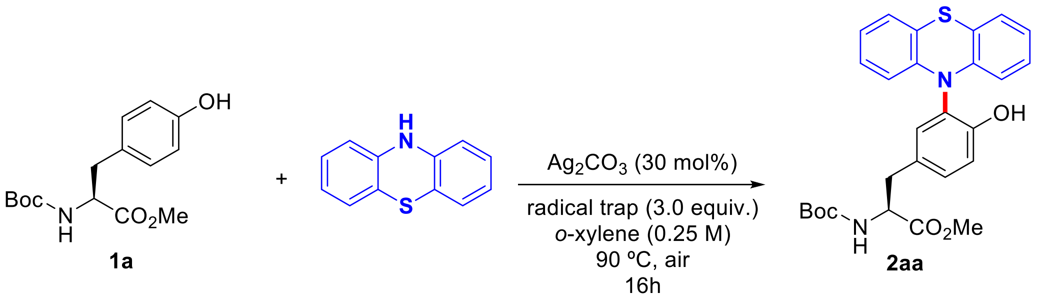

Entry	Radical trap	Yield of 2aa[a]
1	none	99
2	BHT	0
3	TEMPO	68

[a] Reaction conditions: **1a** (0.30 mmol), phenothiazine (0.25 mmol), radical trap (3.0 equiv), Ag_2CO_3 (30 mol %), *o*-xylene (1.0 mL) at 90 ºC for 16 h under air. [b] Yield of isolated product after column chromatography.

Table S4. Influence of the Tyr Compound[a]

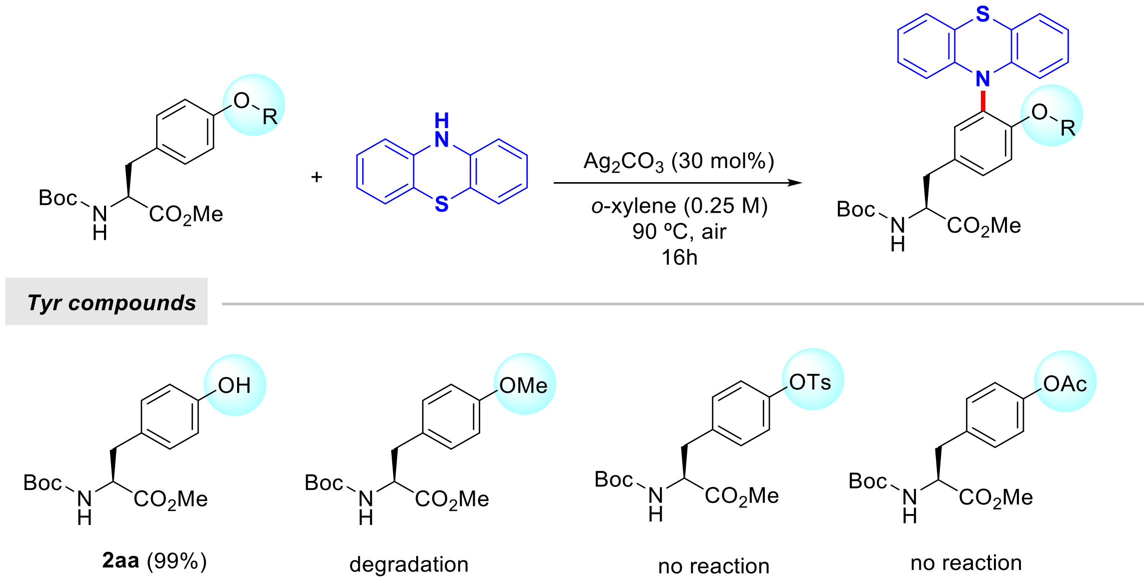

[a] Reaction conditions: Tyrosine compound (0.30 mmol), phenothiazine (0.25 mmol), Ag_2CO_3 (30 mol%), *o*-xylene (1.0 mL) at 90 ºC for 16 h under air.

Scheme S2. Mechanism Proposal

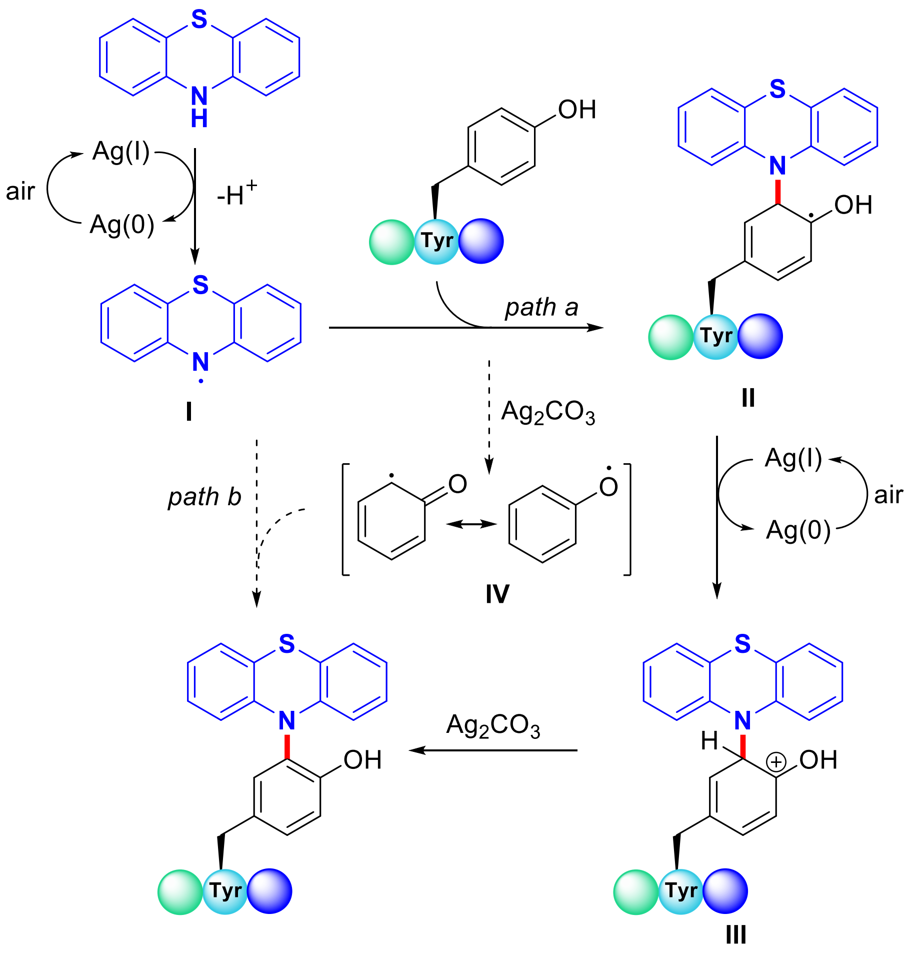

8.-X-Ray Crystallography

The compounds were prepared upon crystallization in a mixture of EtOAc and hexanes. Intensity data were collected on an Agilent Technologies Super-Nova diffractometer, which was equipped with monochromated Cu k_{α} radiation (λ= 1.54184 Å) and Atlas CCD detector. Measurement was carried out at 150.00(10) K with the help of an Oxford Cryostream 700 PLUS temperature device. Data frames were processed (united cell determination, analytical absorption correction with face indexing, intensity data integration and correction for Lorentz and polarization effects) using the Crysalis software package. The structure was solved using SHELXT and refined by full-matrix least-squares with SHELXL-97. Final geometrical calculations were carried out with Mercury and PLATON as integrated in WinGX. Analysis of the absolute structure using likelihood methods (Hooft, Straver & Spek, 2008) was performed using PLATON (Spek, 2010). The results indicated that the absolute structure had been correctly assigned. The method calculated that the probability that the structure is inverted is smaller than 10^{-5}. The absolute structure parameter y (Hooft, Straver & Spek, 2008) was calculated using PLATON (Spek, 2010). The resulting value was y=-0.04(12), which together with Flack parameter value, indicate that the absolute structure has probably been determined correctly.

8.1.-Compound 2aa

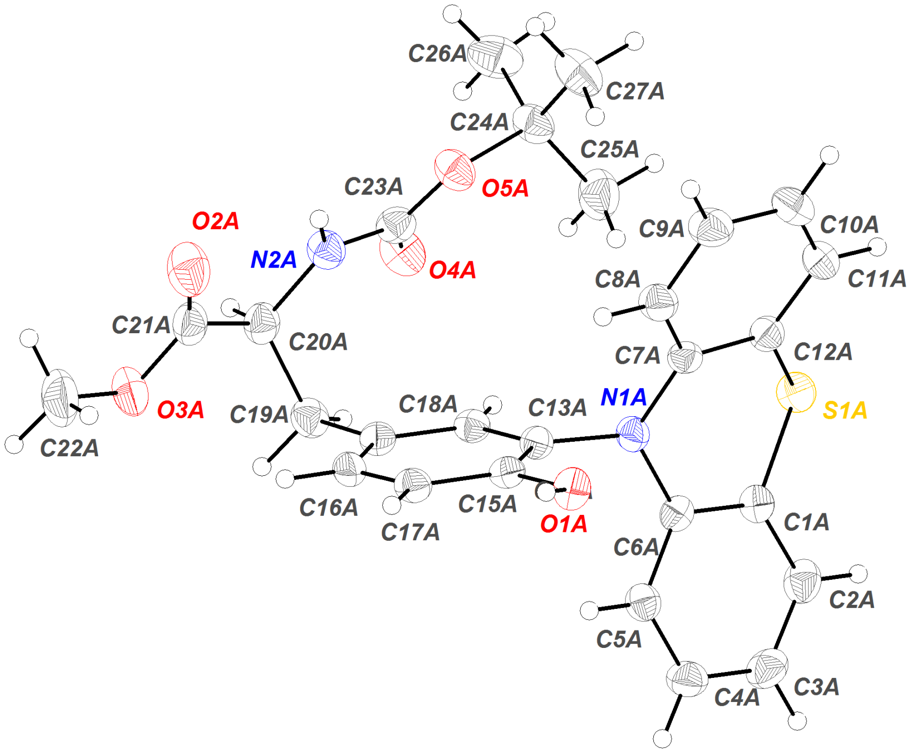

View of the molecular structure of ***2aa*** *(asymmetric unit), with 50% probability displacement ellipsoids. See CIF file attached* (**CCDC 2257500**)

Empirical formula	$C_{27}H_{28}N_2O_5S$
Formula weight	492.57
Temperature/K	170.00(10)
Crystal system	monoclinic
Space group	$P2_1$
a/Å	8.95486(6)
b/Å	14.53560(11)
c/Å	19.34904(16)
α/°	90.0
β/°	91.3948(7)
γ/°	90.0
Volume/Å3	2517.81(3)
Z	4
ρcalcg/cm^3	1.299
μ/mm^{-1}	1.475

F(000)	1040.0
Crystal size/mm^3	0.41 × 0.28 × 0.22
Radiation	CuKα (λ = 1.54184)
2Θ range for data collection/°	4.568 to 137.93
Index ranges	$-10 \le h \le 10$, $-17 \le k \le 17$, $-22 \le l \le 23$
Reflections collected	47005
Independent reflections	9354 [R_{int} = 0.0349, R_{sigma} = 0.0236]
Data/restraints/parameters	9354/1/641
Goodness-of-fit on F2	1.024
Final R indexes [I>=2σ (I)]	R_1 = 0.0310, wR_2 = 0.0810
Final R indexes [all data]	R_1 = 0.0315, wR_2 = 0.0815
Largest diff. peak/hole / e $Å^{-3}$	0.18/-0.28
Flack parameter	-0.002(5)

8.2.-Compound 6c

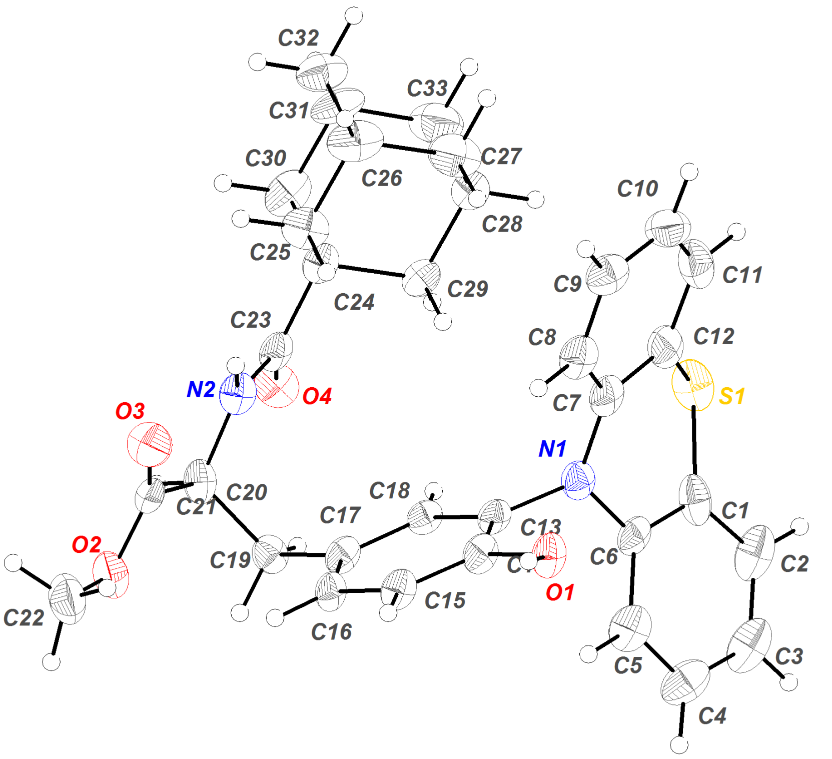

View of the molecular structure of ***6c*** *(asymmetric unit), with 50% probability displacement ellipsoids. See CIF file attached* **(CCDC 2257502)**

Empirical formula	$C_{33}H_{34}N_2O_4S$
Formula weight	554.68
Temperature/K	170.01(10)
Crystal system	monoclinic
Space group	$P2_1$
a/Å	9.4203(5)
b/Å	15.6707(9)
c/Å	10.5353(6)
α/°	90.0
β/°	115.343(7)
γ/°	90.0
Volume/Å3	1405.59(14)
Z	2
ρcalcg/cm^3	1.311
μ/mm^{-1}	1.355

F(000)	588.0
Crystal size/mm^3	0.31 × 0.14 × 0.09
Radiation	CuKα (λ = 1.54184)
2Θ range for data collection/°	9.288 to 137.956
Index ranges	-11 ≤ h ≤ 11, -18 ≤ k ≤ 18, -12 ≤ l ≤ 10
Reflections collected	14275
Independent reflections	5160 [R_{int} = 0.0569, R_{sigma} = 0.0558]
Data/restraints/parameters	5160/1/363
Goodness-of-fit on F2	1.147
Final R indexes [I>=2σ (I)]	R_1 = 0.0889, wR_2 = 0.02254
Final R indexes [all data]	R_1 = 0.0957, wR_2 = 0.2307
Largest diff. peak/hole / e Å^{-3}	0.56/-0.42
Flack parameter	0.02(3)

9.-^{1}H NMR, ^{13}C NMR and ^{19}F NMR Spectra

^{1}H NMR (400 MHz, CDCl$_3$)

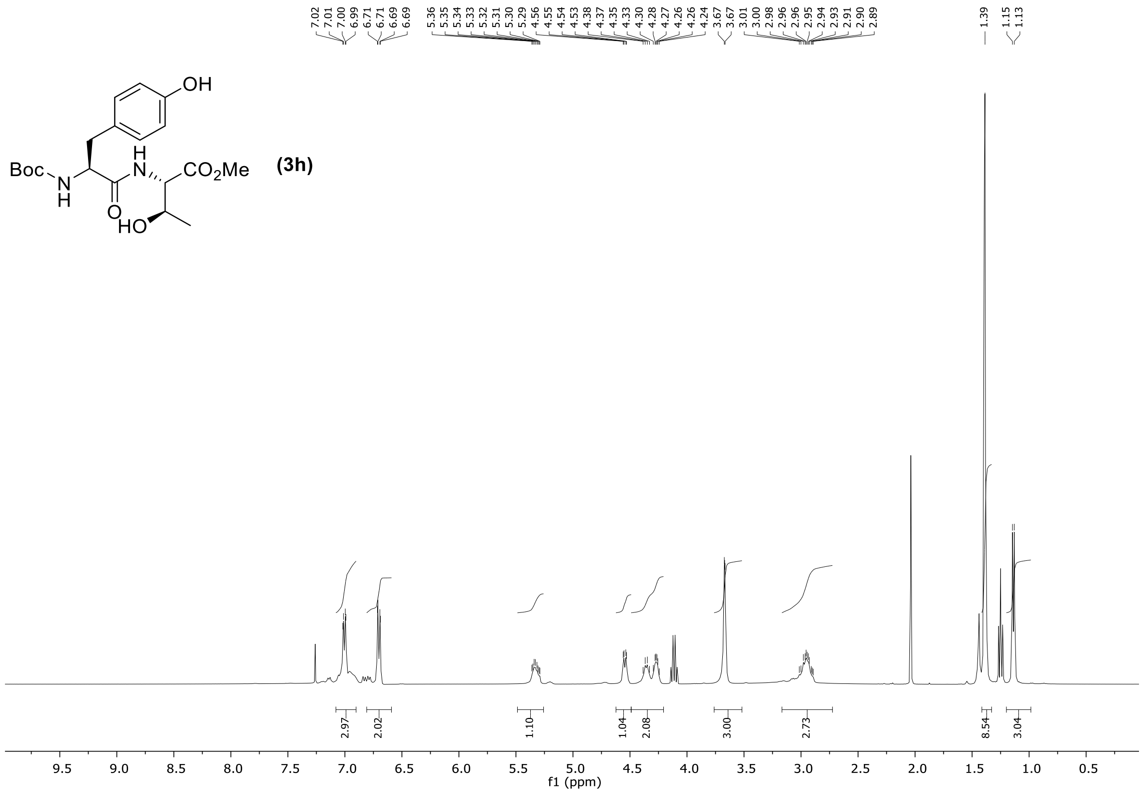

(3h)

^{13}C NMR (101 MHz, CDCl$_3$)

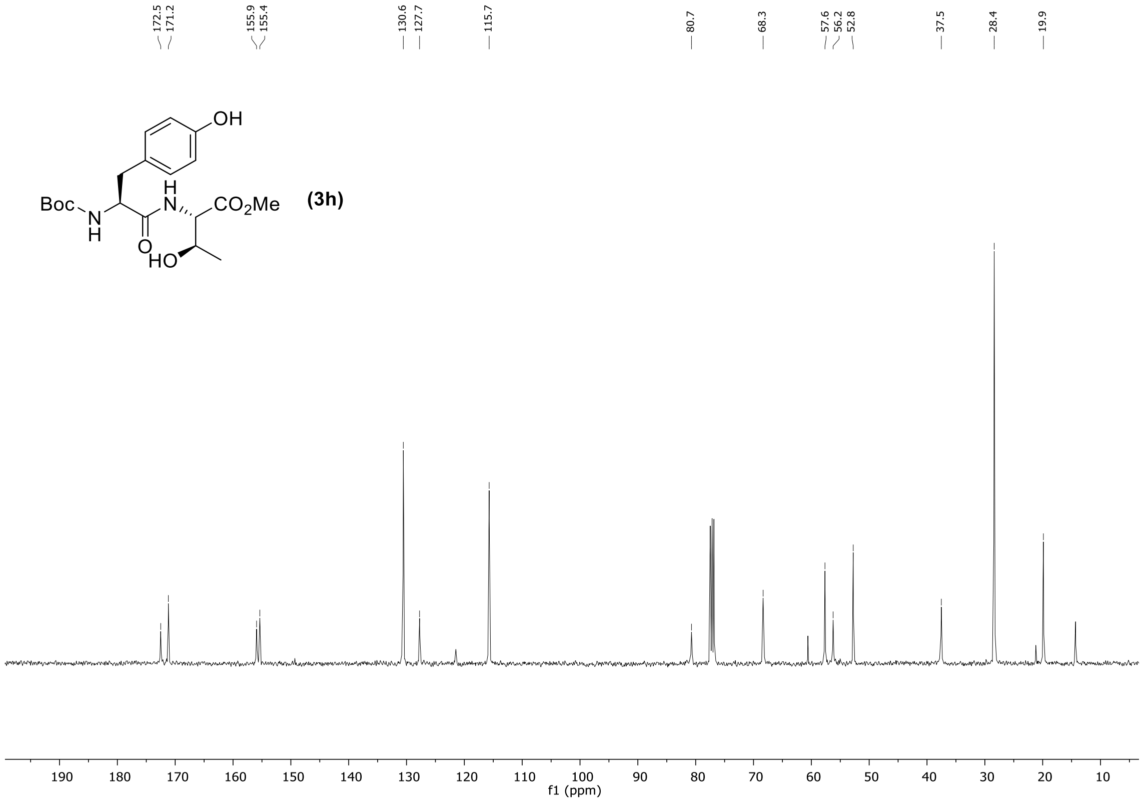

(3h)

^{1}H NMR (400 MHz, $CDCl_3$)

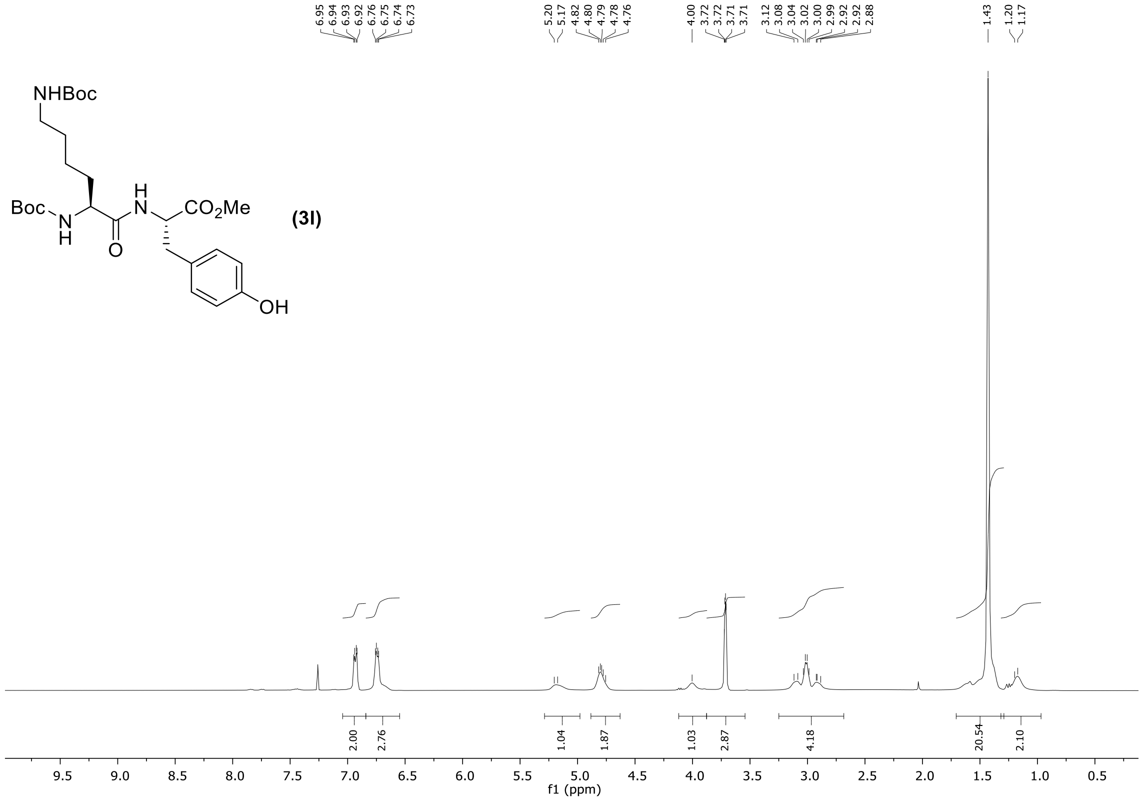

(3l)

^{13}C NMR (101 MHz, $CDCl_3$)

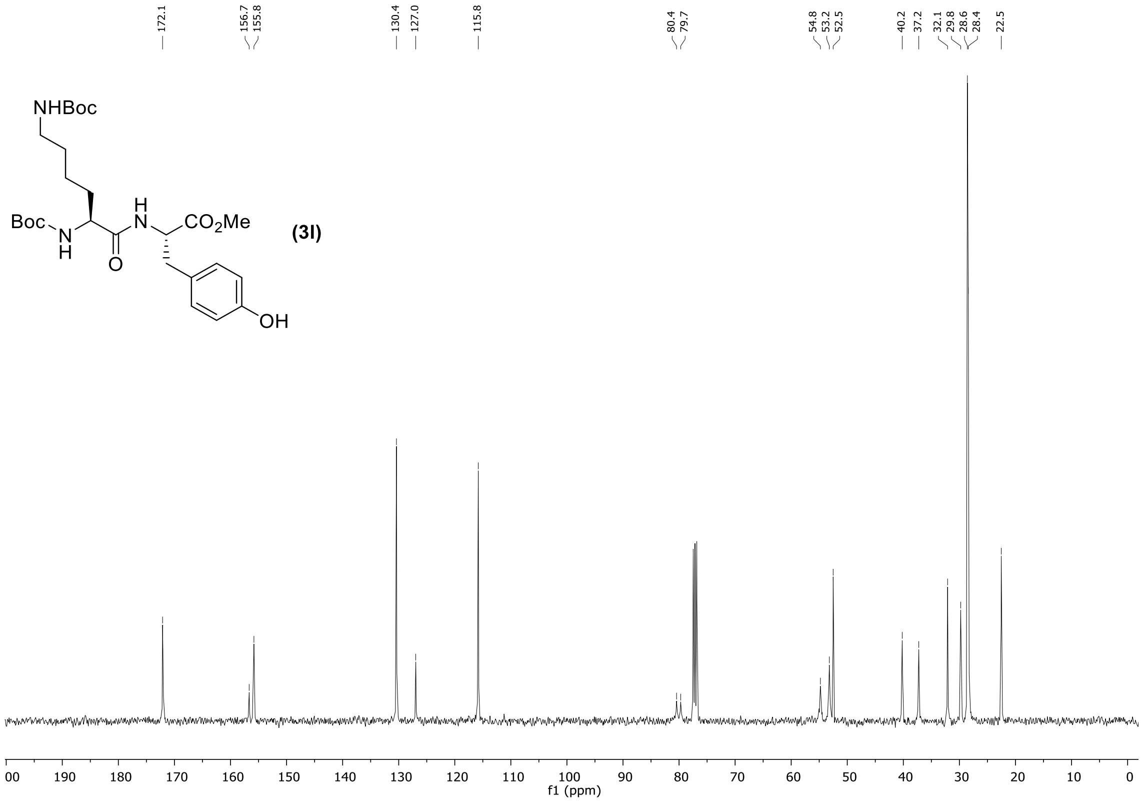

(3l)

^{1}H NMR (400 MHz, $CDCl_3$)

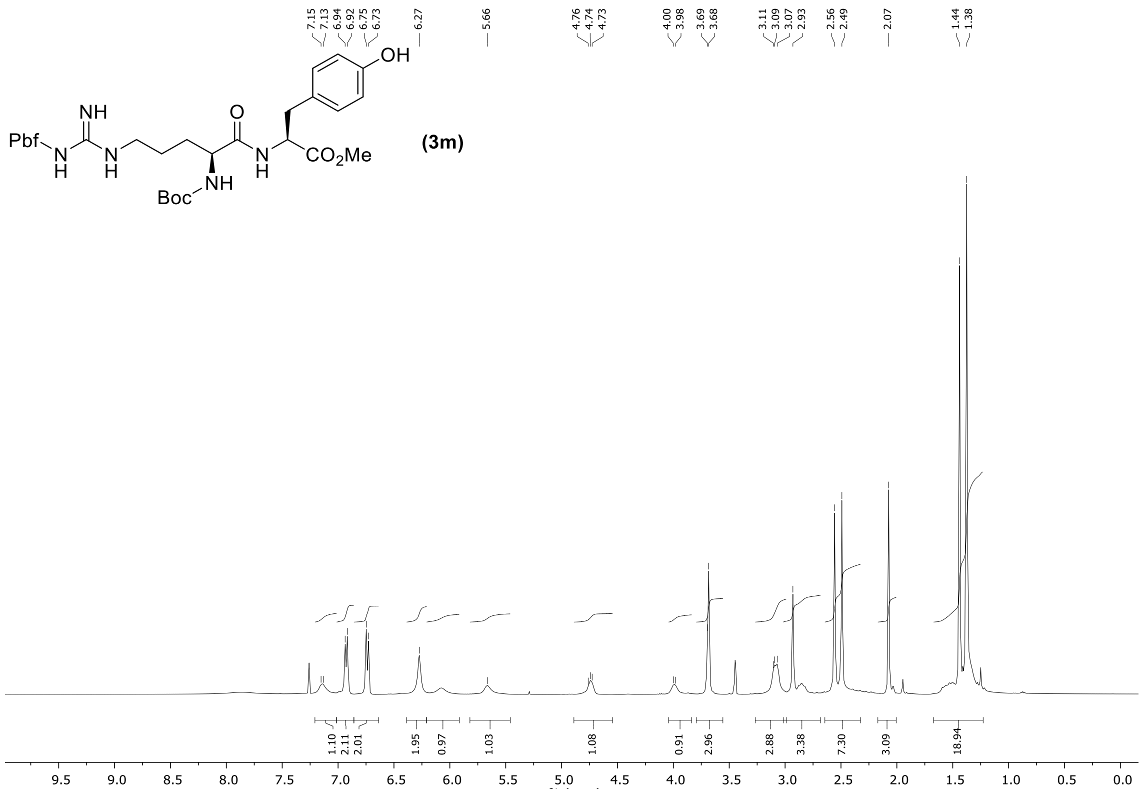

^{13}C NMR (101 MHz, $CDCl_3$)

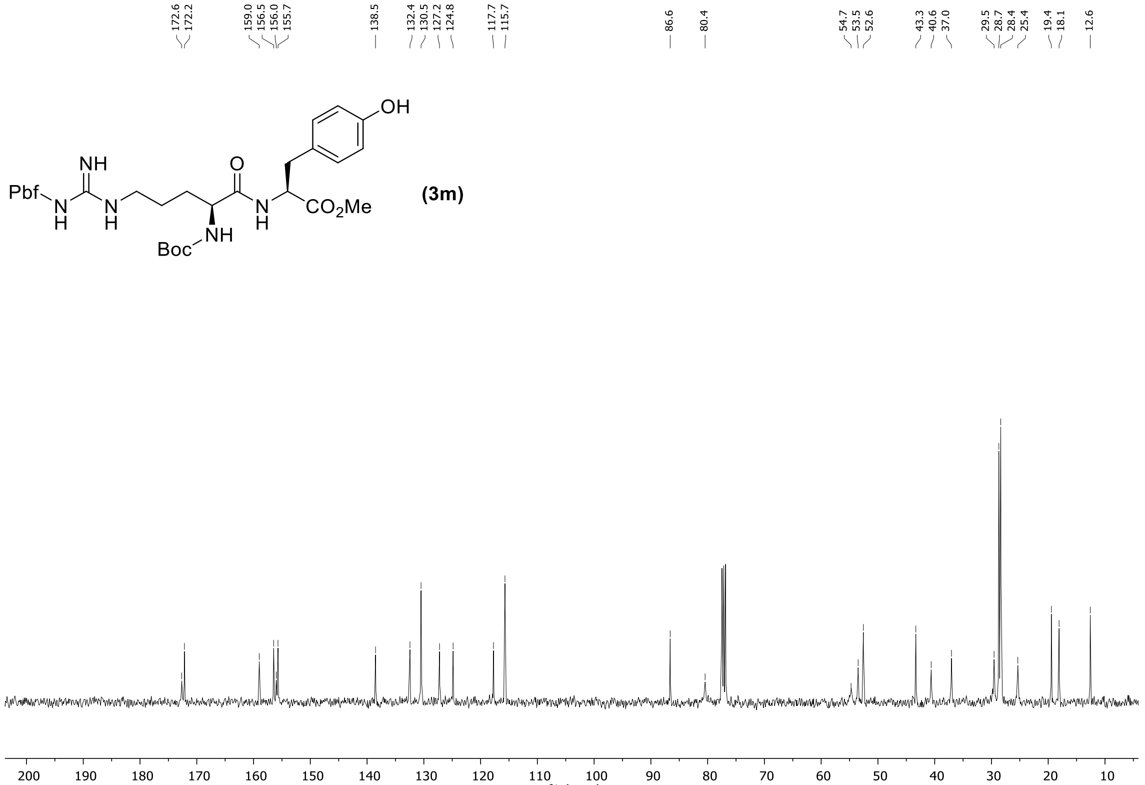

^{1}H NMR (400 MHz, $CDCl_3$)

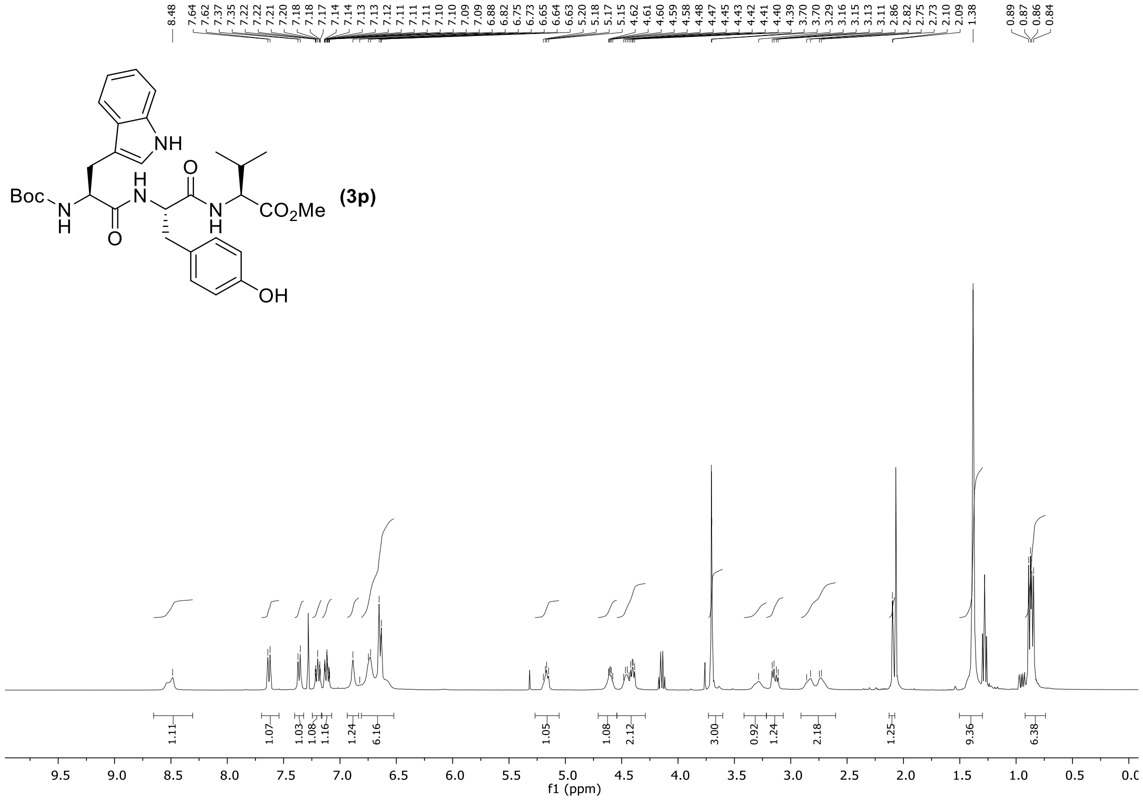

^{13}C NMR (101 MHz, $CDCl_3$)

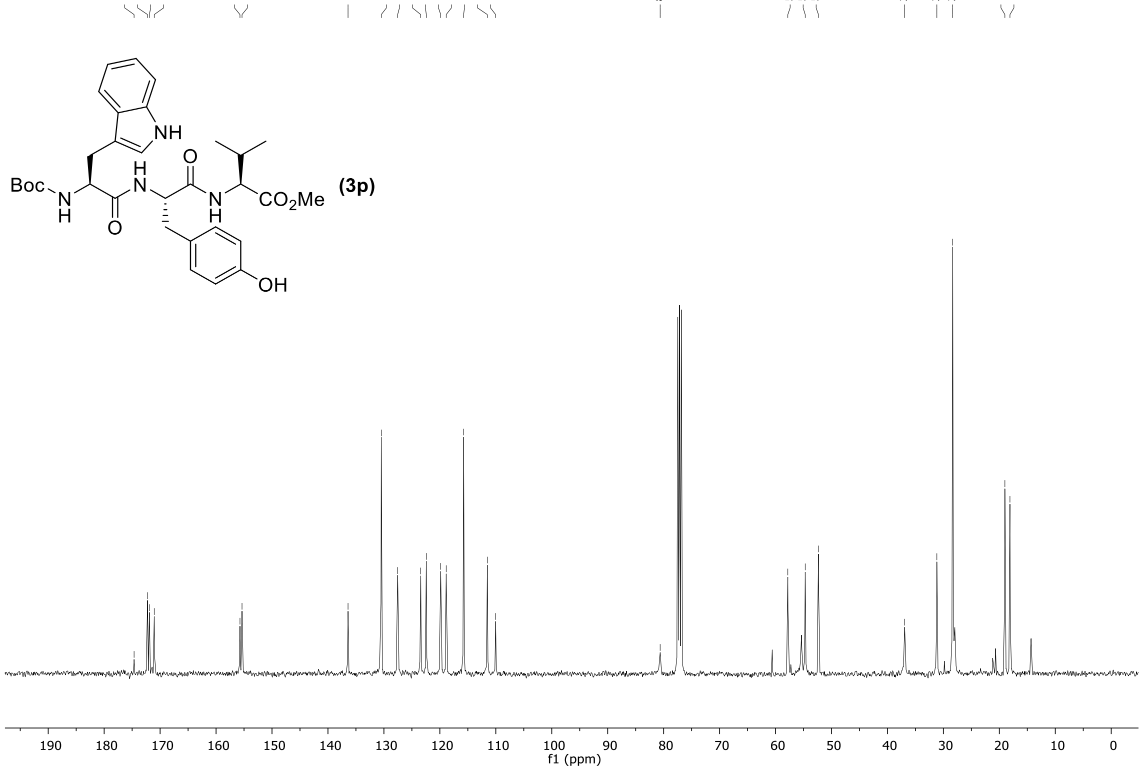

^{1}H NMR (400 MHz, $CDCl_3$)

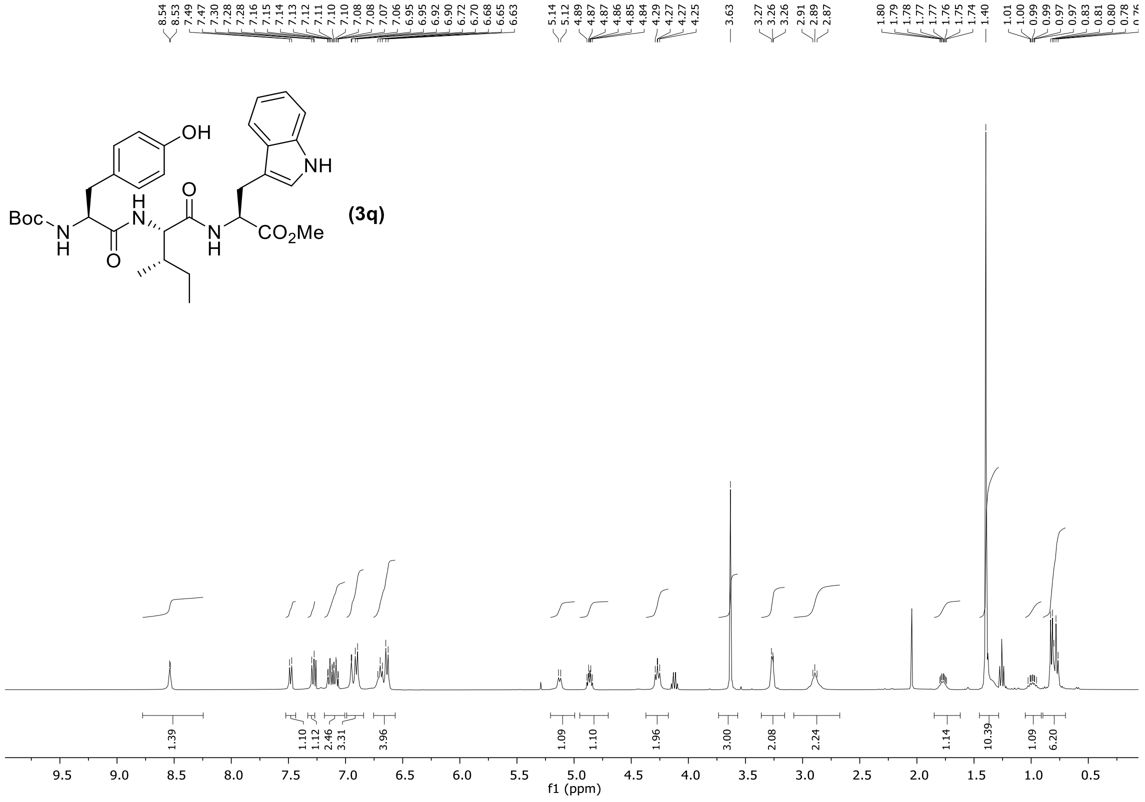

^{13}C NMR (101 MHz, $CDCl_3$)

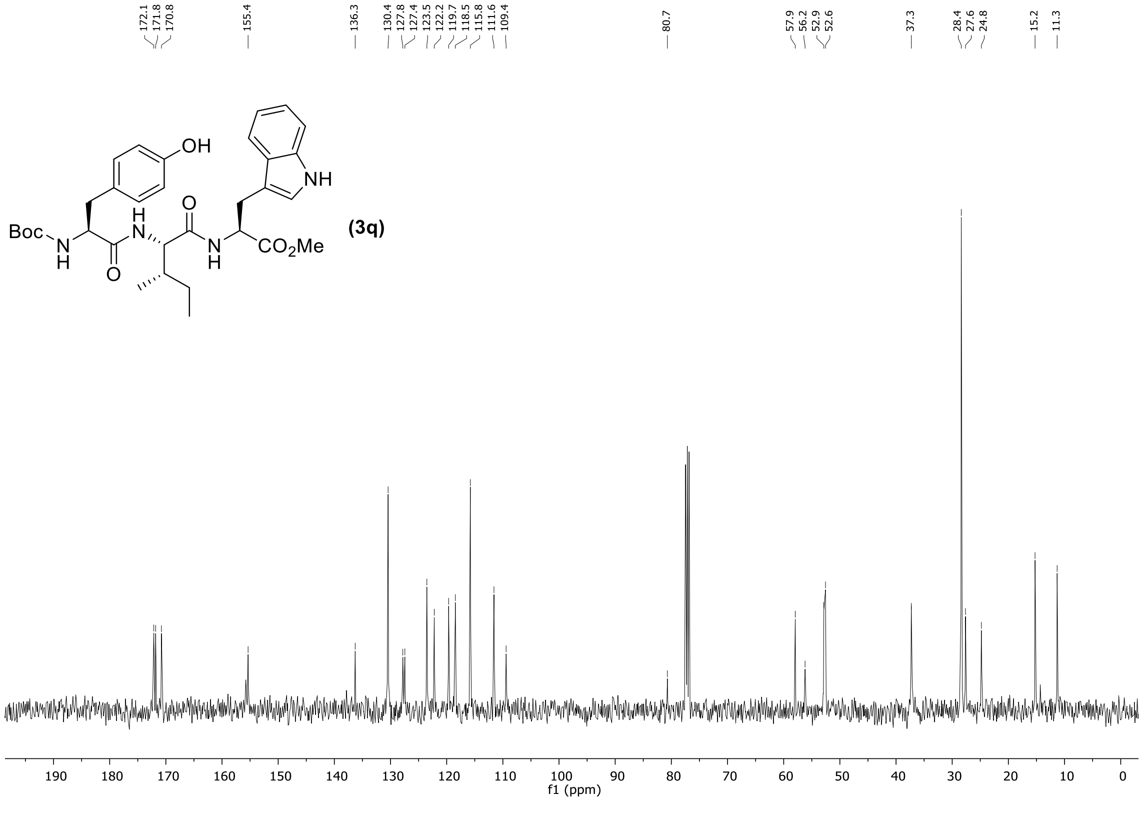

^{1}H NMR (400 MHz, $CDCl_3$)

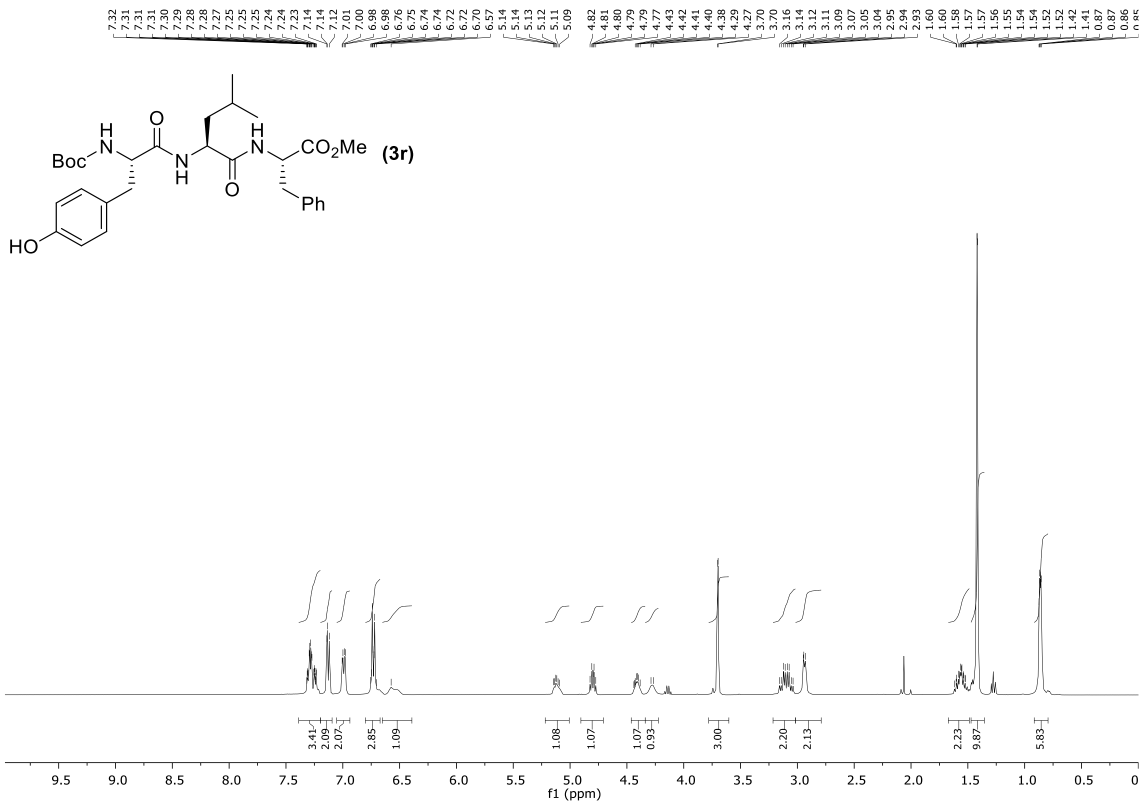

^{13}C NMR (101 MHz, $CDCl_3$)

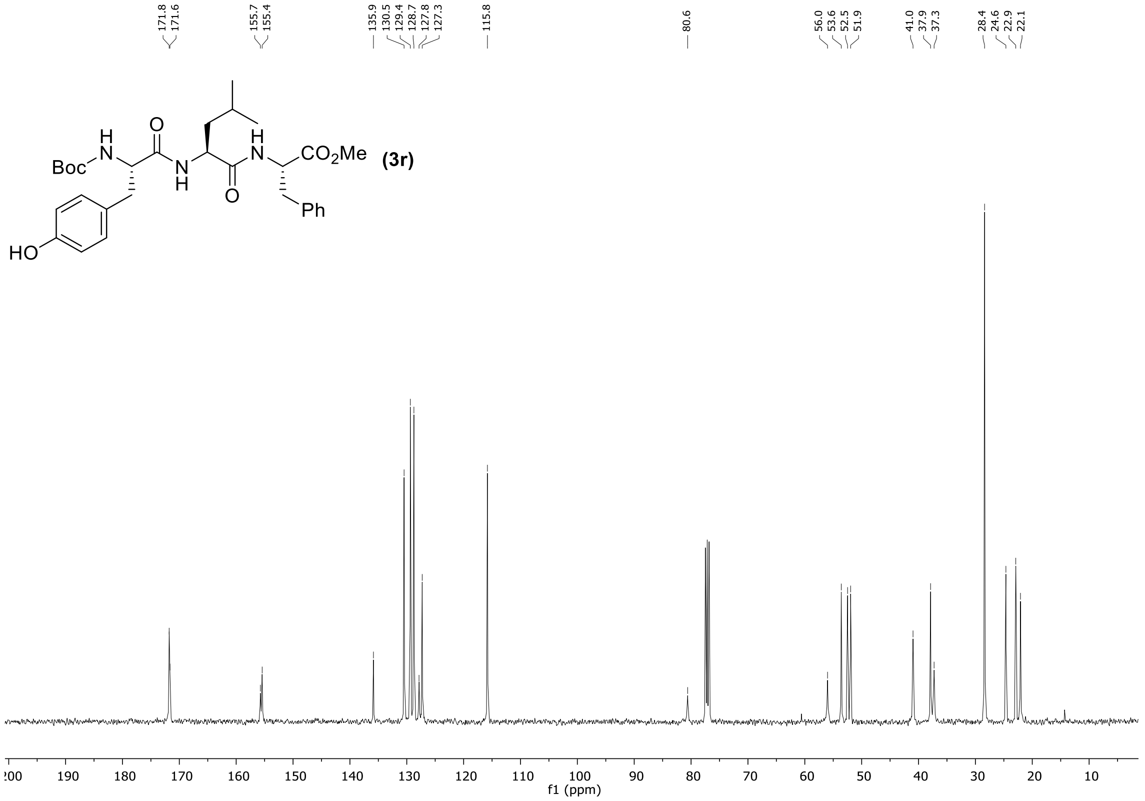

^{1}H NMR (400 MHz, $CDCl_3$)

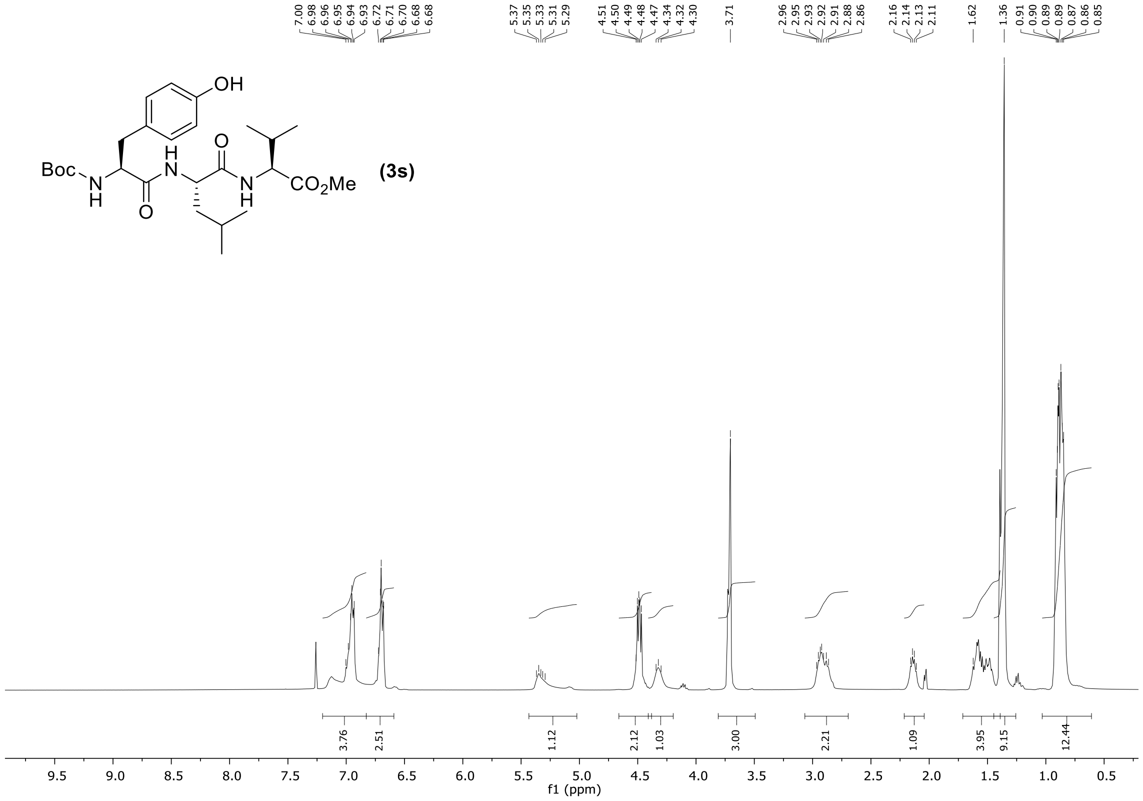

^{13}C NMR (101 MHz, $CDCl_3$)

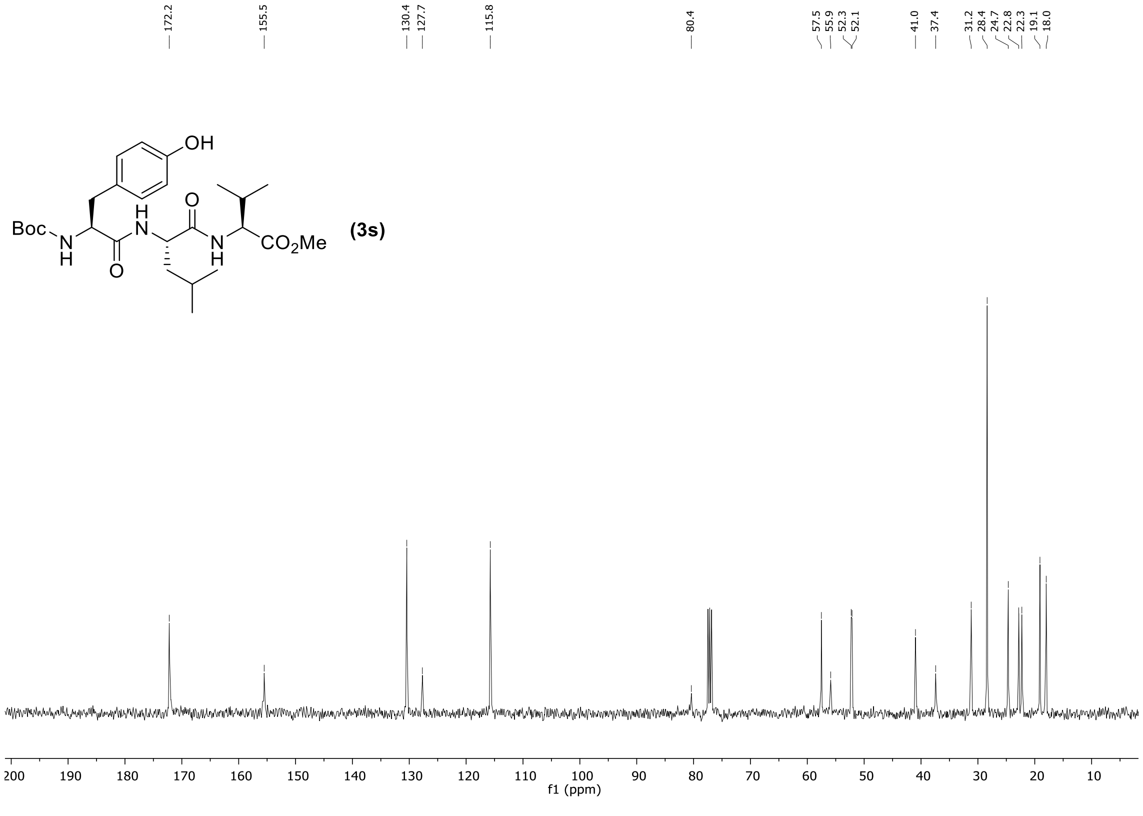

^{1}H NMR (400 MHz, $CDCl_3$)

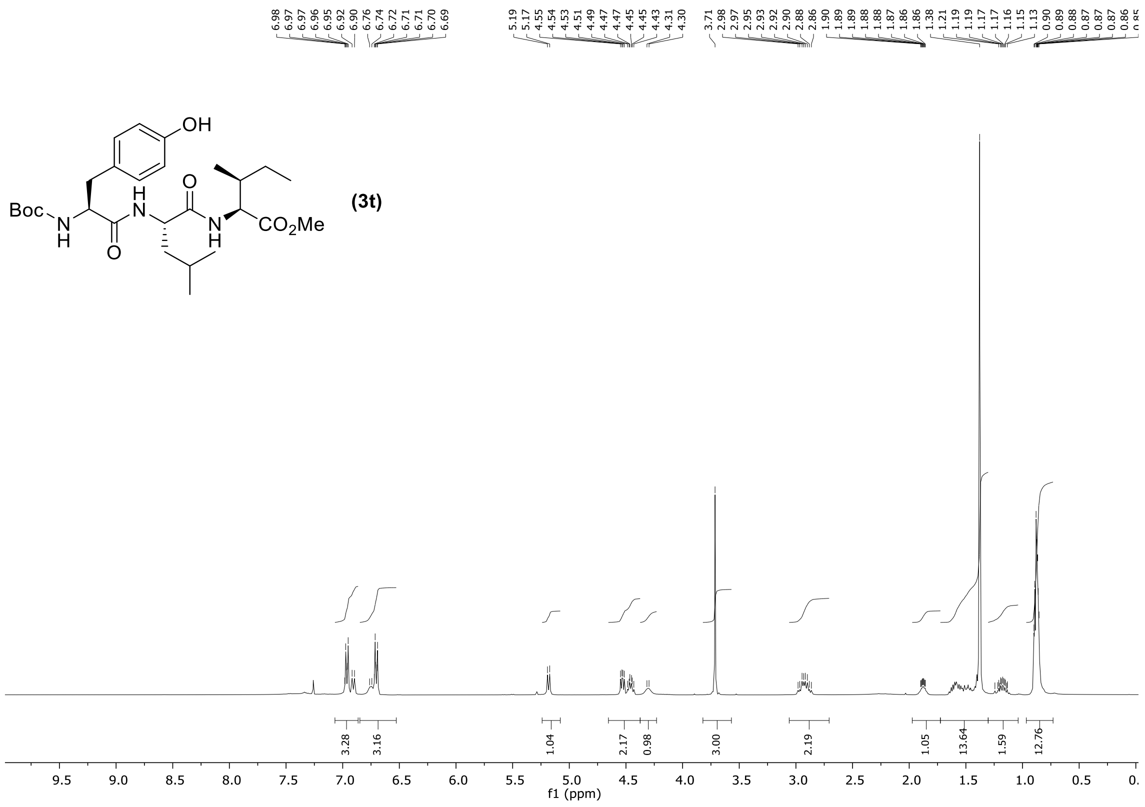

^{13}C NMR (101 MHz, $CDCl_3$)

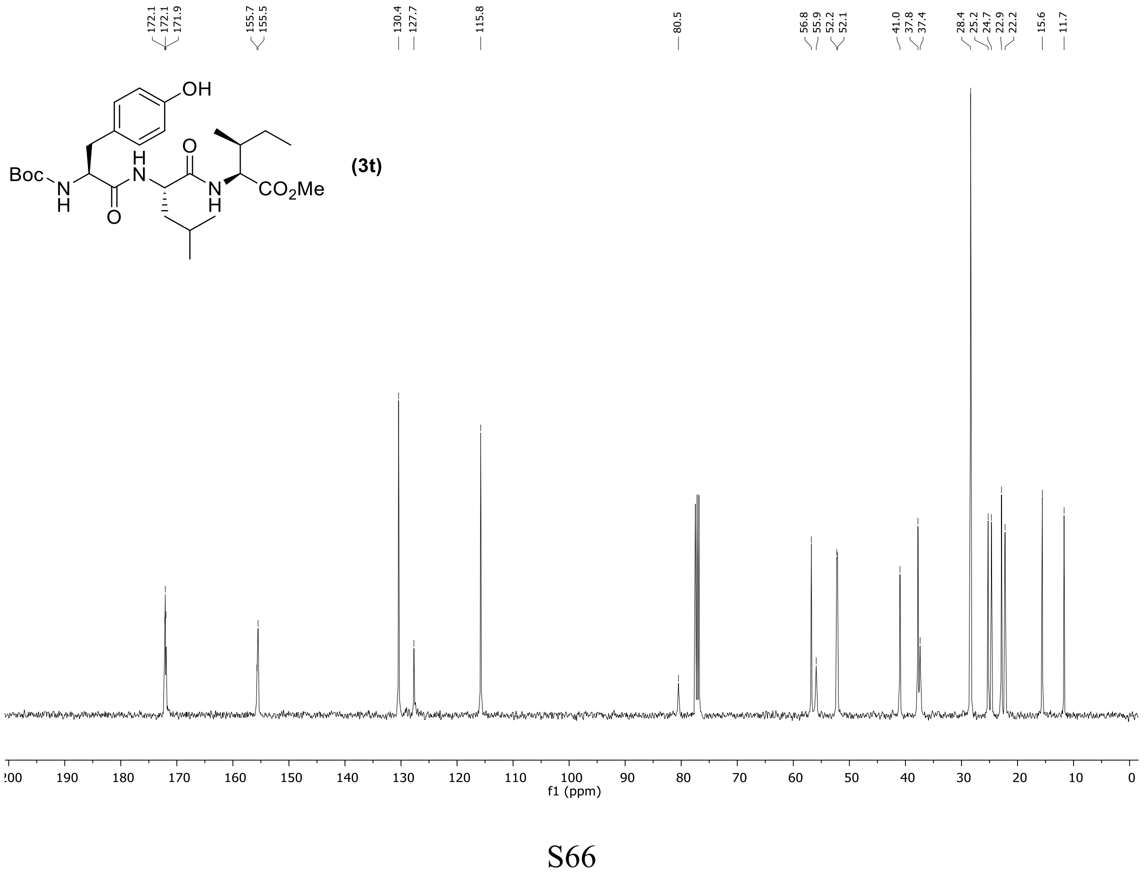

^{1}H NMR (500 MHz, DMSO-d_6 at 80 ºC)

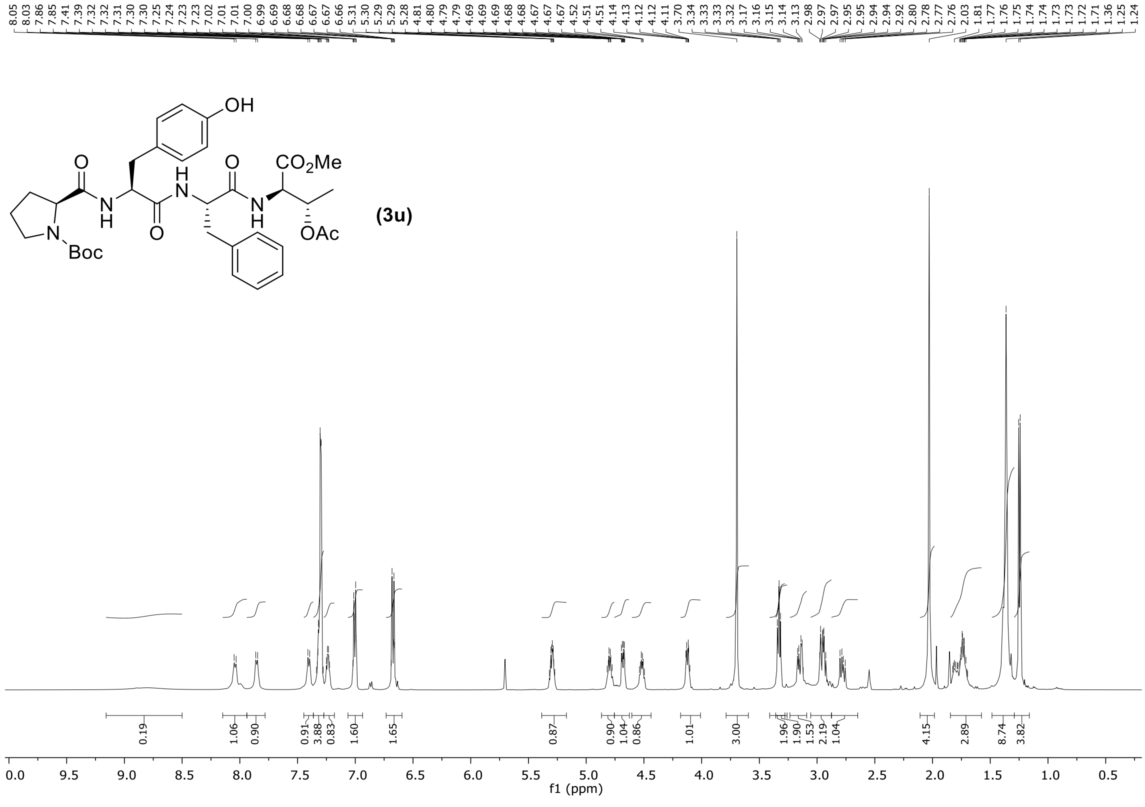

^{13}C NMR (126 MHz, DMSO-d_6 at 80 ºC)

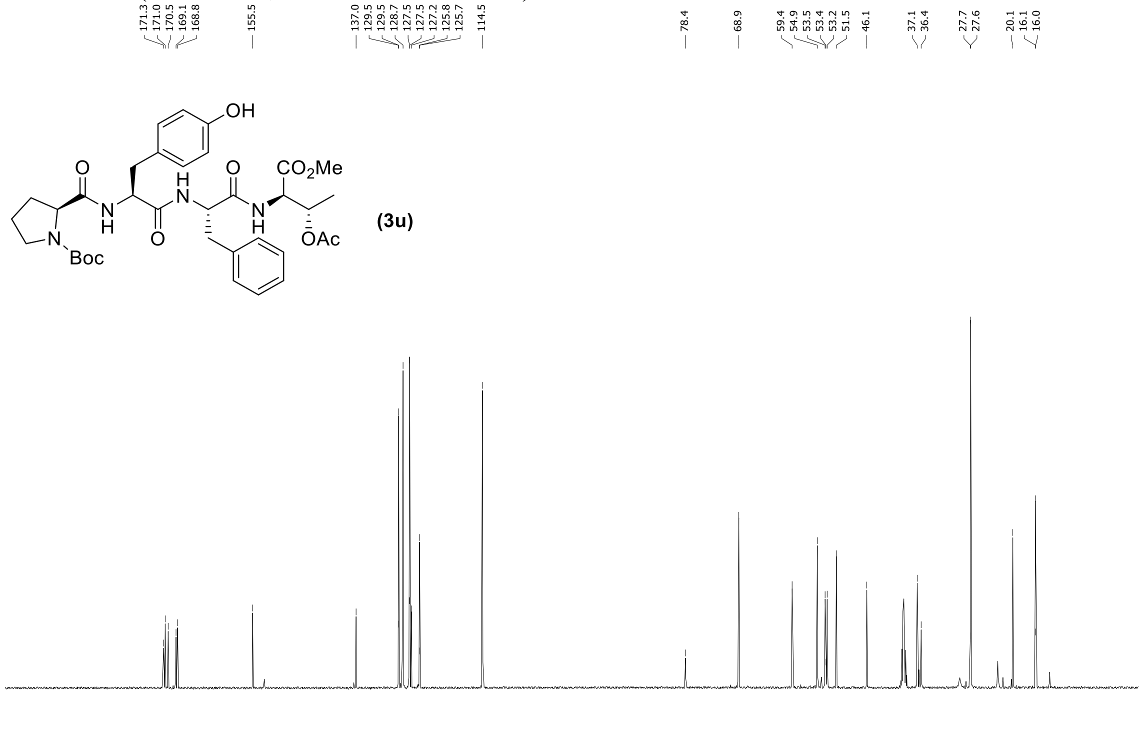

^{1}H NMR (400 MHz, $CDCl_3$)

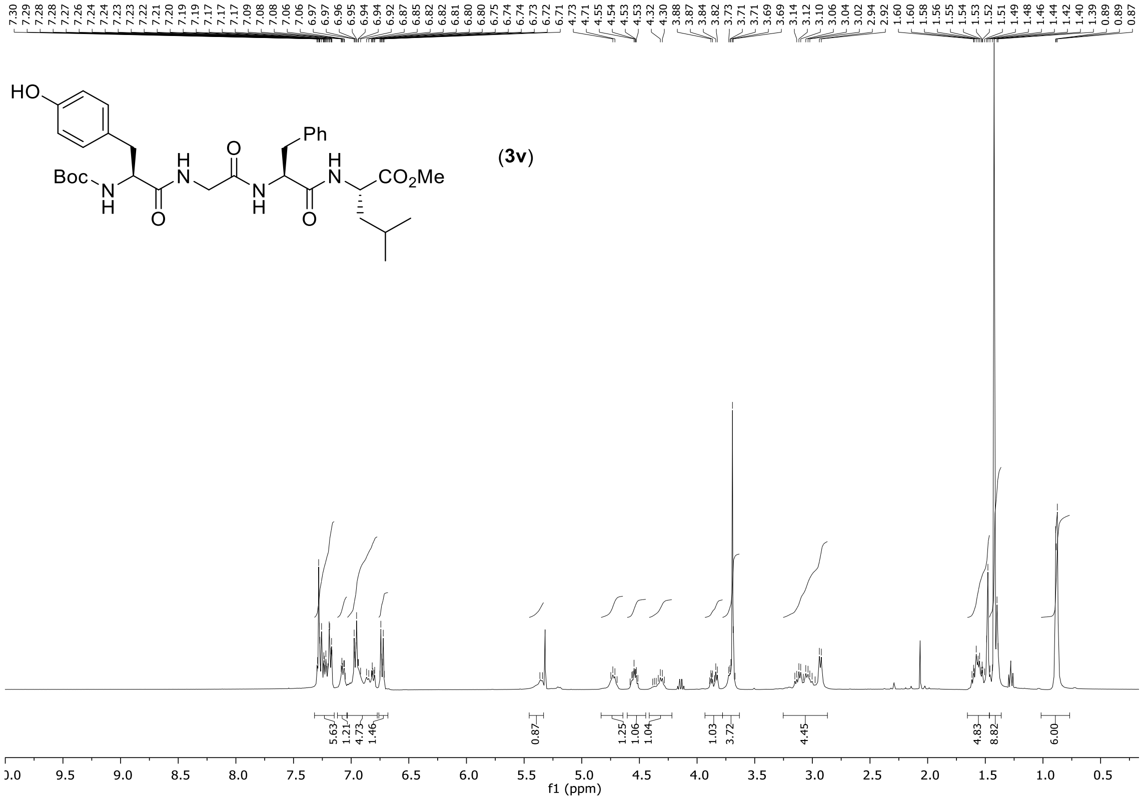

(**3v**)

^{13}C NMR (101 MHz, $CDCl_3$)

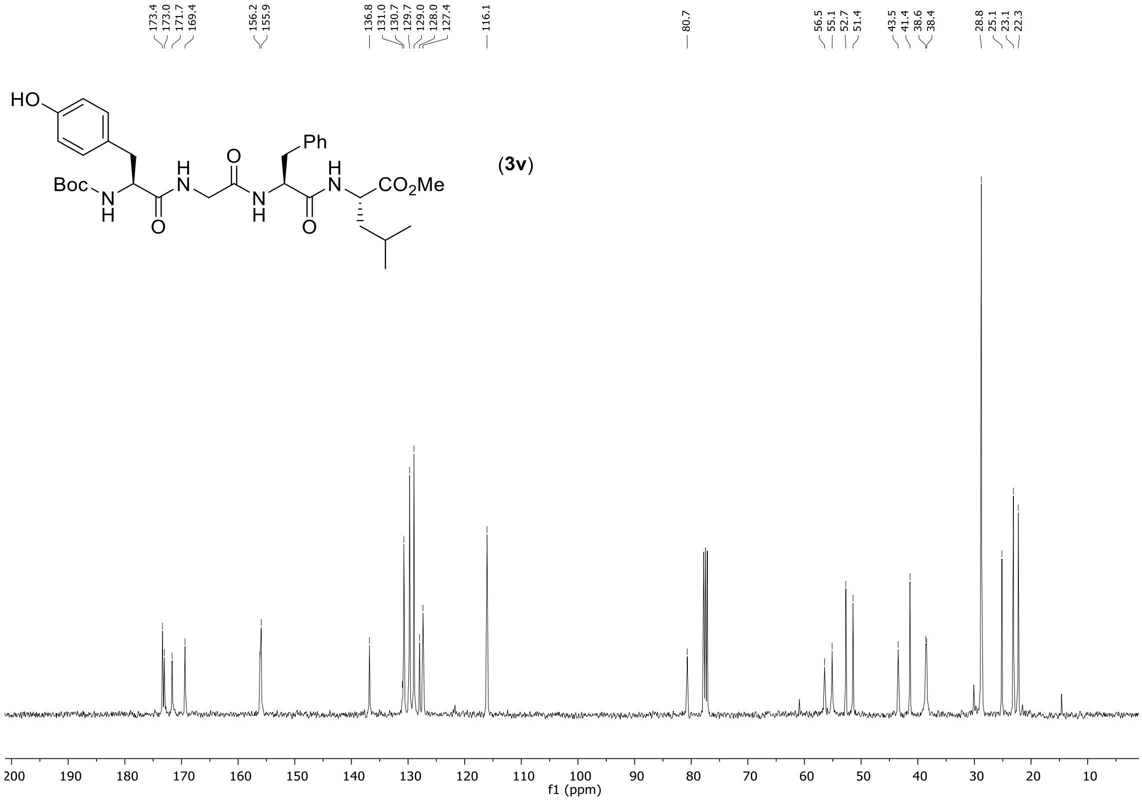

(**3v**)

^{1}H NMR (500 MHz, DMSO-d_6 at 80 ºC)

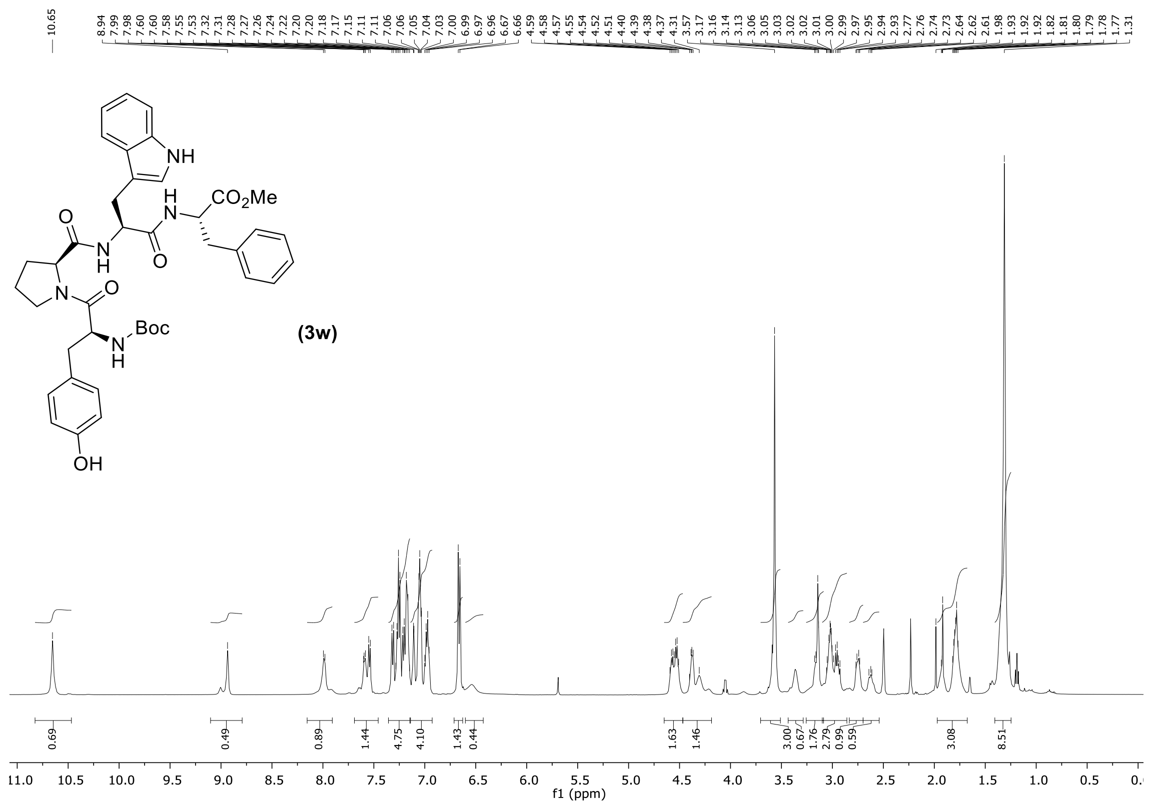

^{13}C NMR (126 MHz, DMSO-d_6 at 80 ºC)

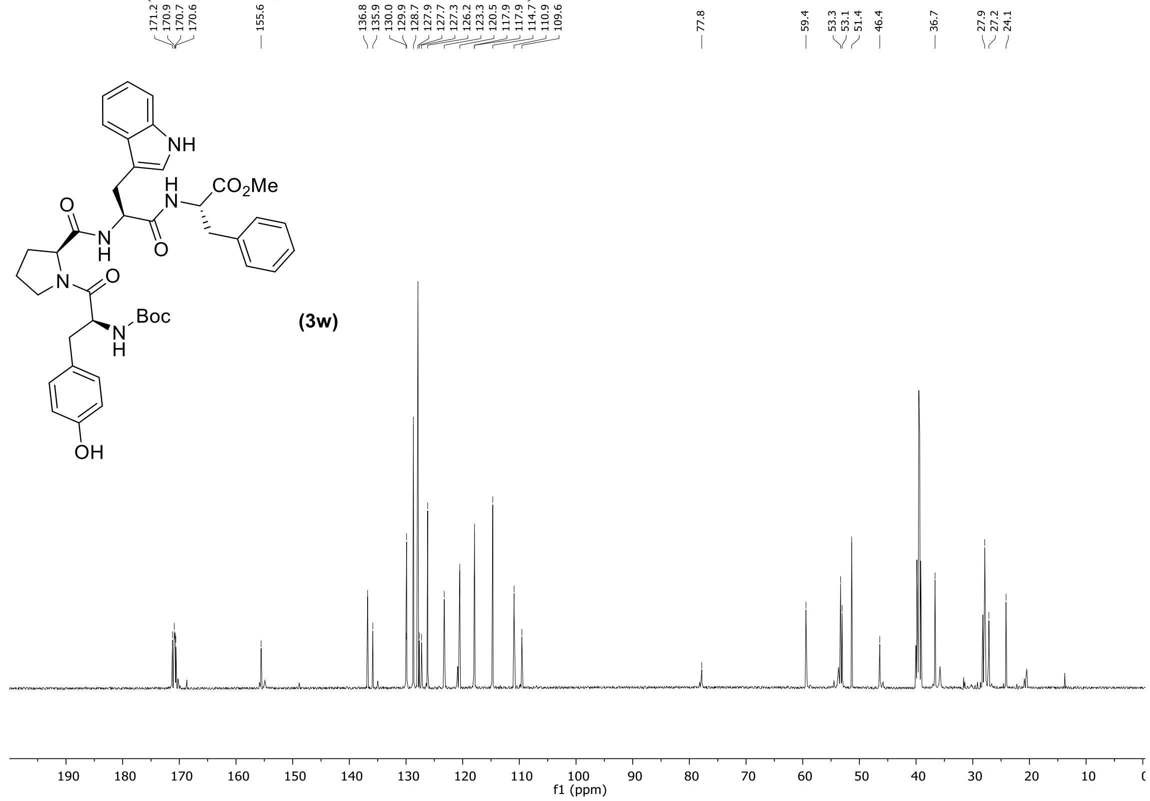

^{1}H NMR (500 MHz, DMSO-d_6 at 80 ºC)

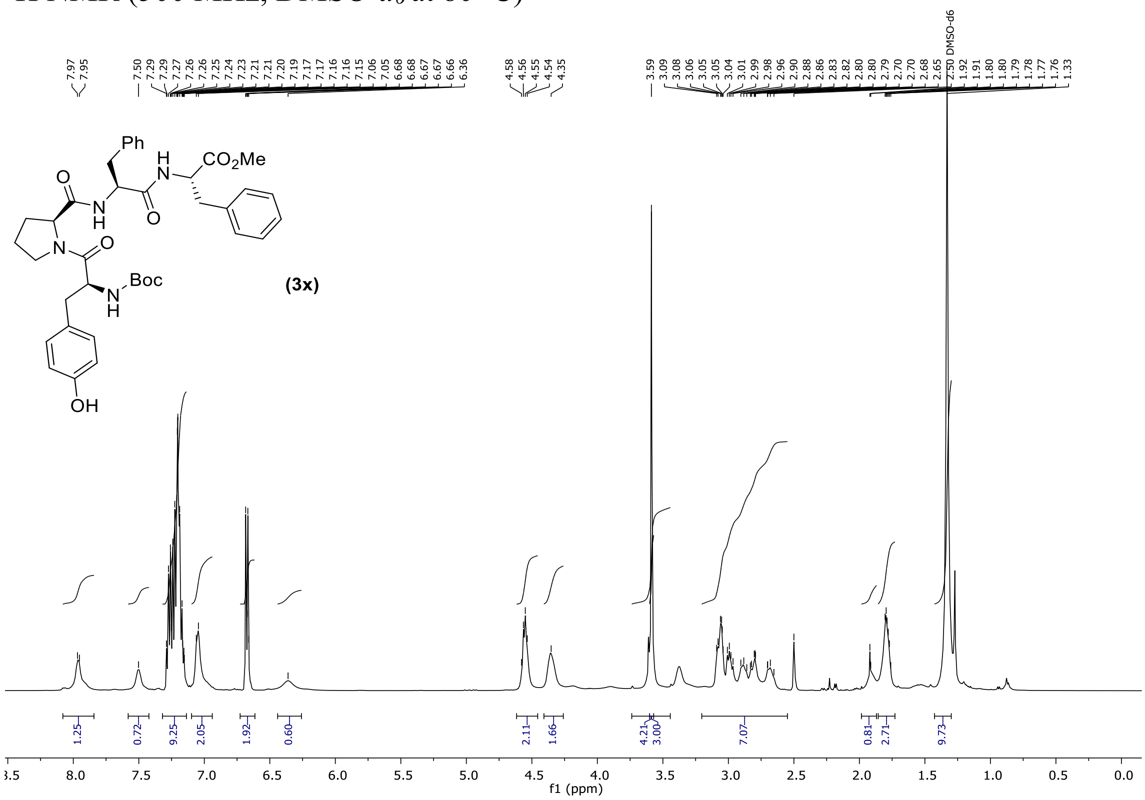

^{13}C NMR (126 MHz, DMSO-d_6 at 80º C)

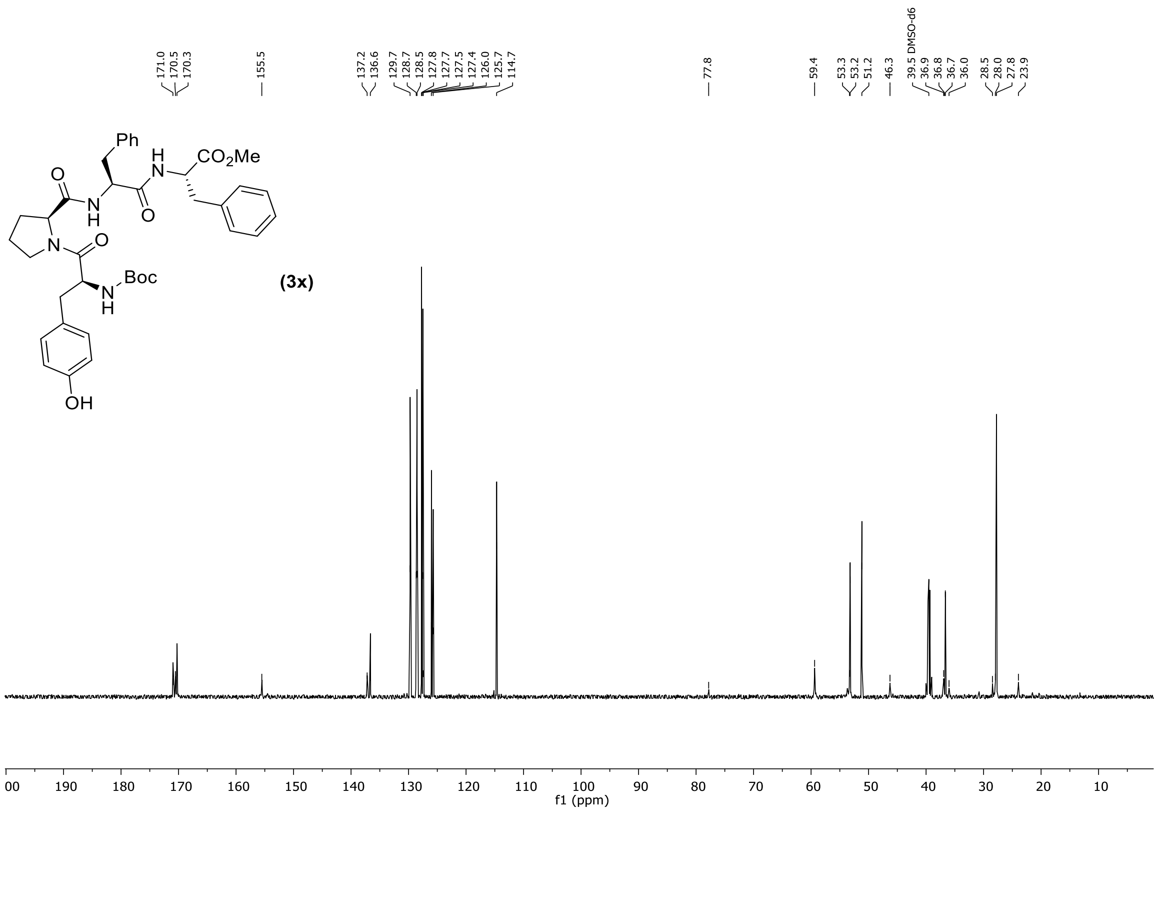

^{1}H NMR (400 MHz, $CDCl_3$)

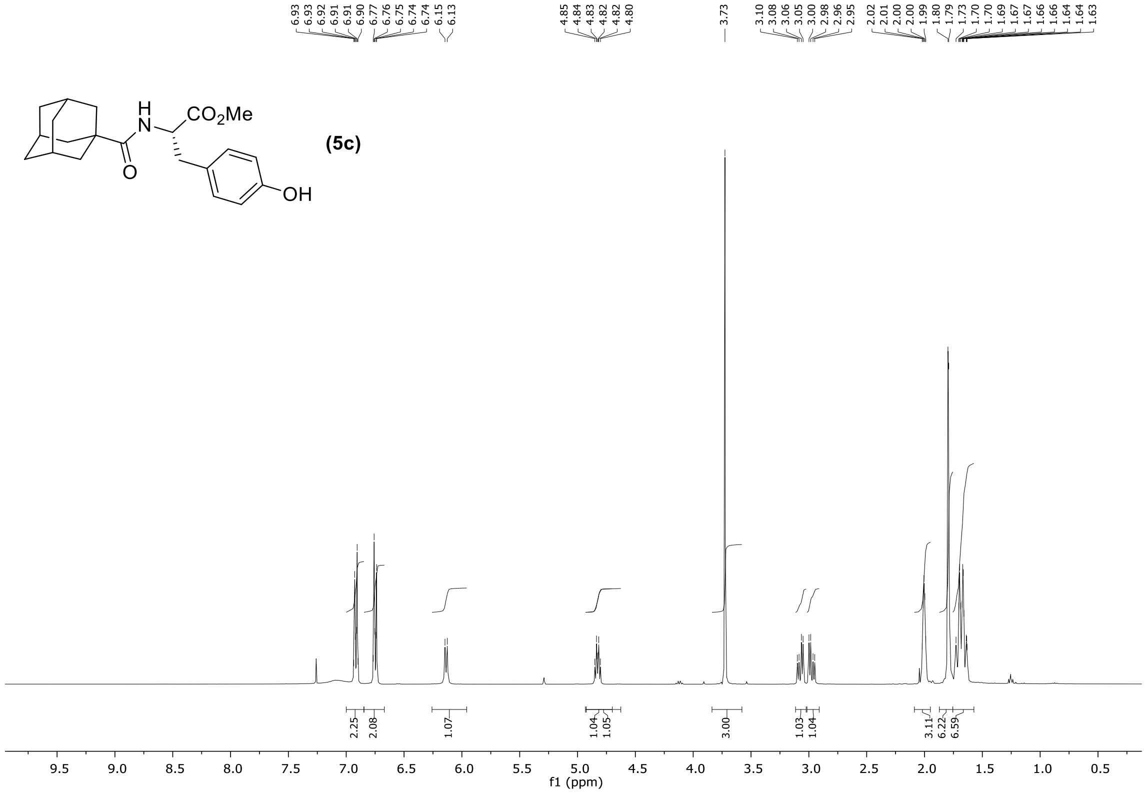

^{13}C NMR (101 MHz, $CDCl_3$)

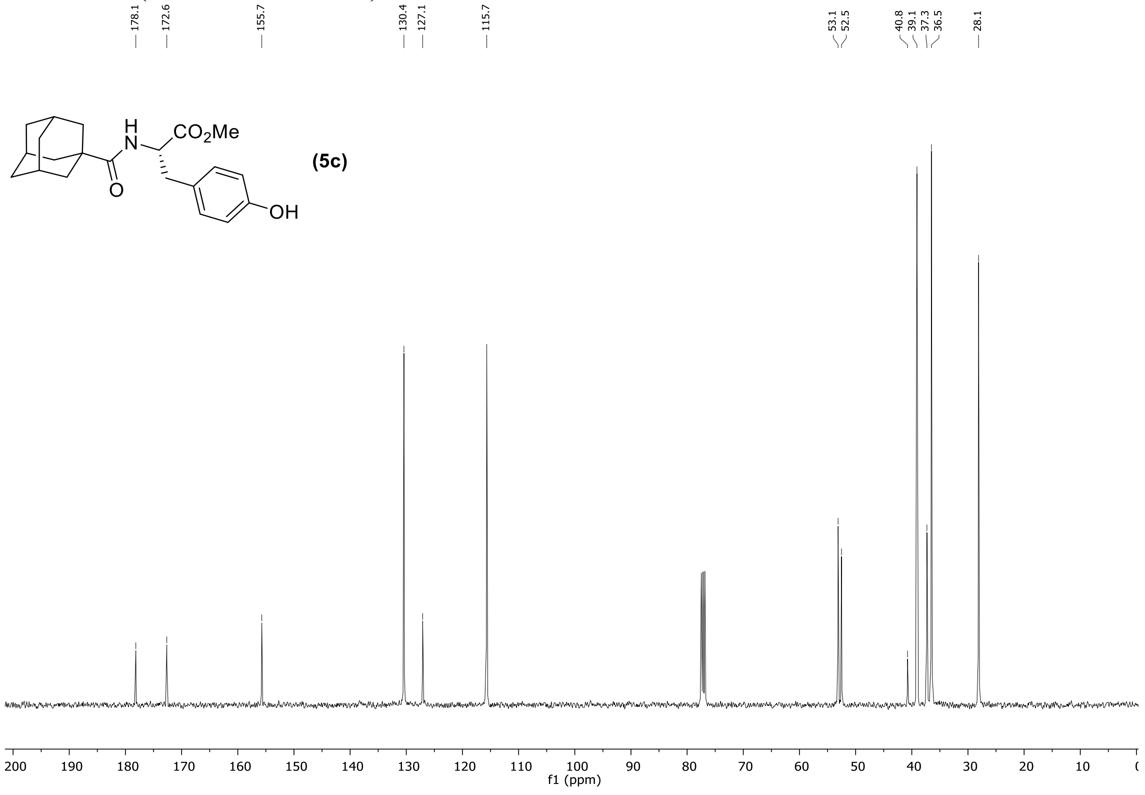

^{1}H NMR (400 MHz, $CDCl_3$)

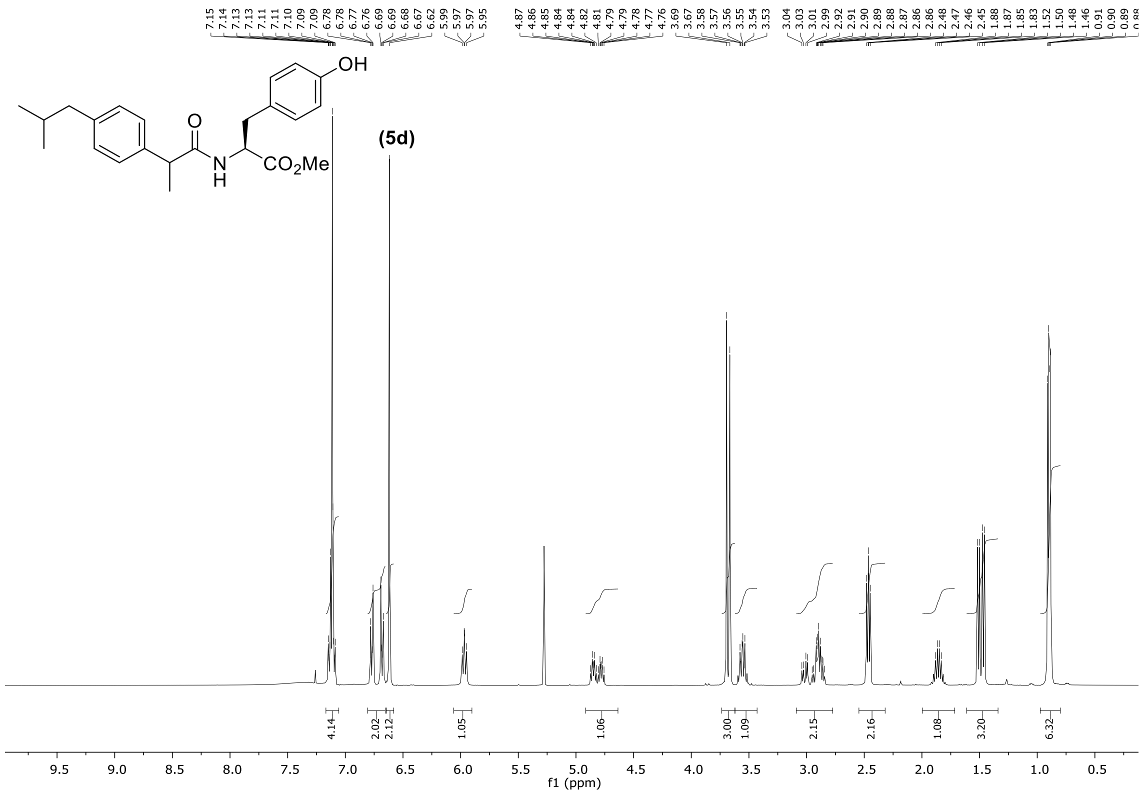

^{13}C NMR (101 MHz, $CDCl_3$)

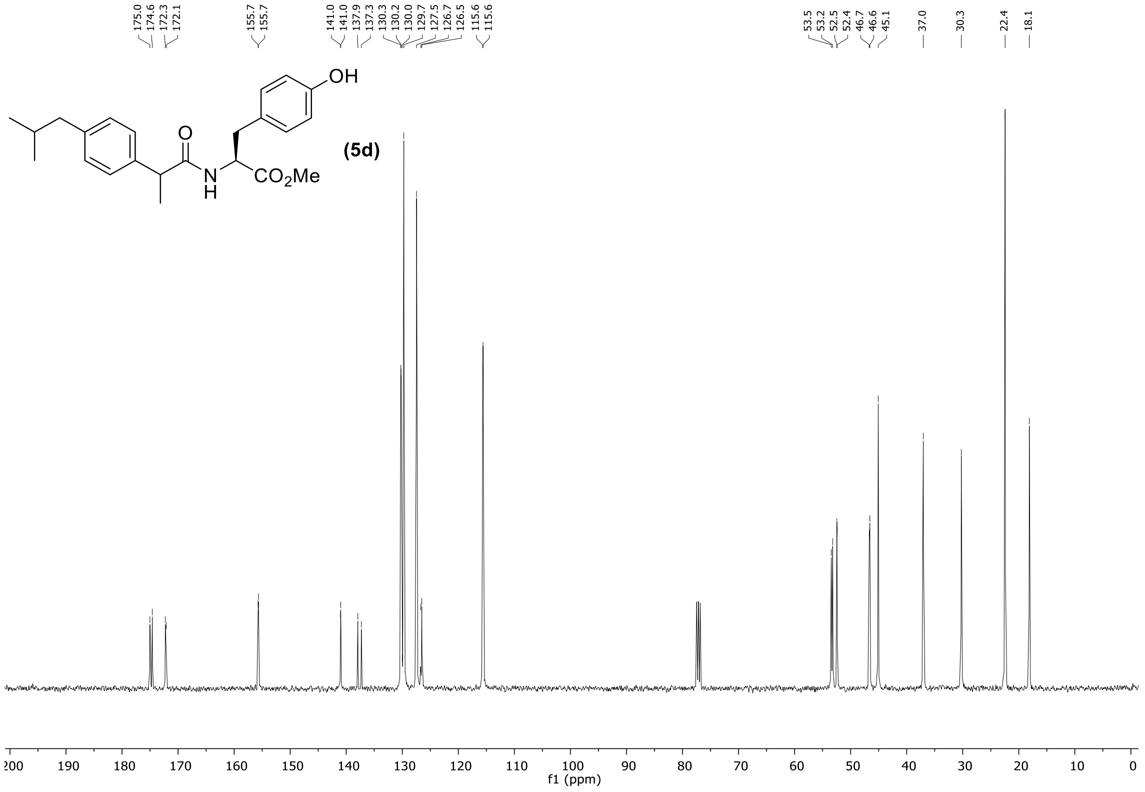

^{1}H NMR (400 MHz, $CDCl_3$)

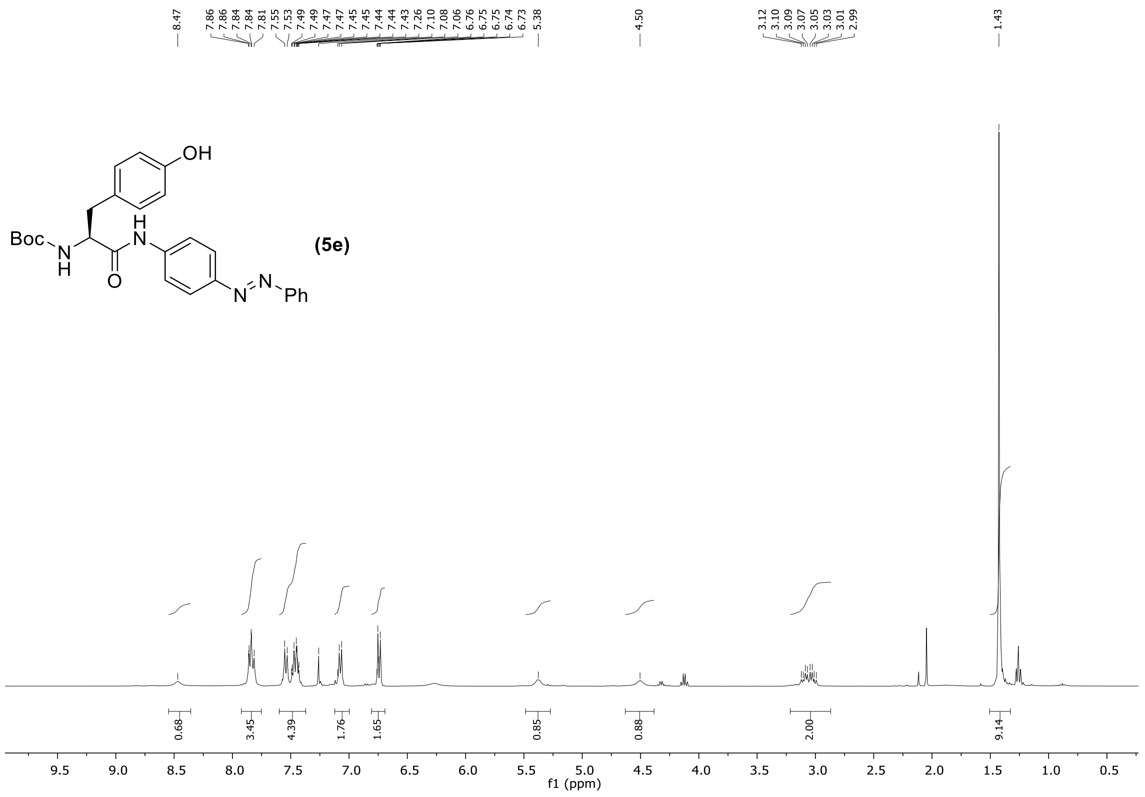

^{13}C NMR (101 MHz, $CDCl_3$)

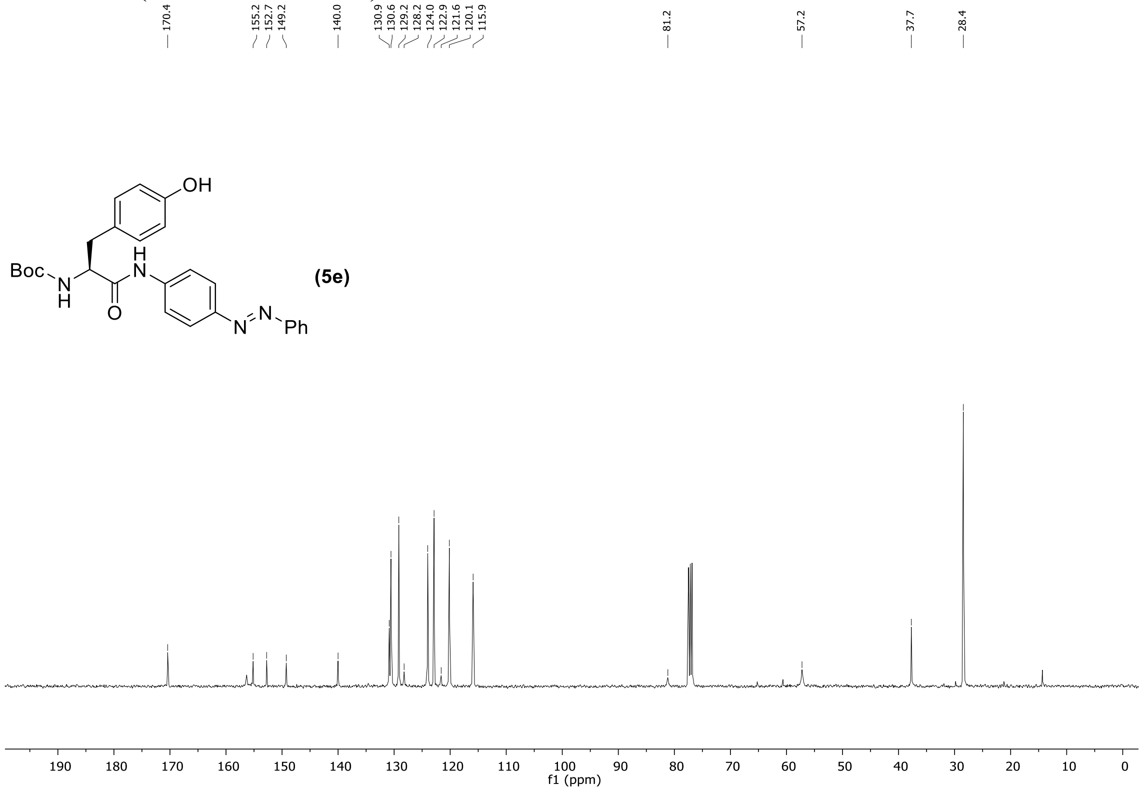

^{1}H NMR (400 MHz, $CDCl_3$)

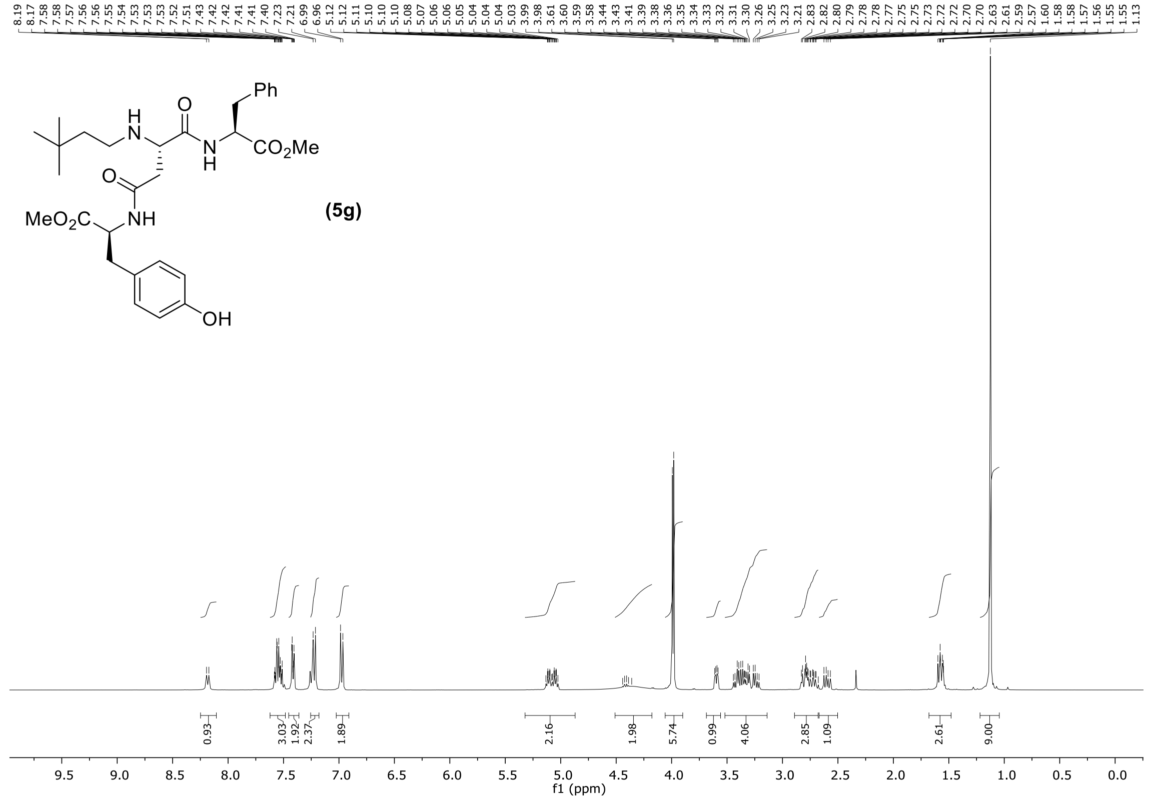

^{13}C NMR (101 MHz, $CDCl_3$)

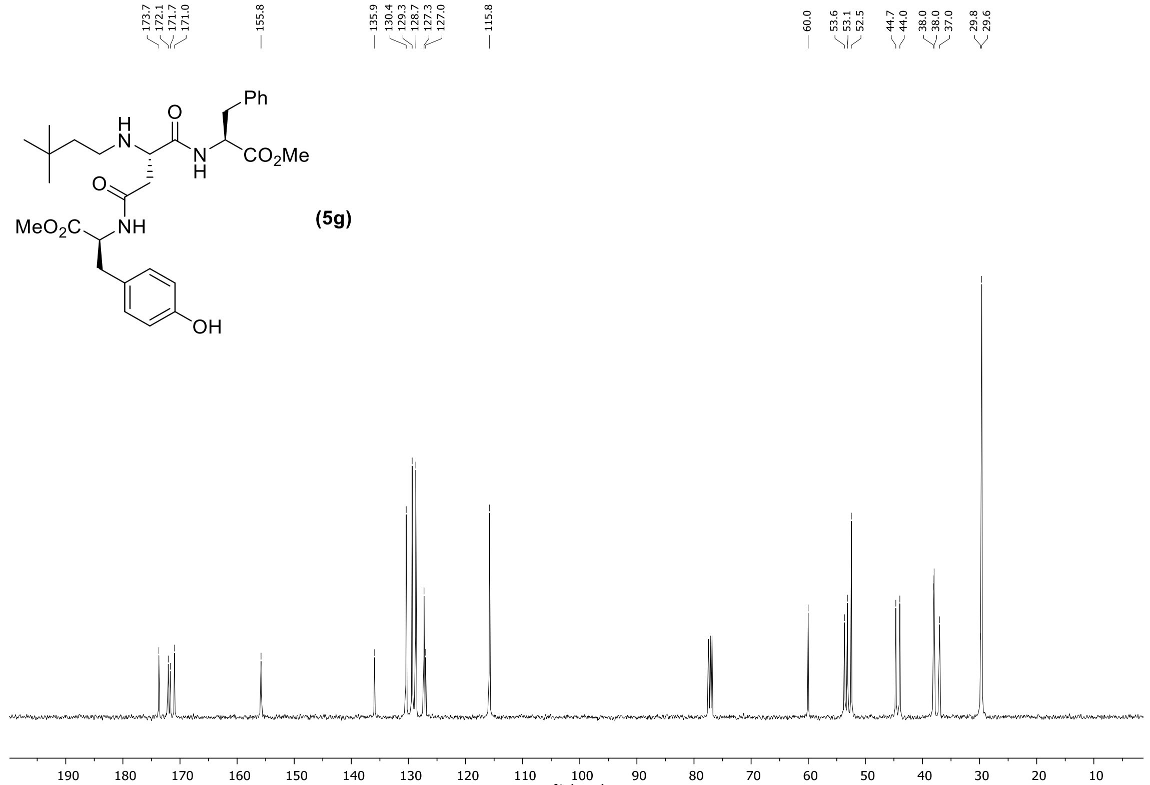

^{1}H NMR (400 MHz, DMSO-d_6 **at rt)**

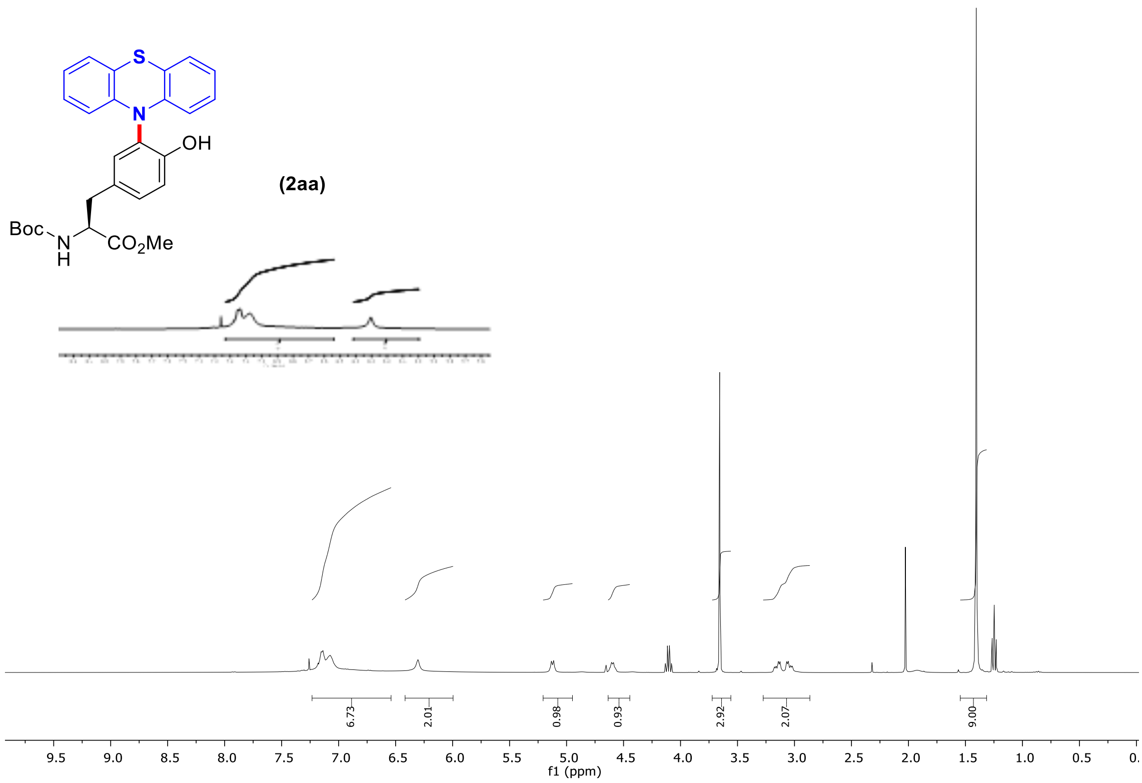

(2aa)

^{13}C NMR (101 MHz, DMSO-d_6 **at rt)**

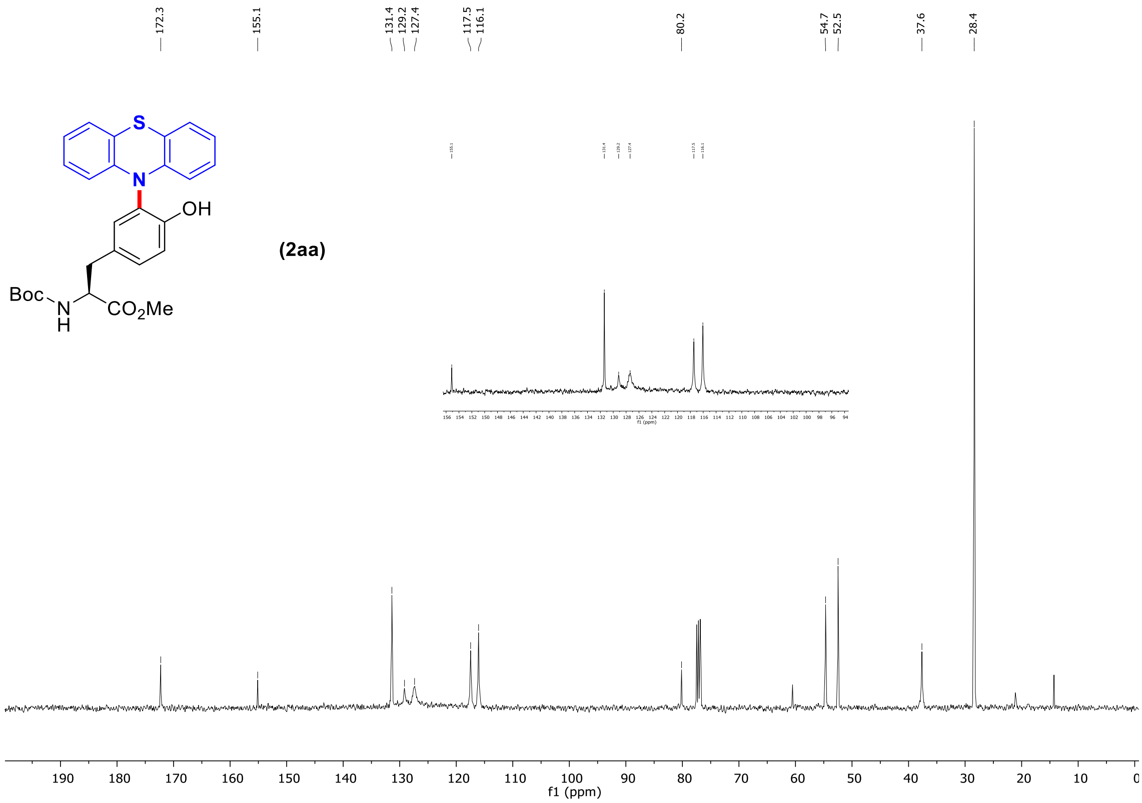

(2aa)

^{1}H NMR (500 MHz, DMSO-d_6 at 80 ºC)

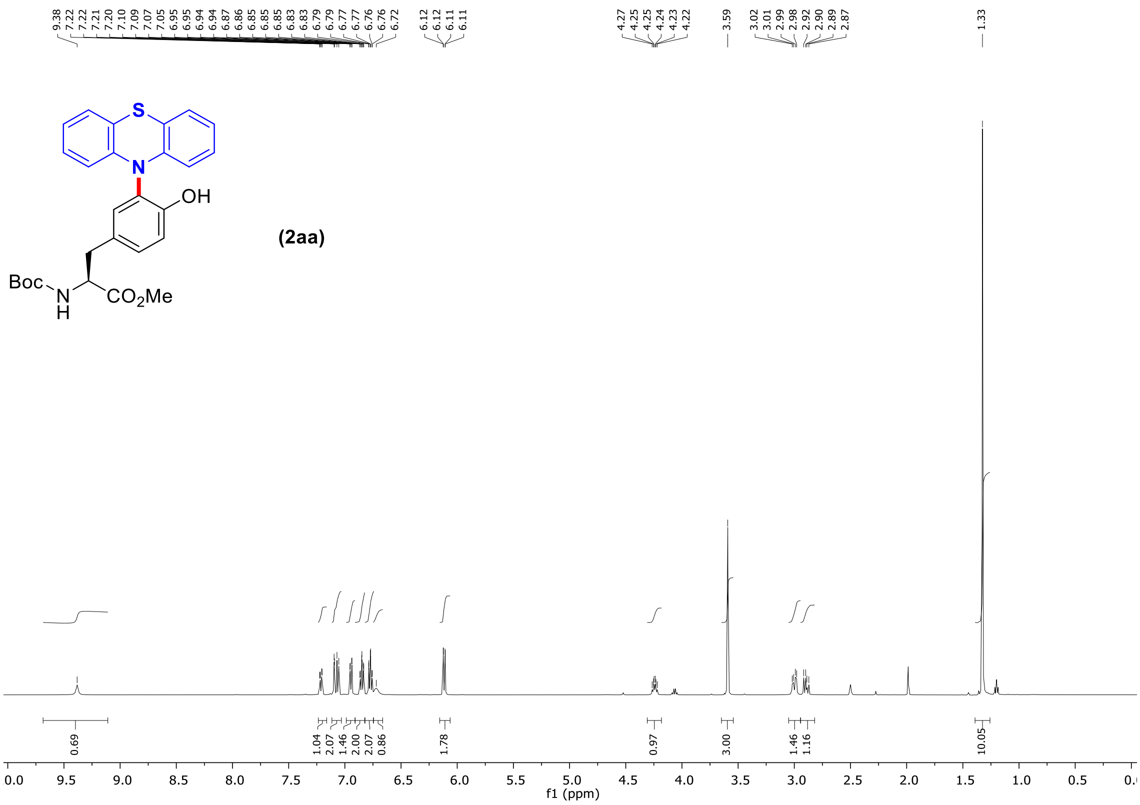

^{13}C NMR (126 MHz, DMSO-d_6 at 80 ºC)

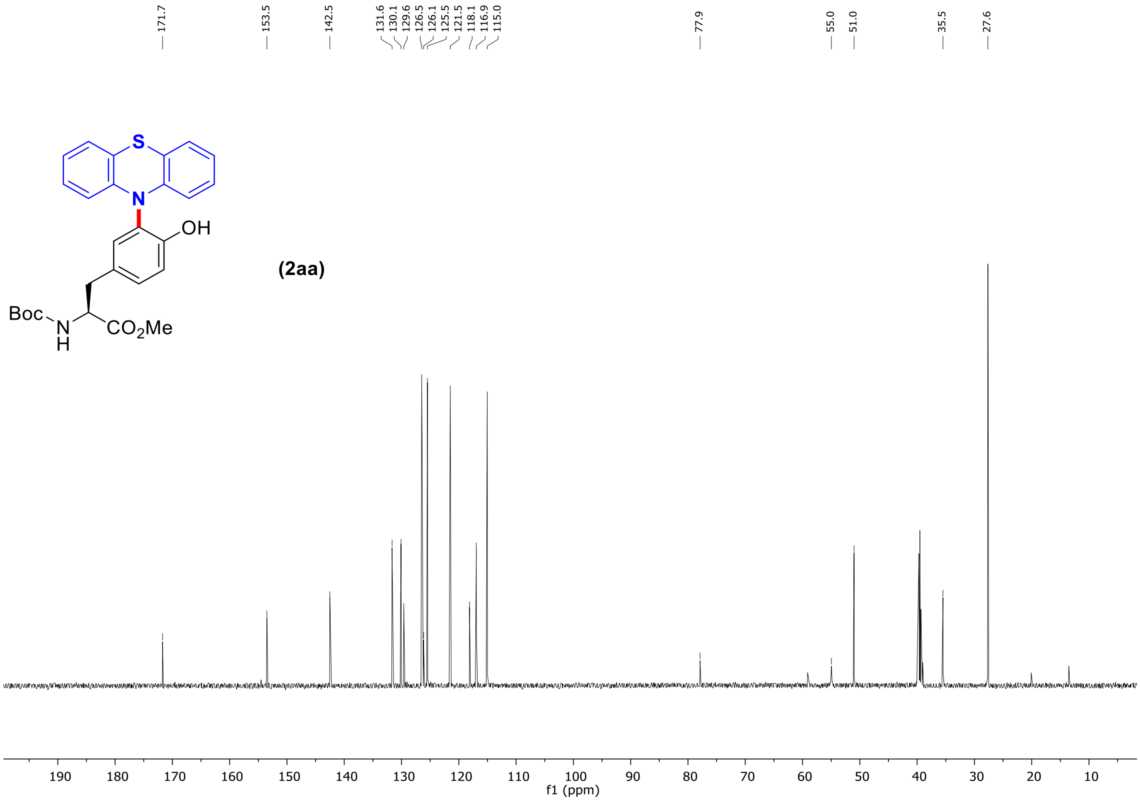

^{1}H NMR (500 MHz, DMSO-d_6 at 80 ºC)

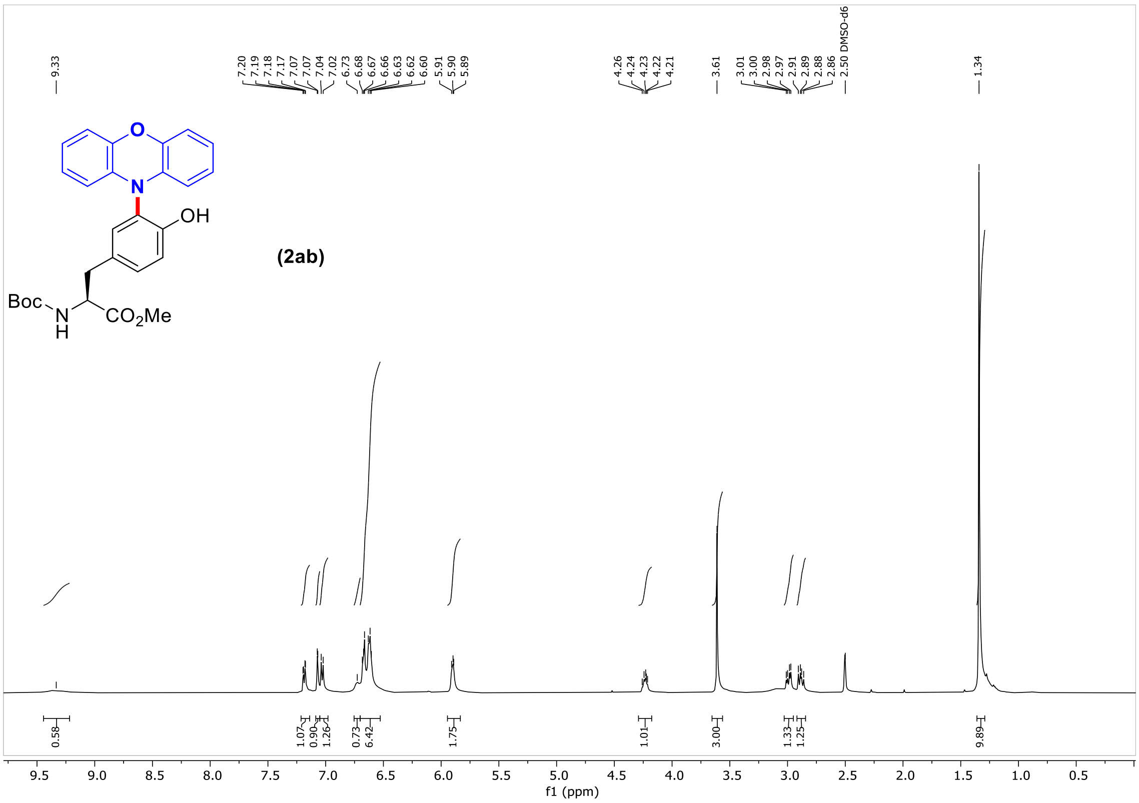

^{13}C NMR (126 MHz, DMSO-d_6 at 80 ºC)

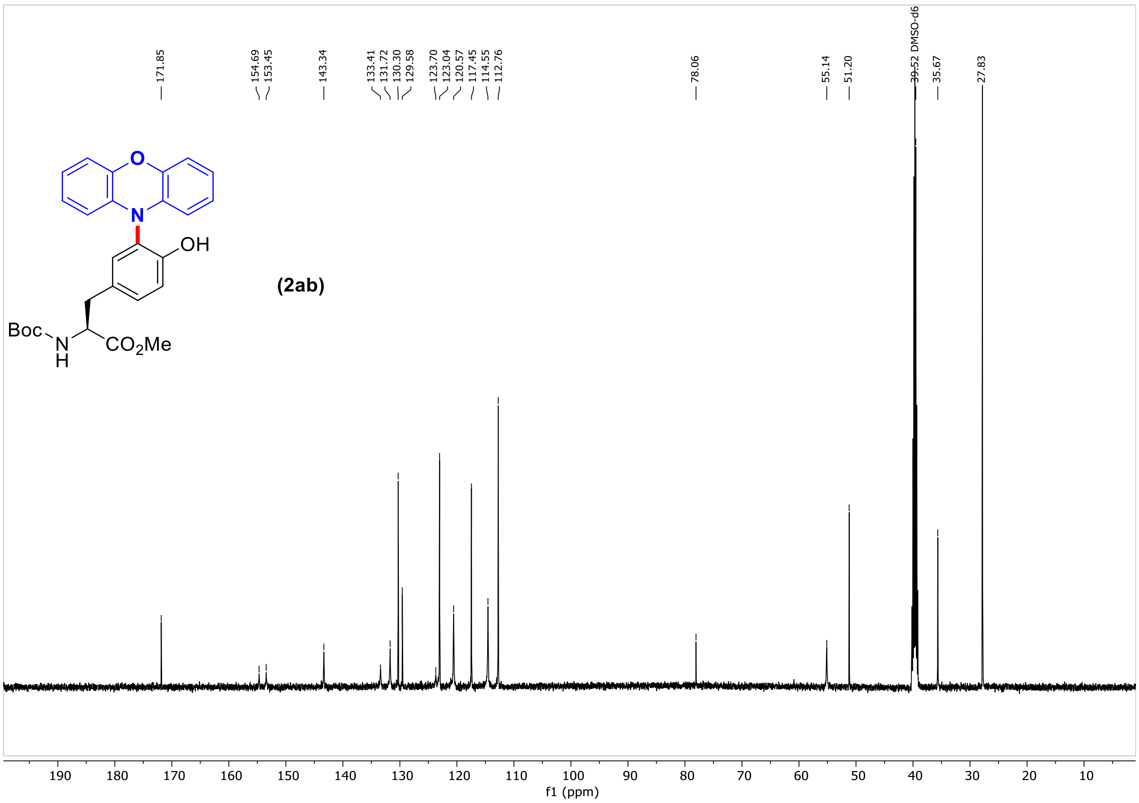

^{1}H NMR (500 MHz, DMSO-d_6 at 80 ºC)

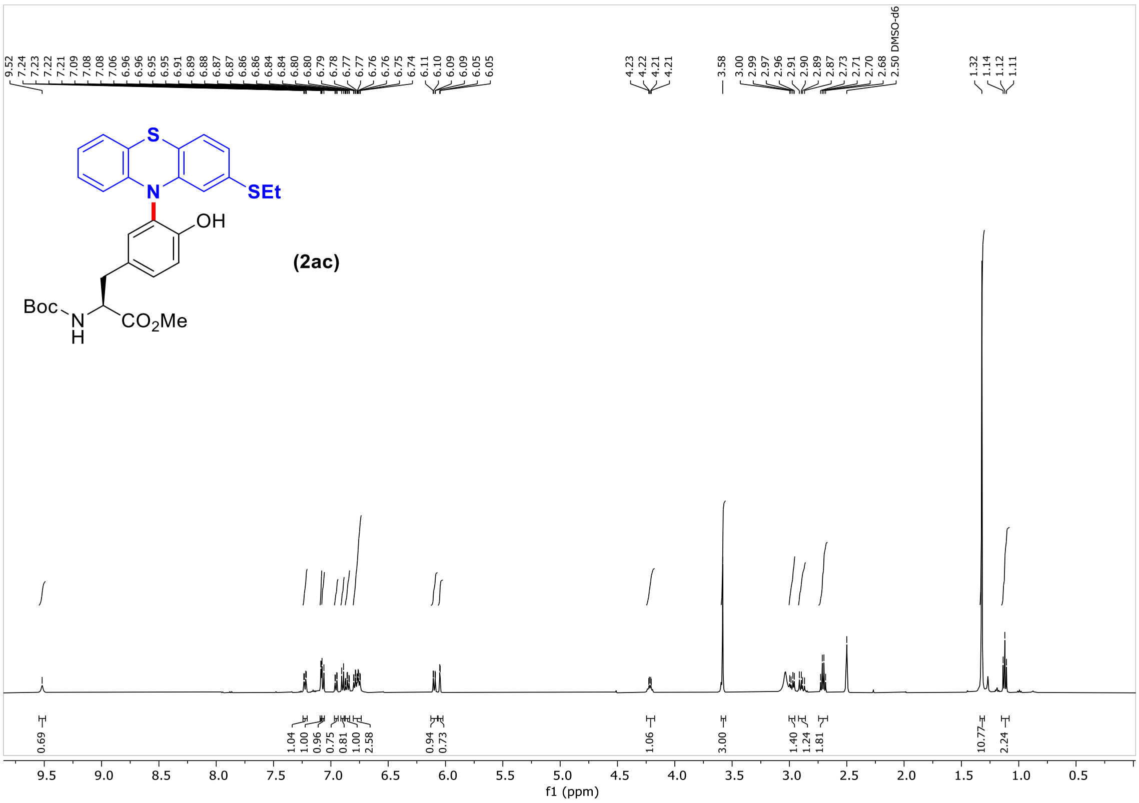

^{13}C NMR (126 MHz, DMSO-d_6 at 80 ºC)

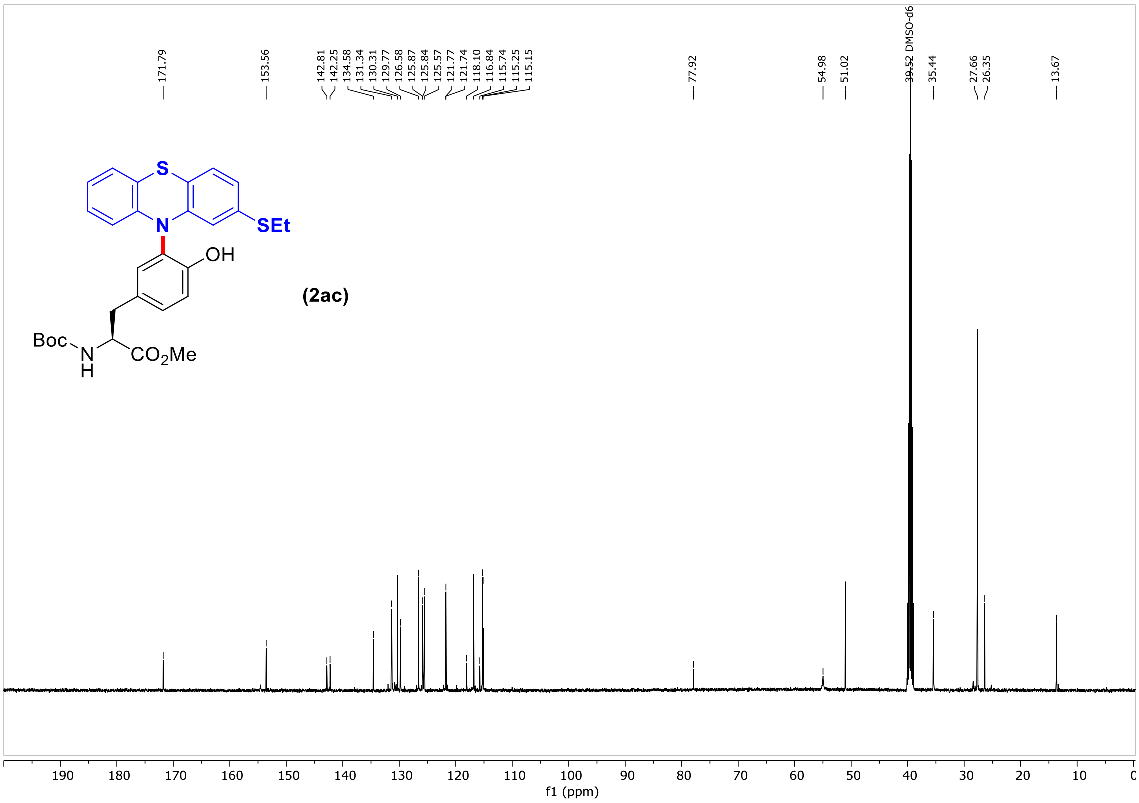

^{1}H NMR (500 MHz, DMSO-d_6 at 80 ºC)

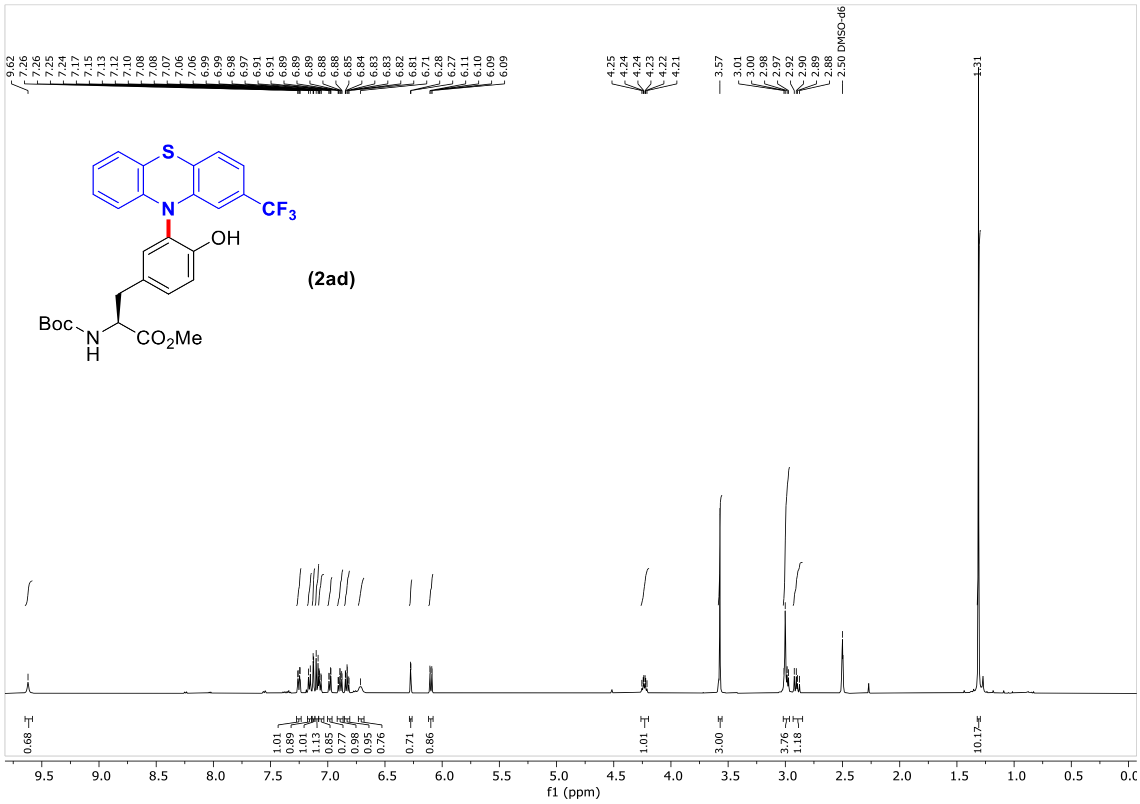

^{13}C NMR (126 MHz, DMSO-d_6 at 80 ºC)

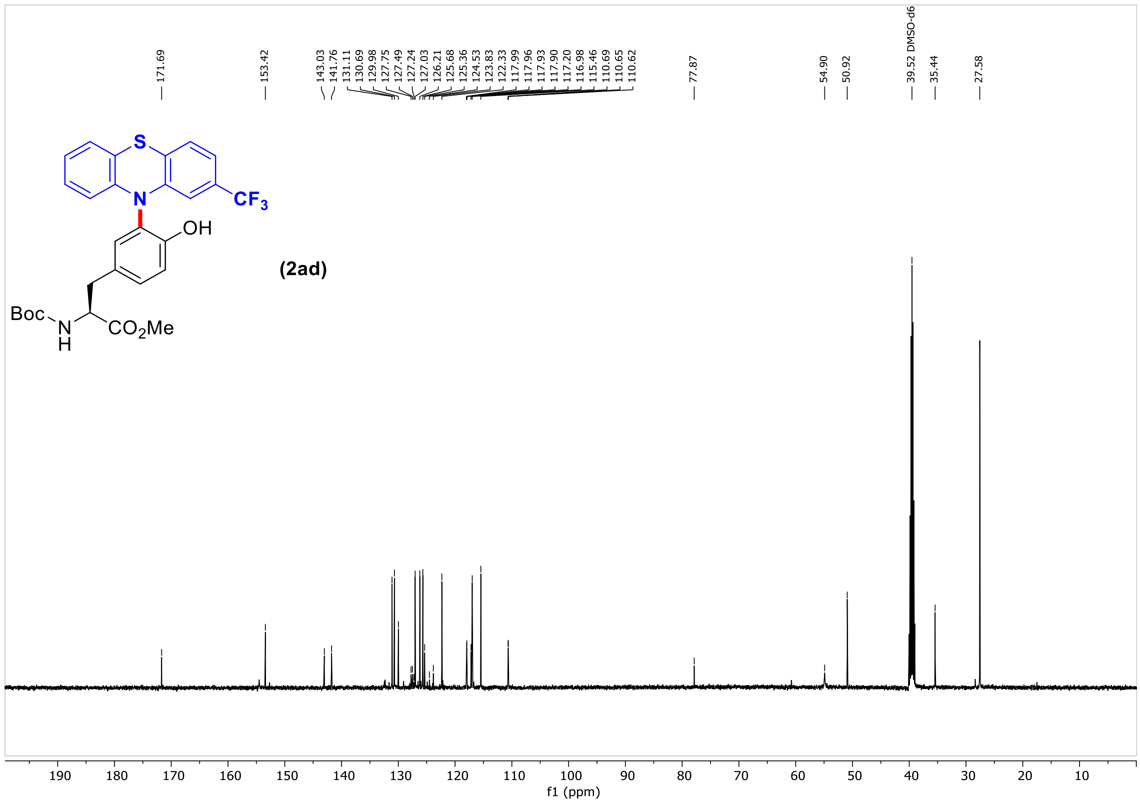

^{1}H NMR (500 MHz, DMSO-d_6 at 80 ºC)

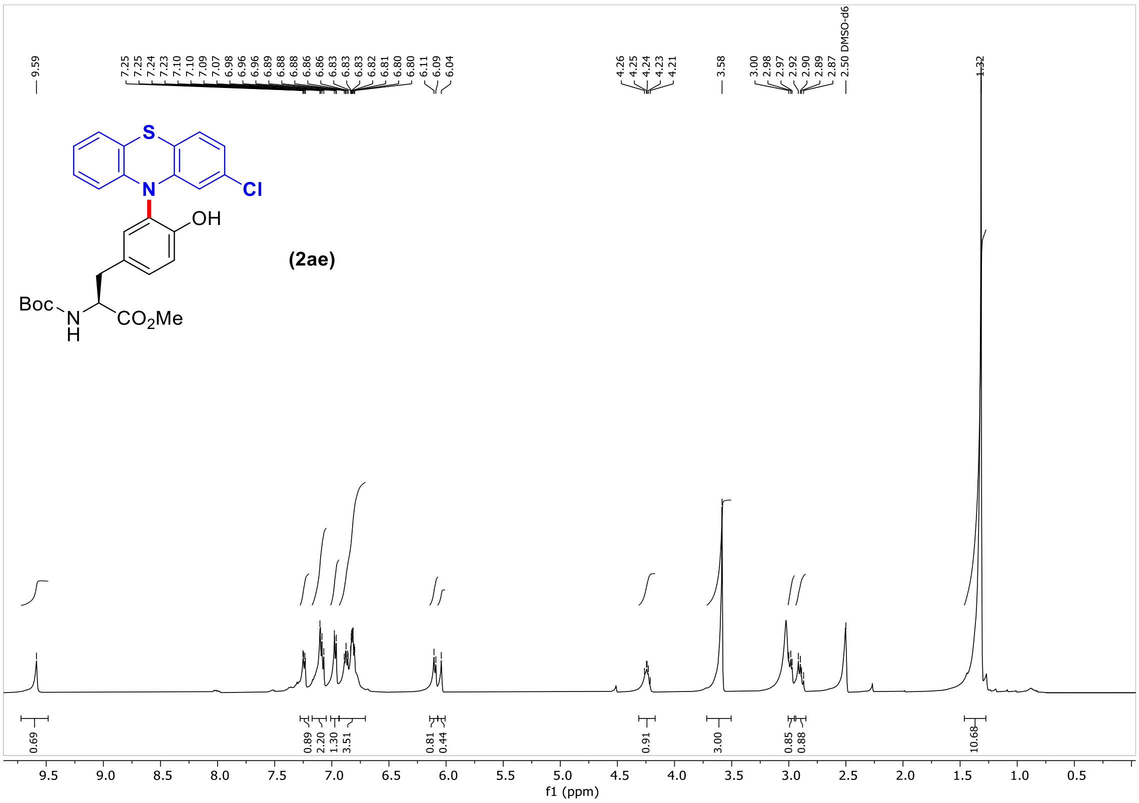

^{13}C NMR (126 MHz, DMSO-d_6 at 80 ºC)

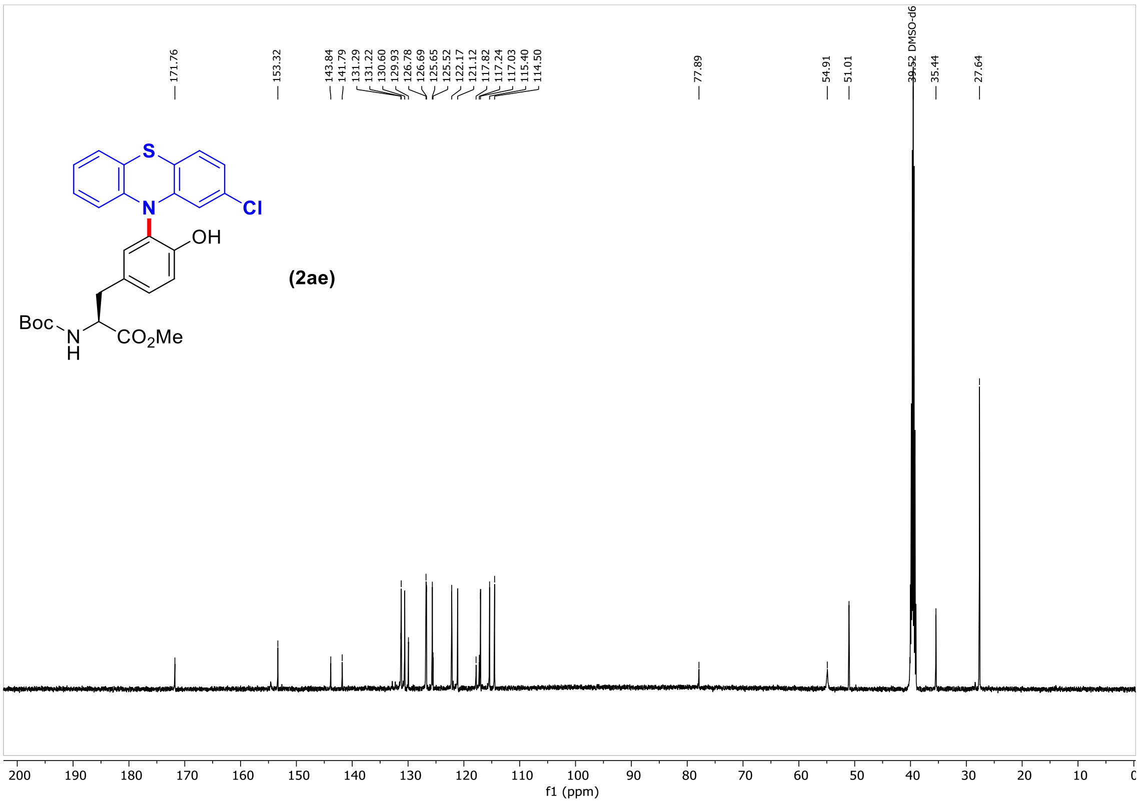

^{1}H NMR (500 MHz, DMSO-d_6 at 80 ºC)

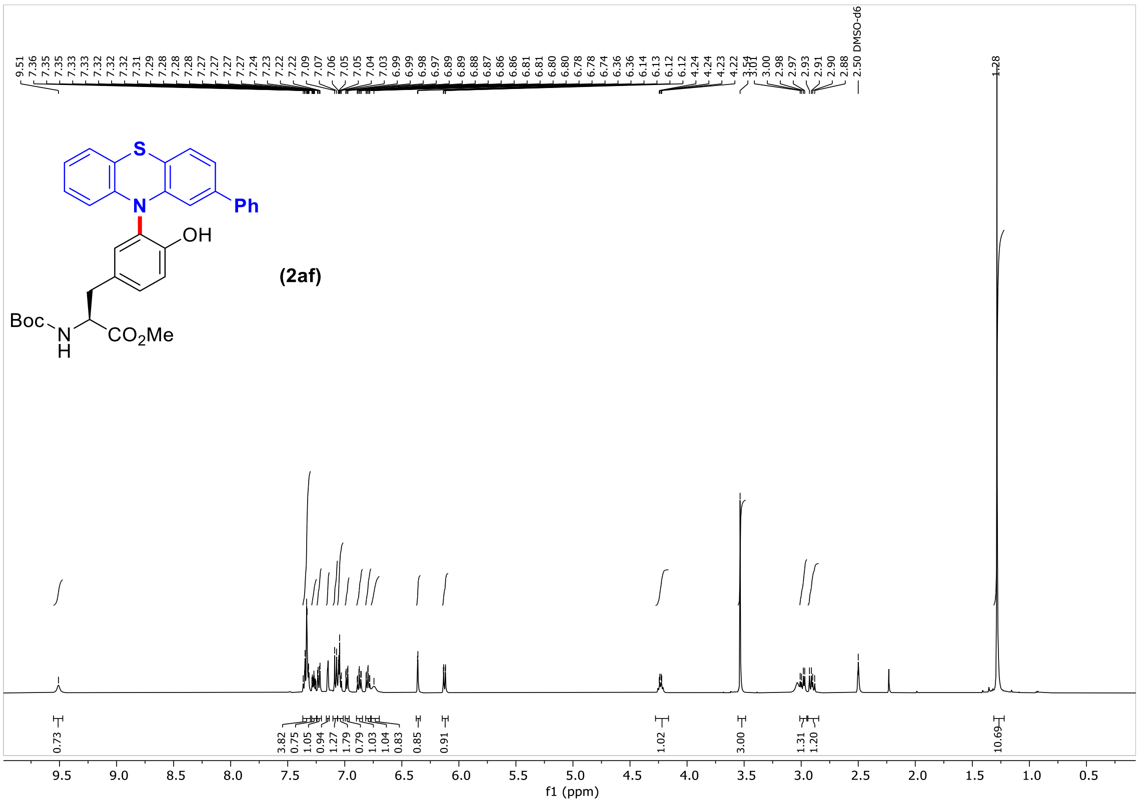

^{13}C NMR (126 MHz, DMSO-d_6 at 80 ºC)

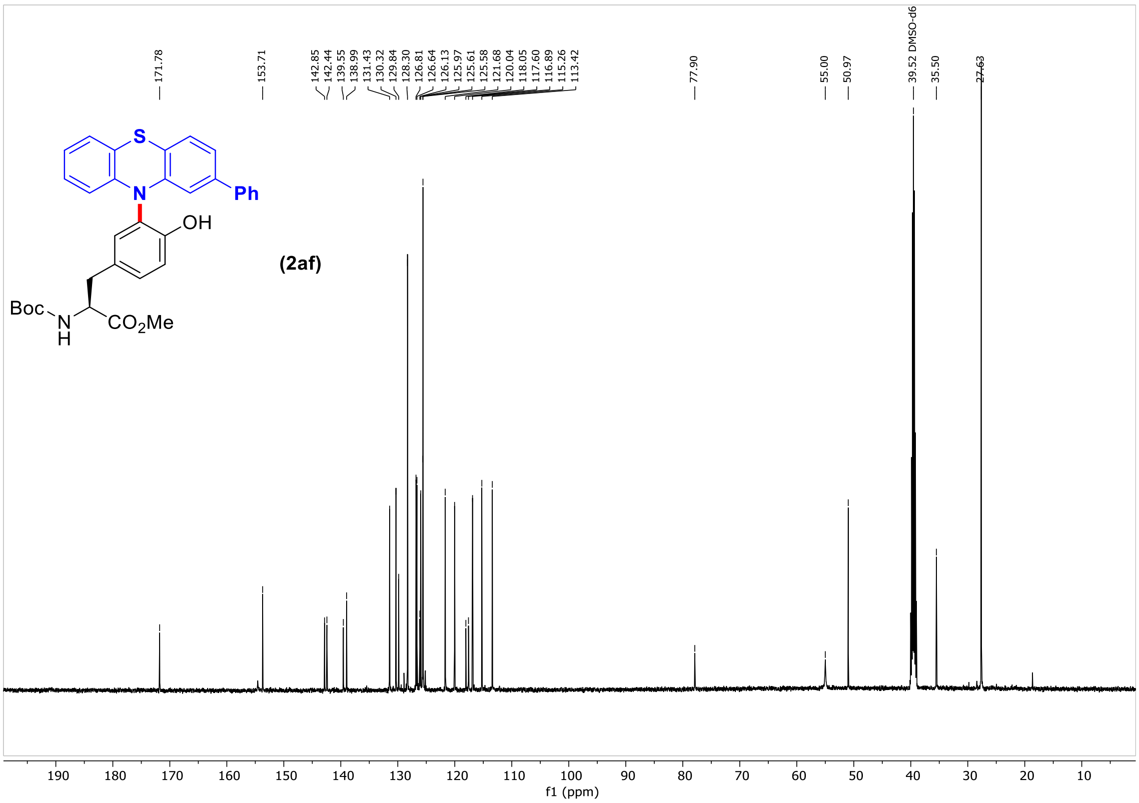

^{1}H NMR (500 MHz, DMSO-d_6 at 80 ºC)

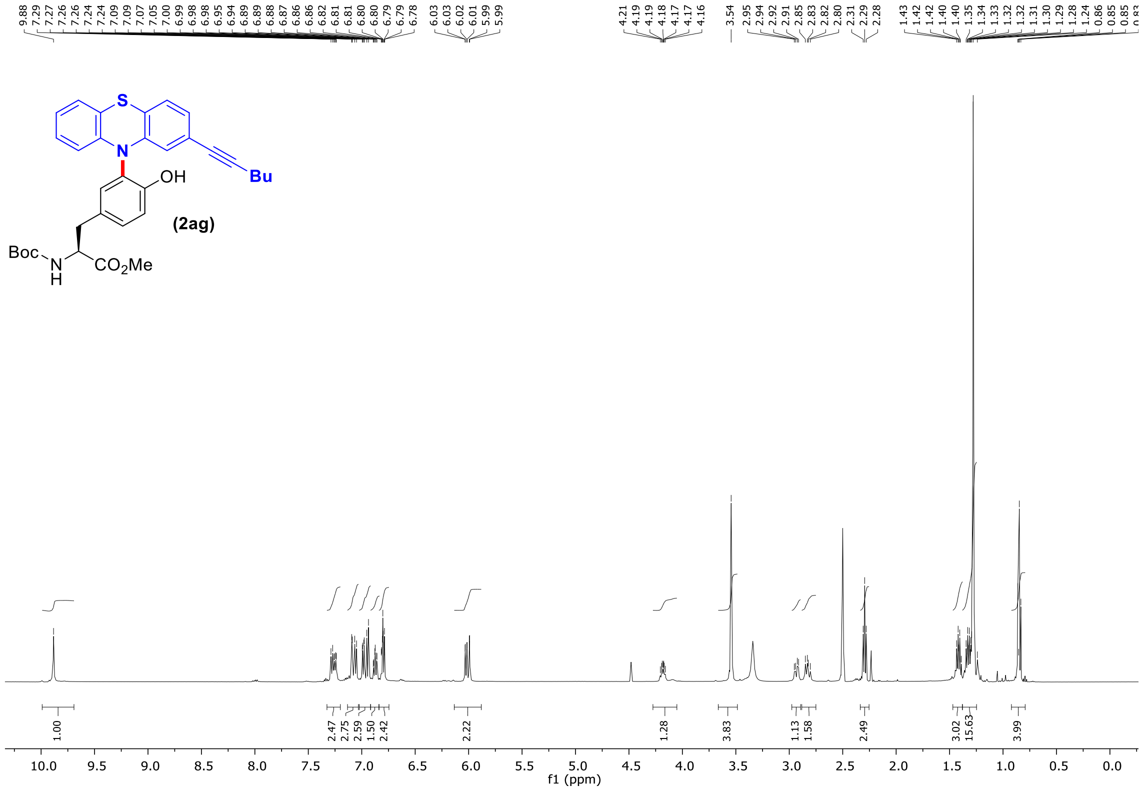

^{13}C NMR (126 MHz, DMSO-d_6 at 80 ºC)

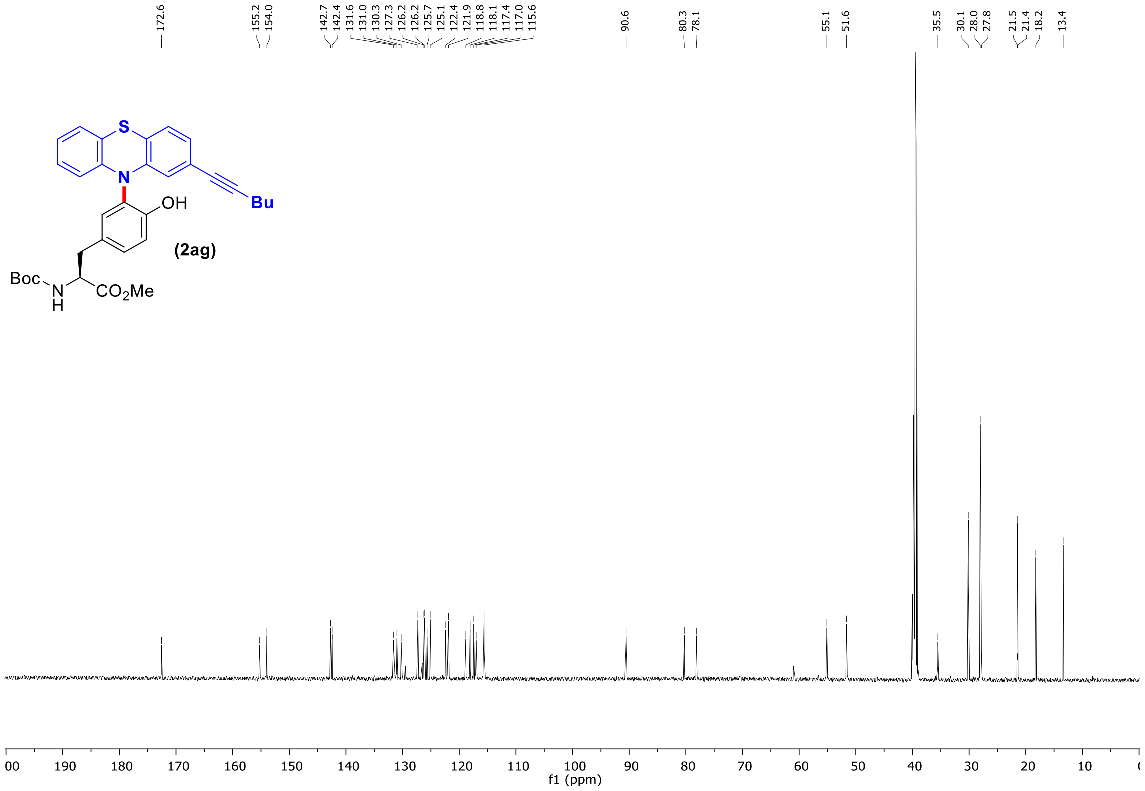

^{1}H NMR (500 MHz, DMSO-d_6 at 80 ºC)

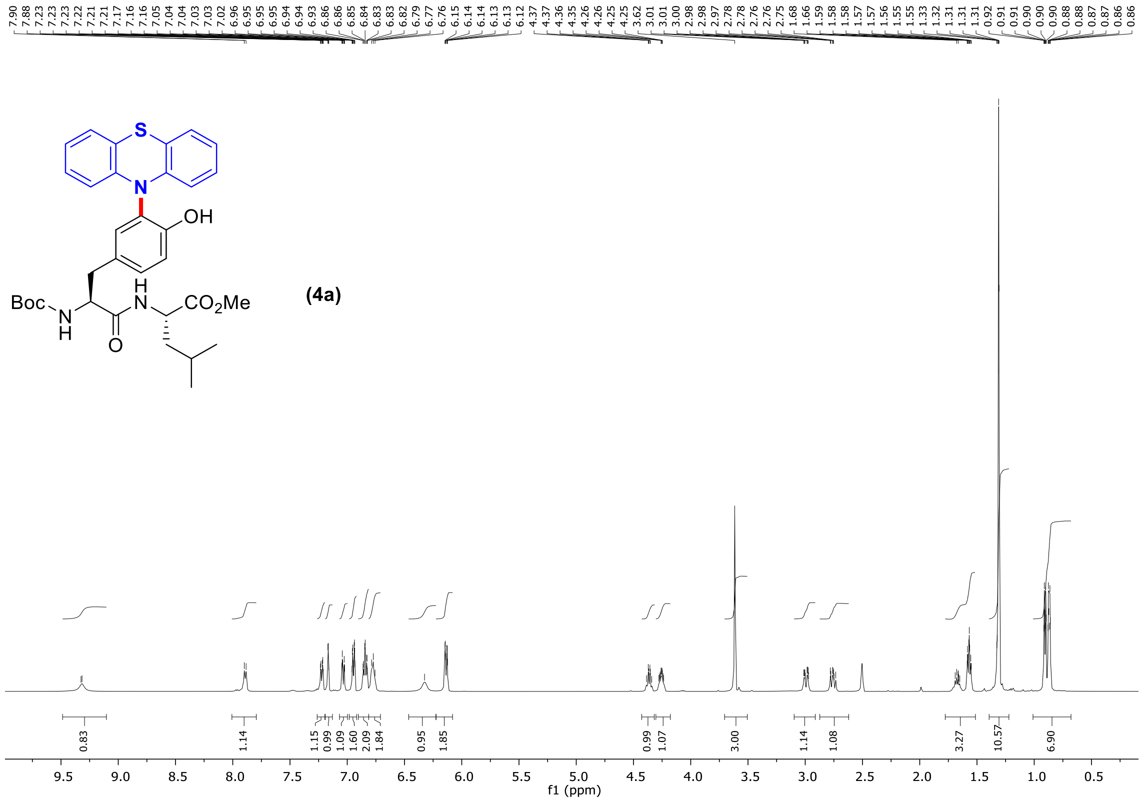

^{13}C NMR (126 MHz, DMSO-d_6 at 80 ºC)

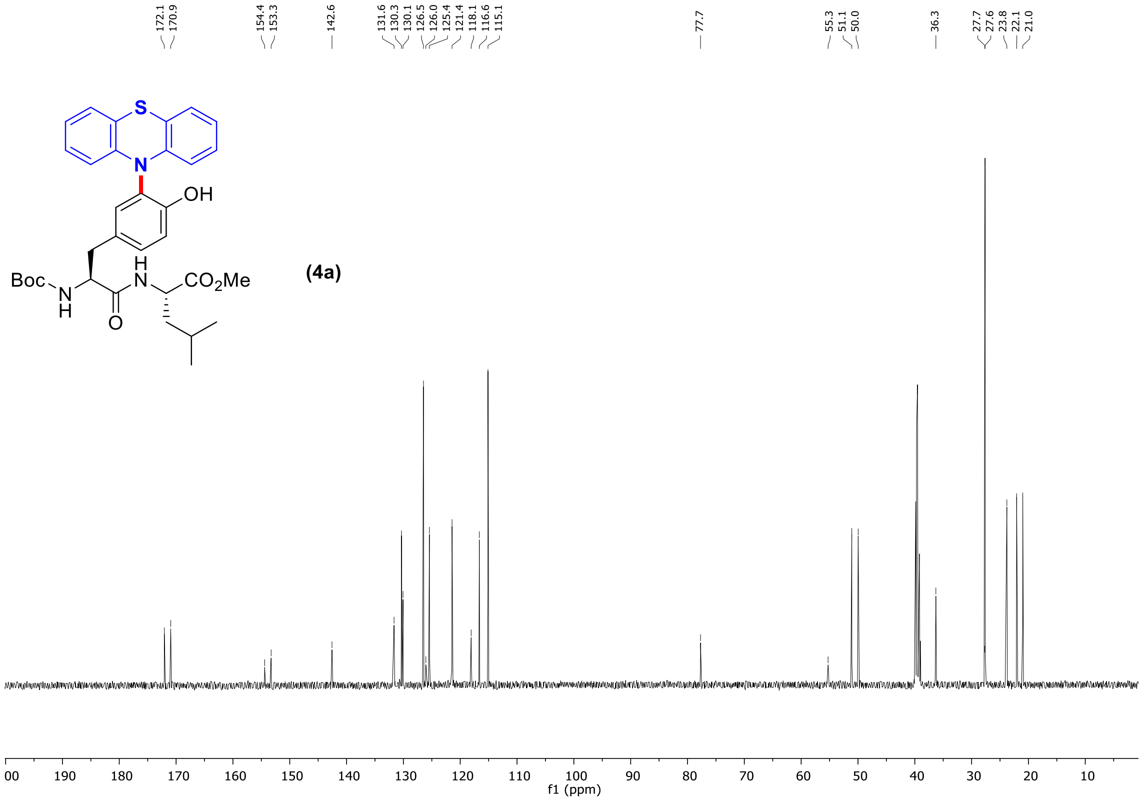

^{1}H NMR (500 MHz, DMSO-d_6 at 80 ºC)

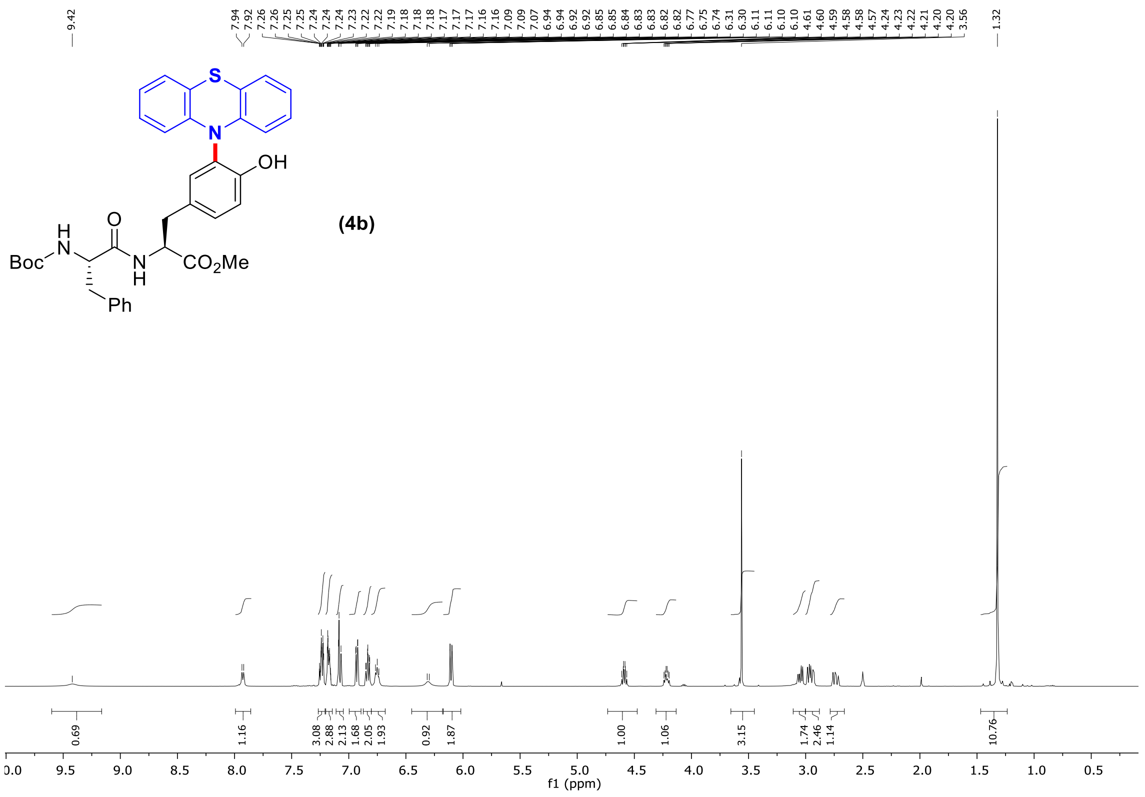

^{13}C NMR (126 MHz, DMSO-d_6 at 80 ºC)

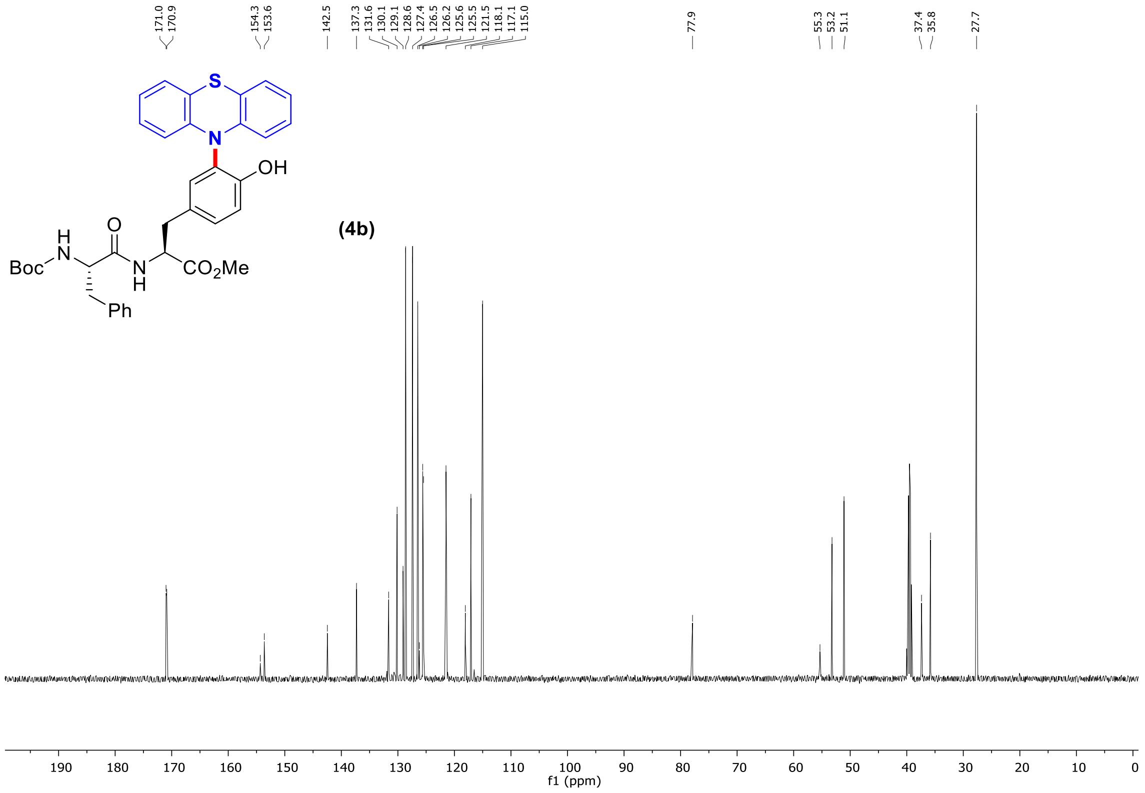

^{1}H NMR (500 MHz, DMSO-d_6 at 80 ºC)

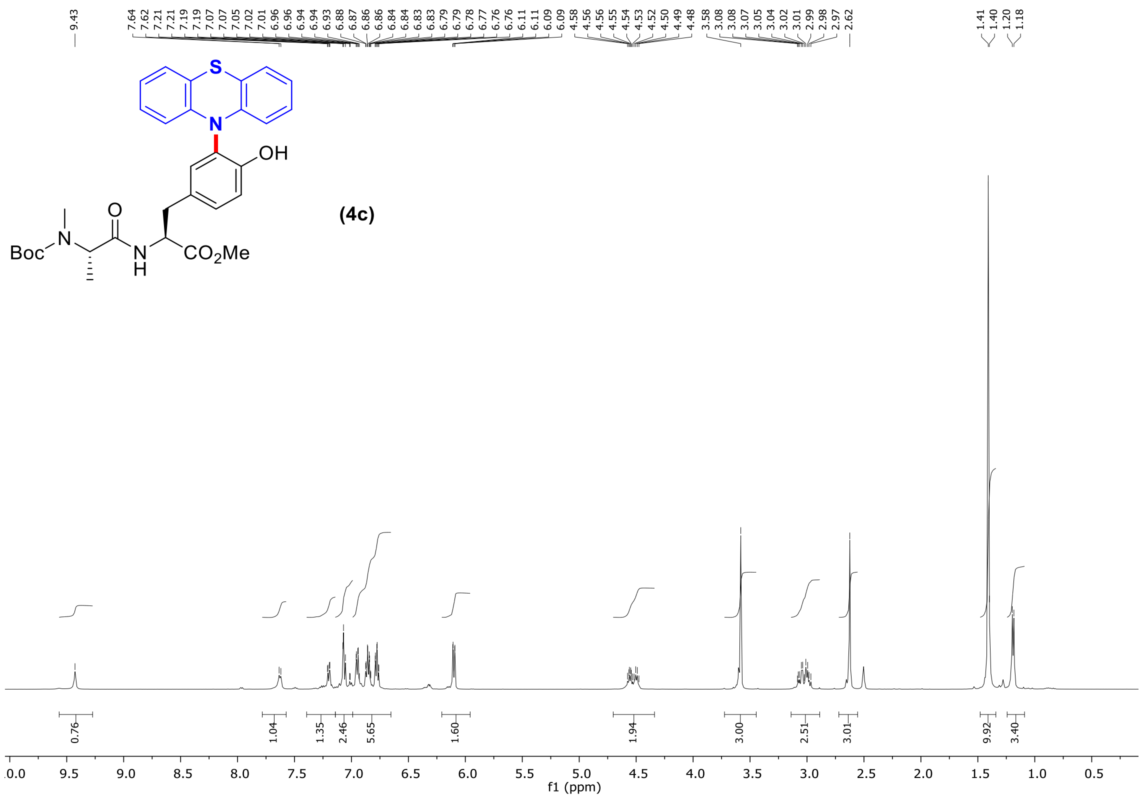

^{13}C NMR (126 MHz, DMSO-d_6 at 80 ºC)

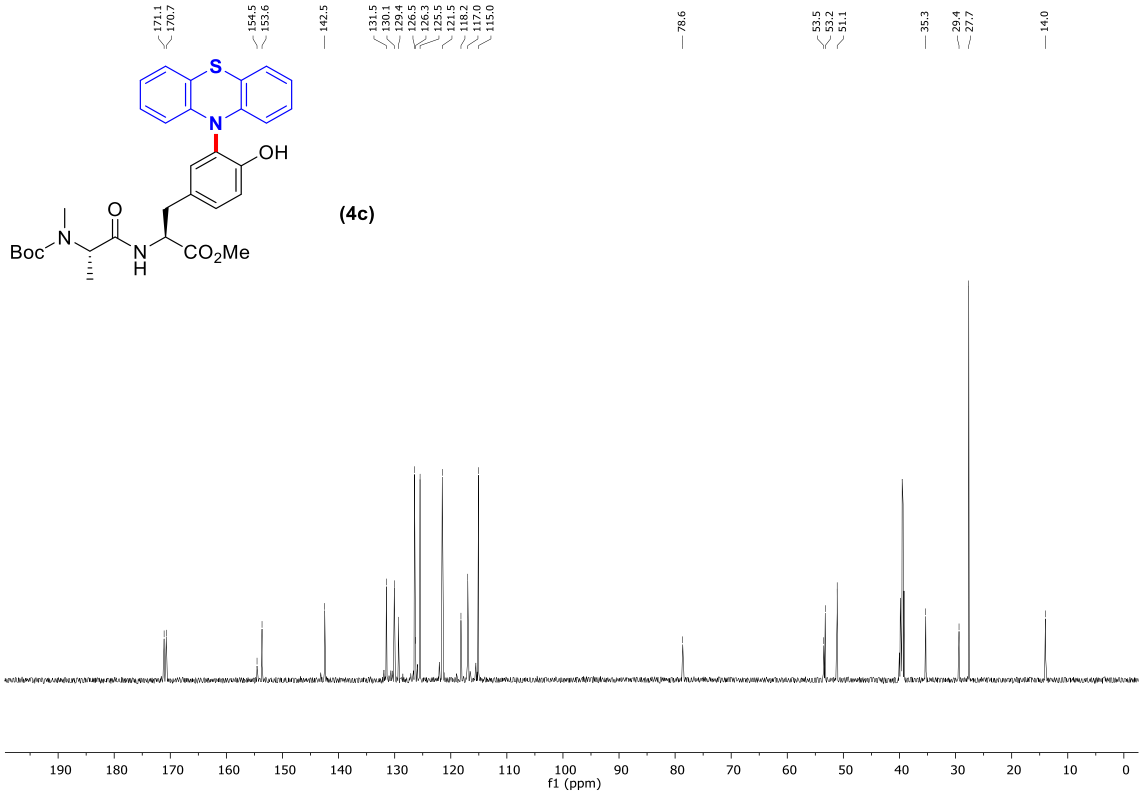

^{1}H NMR (500 MHz, DMSO-d_6 at 80 ºC)

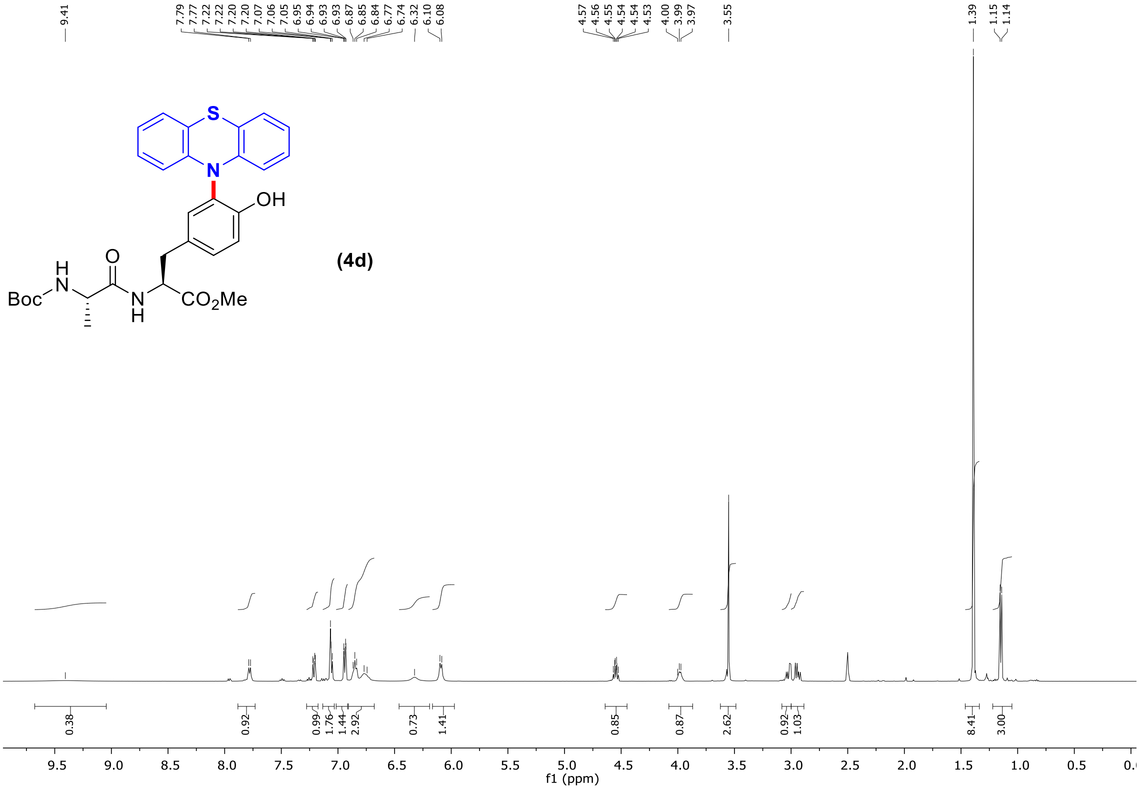

^{13}C NMR (126 MHz, DMSO-d_6 at 80 ºC)

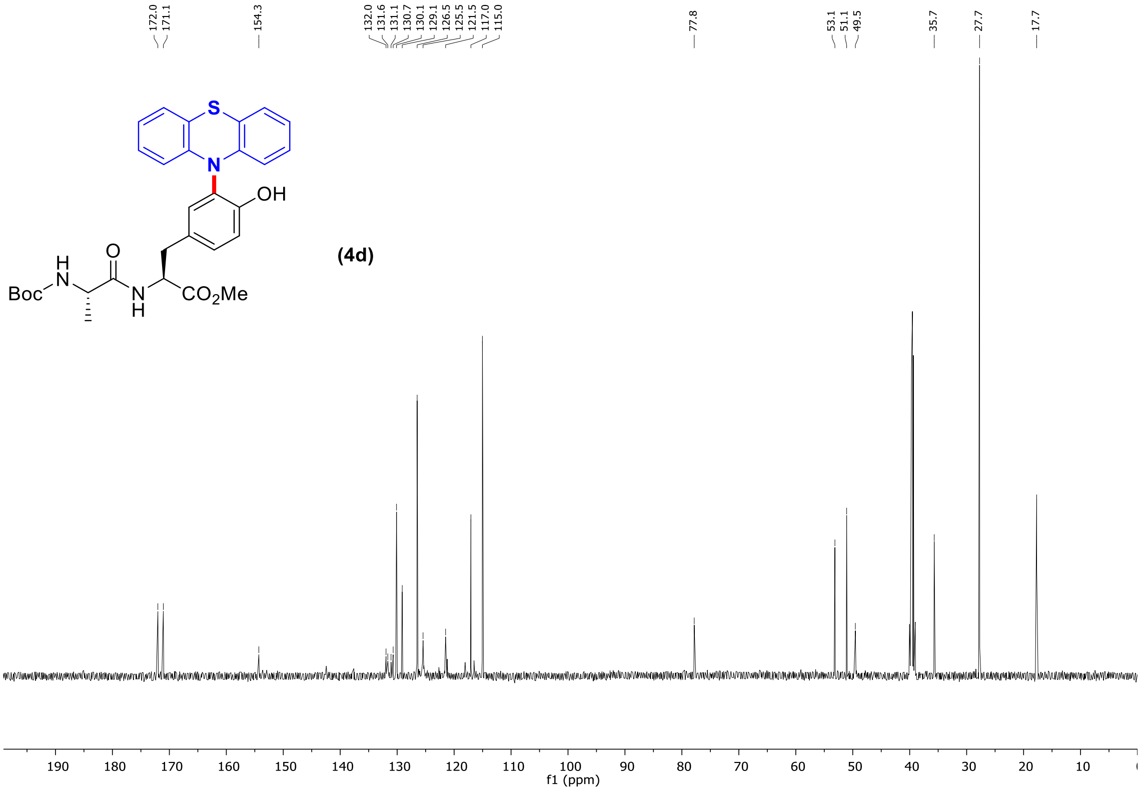

^{1}H NMR (500 MHz, DMSO-d_6 at 80 ºC)

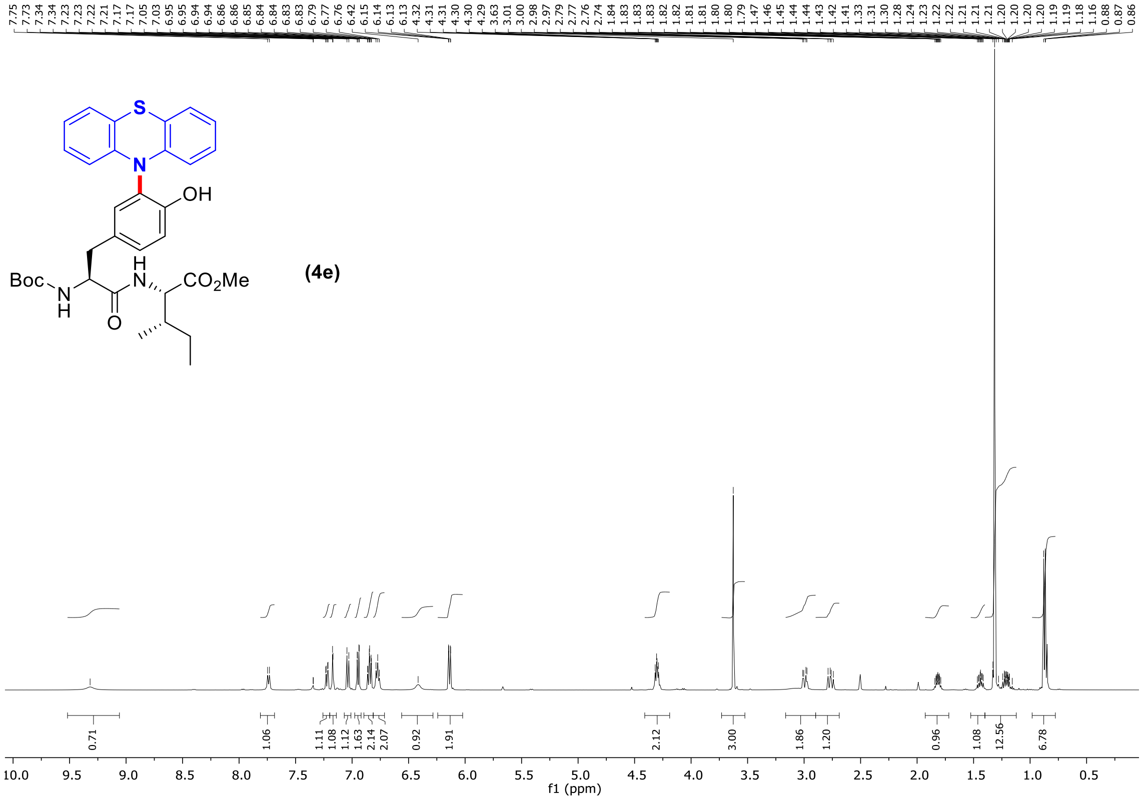

^{13}C NMR (126 MHz, DMSO-d_6 at 80 ºC)

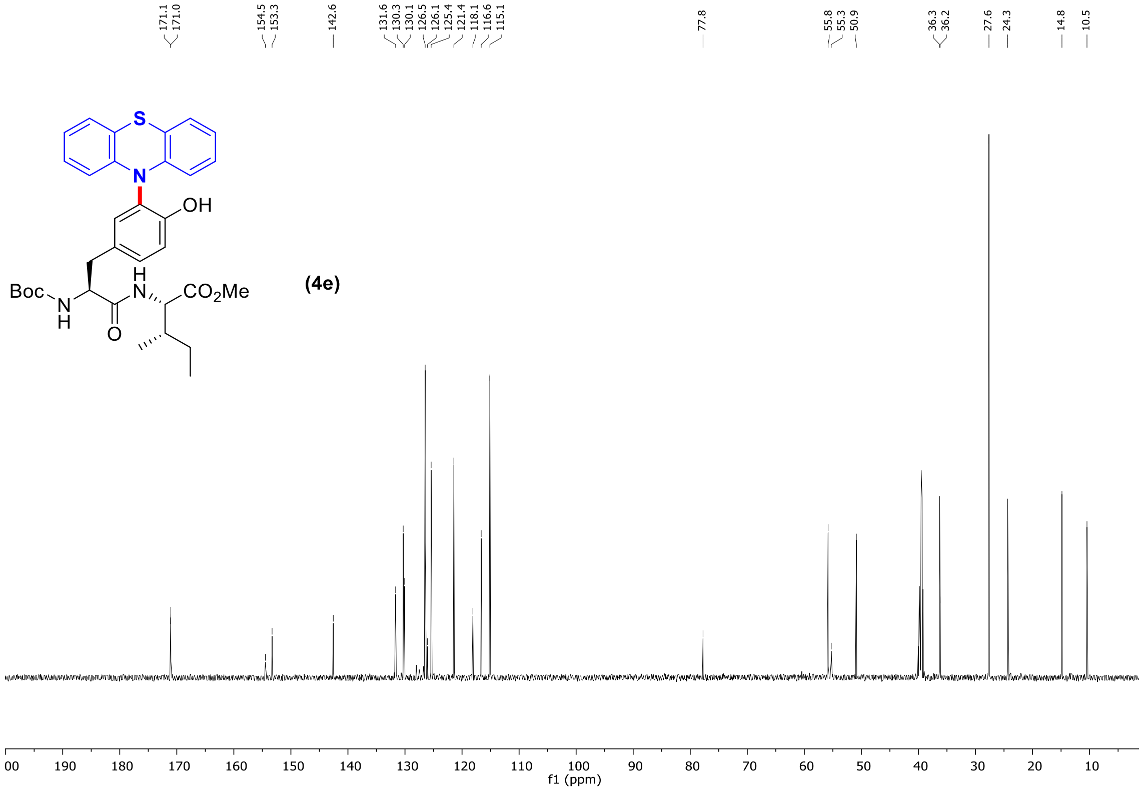

^{1}H NMR (500 MHz, DMSO-d_6 at 80 ºC)

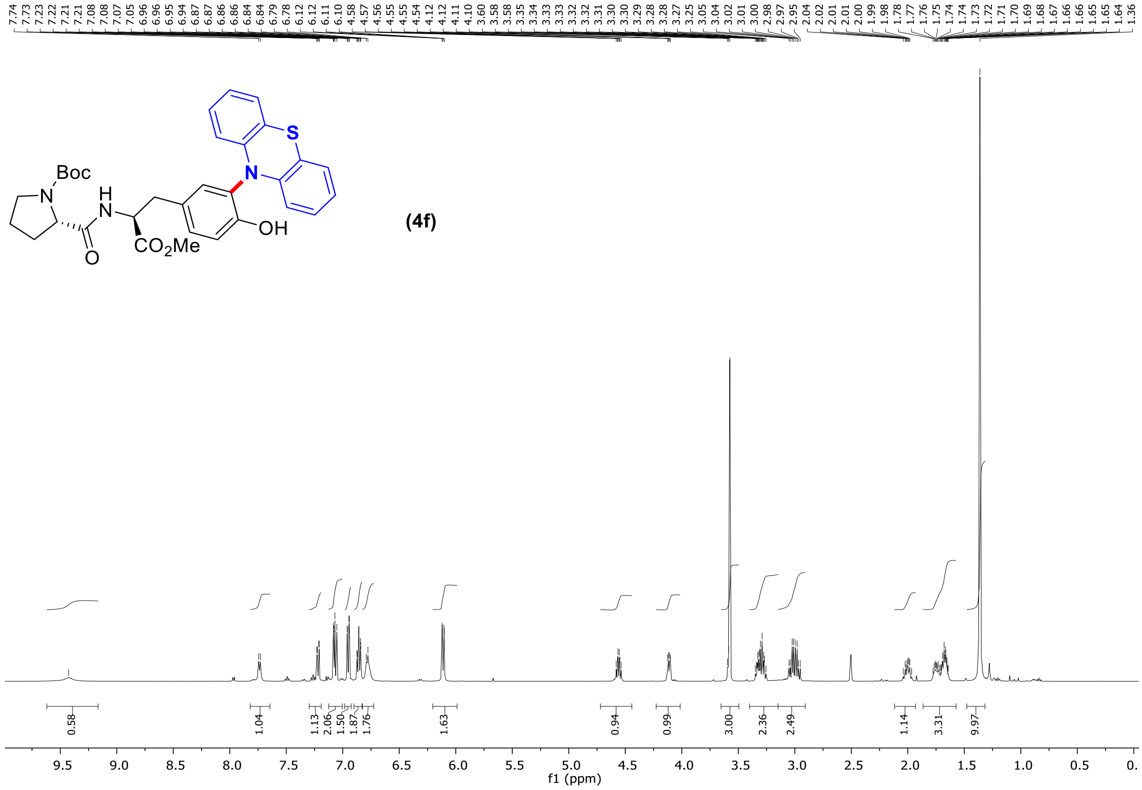

^{13}C NMR (126 MHz, DMSO-d_6 at 80 ºC)

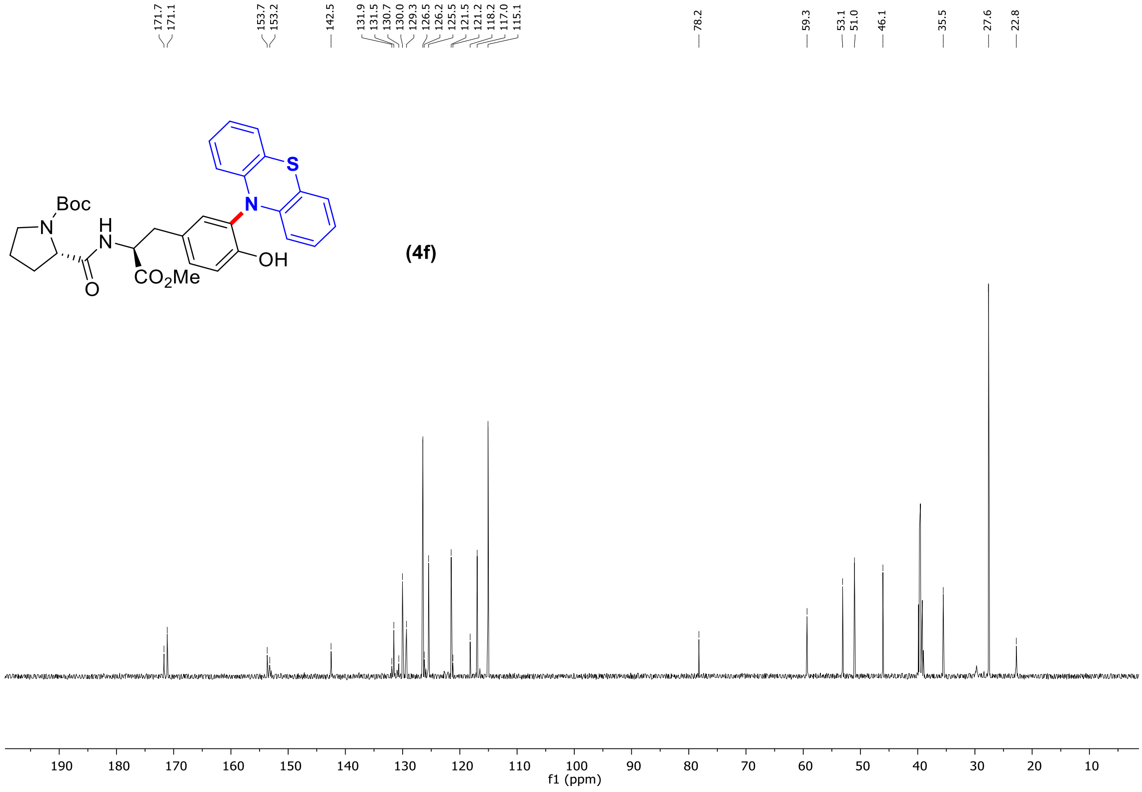

^{1}H NMR (500 MHz, DMSO-d_6 at 80 ºC)

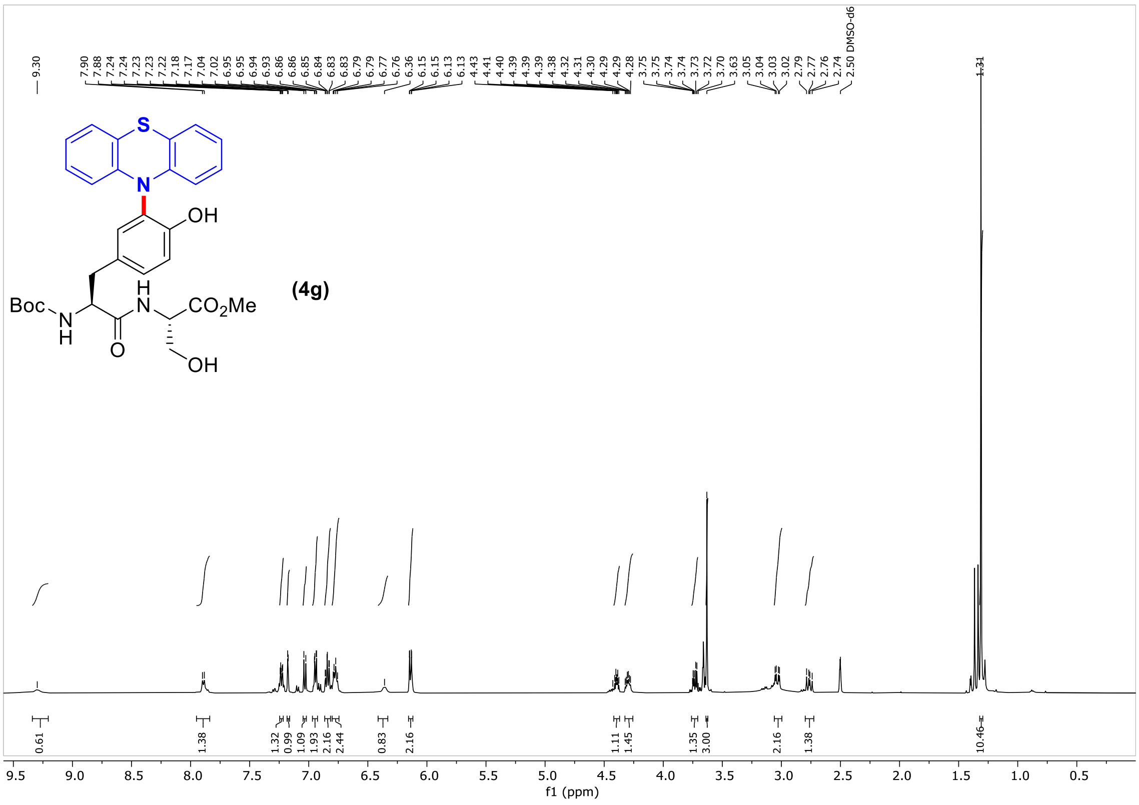

^{13}C NMR (126 MHz, DMSO-d_6 at 80 ºC)

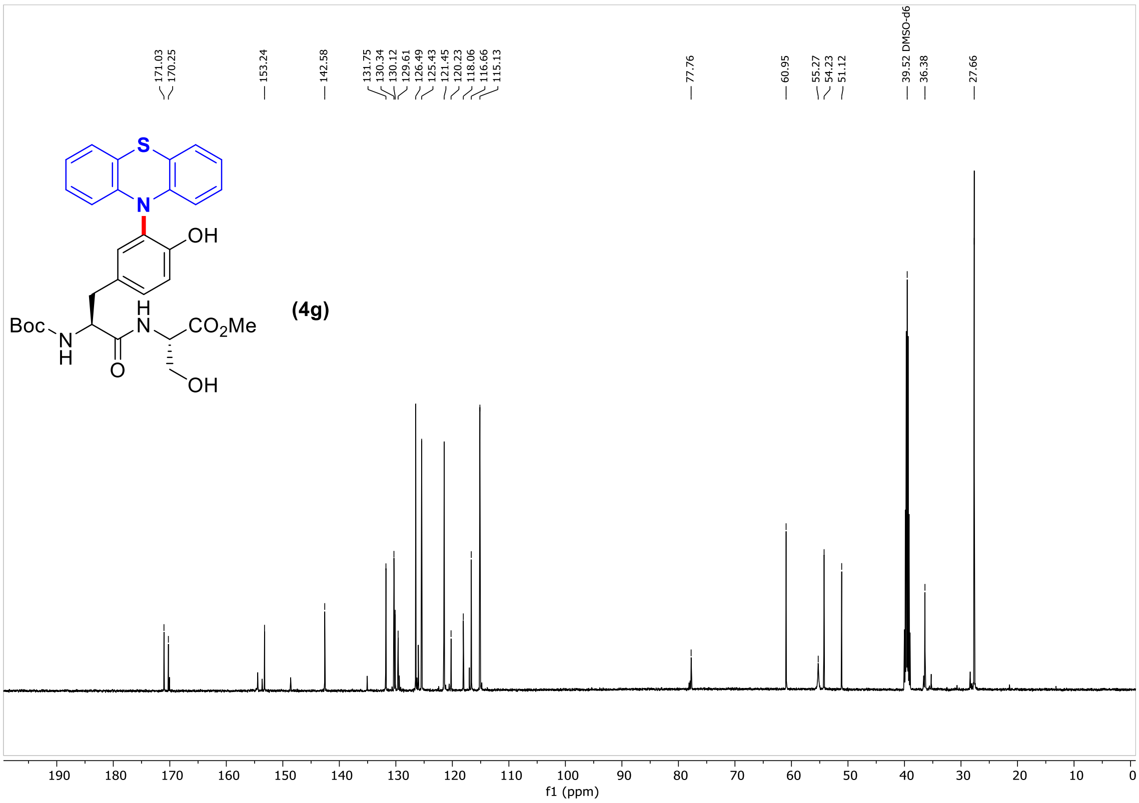

^{1}H NMR (500 MHz, DMSO-d_6 at 80 ºC)

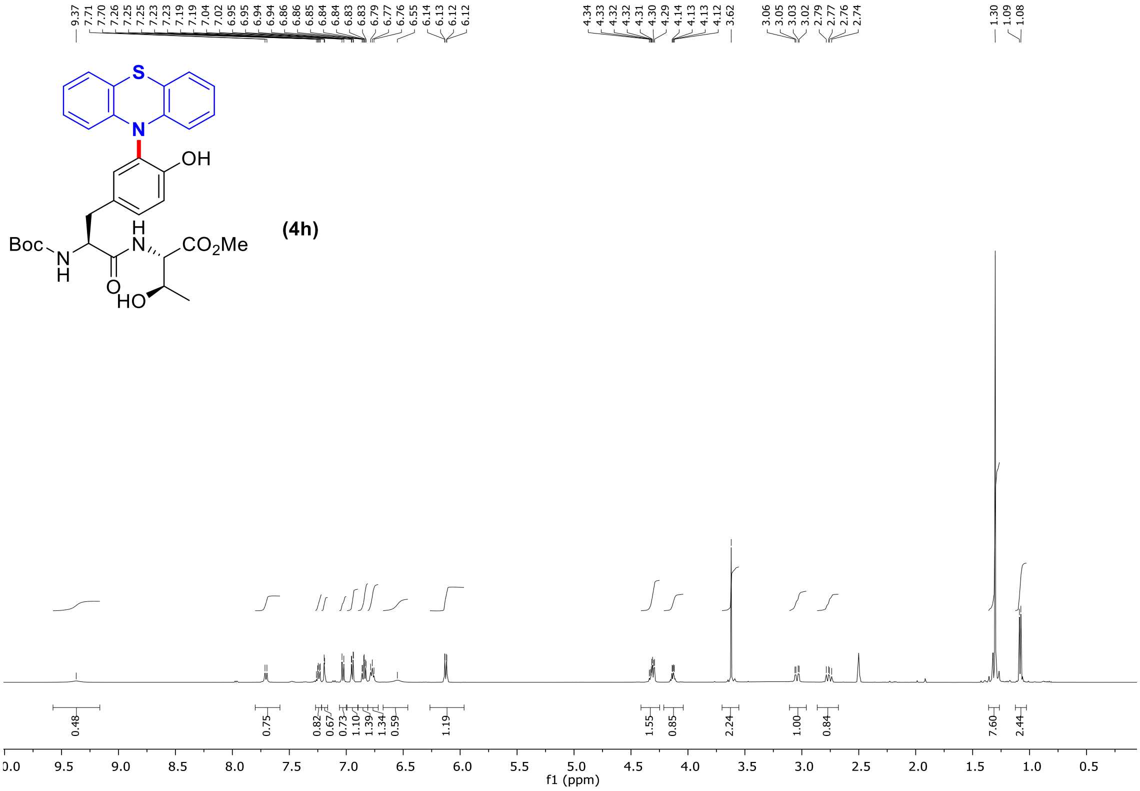

^{13}C NMR (126 MHz, DMSO-d_6 at 80 ºC)

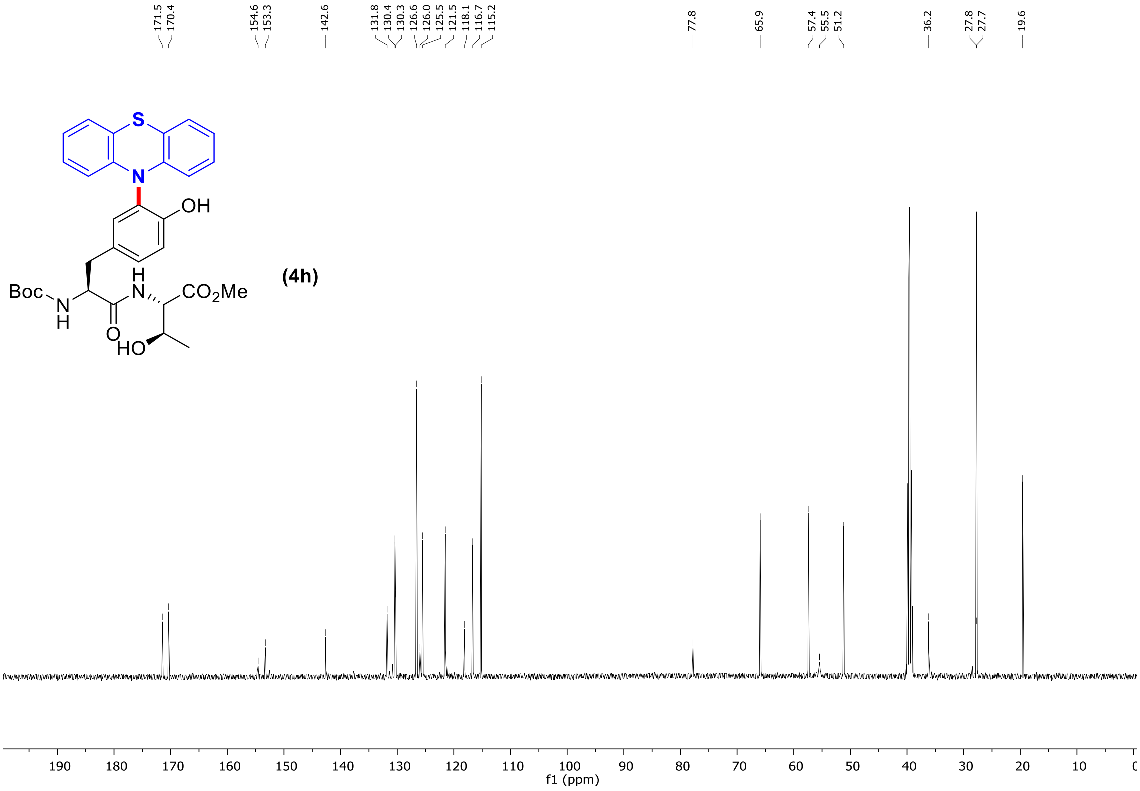

^{1}H NMR (500 MHz, DMSO-d_6 at 80 ºC)

(4i)

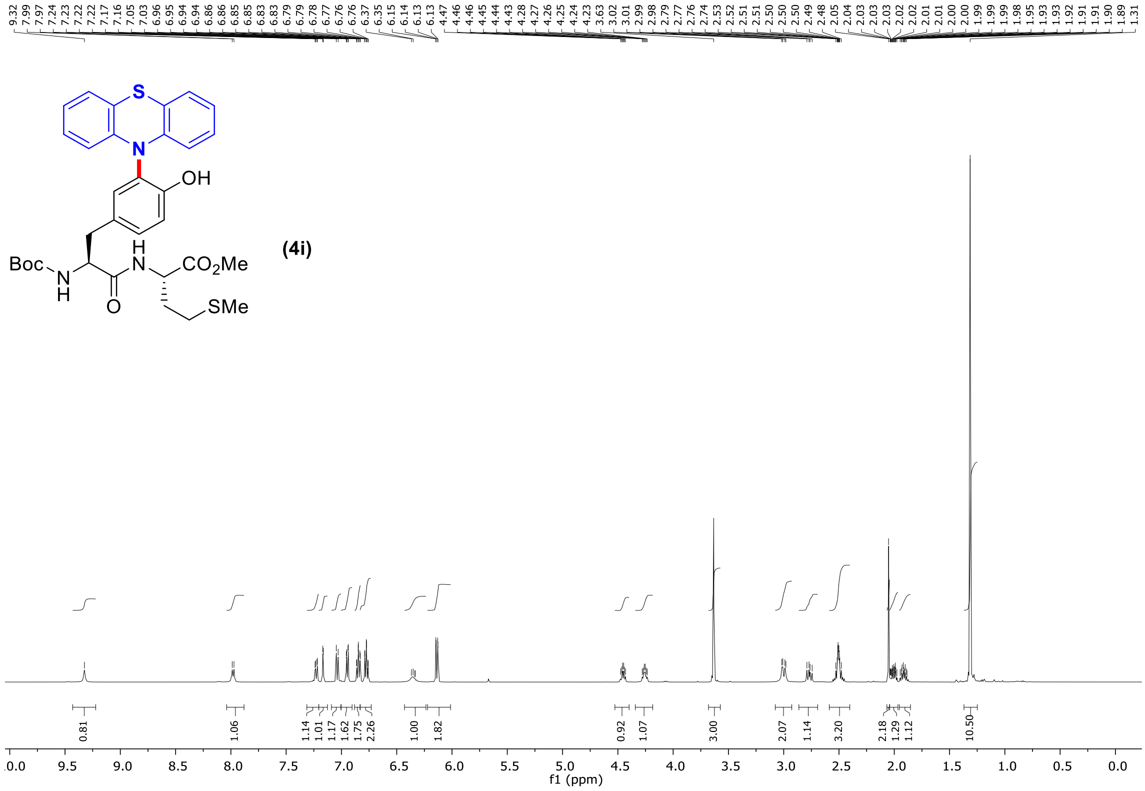

^{13}C NMR (126 MHz, DMSO-d_6 at 80 ºC)

(4i)

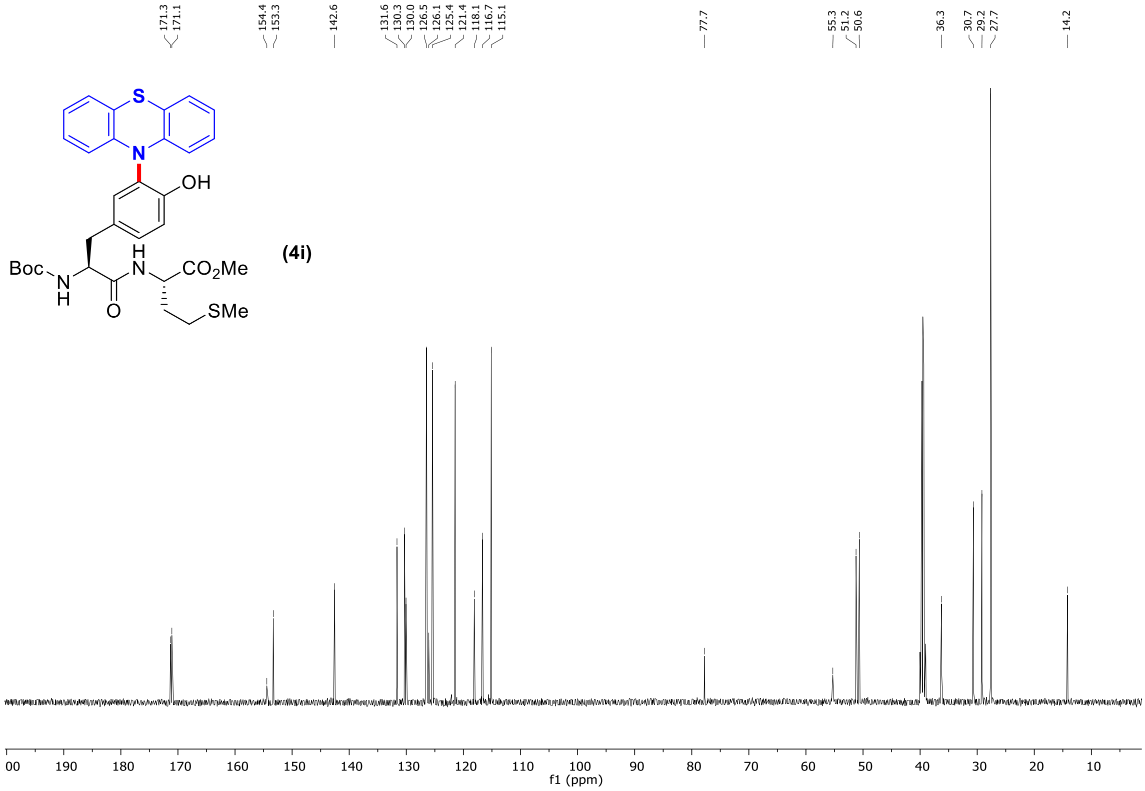

^{1}H NMR (500 MHz, DMSO-d_6 at 80 ºC)

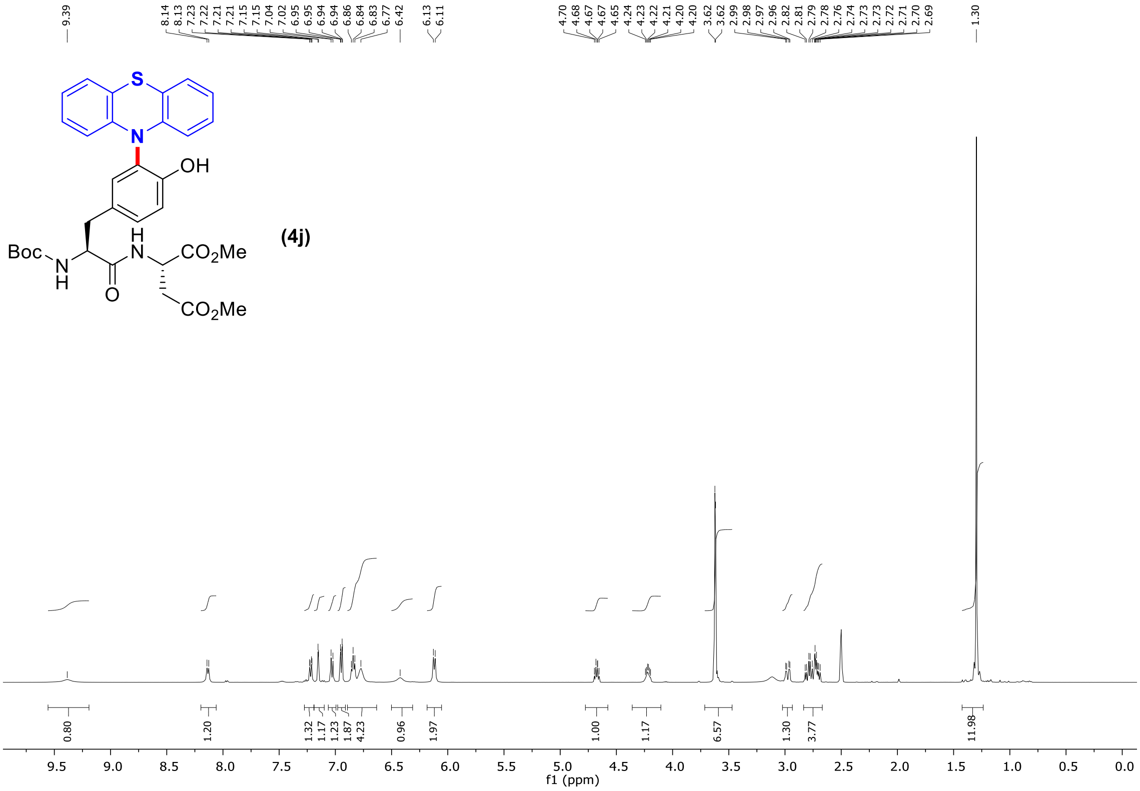

^{13}C NMR (126 MHz, DMSO-d_6 at 80 ºC)

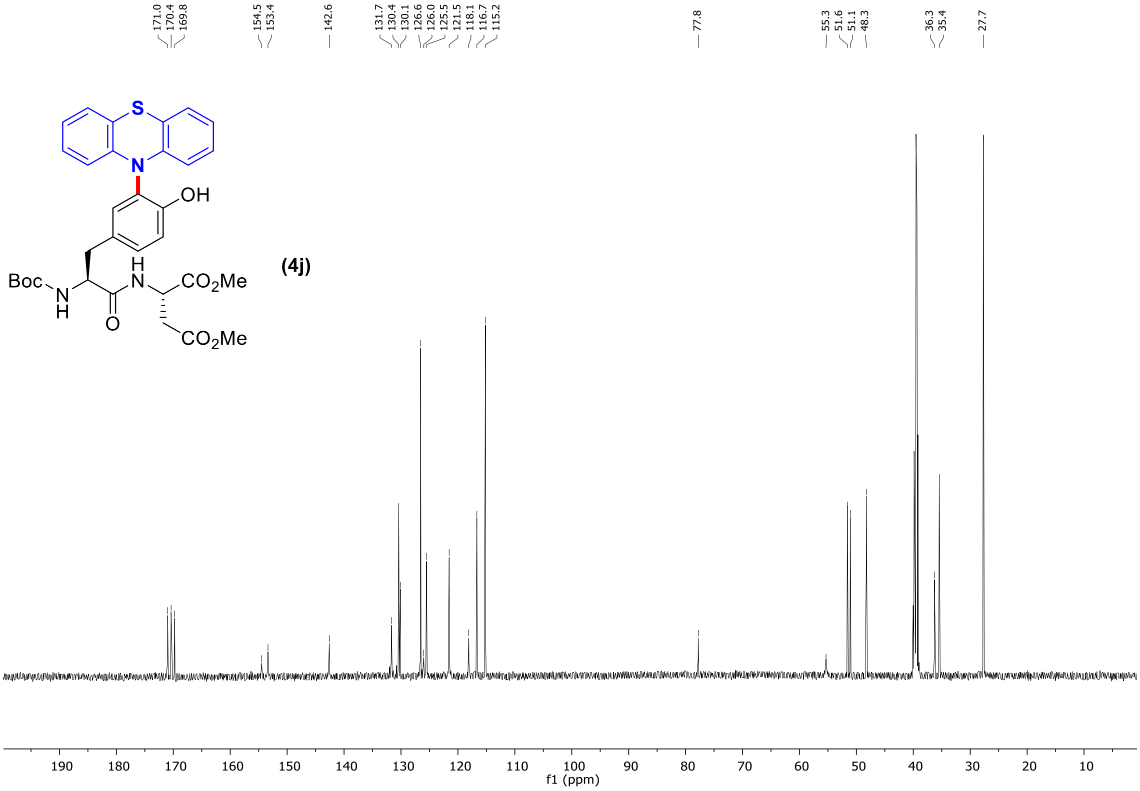

^{1}H NMR (500 MHz, DMSO-d_6 at 80 ºC)

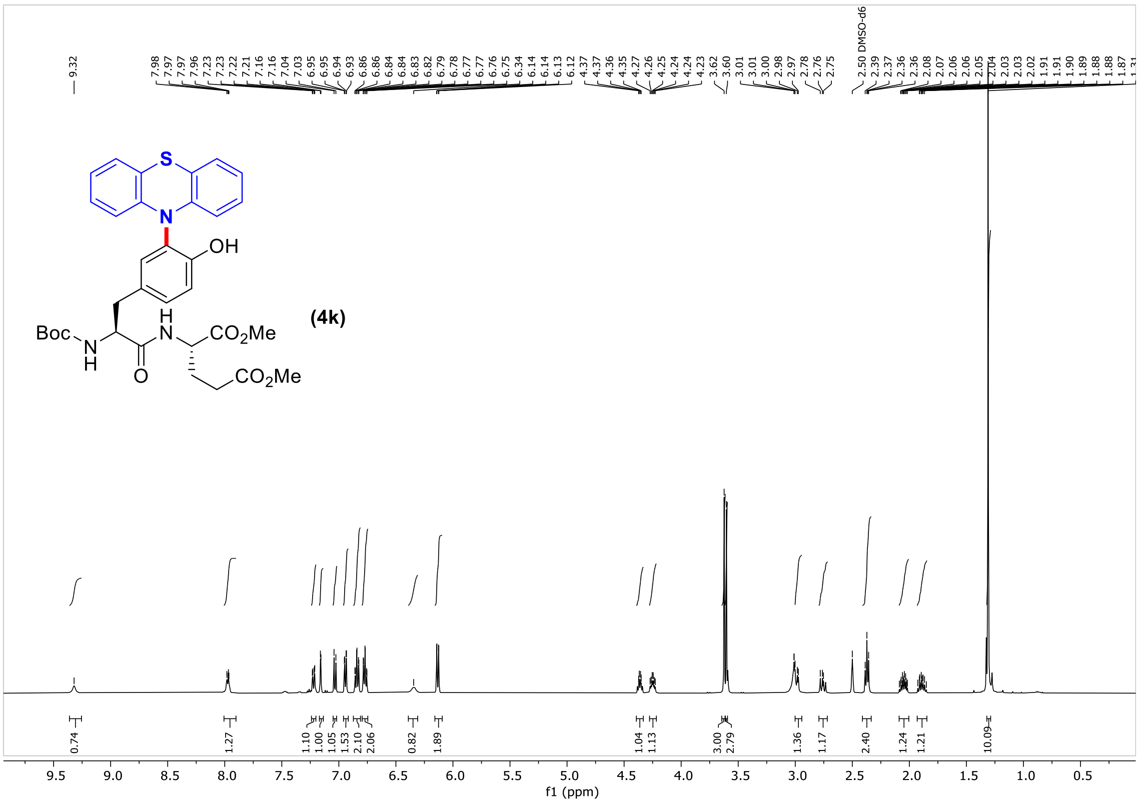

^{13}C NMR (126 MHz, DMSO-d_6 at 80 ºC)

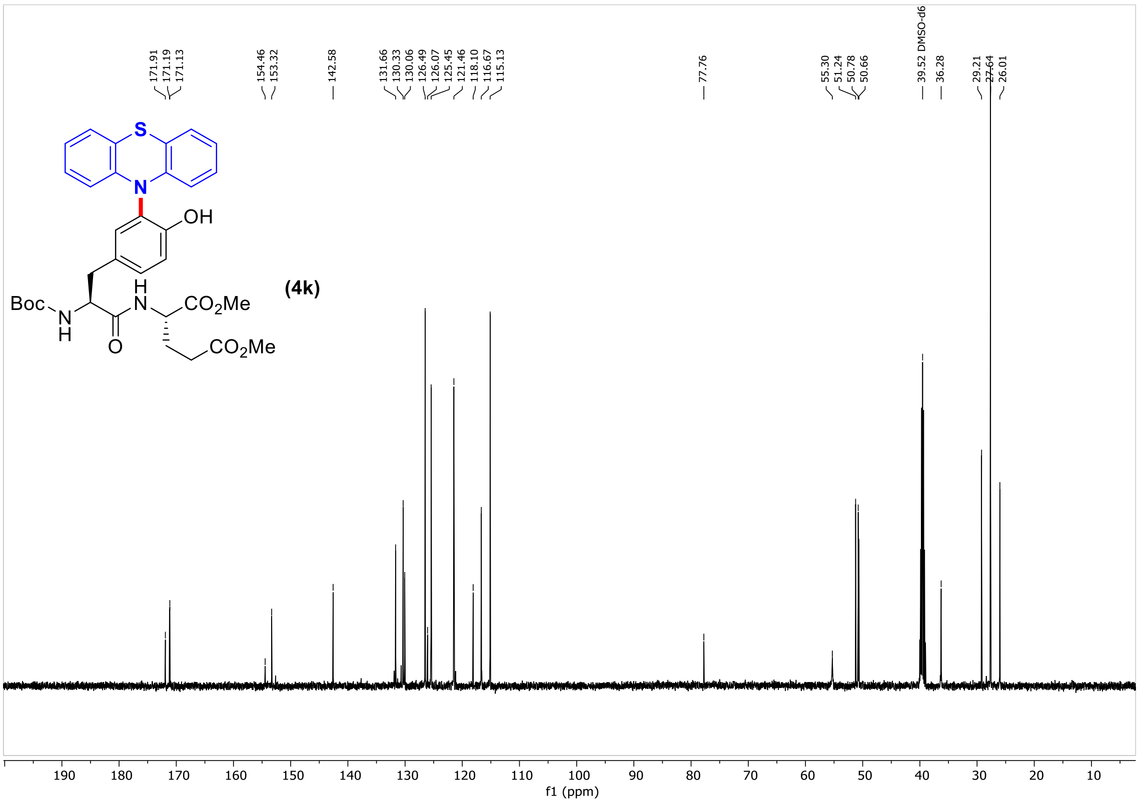

^1H NMR (500 MHz, DMSO-d_6 at 80 ºC)

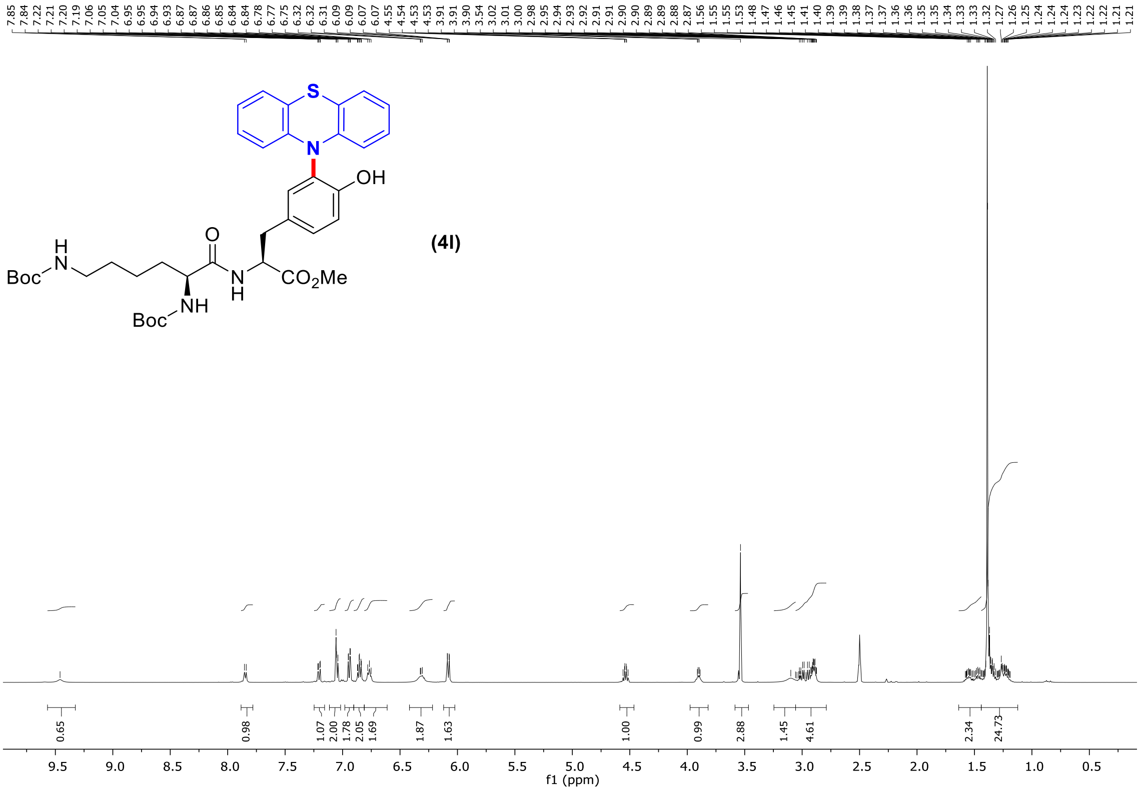

^{13}C NMR (126 MHz, DMSO-d_6 at 80 ºC)

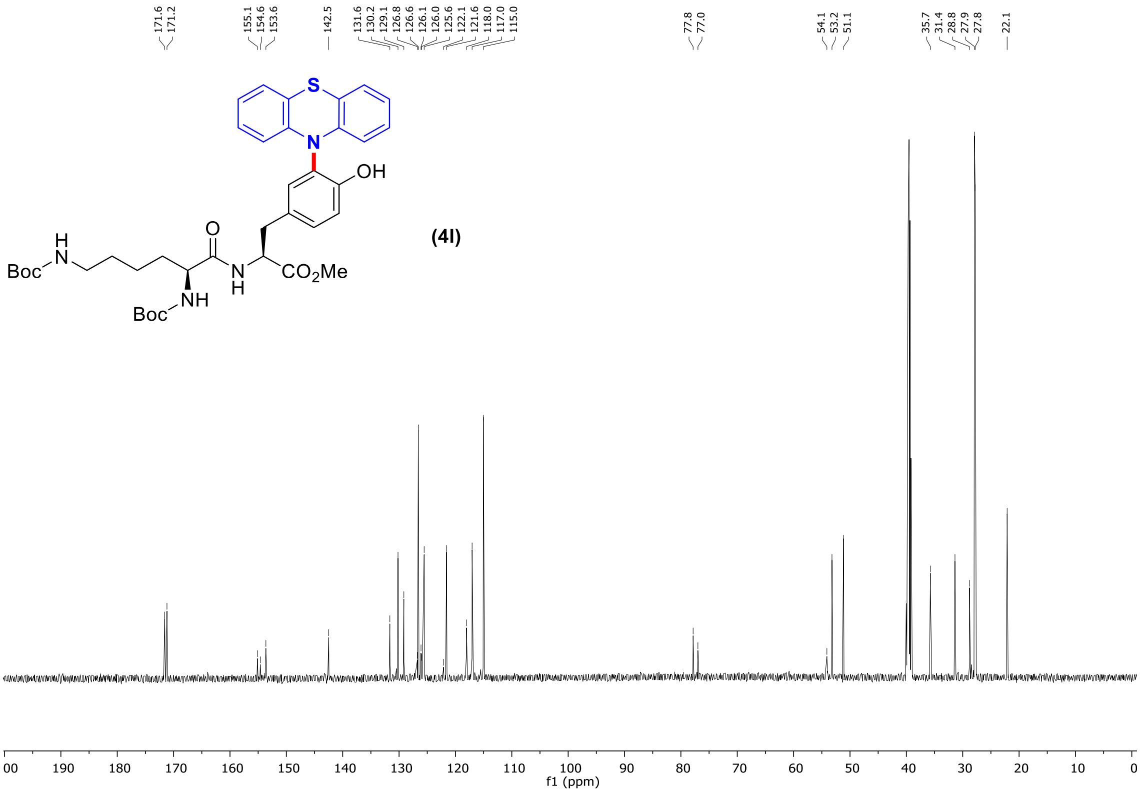

^{1}H NMR (500 MHz, DMSO-d_6 at 80 ºC)

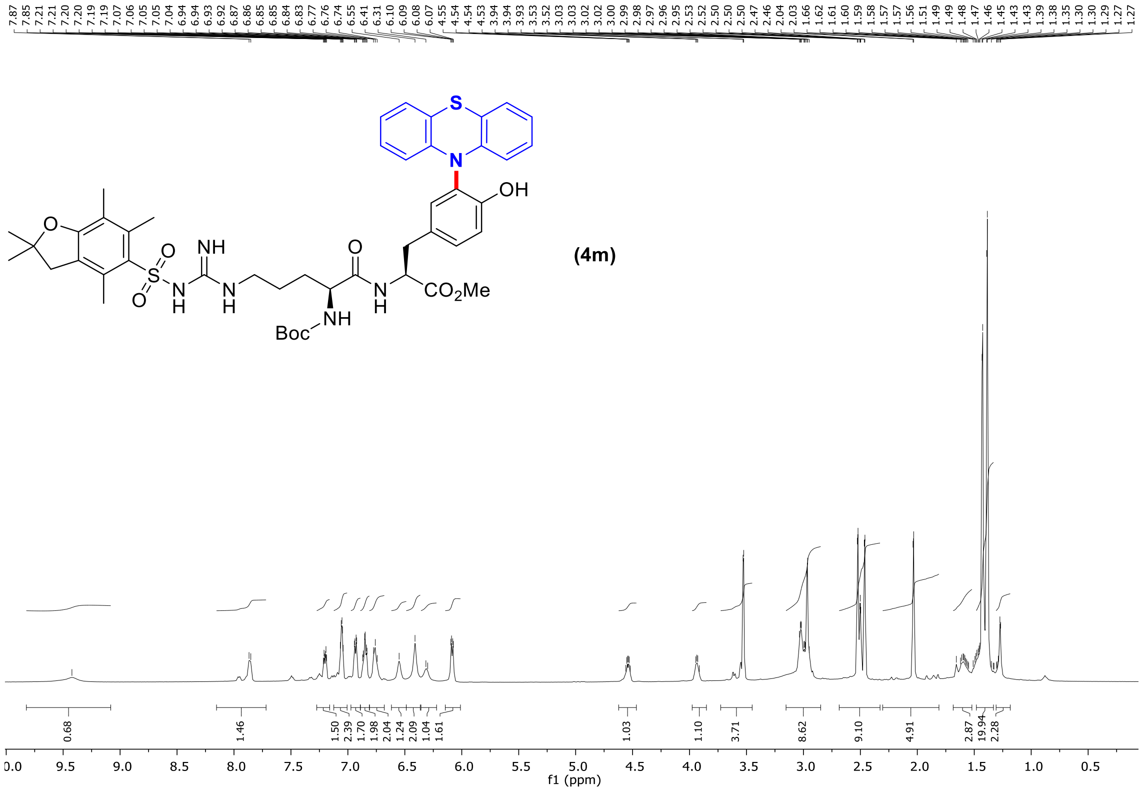

^{13}C NMR (126 MHz, DMSO-d_6 at 80 ºC)

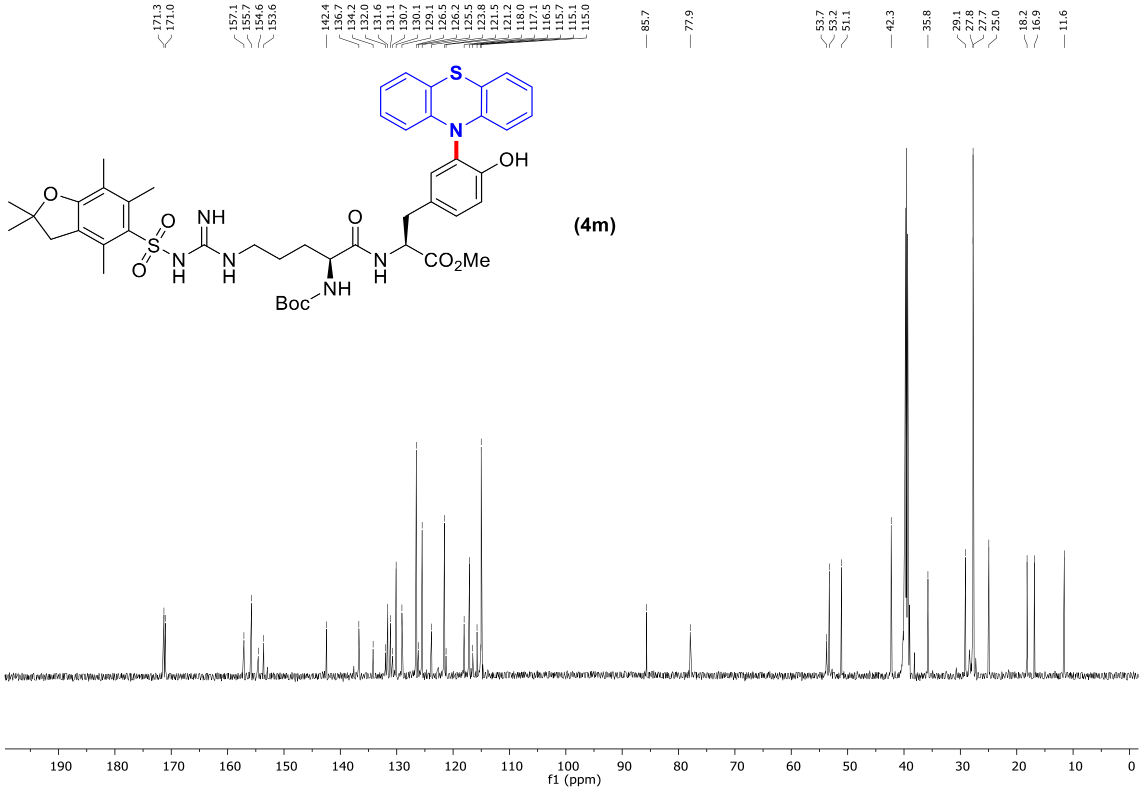

^{1}H NMR (500 MHz, DMSO-d_6 at 80 ºC)

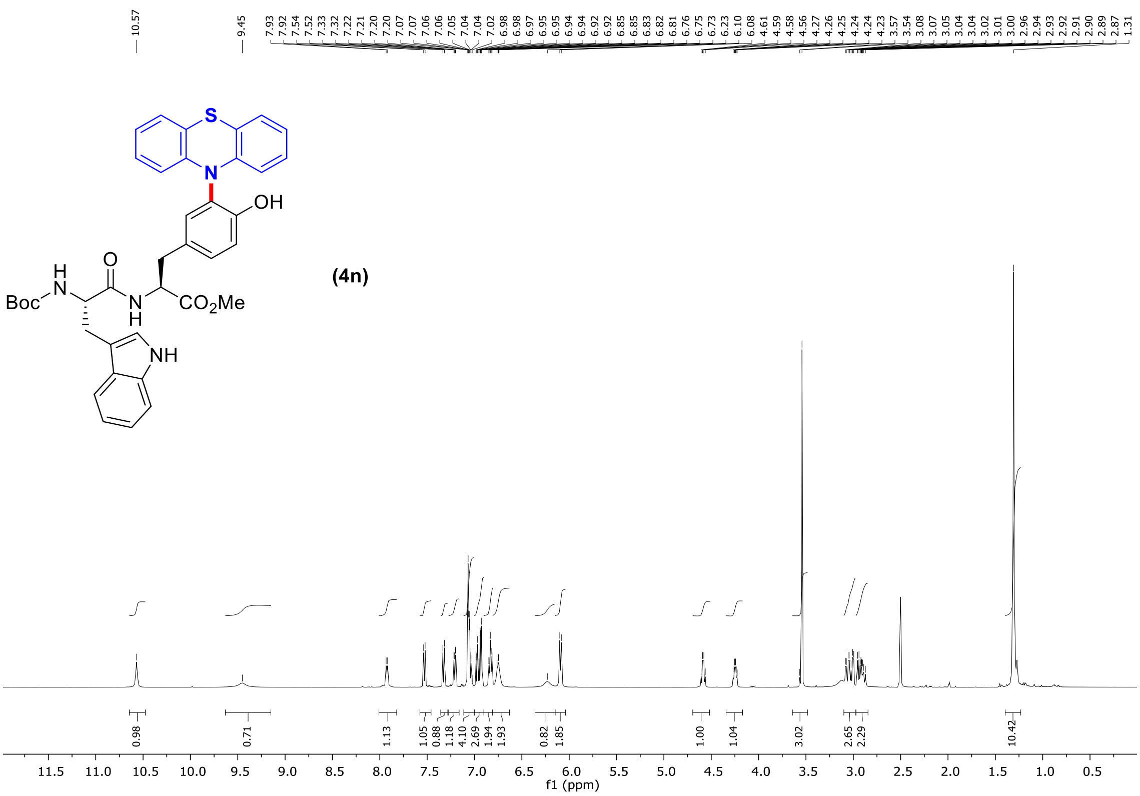

^{13}C NMR (126 MHz, DMSO-d_6 at 80 ºC)

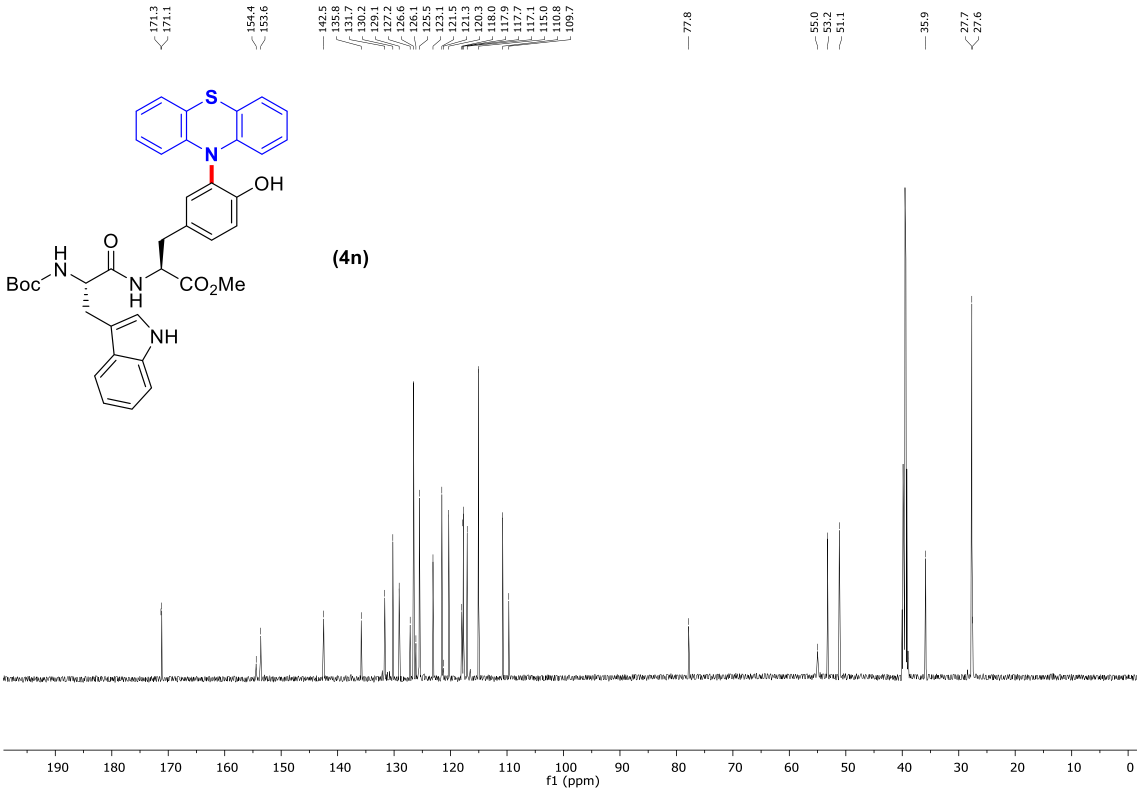

^{1}H NMR (500 MHz, DMSO-d_6 at 80 ºC)

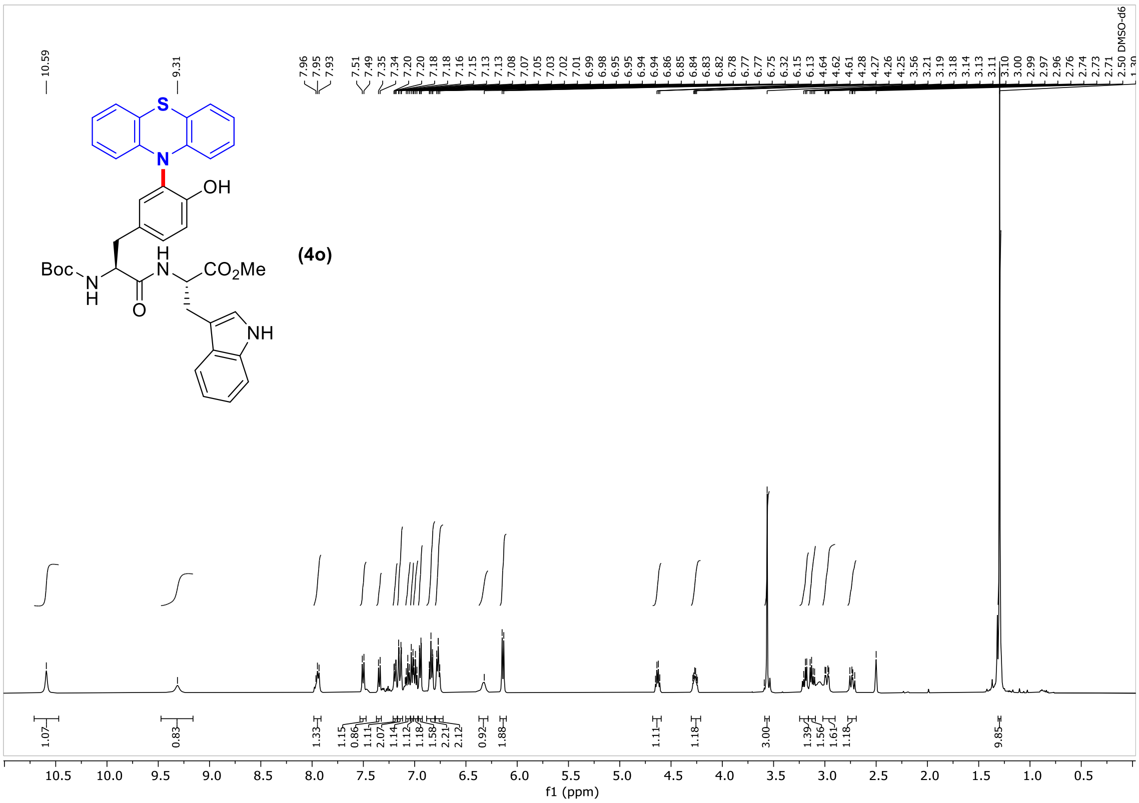

^{13}C NMR (126 MHz, DMSO-d_6 at 80 ºC)

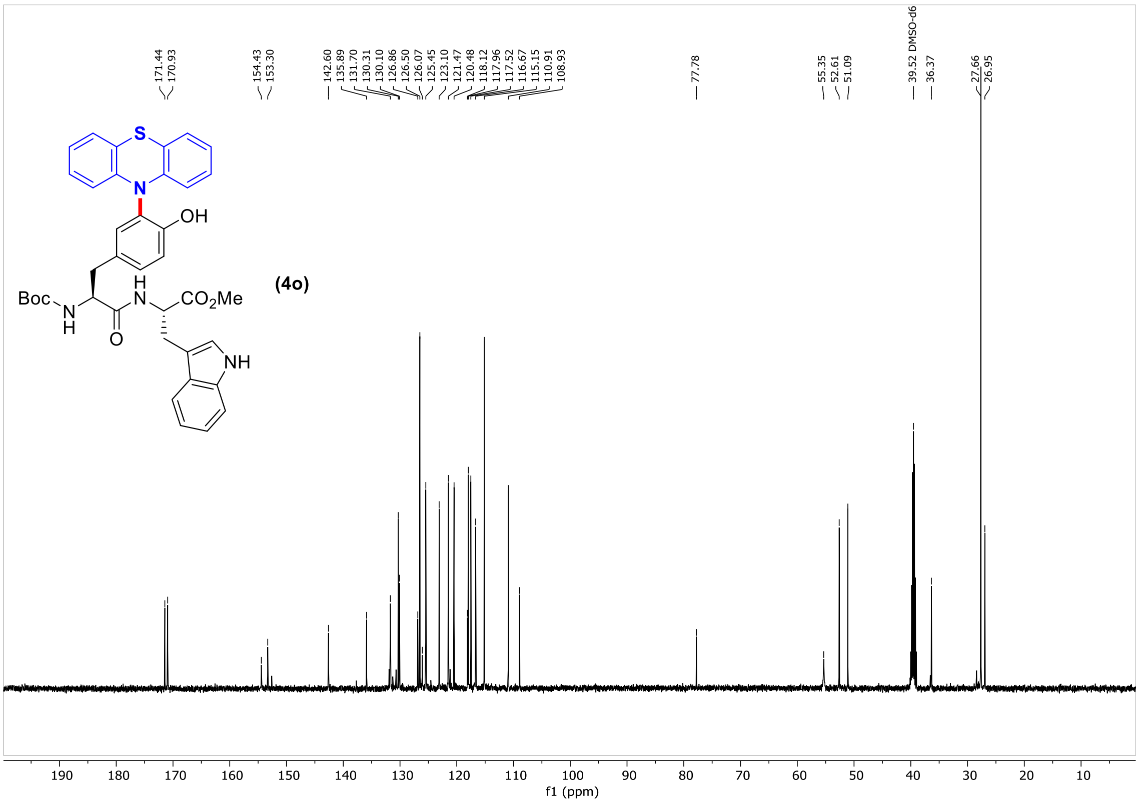

^{1}H NMR (500 MHz, DMSO-d_6 at 80 ºC)

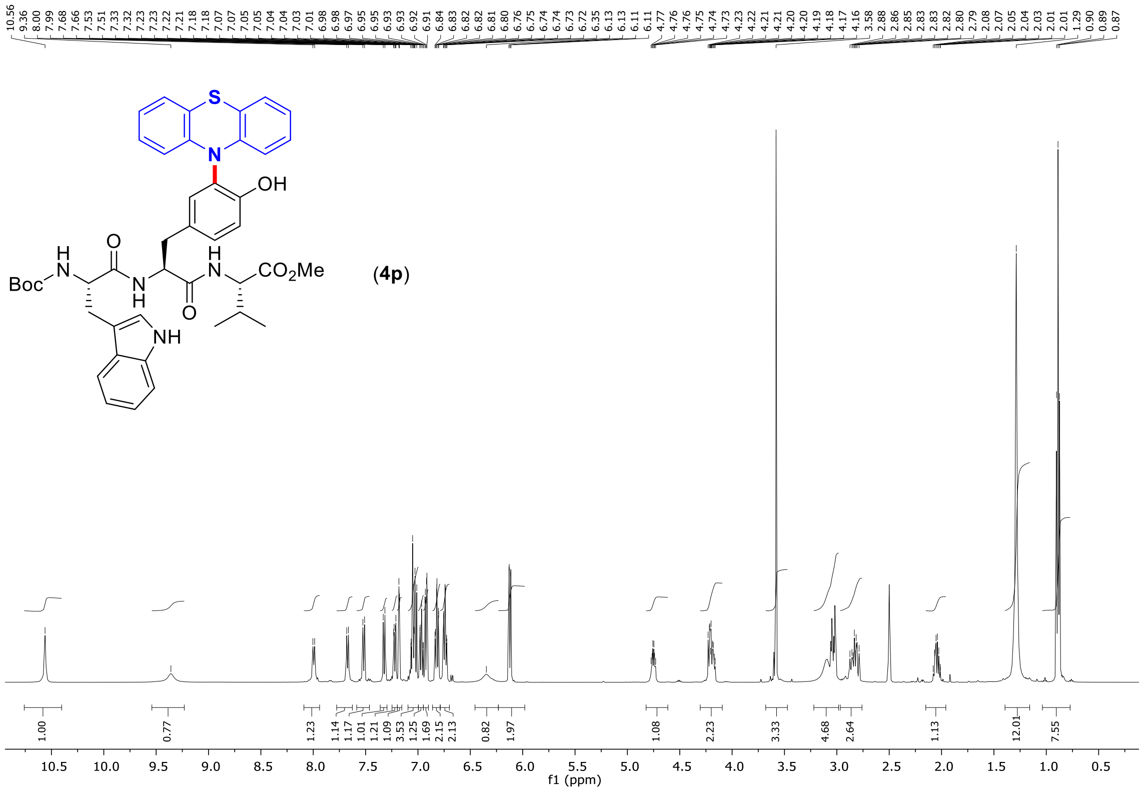

^{13}C NMR (126 MHz, DMSO-d_6 at 80 ºC)

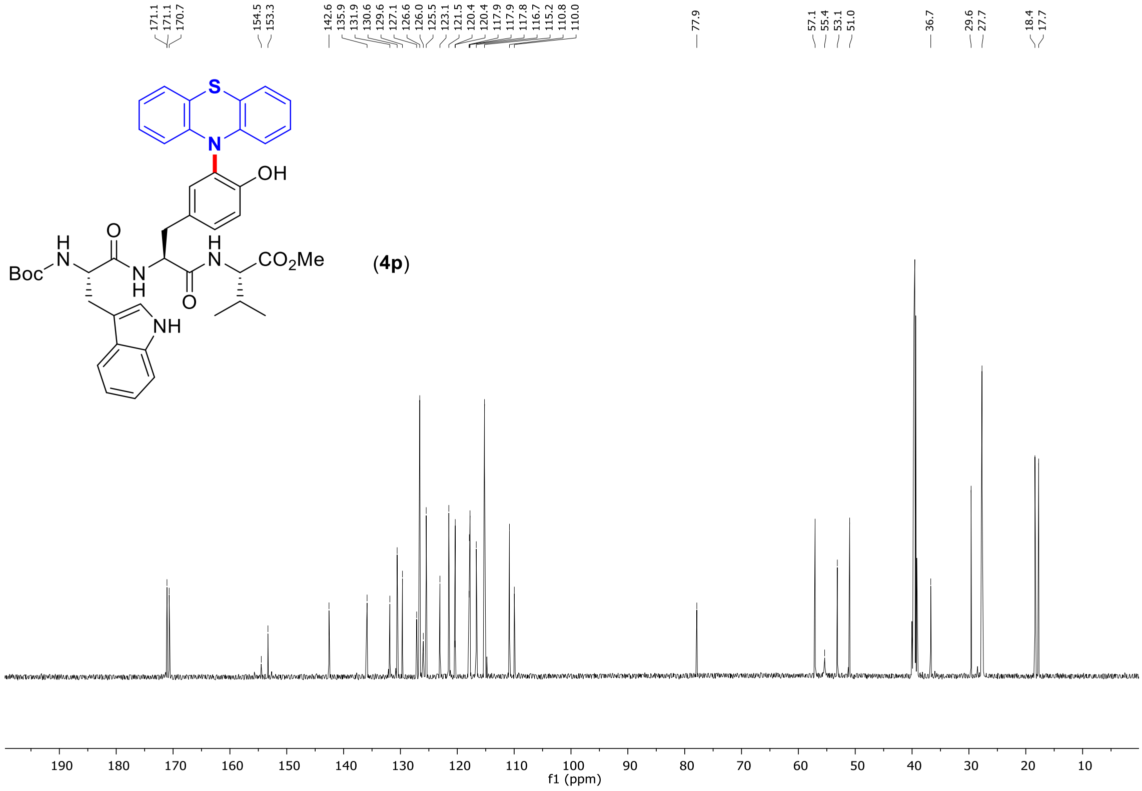

^{1}H NMR (500 MHz, DMSO-d_6 at 80 ºC)

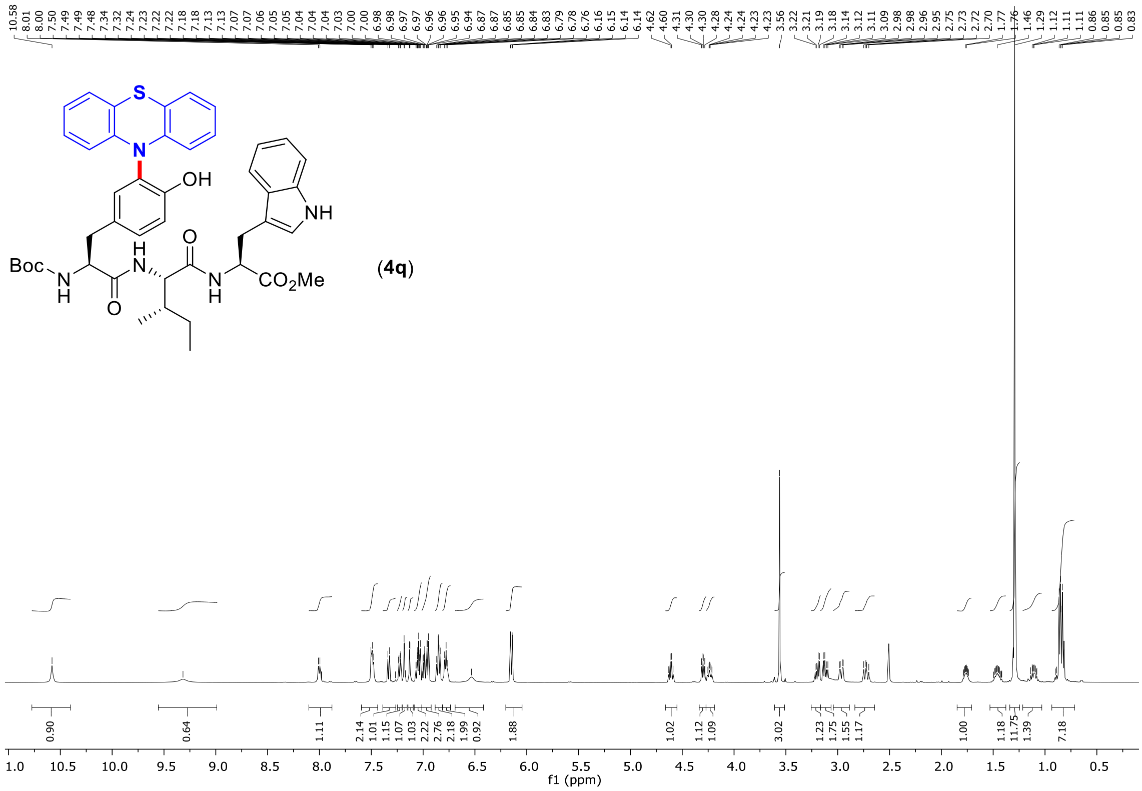

^{13}C NMR (126 MHz, DMSO-d_6 at 80 ºC)

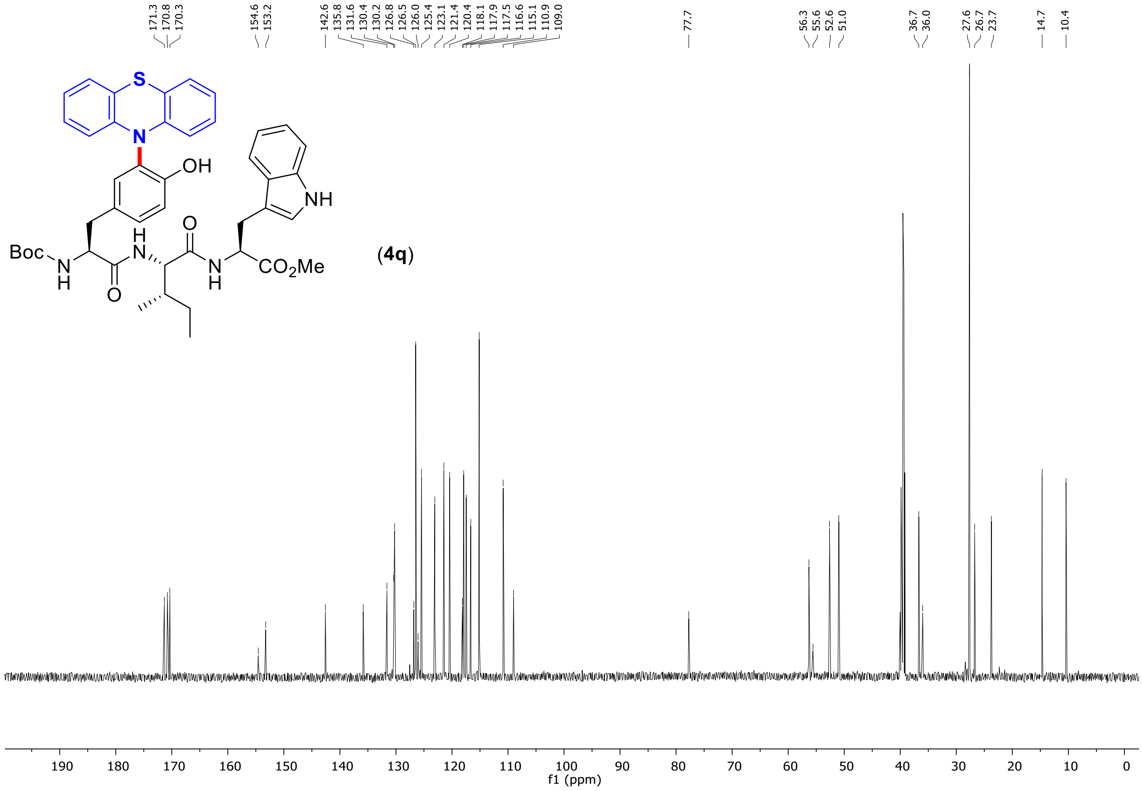

^{1}H NMR (500 MHz, DMSO-d_6 at 80 ºC)

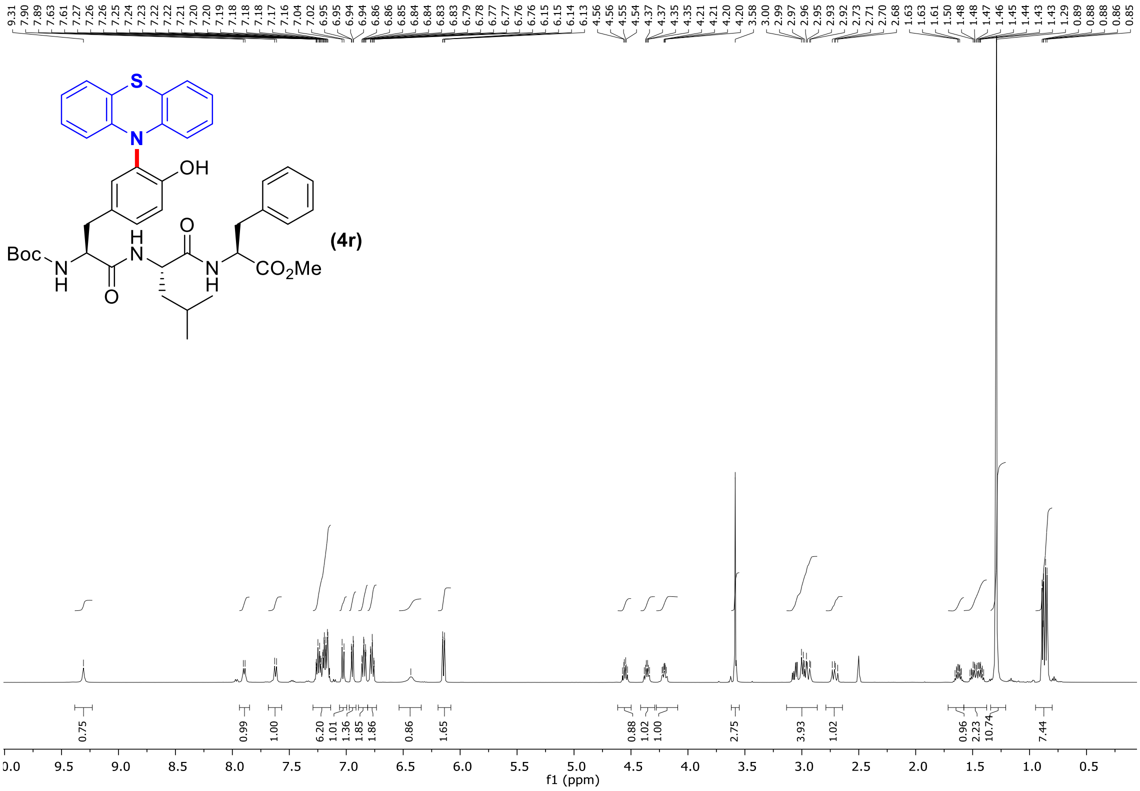

^{13}C NMR (126 MHz, DMSO-d_6 at 80 ºC)

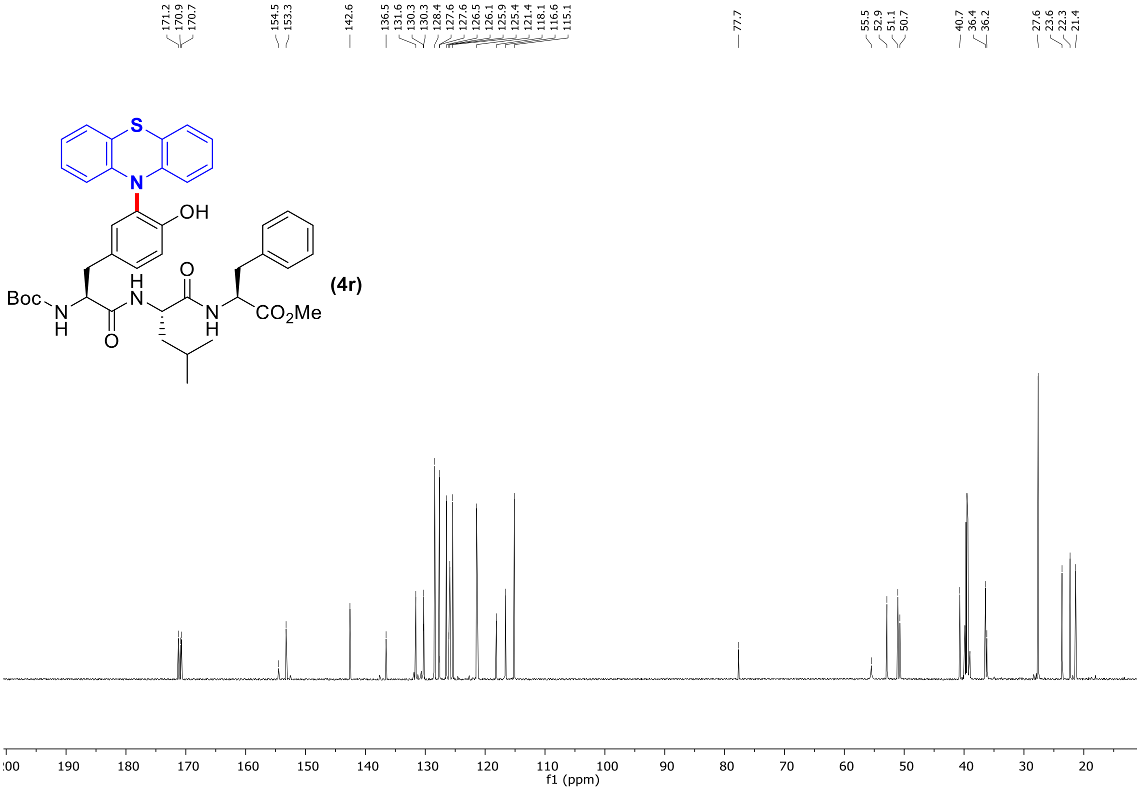

^{1}H NMR (500 MHz, DMSO-d_6 at 80 ºC)

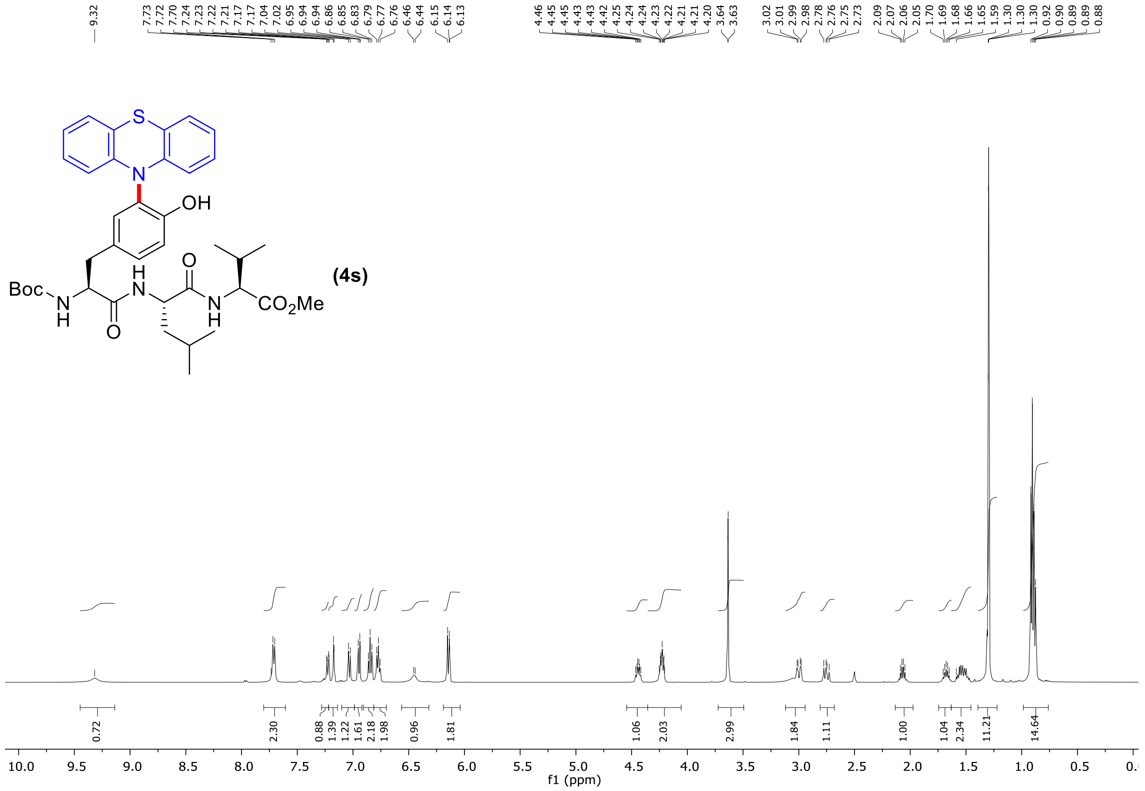

^{13}C NMR (126 MHz, DMSO-d_6 at 80 ºC)

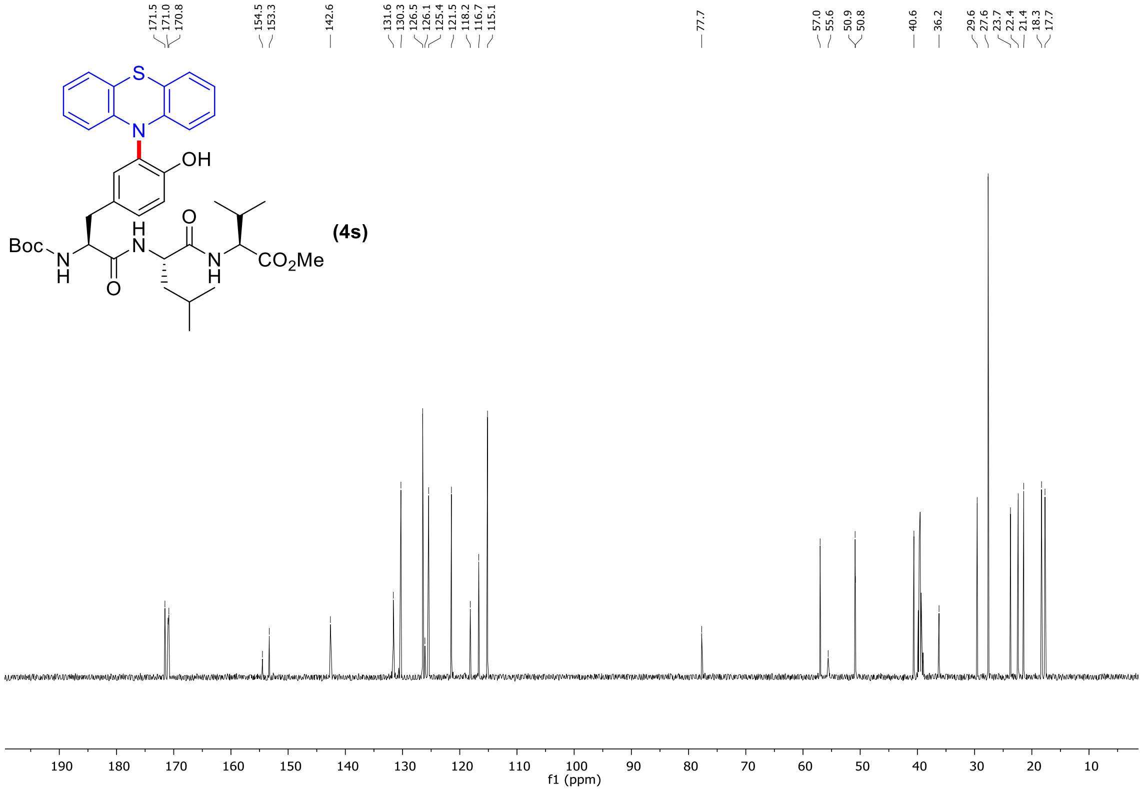

^{1}H NMR (500 MHz, DMSO-d_6 at 80 ºC)

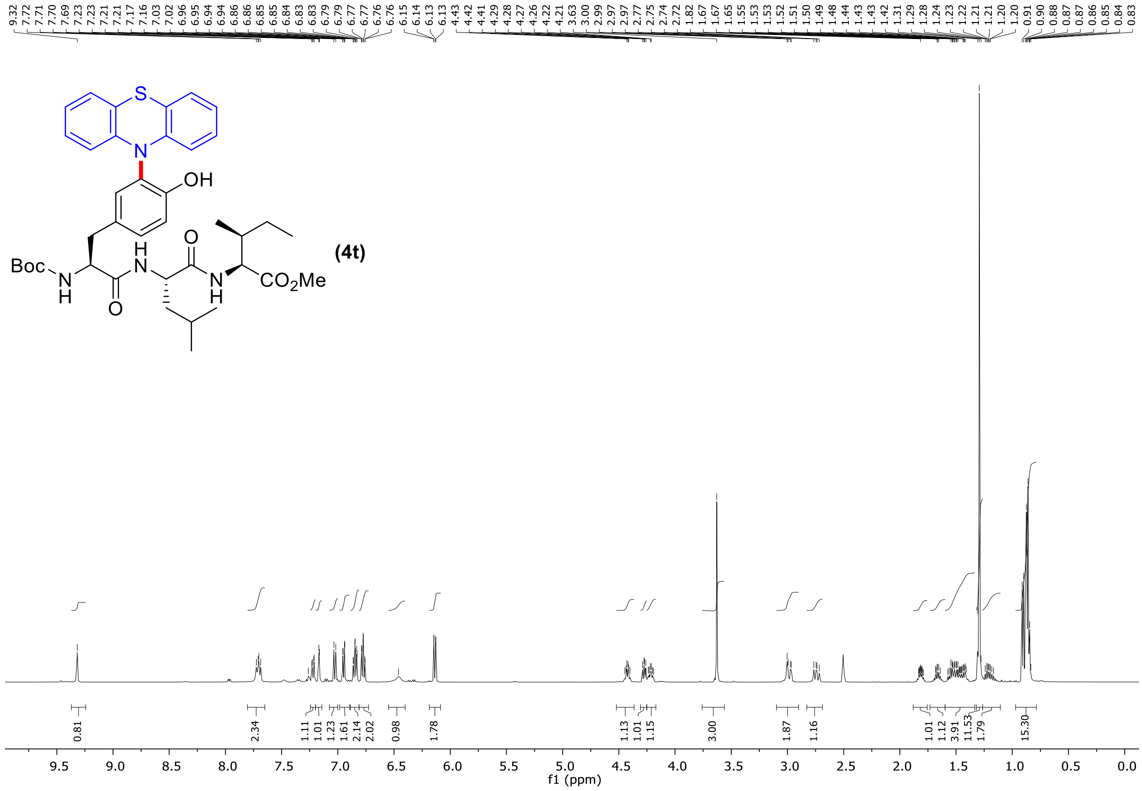

^{13}C NMR (126 MHz, DMSO-d_6 at 80 ºC)

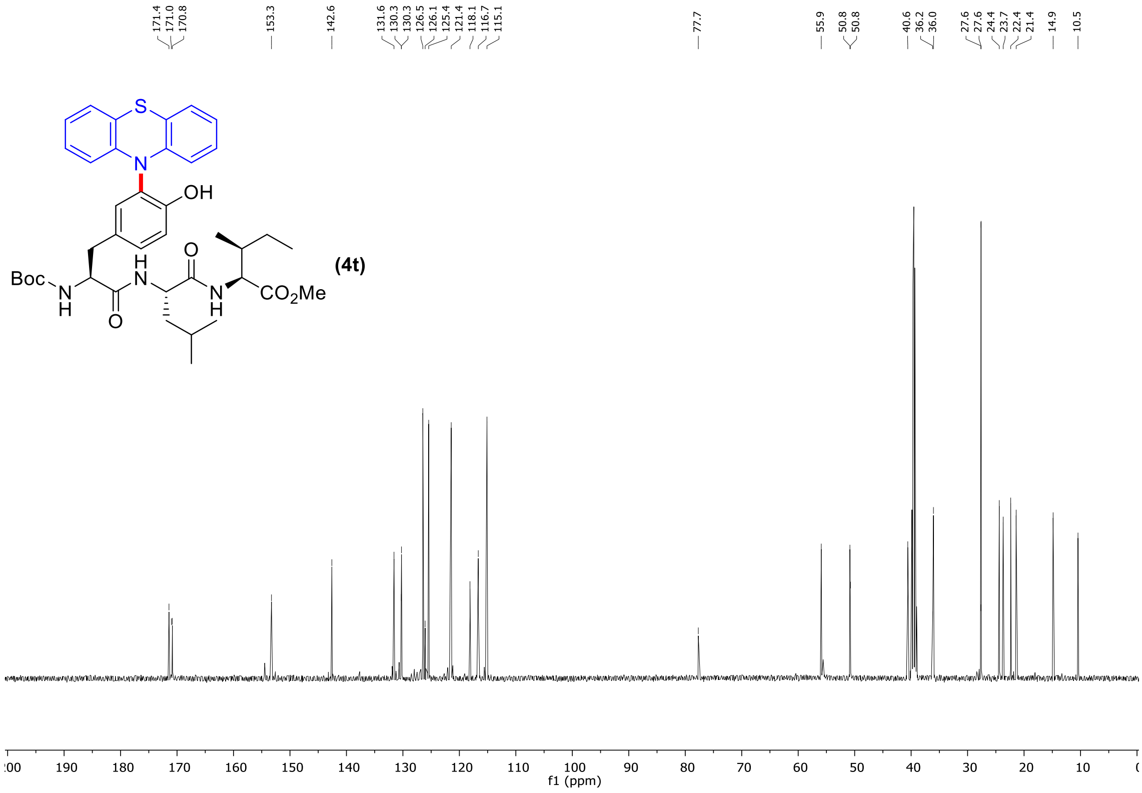

^{1}H NMR (500 MHz, DMSO-d_6 at 80 ºC)

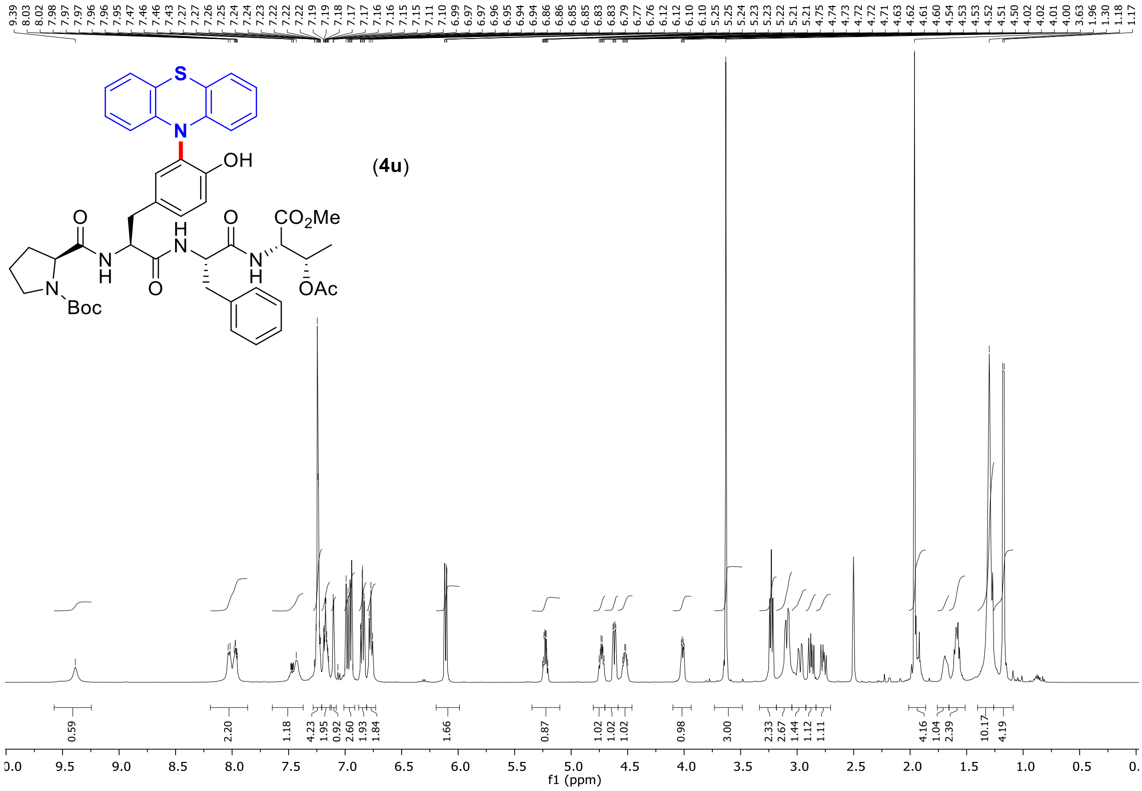

^{13}C NMR (126 MHz, DMSO-d_6 at 80 ºC)

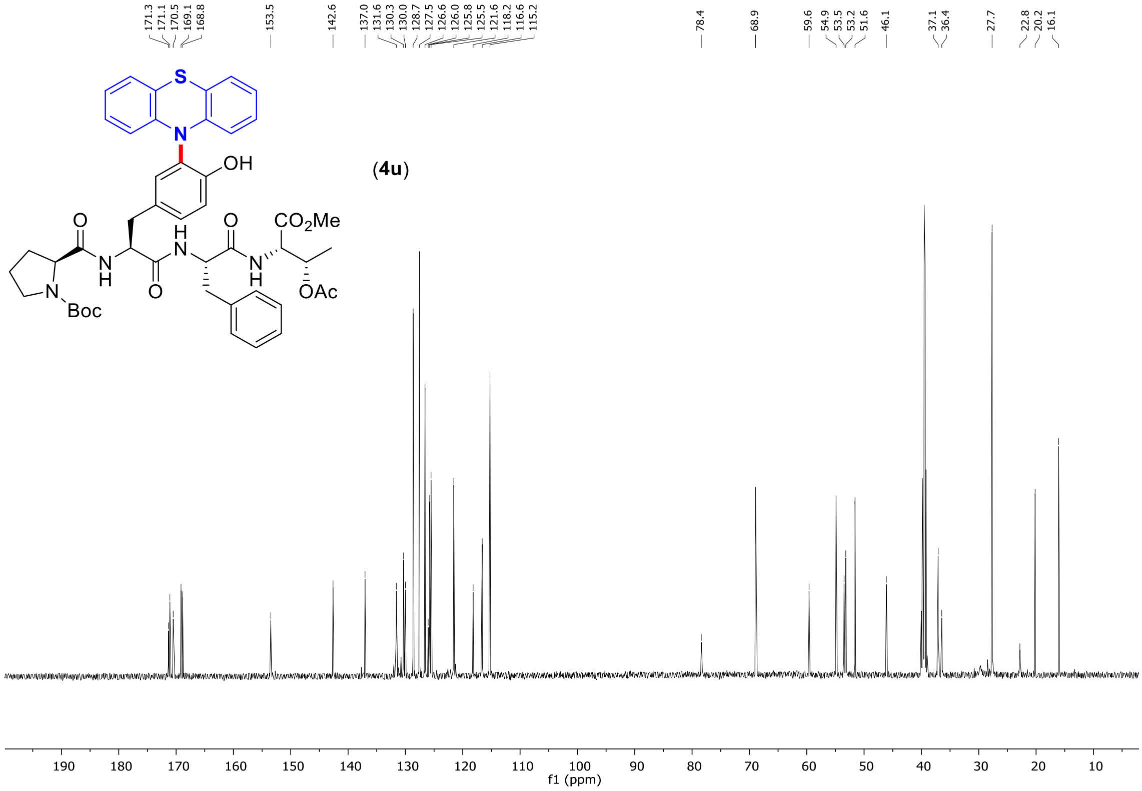

^{1}H NMR (500 MHz, DMSO-d_6 at 80 ºC)

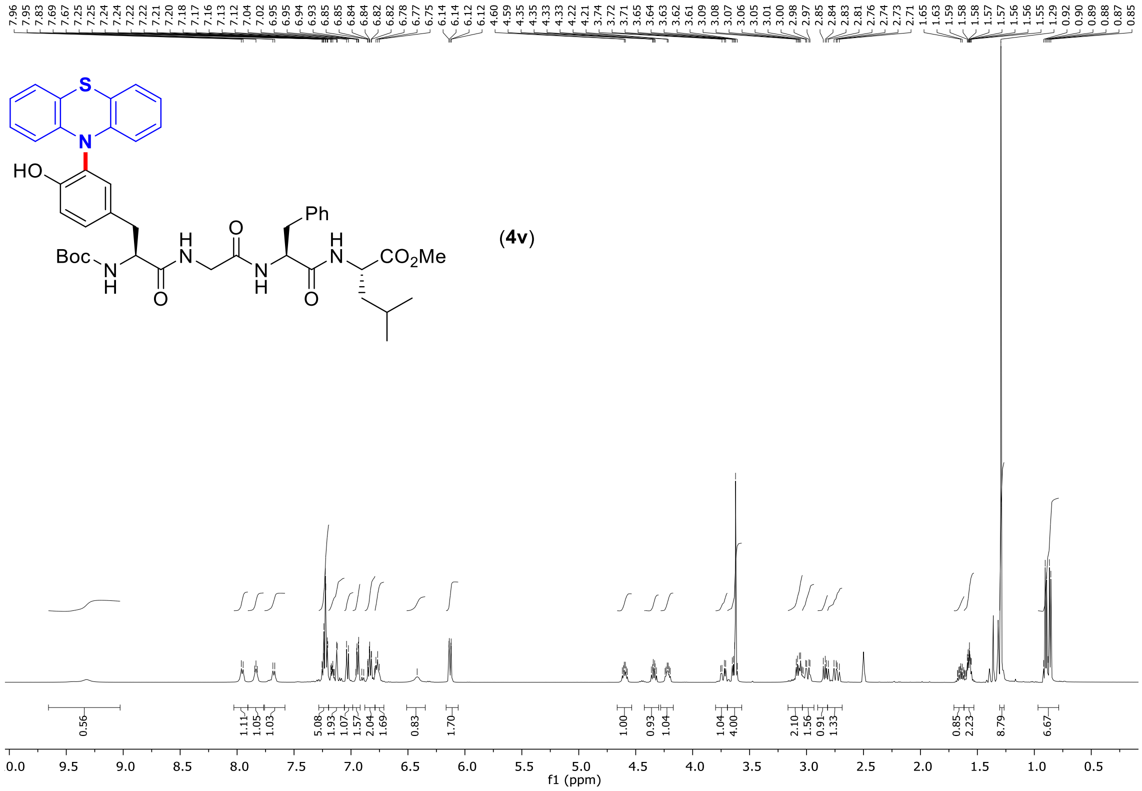

^{13}C NMR (126 MHz, DMSO-d_6 at 80 ºC)

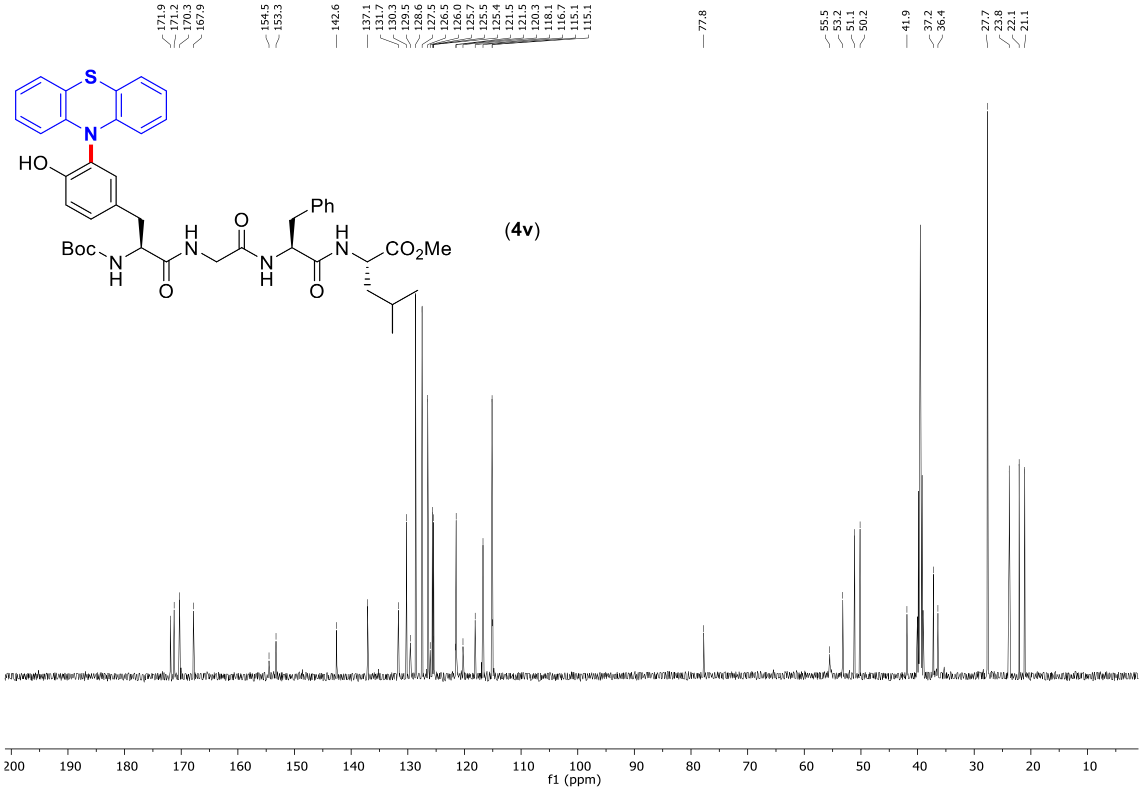

^{1}H NMR (500 MHz, DMSO-d_6 at 80 ºC)

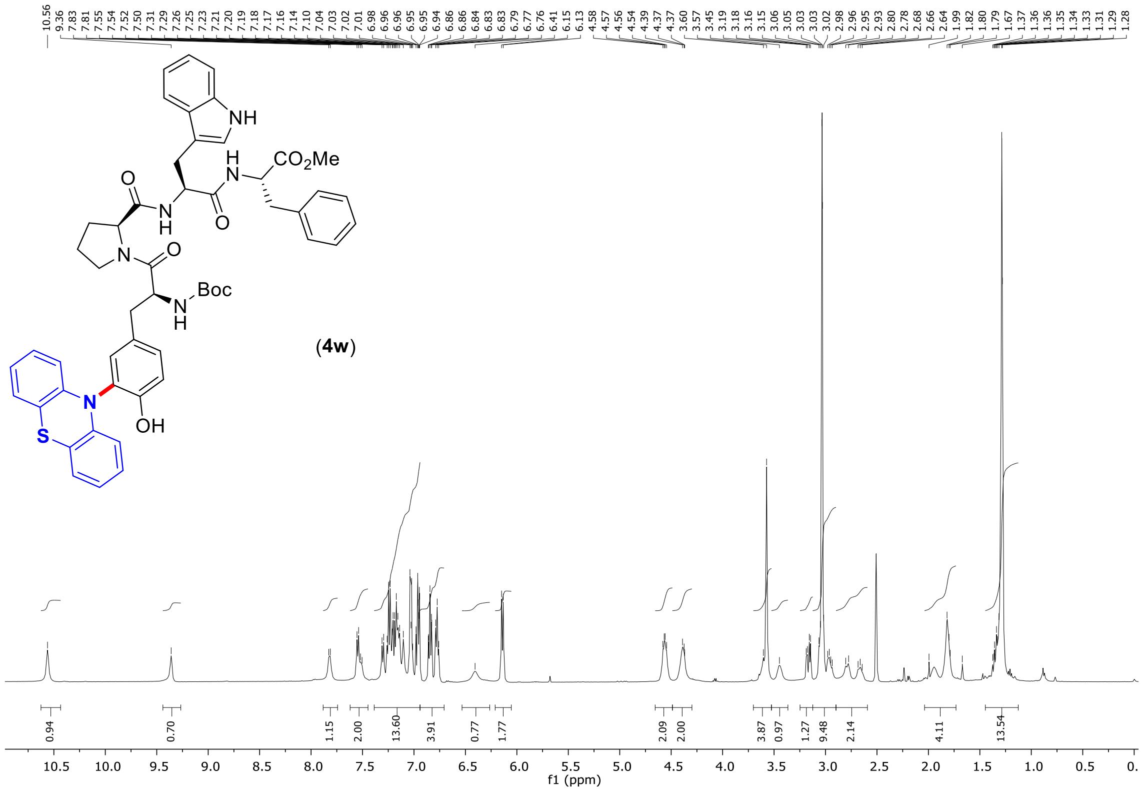

^{13}C NMR (126 MHz, DMSO-d_6 at 80 ºC)

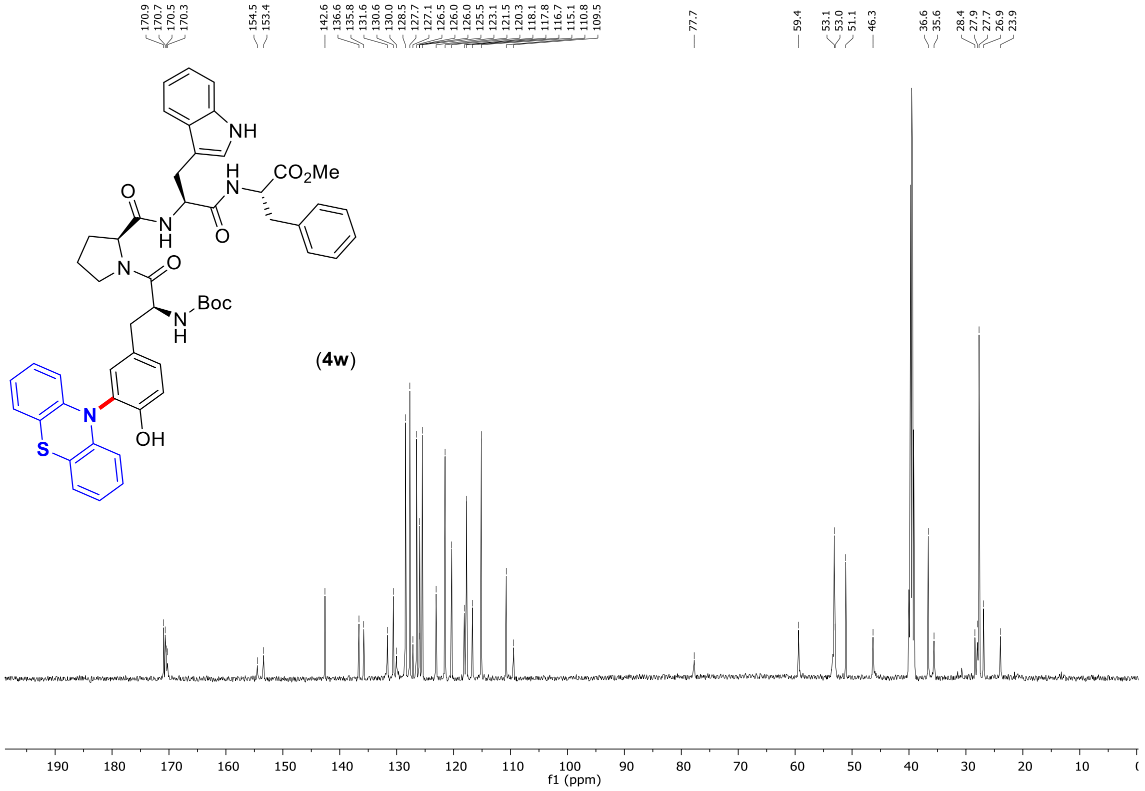

^{1}H NMR (500 MHz, DMSO-d_6 at 80 ºC)

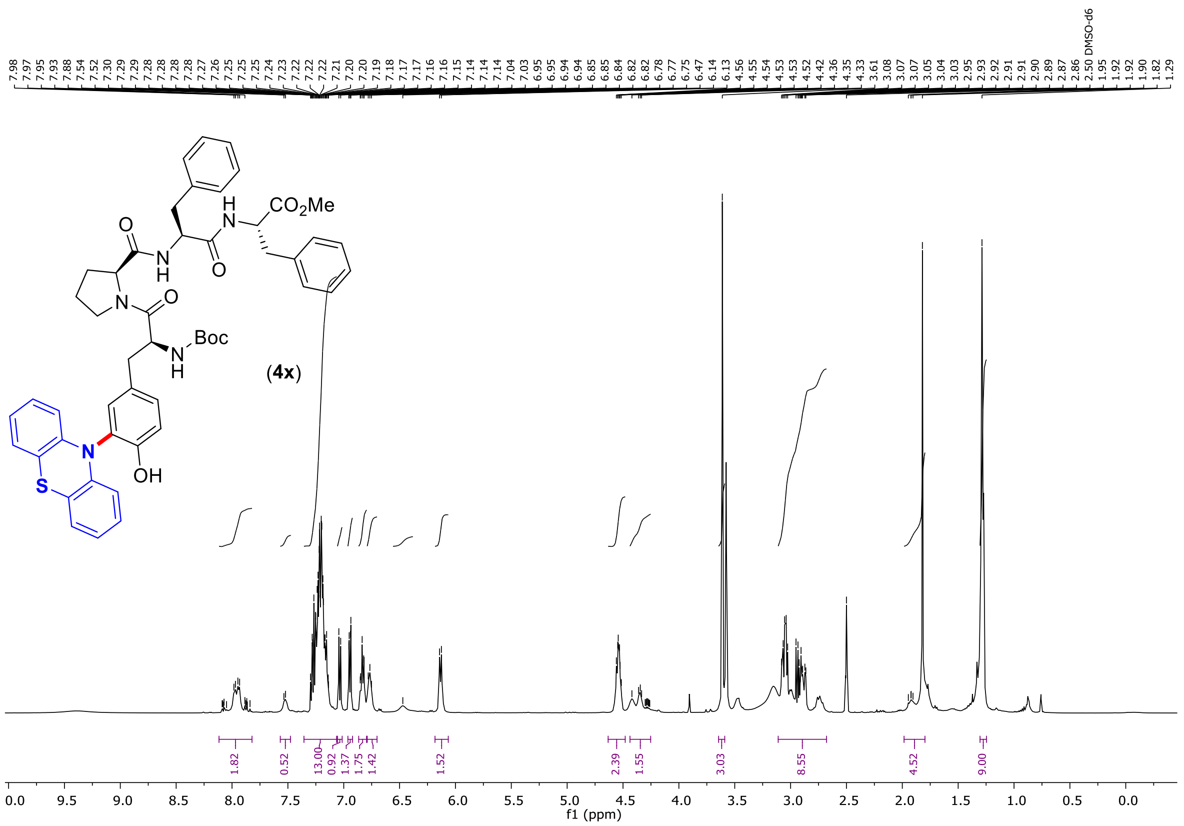

^{13}C NMR (126 MHz, DMSO-d_6 at 80º C)

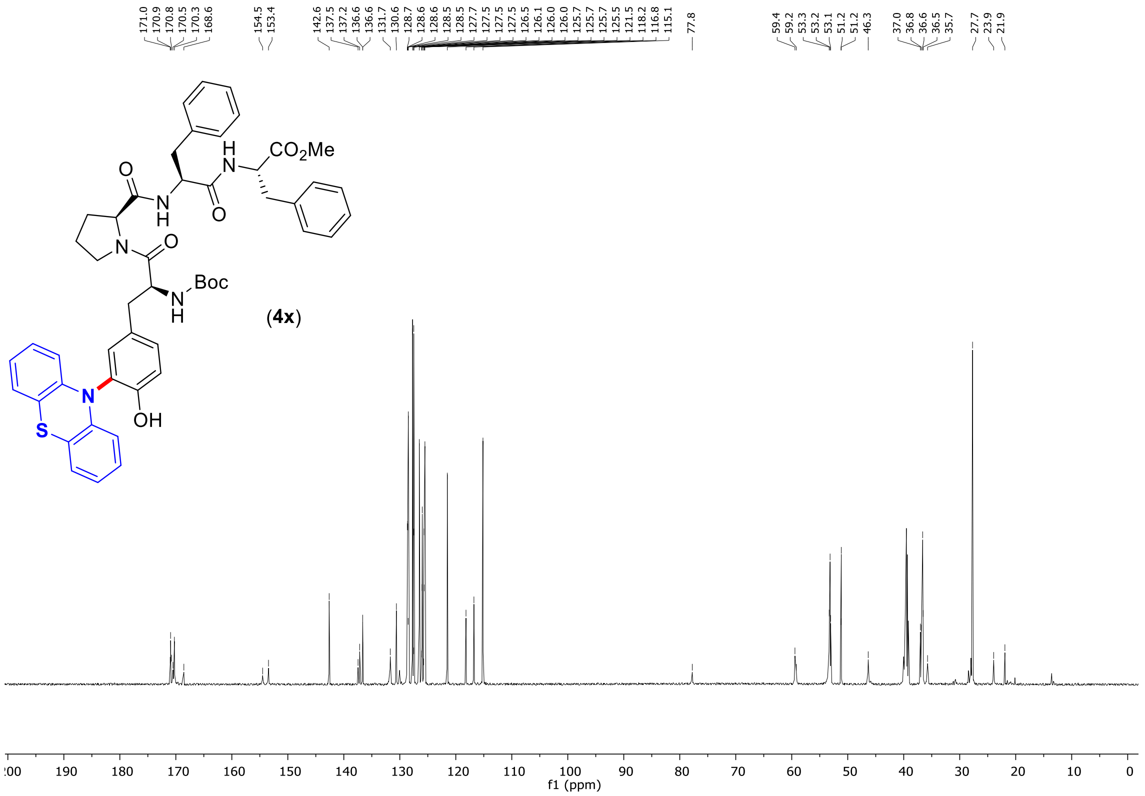

^{1}H NMR (500 MHz, DMSO-d_6 at 80 ºC)

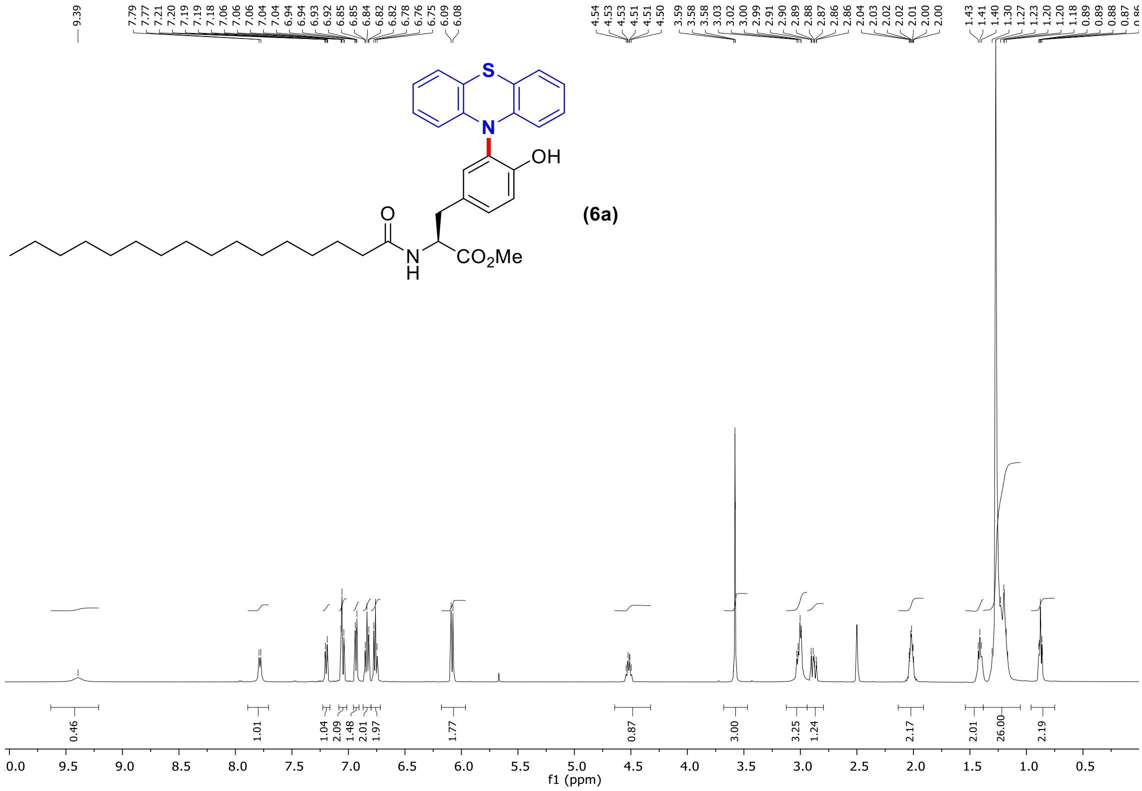

^{13}C NMR (126 MHz, DMSO-d_6 at 80 ºC)

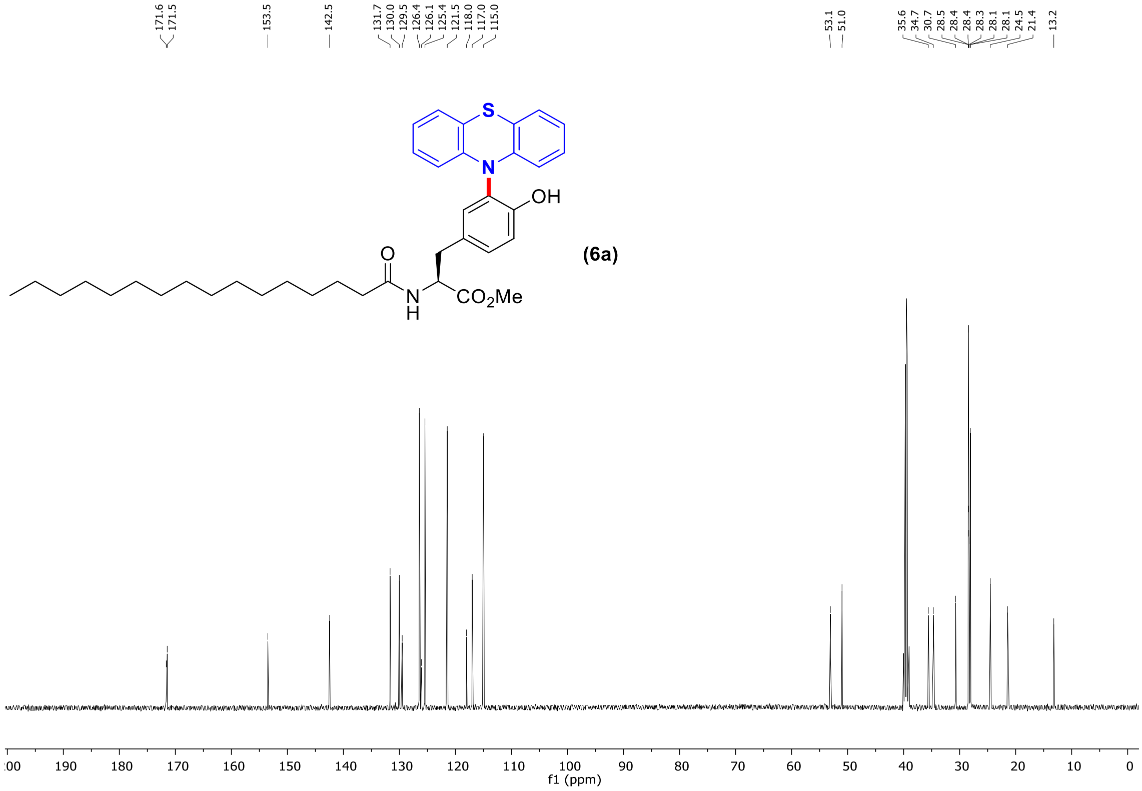

^{1}H NMR (500 MHz, DMSO-d_6 at 80 ºC)

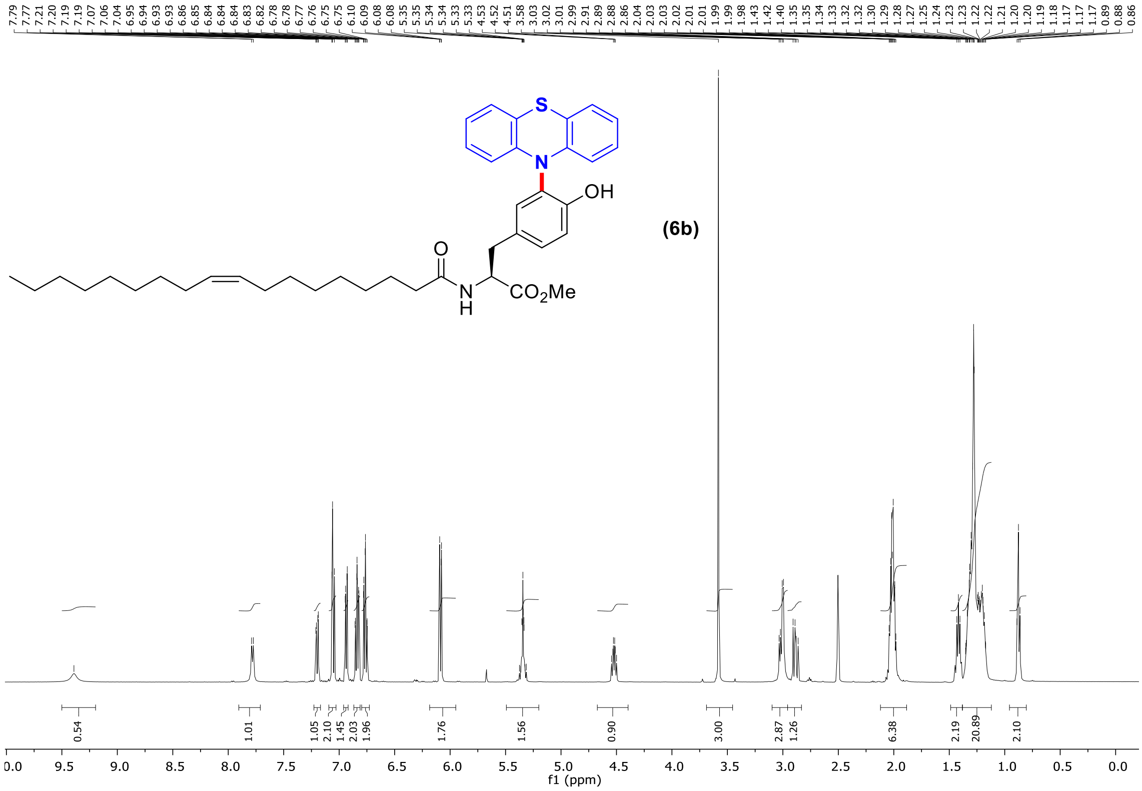

^{13}C NMR (126 MHz, DMSO-d_6 at 80 ºC)

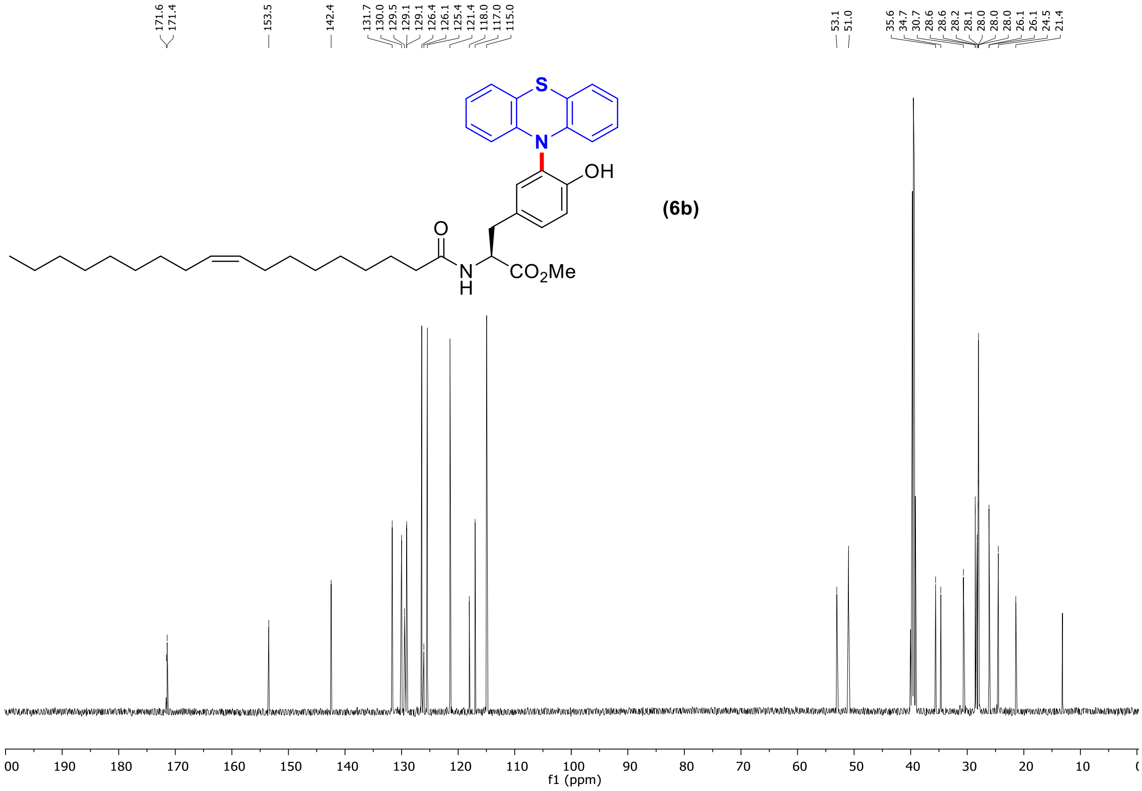

^{1}H NMR (500 MHz, DMSO-d_6 at 80 ºC)

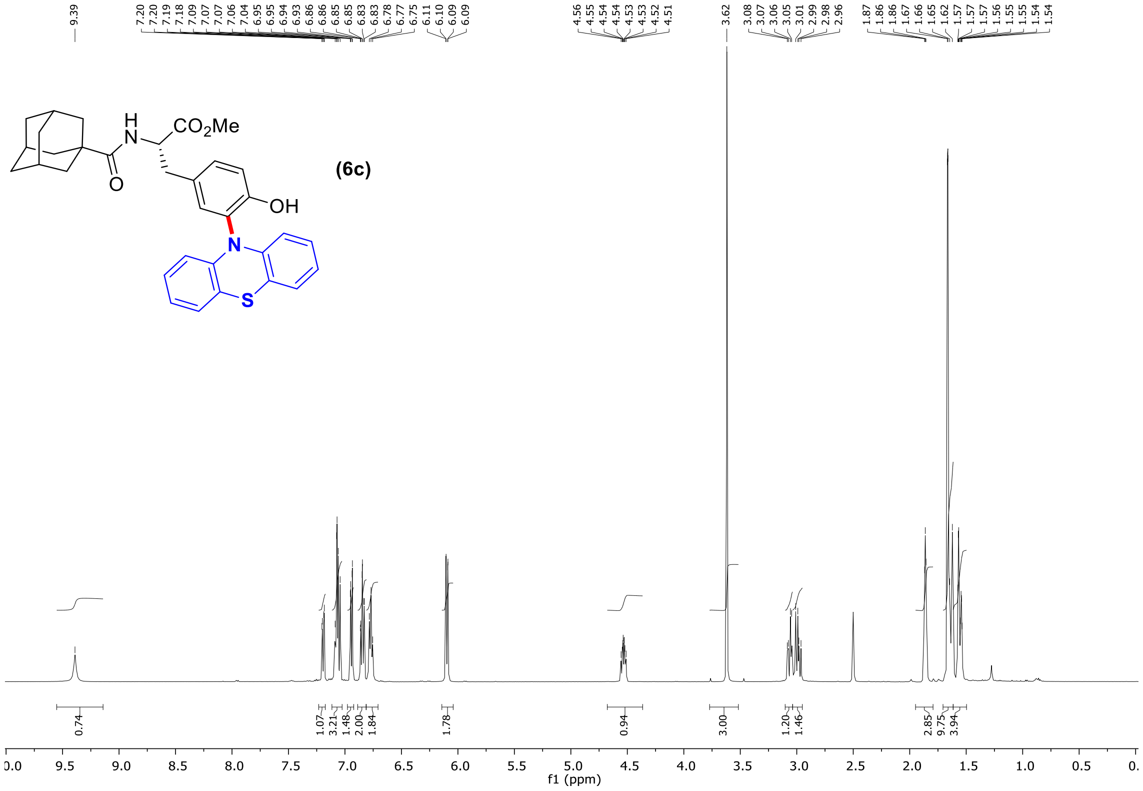

^{13}C NMR (126 MHz, DMSO-d_6 at 80 ºC)

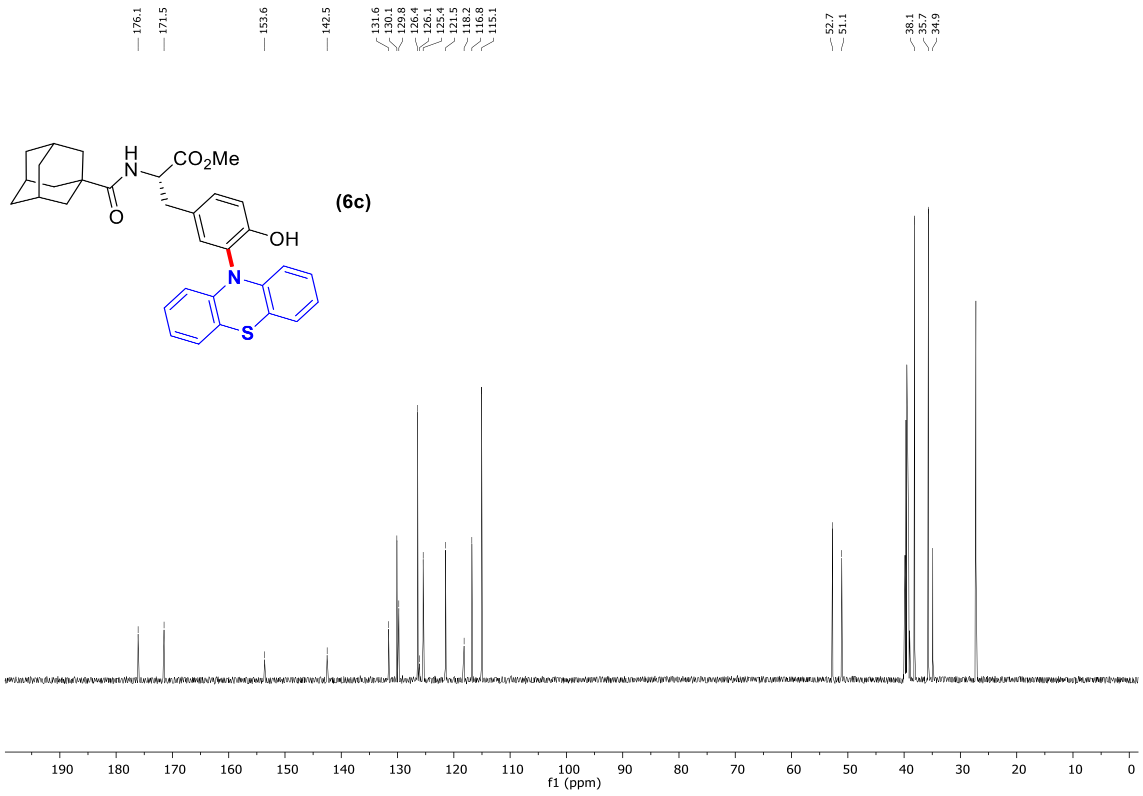

^{1}H NMR (500 MHz, DMSO-d_6 at 80 ºC)

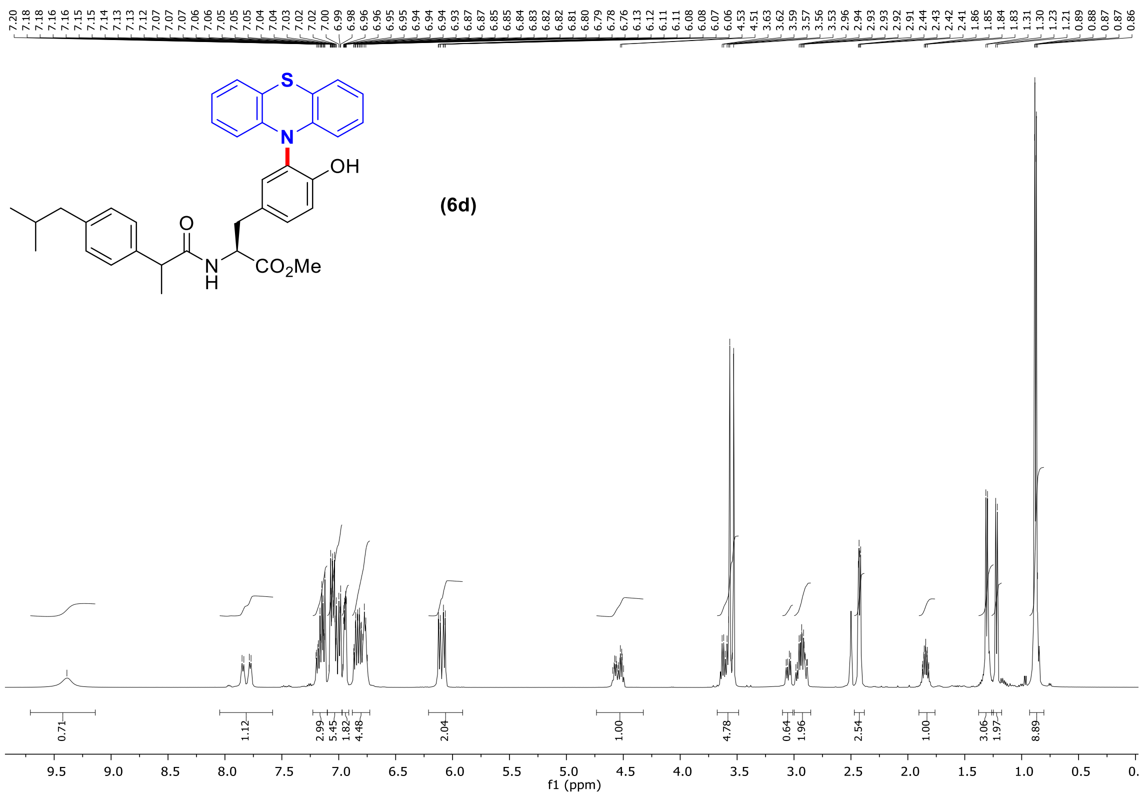

^{13}C NMR (126 MHz, DMSO-d_6 at 80 ºC)

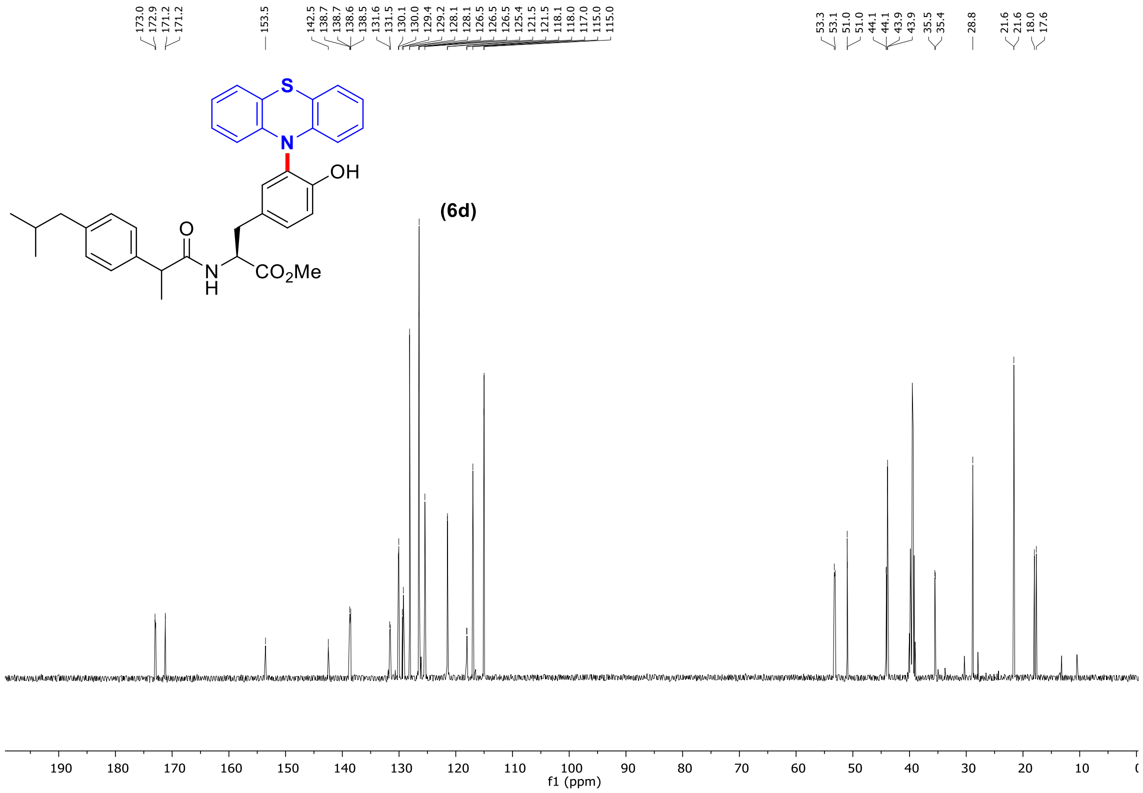

^{1}H NMR (500 MHz, DMSO-d_6 at 80 ºC)

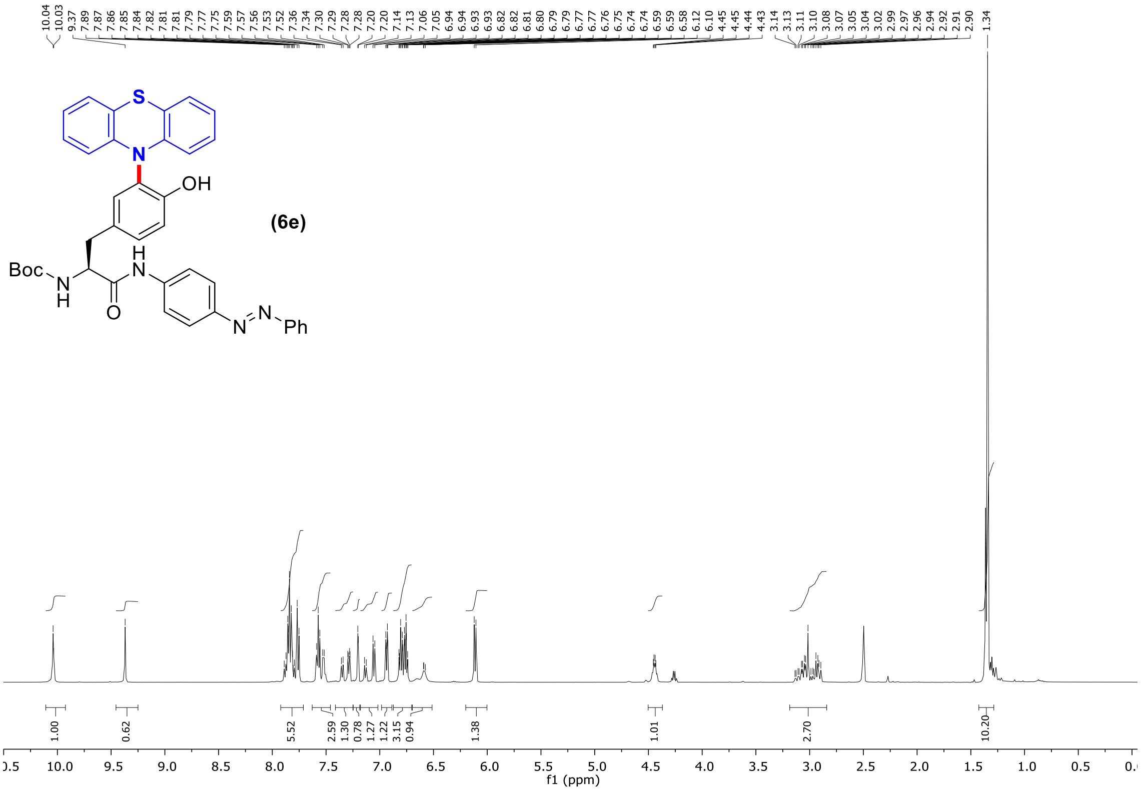

^{13}C NMR (126 MHz, DMSO-d_6 at 80 ºC)

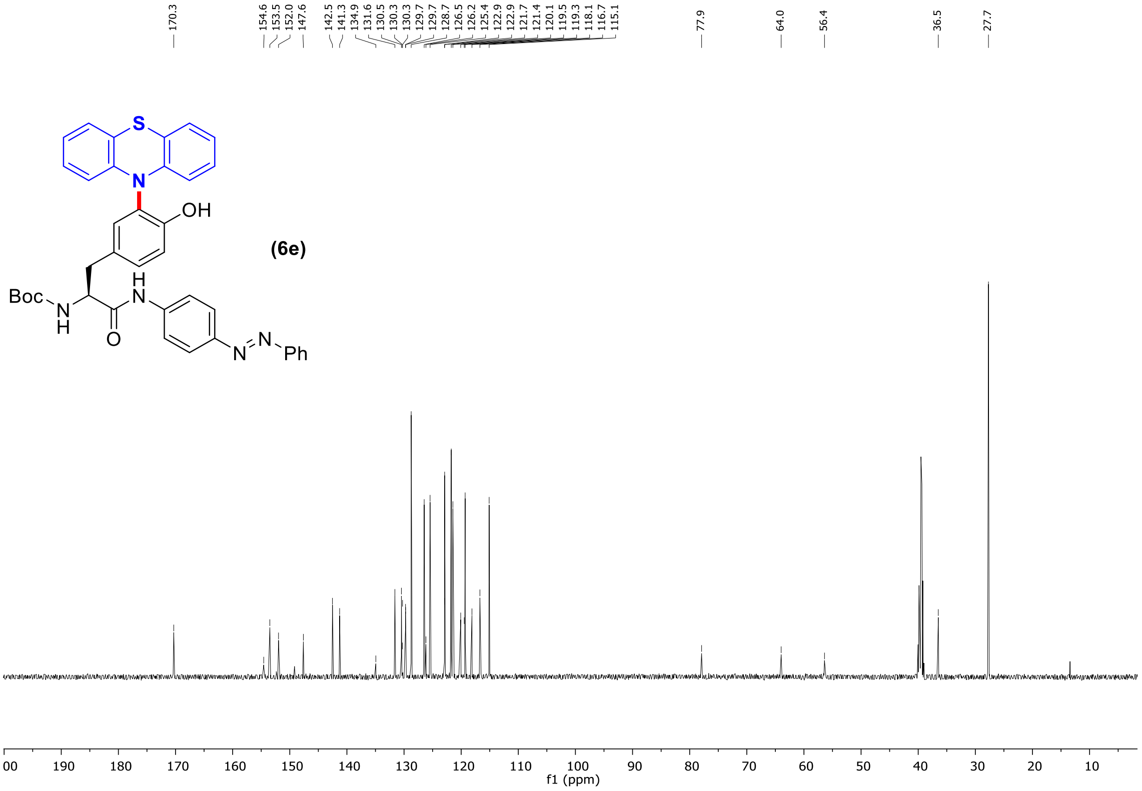

^{1}H NMR (500 MHz, DMSO-d_6 at 80 ºC)

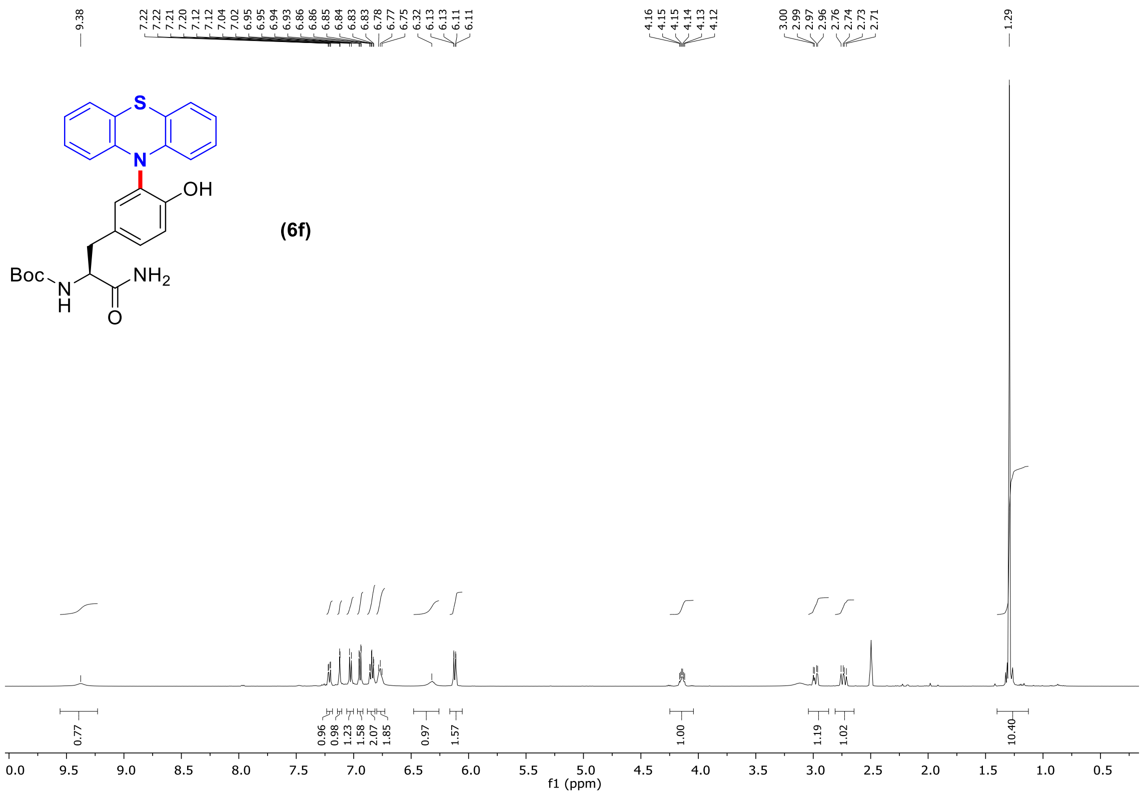

^{13}C NMR (126 MHz, DMSO-d_6 at 80 ºC)

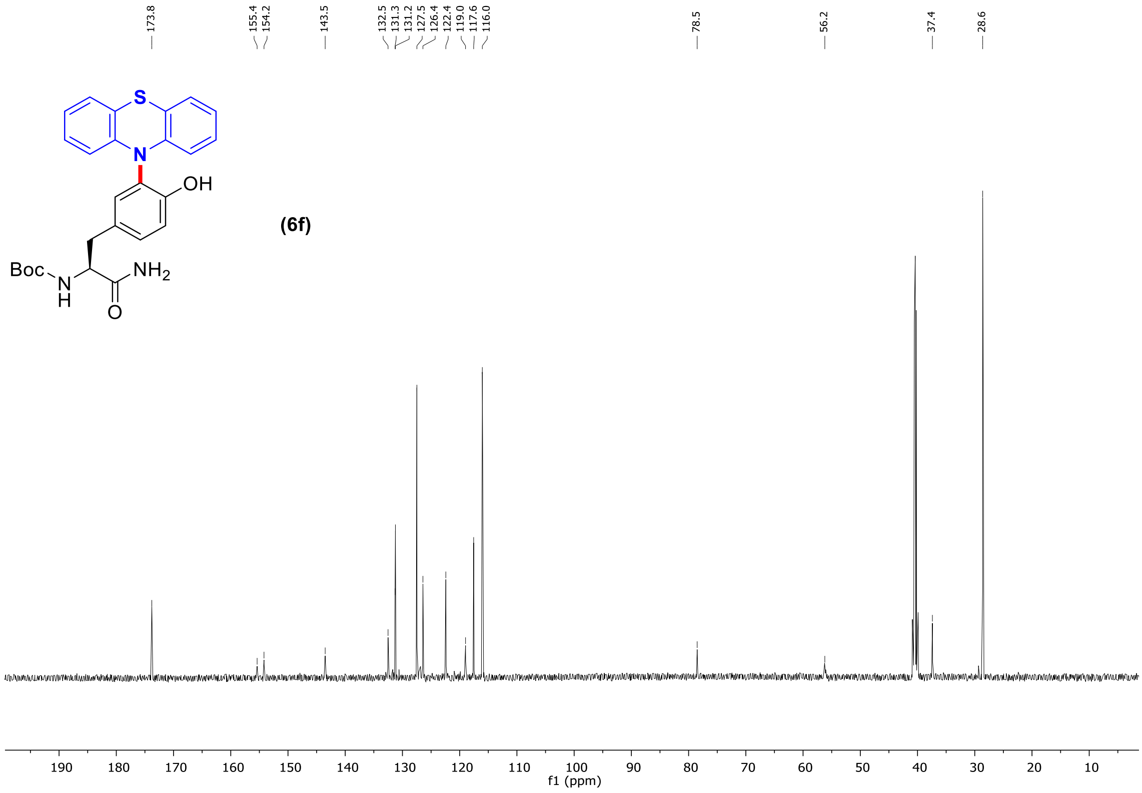

^{1}H NMR (500 MHz, DMSO-d_6 at 80 ºC)

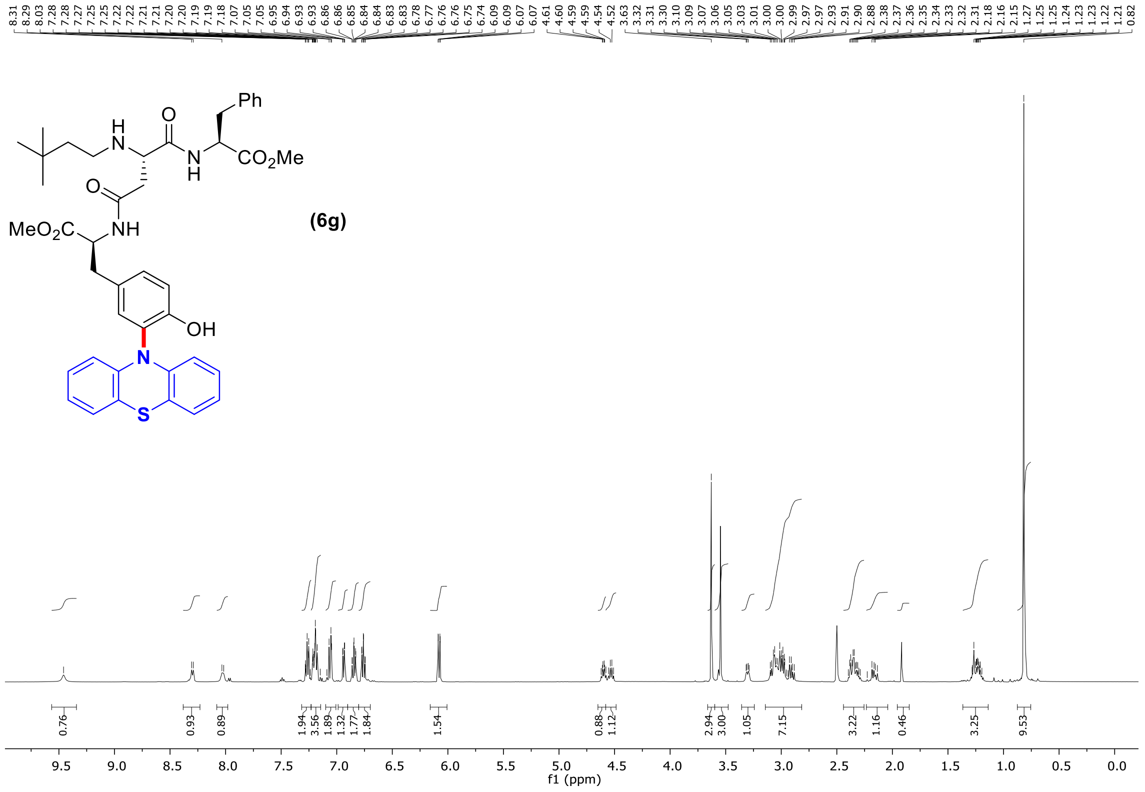

^{13}C NMR (126 MHz, DMSO-d_6 at 80 ºC)

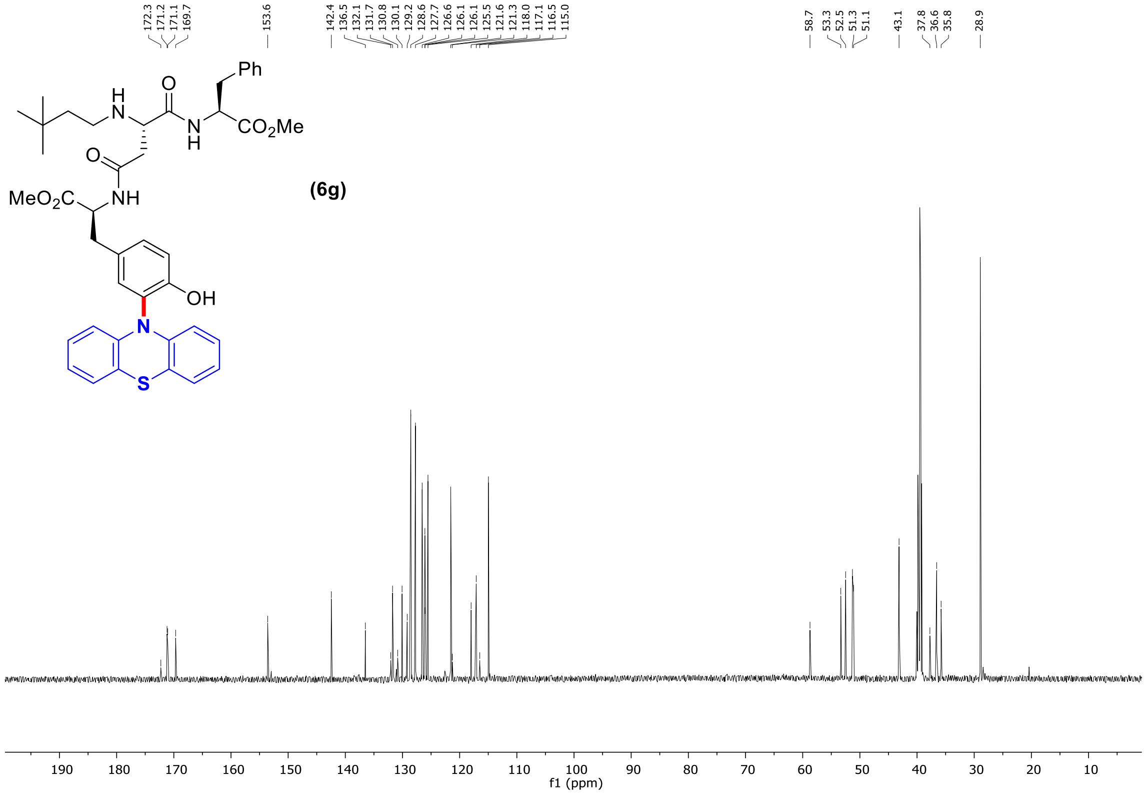

^{1}H NMR (500 MHz, DMSO-d_6 at 80 ºC)

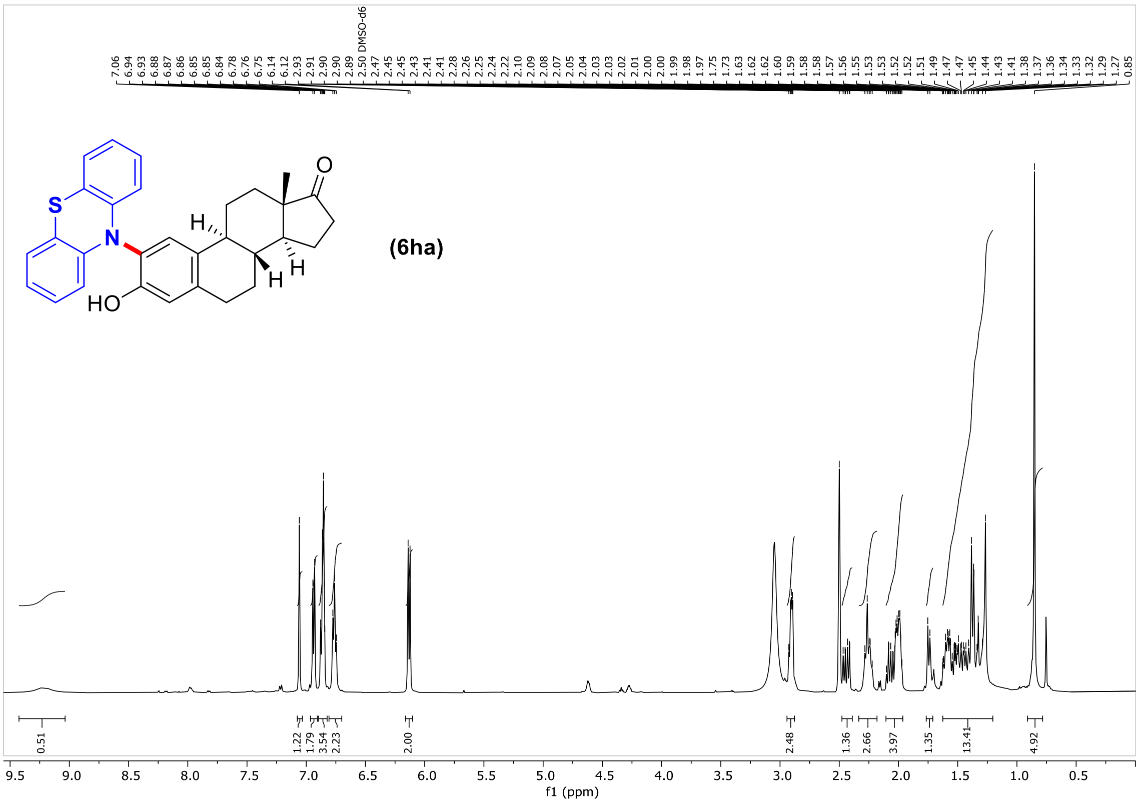

^{13}C NMR (126 MHz, DMSO-d_6 at 80 ºC)

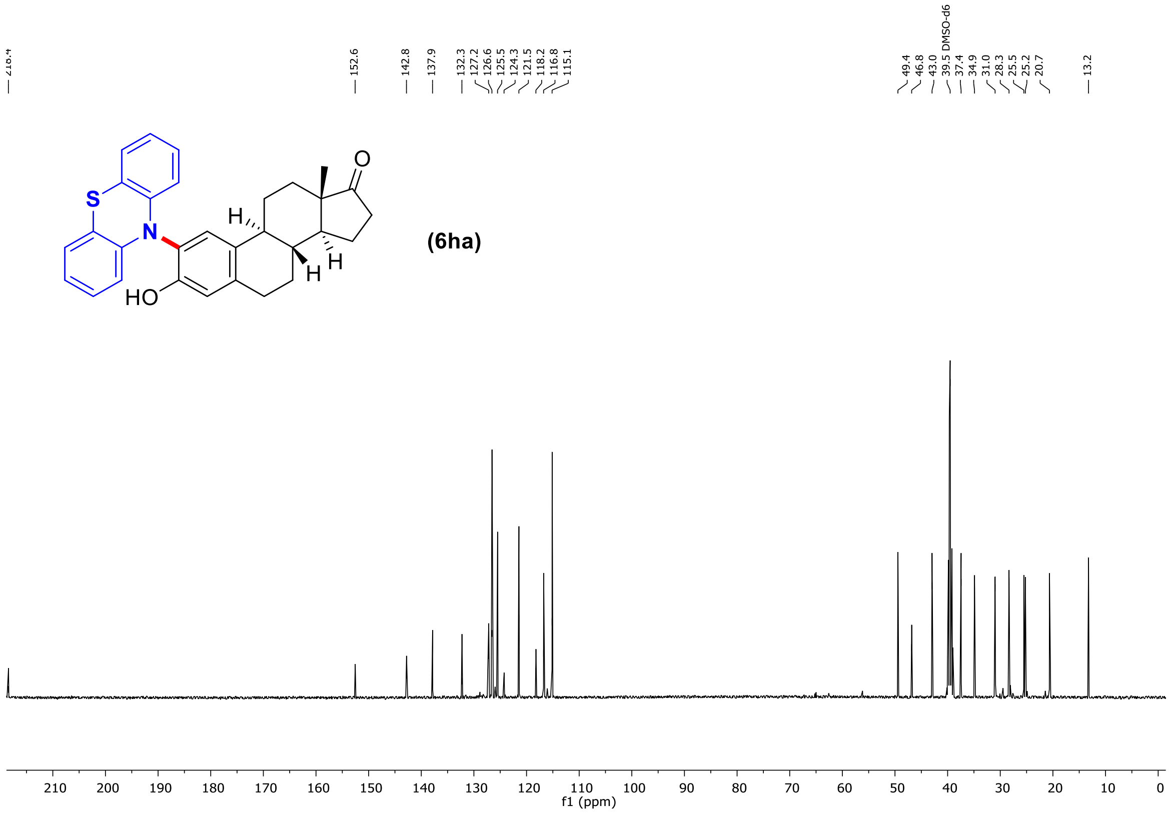

^{1}H NMR (500 MHz, DMSO-d_6 at 80 ºC)

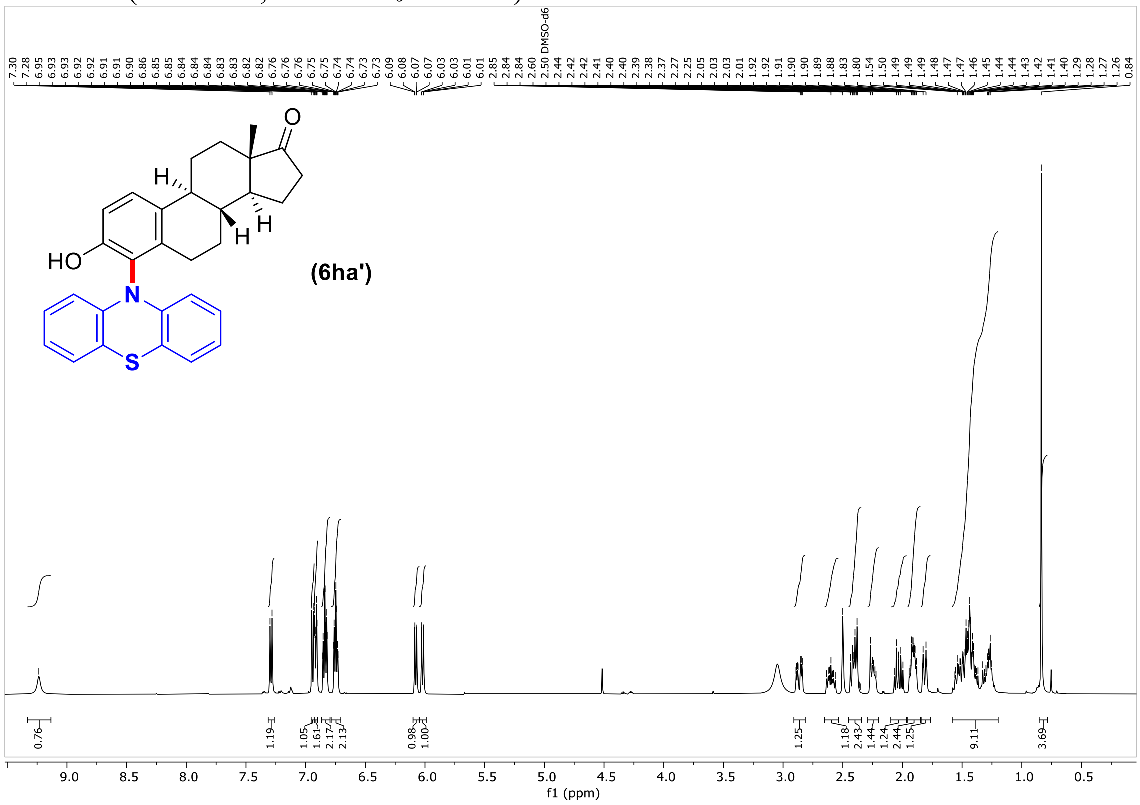

^{13}C NMR (126 MHz, DMSO-d_6 at 80 ºC)

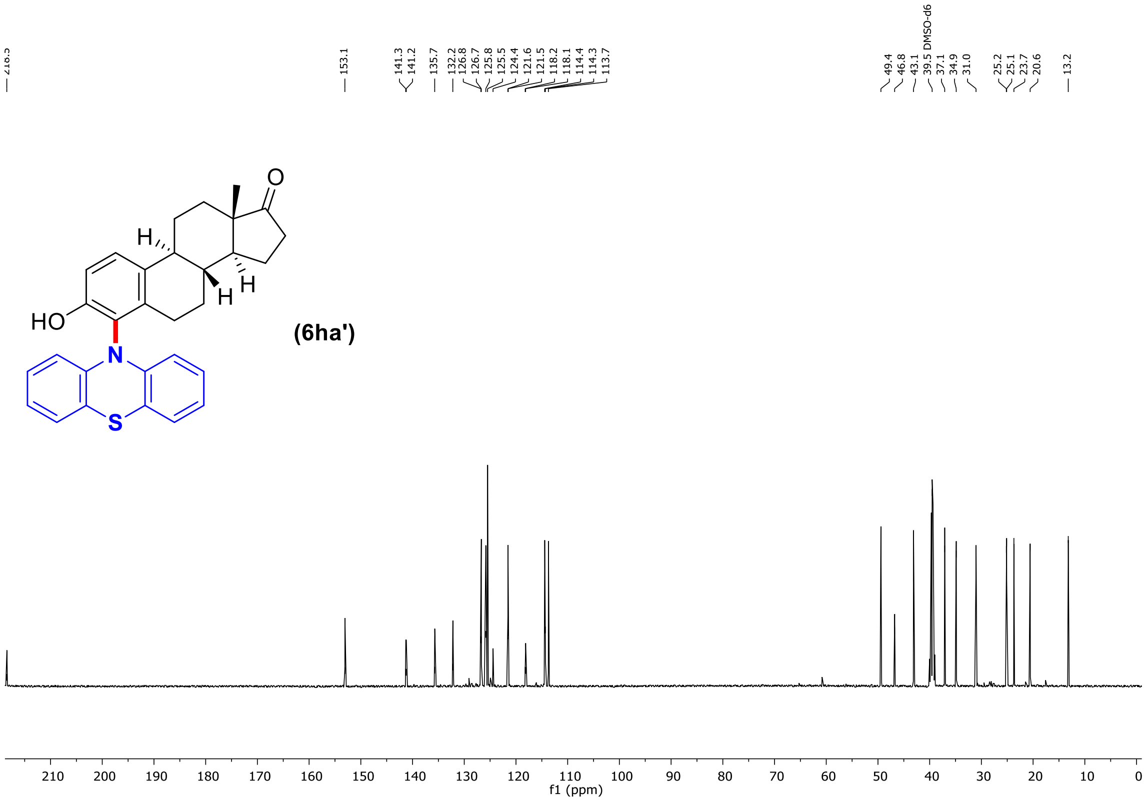

^{1}H NMR (500 MHz, DMSO-d_6 at 80 ºC)

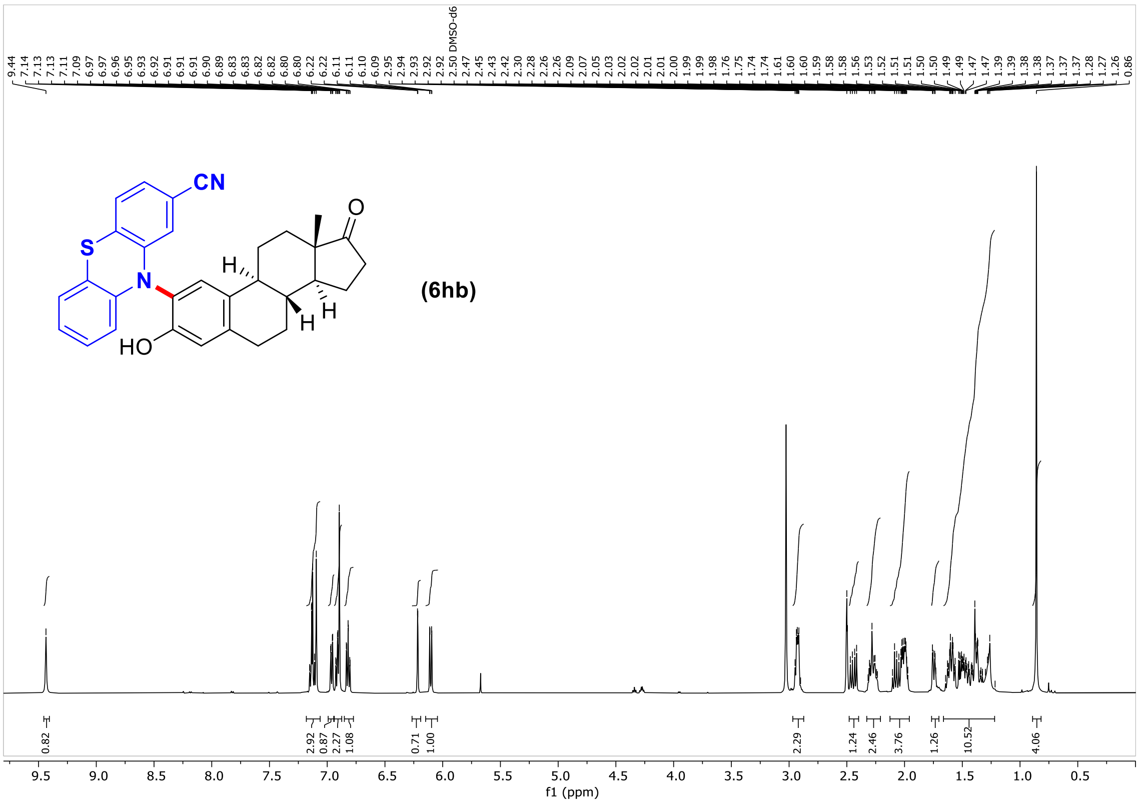

^{13}C NMR (126 MHz, DMSO-d_6 at 80 ºC)

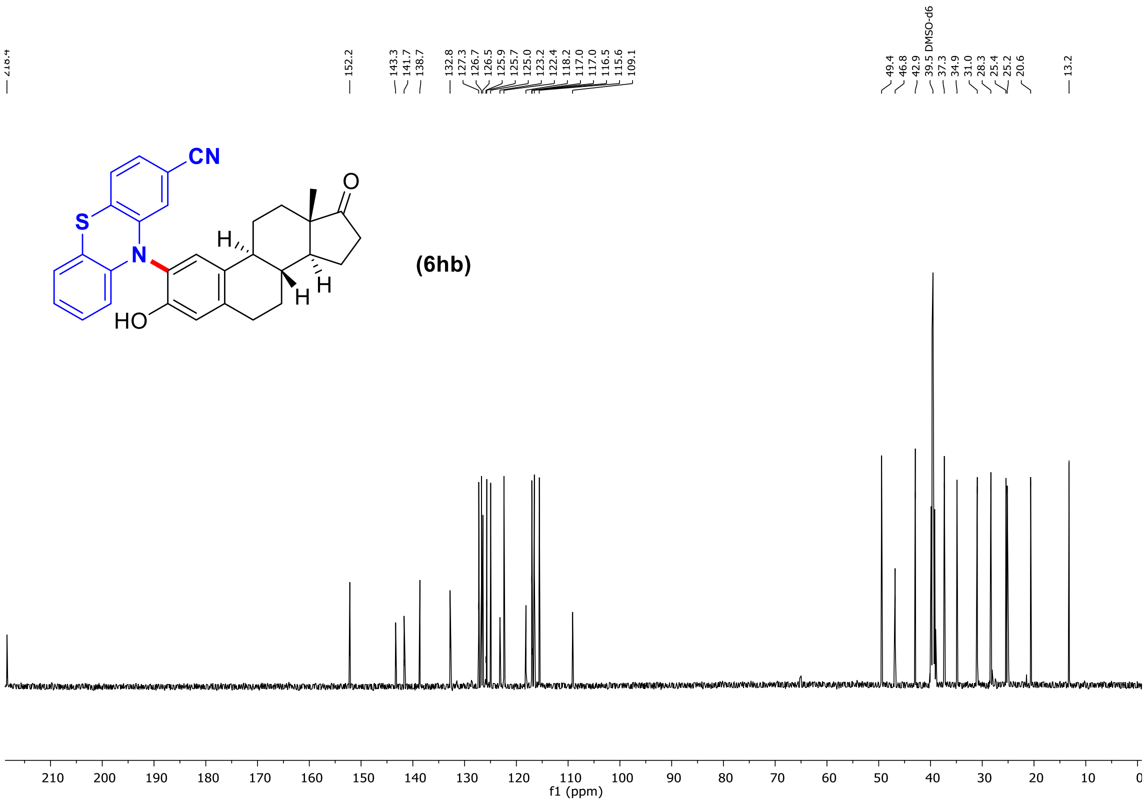

^{1}H NMR (500 MHz, DMSO-d_6 at 80 ºC)

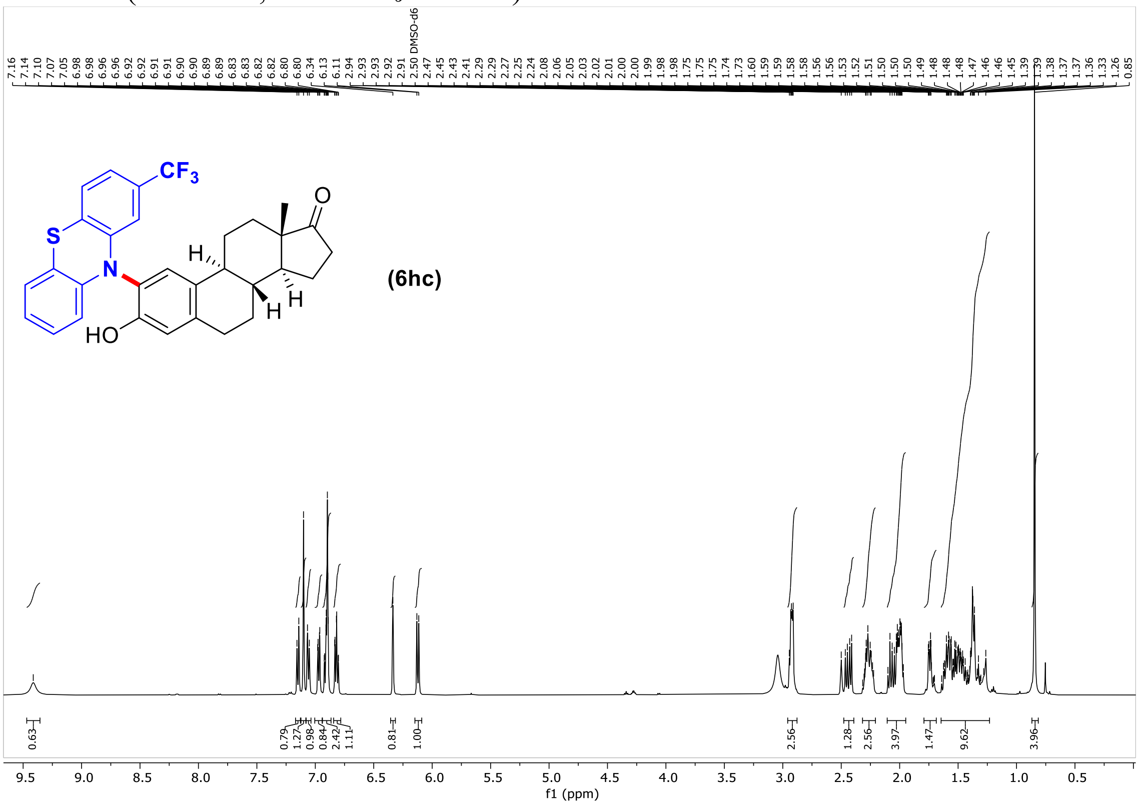

^{13}C NMR (126 MHz, DMSO-d_6 at 80 ºC)

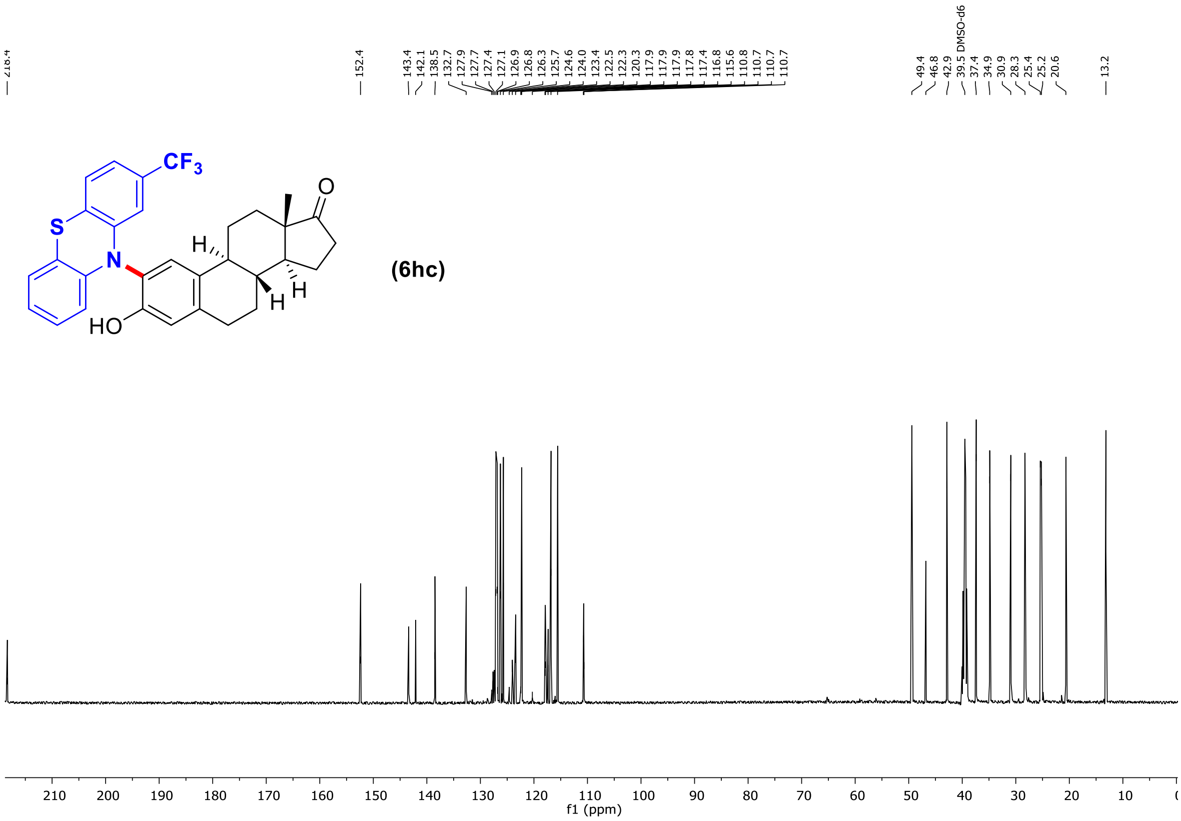

^{1}H NMR (400 MHz, MeOD-d_4)

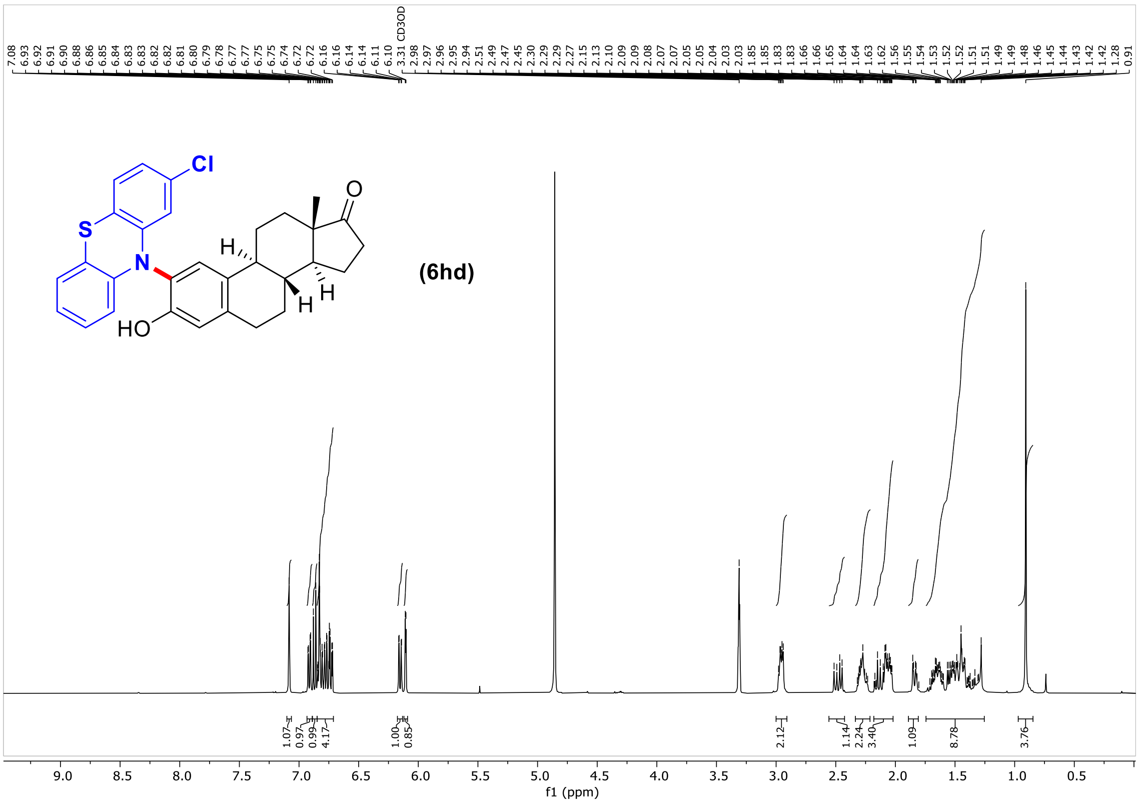

^{13}C NMR (126 MHz, DMSO-d_6 at 80 ºC)

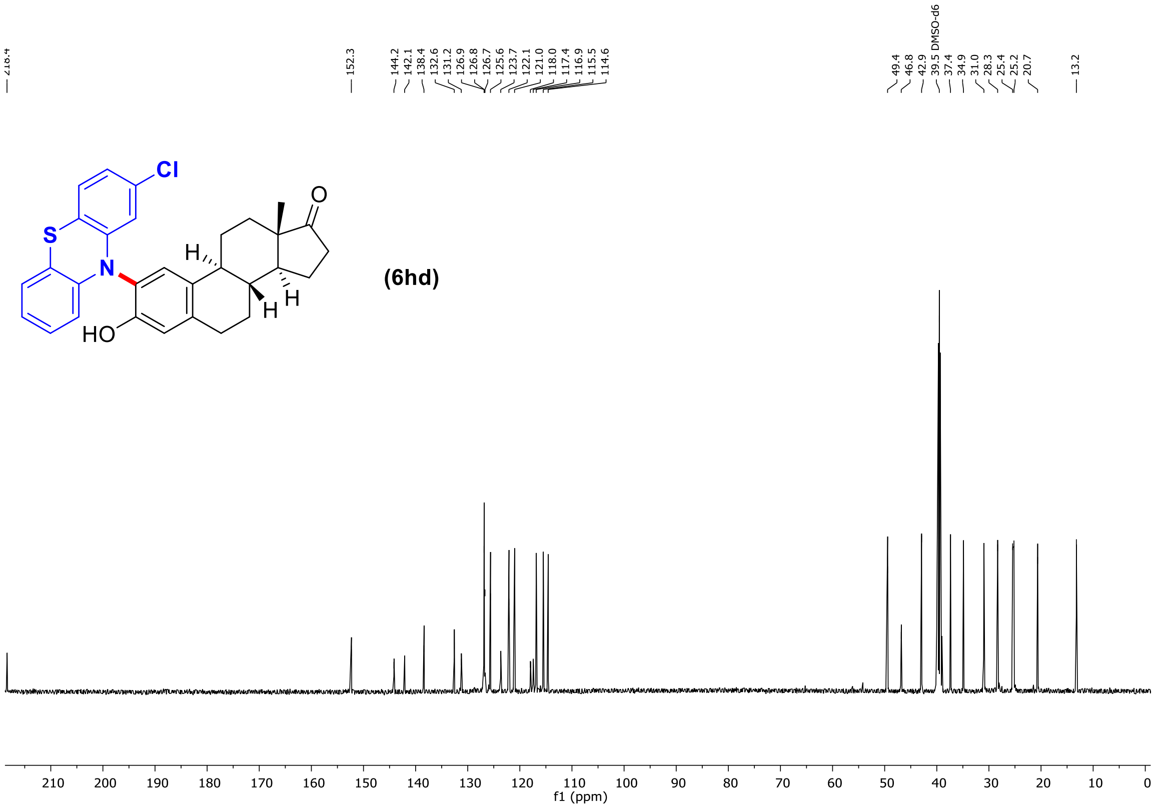

^{1}H NMR (500 MHz, DMSO-d_6 at 80 ºC)

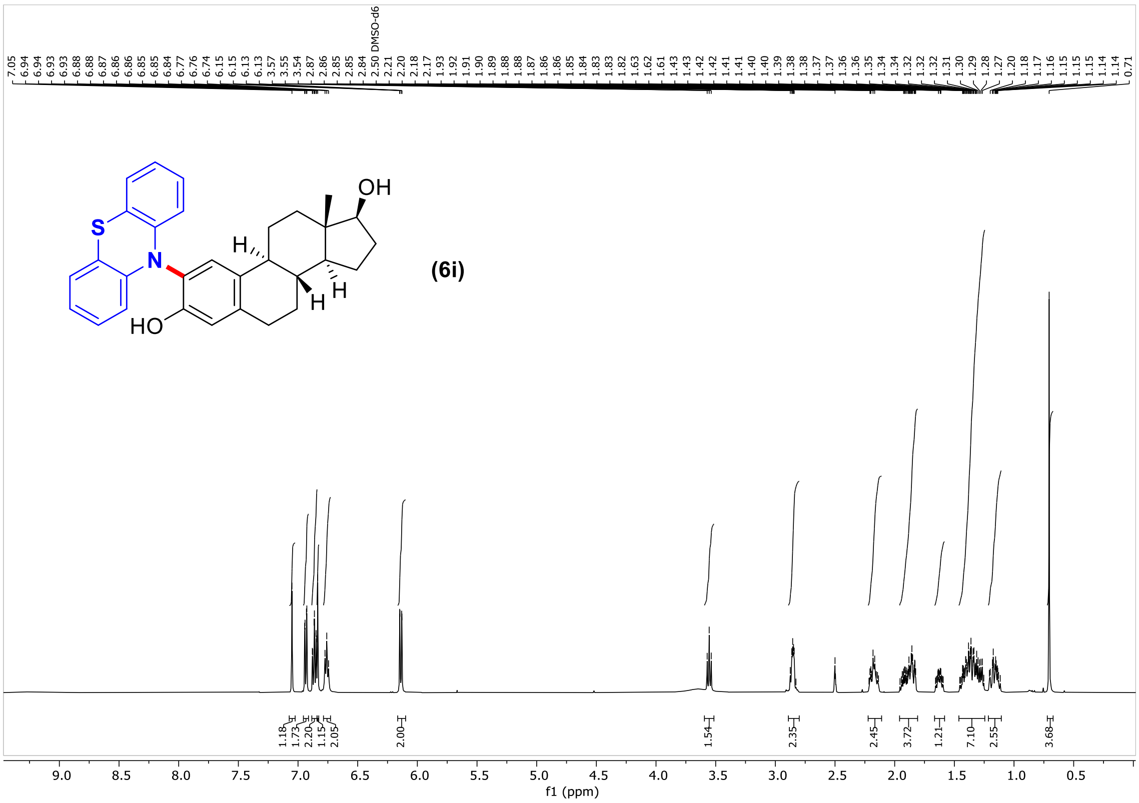

^{13}C NMR (126 MHz, DMSO-d_6 at 80 ºC)

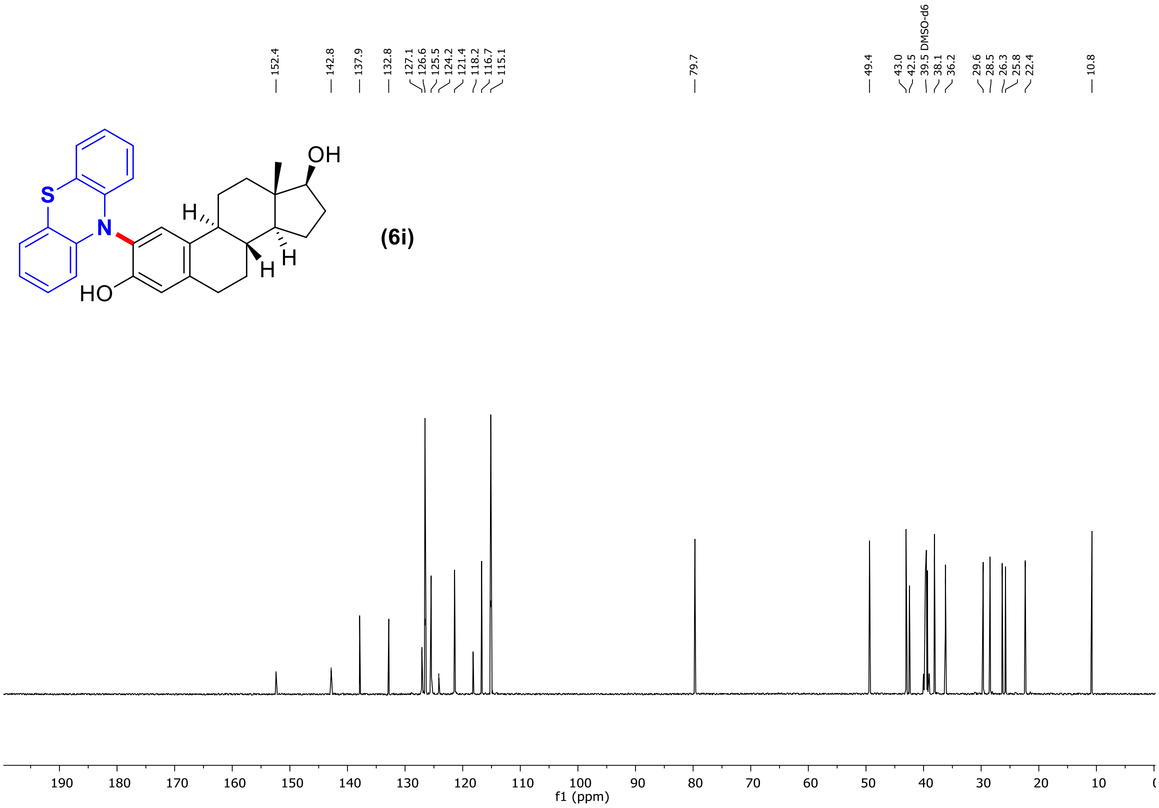

^{1}H NMR (500 MHz, DMSO-d_6 at 80 ºC)

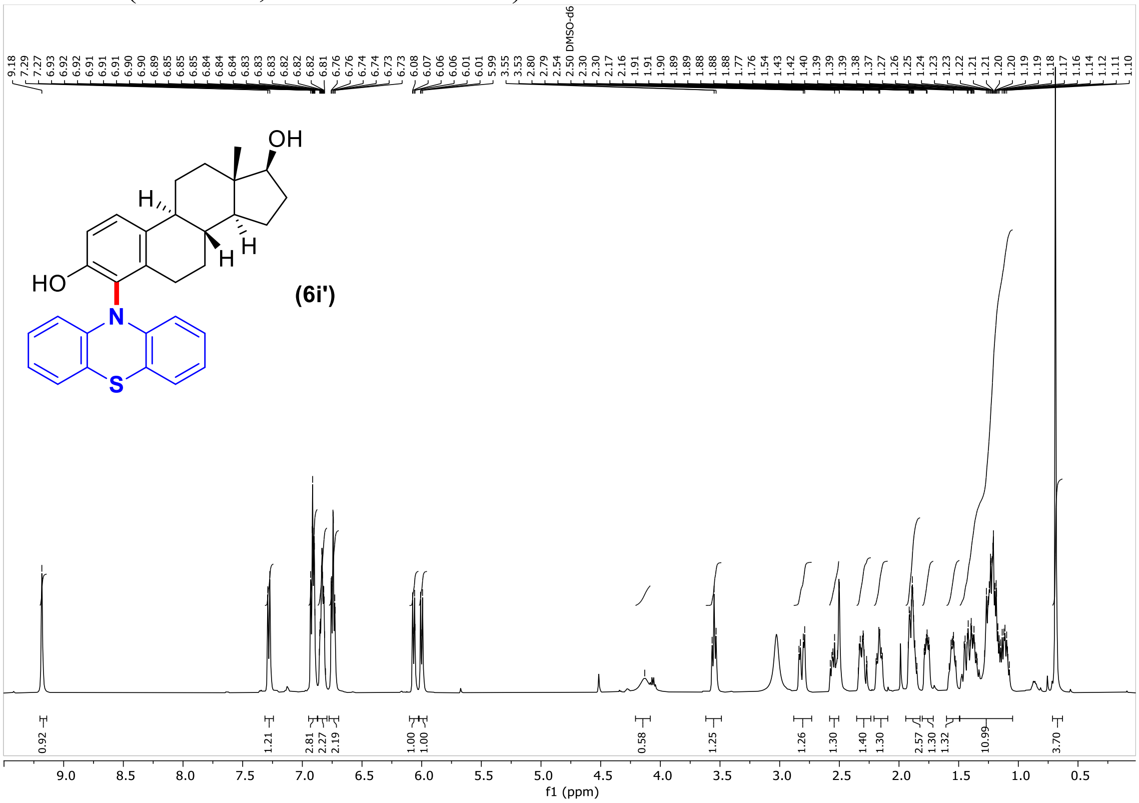

^{13}C NMR (126 MHz, DMSO-d_6 at 80 ºC)

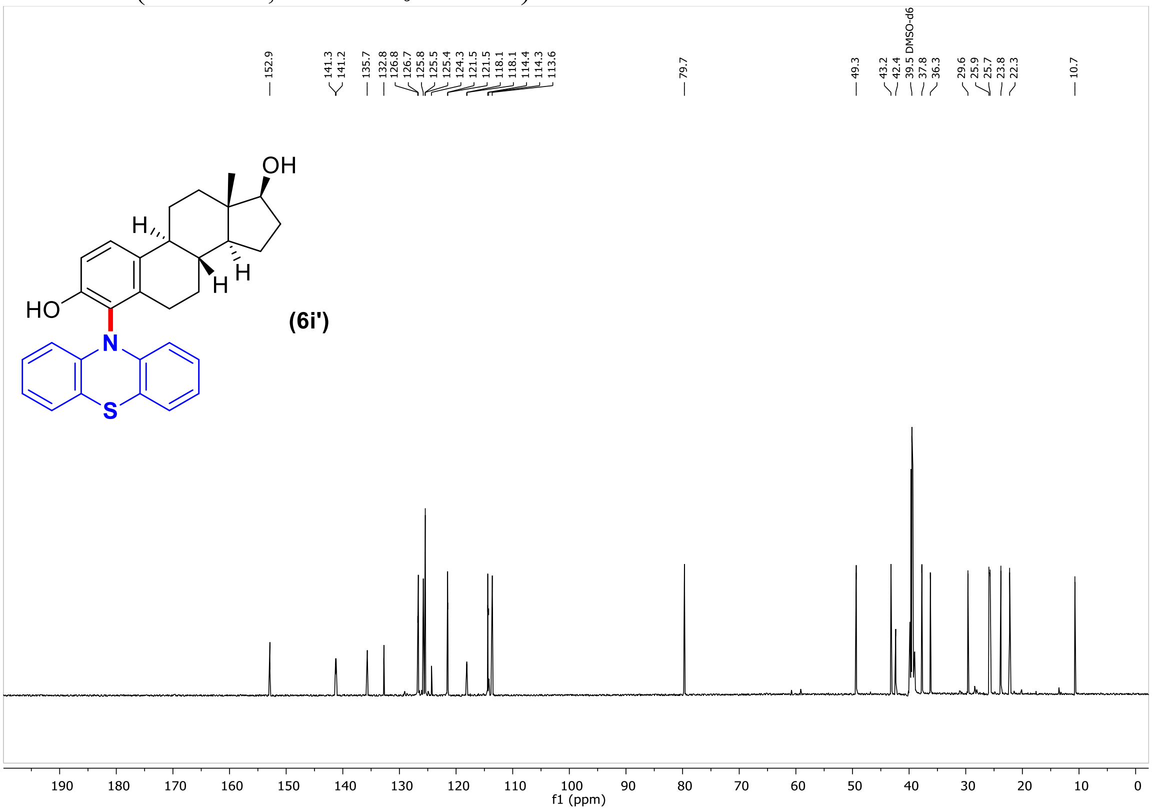

^{19}F NMR (376 MHz, $CDCl_3$)

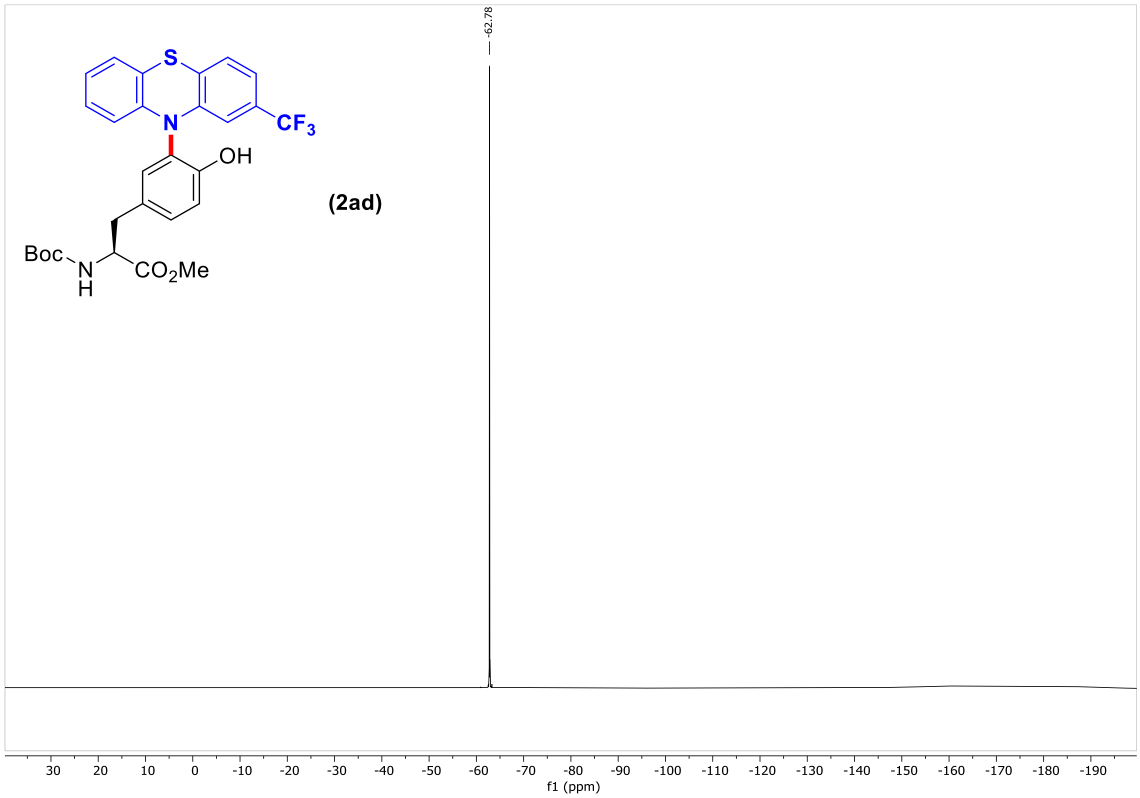

^{19}F NMR (376 MHz, $CDCl_3$)

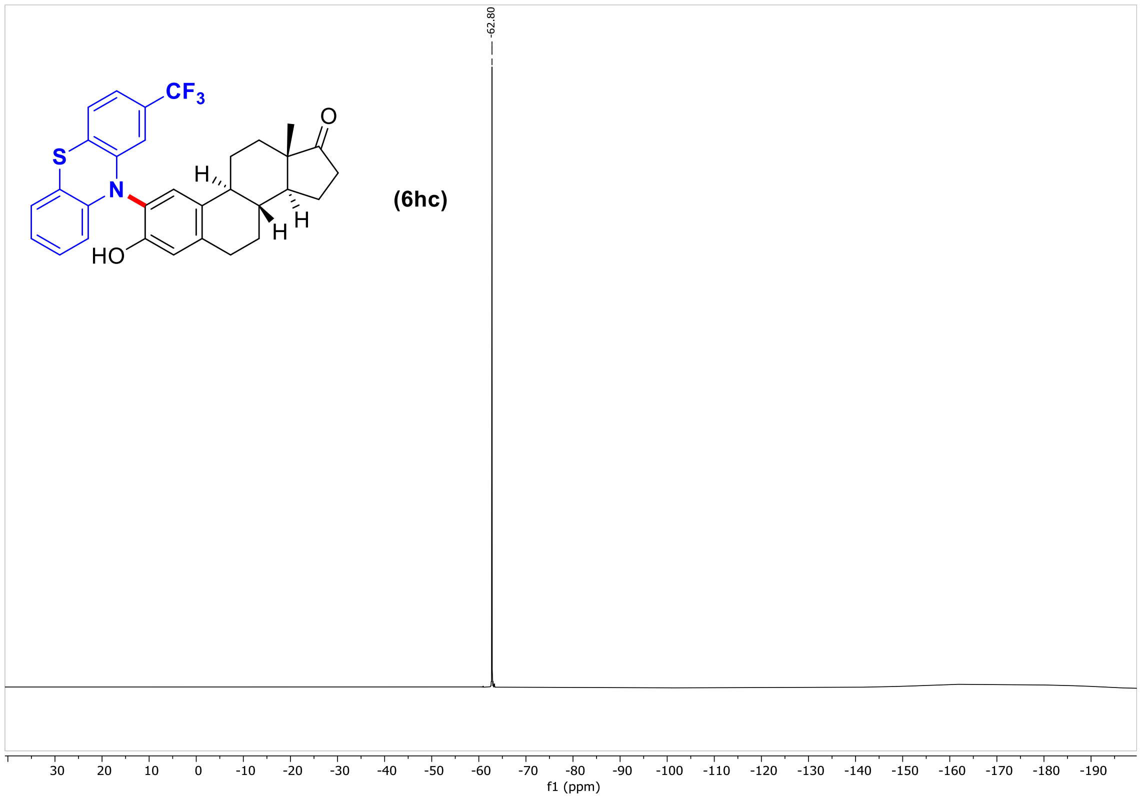

Supplement: Supplementary file 1 — ol3c01560_si_001.pdf [file ol3c01560_si_001.pdf]
